# Supplementary material for: How is maternal employment associated with infant and young child feeding in Bangladesh? A systematic literature review and meta-analysis
Source: PLoS One. 2025 Jan 24;20(1):e0316436. doi: 10.1371/journal.pone.0316436 (PMC11759378; doi:10.1371/journal.pone.0316436)
Supplement: S1 Appendix — (PDF) [file pone.0316436.s001.pdf]

Supporting information: S1 Appendix

How is maternal employment associated with infant and young child feeding in Bangladesh? A systematic literature review and meta-analysis

Contents

1. Quality assessment of the included articles 2
Table s1.1: Quality of the included cross-sectional studies 2
Table s1.2: Quality of the included cohort or longitudinal study 3
2. Leave-one-out meta-analysis 4
Fig s2.1: Leave-one-out meta-analysis for EIBF indicator 4
Fig s2.2: Leave-one-out meta-analysis for EBF indicator 5
Fig s2.3: Leave-one-out meta-analysis for CF indicator 6
3. Meta funnel plot 7
Fig s3.1: Funnel plot for EIBF indicator 7
Fig s3.2: Funnel plot for EBF indicator 8
Fig s3.3: Funnel plot for CF indicator 9
4. Search strategy 10
5. Funding sources of the included studies 11
6. PRISMA checklist 13
7. Records excluded during full text review 16
8. Name of data extractor and date of data extraction 20
9. Dataset for meta-analysis 22
Table s4.1: Early initiation of breastfeeding 22
Table s4.2: Exclusive breastfeeding 22
Table s4.3: Complementary feeding 23
10. Strategy for handling missing data 24

11. List of all articles found in databases searches for title and abstract and full text screening 24

1. Quality assessment of the included articles

Table s1.1: Quality of the included cross-sectional studies

Author, year	Selection				Comparability	Outcome		Overall quality
	Representativeness of the sample	Sample size	Non-respondent	Exposure assignment		Outcome assessment	Statistical accuracy	
Ahmed, 2022	a*	a*	a*	a**	a**	a**	a*	10
Ahmmed, 2022	a*	a*	a*	a**	a**	a**	a*	10
Akter, 2010 (a)	a*	a*	a*	a**	b	a**	a*	8
Akter, 2010 (b)	a*	a*	a*	a**	b	a**	a*	8
Ali, 2019	a*	a*	a*	a**	a**	a**	a*	10
Ayesha, 2021	a*	a*	a*	a**	a**	a**	a*	10
Basnet, 2020	a*	a*	a*	a**	a**	a**	a*	10
Blackstone, 2018 (a)	a*	a*	a*	a**	a**	a**	a*	10
Blackstone, 2018 (b)	a*	a*	a*	a**	a**	a**	a*	10
Hasan, 2021	b*	a*	a*	b*	a**	a**	a*	9
Hasan, 2020	b*	a*	a*	c	a**	a**	a*	8
Hossain, 2018	a*	a*	a*	a**	a**	a**	a*	10
Jain, 1981	a*	a*	a*	a**	a**	a**	a*	10
Kabir 2012	a*	a*	a*	a**	a**	a**	a*	10

Author, year	Selection				Comparability	Outcome		Overall quality
	Representativeness of the sample	Sample size	Non-respondent	Exposure assignment		Outcome assessment	Statistical accuracy	
Khan, 2019	a*	a*	a*	a**	b	a**	a*	8
Kundu, 2022	a*	a*	a*	c	a**	a**	a*	8
Mihrshahi, 2010	a*	a*	a*	a**	a**	a**	a*	10
Nguyen, 2013	a*	a*	a*	a**	a**	a**	a*	10
Rahman, 2020	a*	a*	a*	a**	a**	a**	a*	10
Raihana, 2021	a*	a*	a*	a**	b	a**	a*	08
Rana, 2020	b*	a*	a*	a**	a**	a**	a*	10
Rasheed, 2009	a*	b	b	a**	a**	a**	a*	8
Sheikh, 2019	a*	a*	a*	a**	a**	a**	a*	10

Table s1.2: Quality of the included cohort or longitudinal study

Author, year	Selection				Comparability	Outcome			Overall quality
	Representativeness of exposed cohort (ISA)	Selection of non-exposed cohort (GSA)	Ascertainment of exposure	Absence of outcome interest at the beginning		Assessment of outcome	Adequate follow-up	Adequacy of follow-up	
Haider, 2019	a*	a*	b*	a*	N/A	a*	a*	a*	7

∝The questions were contextualized from Newcastle-Ottawa Scale

2. Leave-one-out meta-analysis

Fig s2.1: Leave-one-out meta-analysis for EIBF indicator

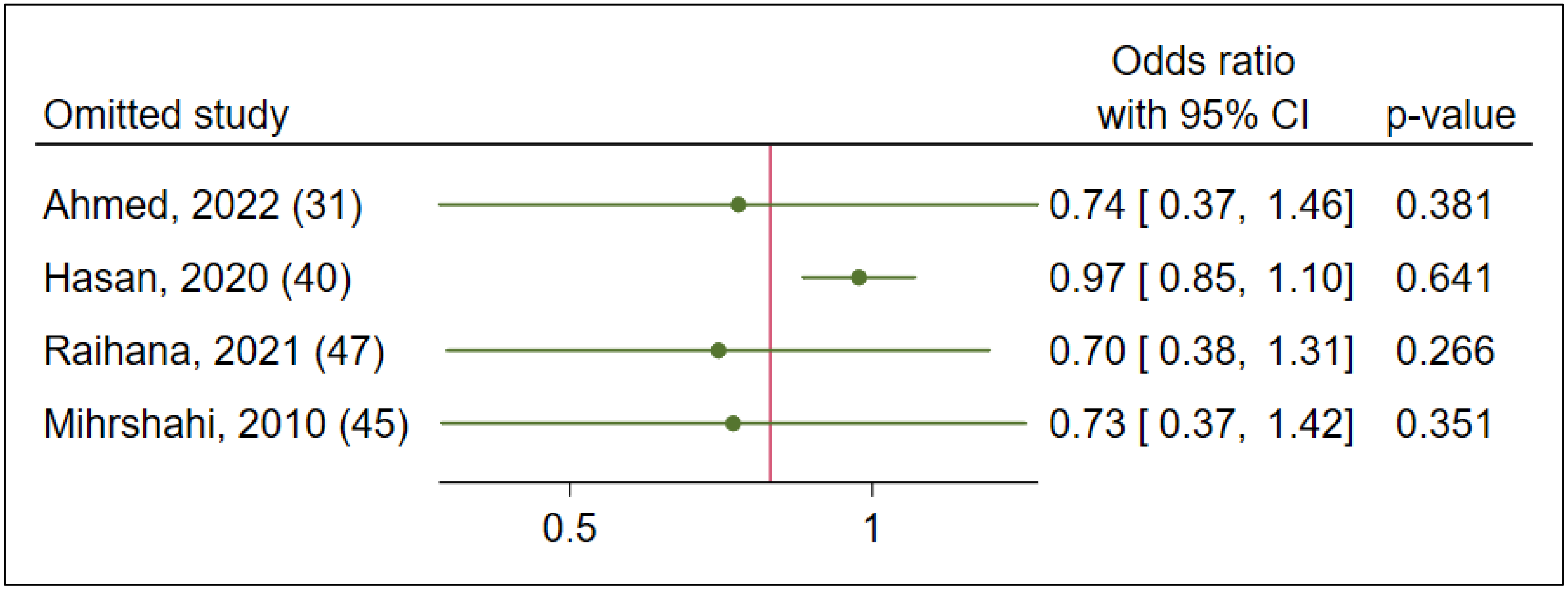

Figure s2.1: Effect on the pooled association between early initiation of breastfeeding and maternal occupation in Bangladesh due to omission of any of the studies included in the meta-analysis.

Fig s2.2: Leave-one-out meta-analysis for EBF indicator

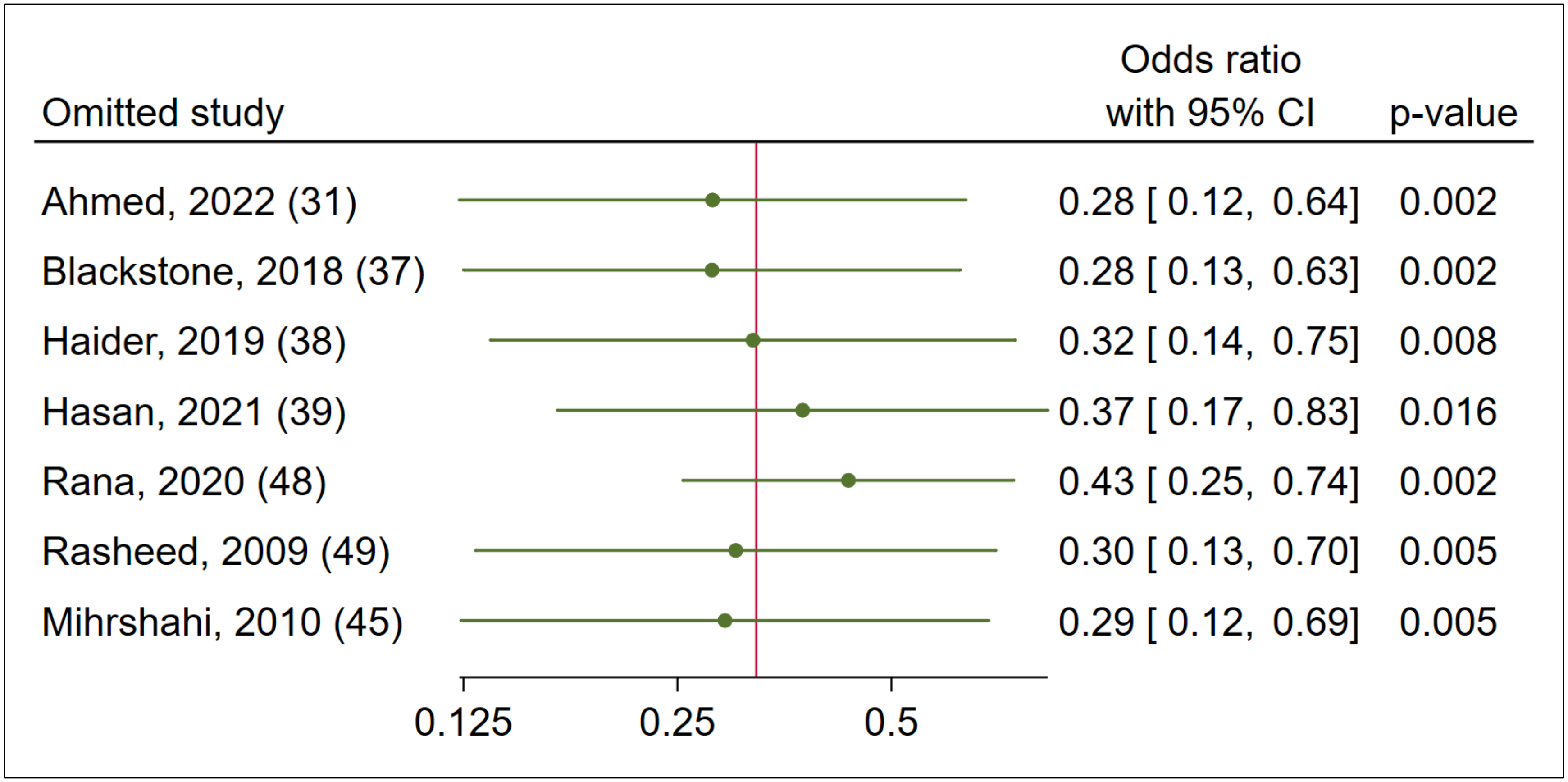

Figure s2.2: Effect on the pooled association between exclusive breastfeeding and maternal occupation in Bangladesh due to omission of any of the studies included in the meta-analysis.

Fig s2.3: Leave-one-out meta-analysis for CF indicator

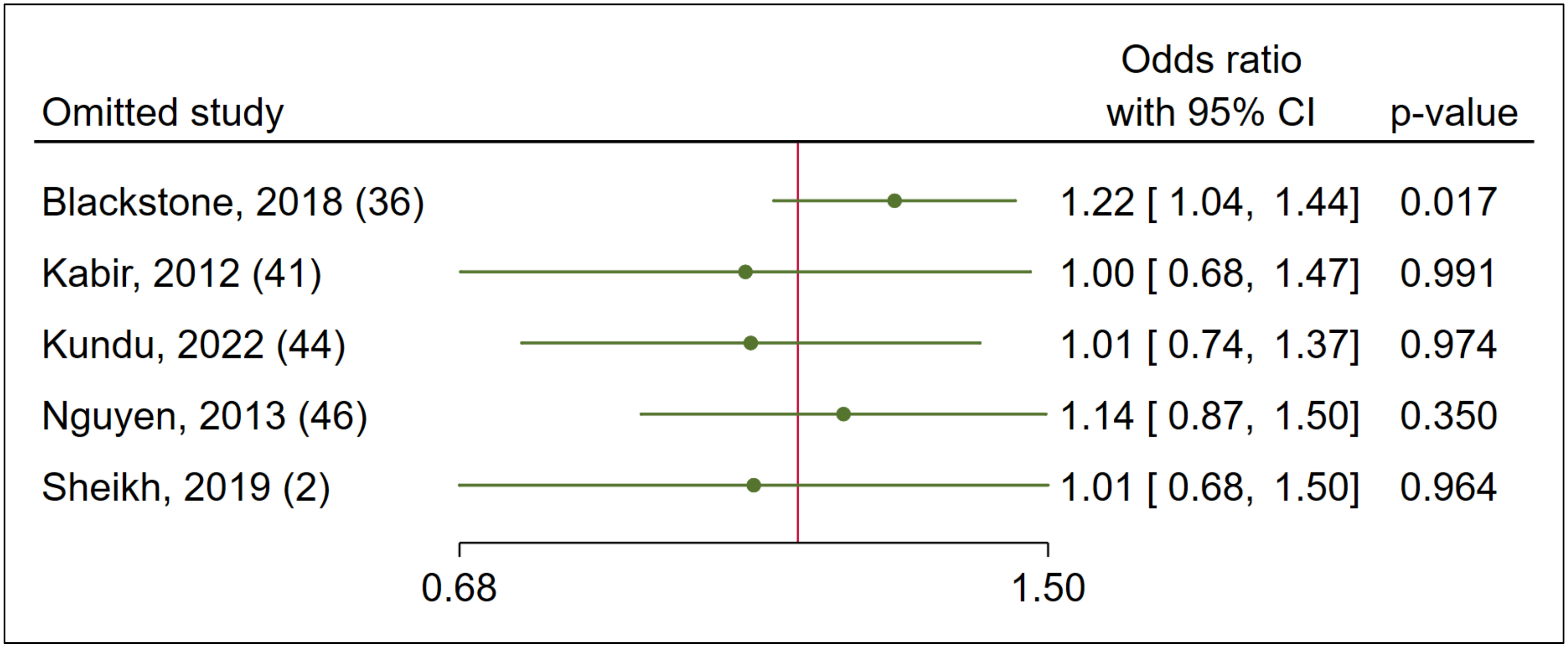

Figure s2.3 Effect on the pooled association between complementary feeding and maternal occupation in Bangladesh due to omission of any of the studies included in the meta-analysis.

3. Meta funnel plot

Fig s3.1: Funnel plot for EIBF indicator

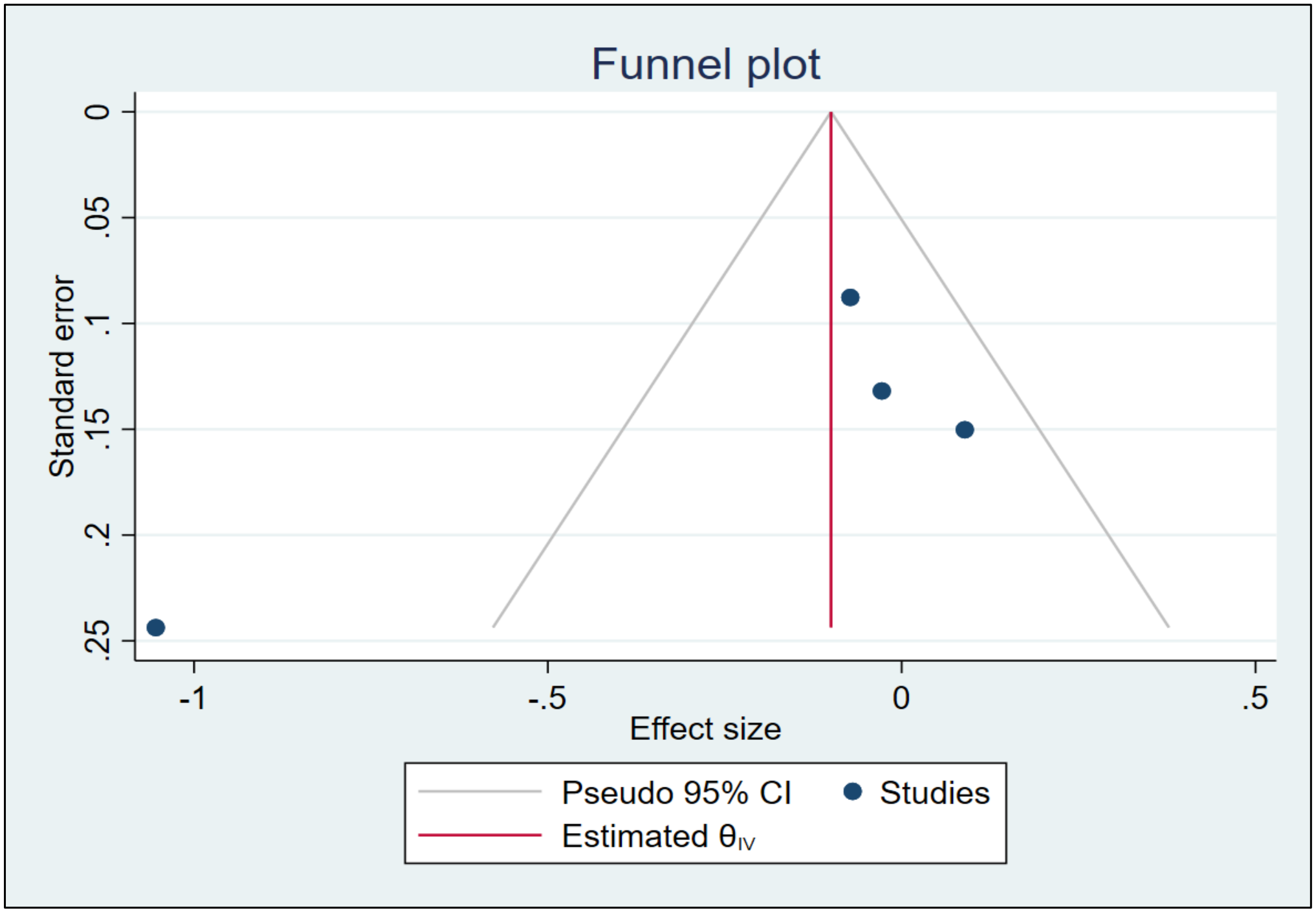

Figure s3.1 Publication bias among the studies included in the meta-analysis of the association between early initiation of breastfeeding and maternal occupation in Bangladesh.

Fig s3.2: Funnel plot for EBF indicator

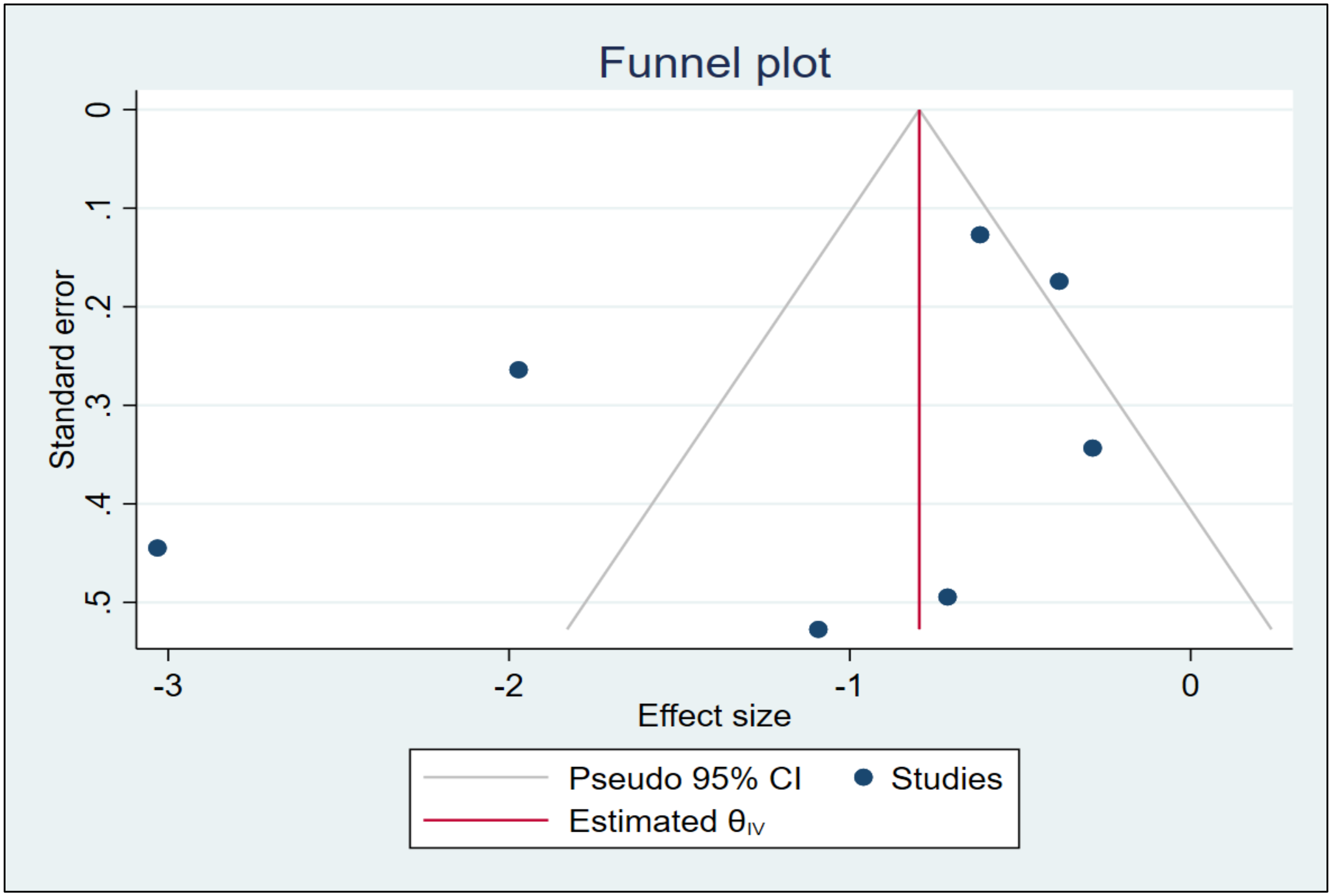

Figure s3.2 Publication bias among the studies included in the meta-analysis of the association between exclusive breastfeeding and maternal occupation in Bangladesh.

Fig s3.3: Funnel plot for CF indicator

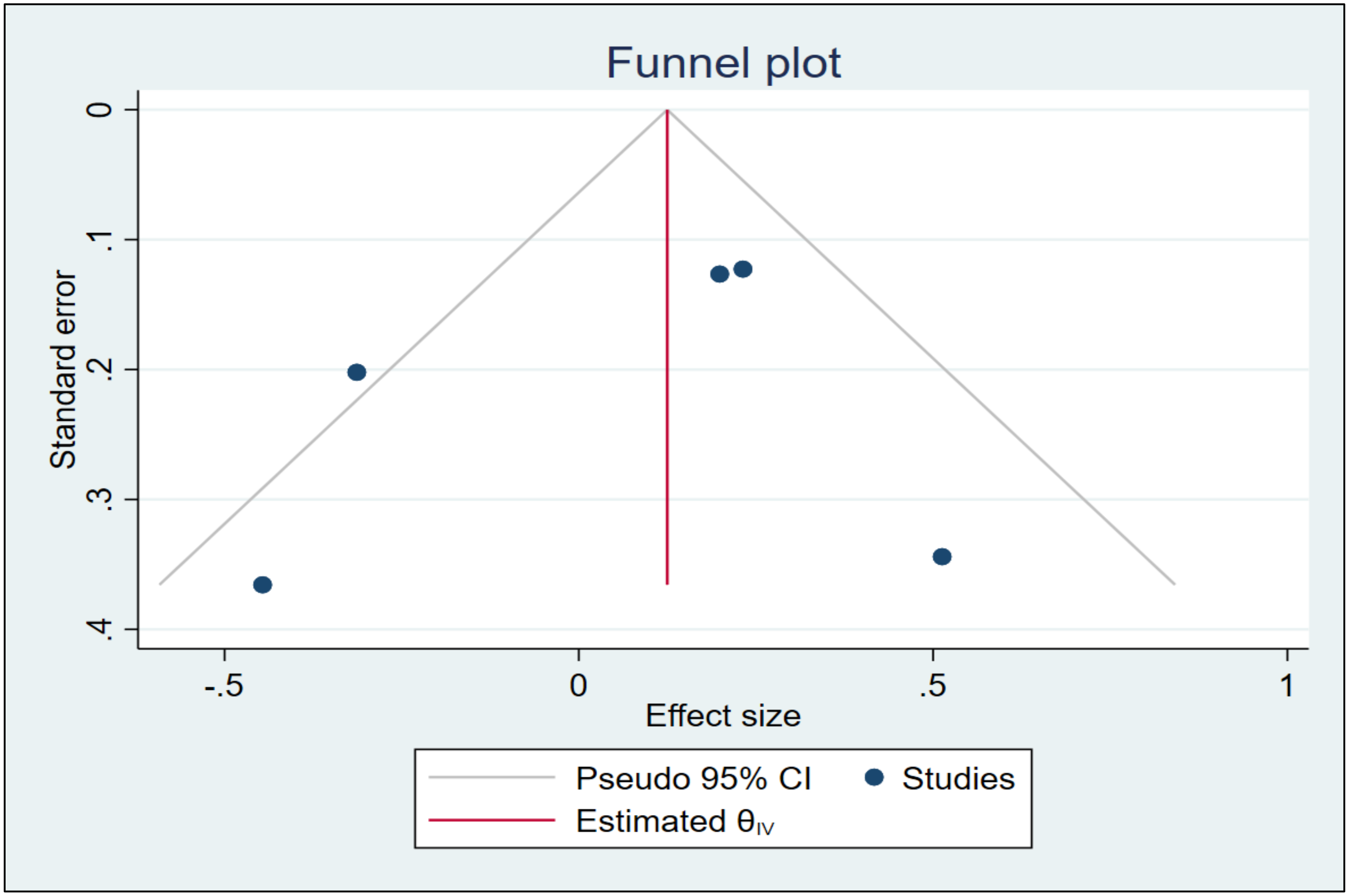

Figure s3.3 Publication bias among the studies included in the meta-analysis of the association between complementary feeding and maternal occupation in Bangladesh.

4. Search strategy

Set no.	Keywords
#12	#7 AND #10 AND #11
#11	Bangladesh[MeSH] OR Bangladesh OR Bangladeshi
#10	#9 AND #8
#9	mother* OR maternal OR lactati* OR wom?n
#8	occupation OR employ* OR job OR income OR work* OR business OR entrepreneur* OR profession*
#7	#1 OR #2 OR #3 OR #4 OR #5 OR #6
#6	"Minimum meal frequency" OR "Minimum acceptable diet" OR "Dietary diversity"
#5	complementary feeding[MeSH Terms] OR complementary feedings[MeSH Terms] OR "Complementary feeding" OR complement* OR supplement* OR "food complement" OR "Appropriate complementary feeding"
#4	"infant feeding" OR "neonatal feeding" OR "child feeding" OR "baby feeding" OR "Infant and young child feeding" OR infant food[MeSH Terms] OR infant foods[MeSH Terms] OR IYCF
#3	weaning OR "weaning practice" OR weaning[MeSH Terms] OR "weaning food" OR "weaning foods"
#2	"Prelacteal feeding" OR "Bottle feeding" OR bottle feeding[MeSH Terms] OR bottle feedings[MeSH Terms] OR "formula feeding" OR "breastmilk substitute" OR infant formula[MeSH Terms] OR infant formulas[MeSH Terms]
#1	Breastfeeding[MeSH] OR Breastfeeding OR "Early Breastfeeding" OR Colostrum[MeSH] OR Colostrum OR "Colostrum feeding" OR "Early initiation of breastfeeding" OR "Exclusive breastfeeding" OR "Timely breastfeeding" OR (breastmilk expression[MeSH Terms]) OR (breastmilk expressions[MeSH Terms]) OR "Breastfeeding continuation" OR "Continuation of breastfeeding" OR "Age appropriate breastfeeding" OR feeding breast OR lactation OR lactation[MeSH Terms] OR breastfeed OR “breast fed”

5. Funding sources of the included studies

Author, year	Geographical area covered	Data source	Funding sources
Ahmed, 2022	National	BDHS 2004-2018	No fund received
Ahmmed, 2022	National	BDHS 2011-2018	Acquired fund but not reported sources
Akter, 2010	National	BDHS 2004	Not reported
Akter, 2010	National	BDHS 2004	Not reported
Ali, 2019	20 sub-districts	Household survey 2014-2017	Research protocol funded by World Vision Bangladesh
Ayesha, 2021	National	BDHS 2014	No fund received
Basnet, 2020	20 sub-districts	Alive and Thrive baseline data 2010	Bill & Melinda Gates Foundation
Blackstone, 2018	National	BDHS 2011 and 2014	Bill & Melinda Gates Foundation
Blackstone, 2018	National	BDHS 2007, 2011 and 2014	Bill and Melinda Gates Foundation: Alive and Thrive.
Haider, 2019	Chattogram	Survey 2015-2017	BSRM, IDLC Finance Ltd., WABA, and Training & Assistance for Health & Nutrition Foundation (TAHN)
Hasan, 2021	Dhaka district	Survey 2019	No fund received
Hasan, 2020	Dhaka district	Survey 2019	Not reported
Hossain, 2018	National	BDHS 2014	No fund received
Kabir, 2012	National	BDHS 2007	Public Sector Linkage Programme of the Australian Agency for International Development (AusAID)
Jain, 1981	National	World Fertility Surveys 1976	Not reported
Khan, 2019	National	BDHS 2011, 2014	Not reported
Kundu, 2022	6 districts	Survey 2021	No fund received

**Author, year**	**Geographical area covered**	**Data source**	**Funding sources**
Mihrshahi, 2010	National	BDHS 2004	Public Sector Linkage Programme of the Australian Agency for International Development (AusAID)
Nguyen, 2013	20 sub-districts	Alive and Thrive baseline data 2010	Bill and Melinda Gates Foundation, through Alive & Thrive, managed by FHI360.
Rahman, 2020	National	BDHS 2011 and 2014	No fund received
Raihana, 2021	National	BDHS 2014	Endeavour Postgraduate Scholarship from the Australian Government, Department of Education for PhD work.
Rana, 2020	Rural Rajshahi	Survey 2015	No fund received
Rasheed, 2009	Matlab	Survey 2002-2004	United Nations Children's Fund, Swedish International Development Cooperation Agency (SIDA), UK Medical Research Council, Swedish Research Council, Department of International development (DFID), International Centre for Diarrhoeal Disease Research, Bangladesh (ICDDR,B), Global Health Research Fund-Japan, Child Health and Nutrition Research Initiative, Uppsala University, and the United States Agency for International Development.
Sheikh, 2019	National	BDHS 2014	No fund received

6. PRISMA checklist

Section and Topic	Item #	Checklist item	Location where item is reported
**TITLE**			
Title	1	Identify the report as a systematic review.	Title
**ABSTRACT**			
Abstract	2	See the PRISMA 2020 for Abstracts checklist.	Abstract
**INTRODUCTION**			
Rationale	3	Describe the rationale for the review in the context of existing knowledge.	Paragraph 3
Objectives	4	Provide an explicit statement of the objective(s) or question(s) the review addresses.	Paragraph 3
**METHODS**			
Eligibility criteria	5	Specify the inclusion and exclusion criteria for the review and how studies were grouped for the syntheses.	Inclusion and exclusion criteria
Information sources	6	Specify all databases, registers, websites, organisations, reference lists and other sources searched or consulted to identify studies. Specify the date when each source was last searched or consulted.	Data source and search strategy
Search strategy	7	Present the full search strategies for all databases, registers and websites, including any filters and limits used.	Data source and search strategy
Selection process	8	Specify the methods used to decide whether a study met the inclusion criteria of the review, including how many reviewers screened each record and each report retrieved, whether they worked independently, and if applicable, details of automation tools used in the process.	Selection process
Data collection process	9	Specify the methods used to collect data from reports, including how many reviewers collected data from each report, whether they worked independently, any processes for obtaining or confirming data from study investigators, and if applicable, details of automation tools used in the process.	Data extraction and analysis
Data items	10a	List and define all outcomes for which data were sought. Specify whether all results that were compatible with each outcome domain in each study were sought (e.g., for all measures, time points, analyses), and if not, the methods used to decide which results to collect.	Data extraction and Data synthesis and Meta-analysis
	10b	List and define all other variables for which data were sought (e.g., participant and intervention characteristics, funding sources). Describe any assumptions made about any missing or unclear information.	Data extraction and Data synthesis and Meta-analysis
Study risk of bias assessment	11	Specify the methods used to assess risk of bias in the included studies, including details of the tool(s) used, how many reviewers assessed each study and whether they worked independently, and if applicable, details of automation tools used in the process.	Assessment of quality and risk of bias
Effect measures	12	Specify for each outcome the effect measure(s) (e.g., risk ratio, mean difference) used in the synthesis or presentation of results.	Data synthesis and Meta-analysis
Synthesis methods	13a	Describe the processes used to decide which studies were eligible for each synthesis (e.g., tabulating the study intervention characteristics and comparing against the planned groups for each synthesis (item #5)).	Data synthesis and Meta-analysis

Section and Topic	Item #	Checklist item	Location where item is reported
	13b	Describe any methods required to prepare the data for presentation or synthesis, such as handling of missing summary statistics, or data conversions.	Data synthesis and Meta-analysis
	13c	Describe any methods used to tabulate or visually display results of individual studies and syntheses.	Data synthesis and Meta-analysis
	13d	Describe any methods used to synthesize results and provide a rationale for the choice(s). If meta-analysis was performed, describe the model(s), method(s) to identify the presence and extent of statistical heterogeneity, and software package(s) used.	Data synthesis and meta-analysis
	13e	Describe any methods used to explore possible causes of heterogeneity among study results (e.g., subgroup analysis, meta-regression).	Data synthesis and meta-analysis
	13f	Describe any sensitivity analyses conducted to assess robustness of the synthesized results.	Data synthesis and meta-analysis
Reporting bias assessment	14	Describe any methods used to assess risk of bias due to missing results in a synthesis (arising from reporting biases).	Assessment of quality and risk of bias
Certainty assessment	15	Describe any methods used to assess certainty (or confidence) in the body of evidence for an outcome.	Not carried out
**RESULTS**			
Study selection	16a	Describe the results of the search and selection process, from the number of records identified in the search to the number of studies included in the review, ideally using a flow diagram.	Results, Figure 1
	16b	Cite studies that might appear to meet the inclusion criteria, but which were excluded, and explain why they were excluded.	Figure 1
Study characteristics	17	Cite each included study and present its characteristics.	Table 1-3
Risk of bias in studies	18	Present assessments of risk of bias for each included study.	Table 1 and supplementary file
Results of individual studies	19	For all outcomes, present, for each study: (a) summary statistics for each group (where appropriate) and (b) an effect estimate and its precision (e.g. confidence/credible interval), ideally using structured tables or plots.	Table 2-3
Results of syntheses	20a	For each synthesis, briefly summarise the characteristics and risk of bias among contributing studies.	Table 2 and supplementary file
	20b	Present results of all statistical syntheses conducted. If meta-analysis was done, present for each the summary estimate and its precision (e.g., confidence/credible interval) and measures of statistical heterogeneity. If comparing groups, describe the direction of the effect.	Figure 2-4
	20c	Present results of all investigations of possible causes of heterogeneity among study results.	Meta-analysis, Figure 2-4
	20d	Present results of all sensitivity analyses conducted to assess the robustness of the synthesized results.	Leave-one-out meta-analysis, supplementary file
Reporting	21	Present assessments of risk of bias due to missing results (arising from reporting biases) for each synthesis assessed.	Results: Quality of

Section and Topic	Item #	Checklist item	Location where item is reported
biases			included articles
Certainty of evidence	22	Present assessments of certainty (or confidence) in the body of evidence for each outcome assessed.	Not carried out
**DISCUSSION**			
Discussion	23a	Provide a general interpretation of the results in the context of other evidence.	Paragraph 1-4
	23b	Discuss any limitations of the evidence included in the review.	Strengths, limitations, and future scopes
	23c	Discuss any limitations of the review processes used.	Strengths, limitations, and future scopes
	23d	Discuss implications of the results for practice, policy, and future research.	Policy implications and recommendations
**OTHER INFORMATION**			
Registration and protocol	24a	Provide registration information for the review, including register name and registration number, or state that the review was not registered.	Abstract and Methods (conceptualization)
	24b	Indicate where the review protocol can be accessed, or state that a protocol was not prepared.	Abstract and Methods (conceptualization)
	24c	Describe and explain any amendments to information provided at registration or in the protocol.	Abstract and Methods (conceptualization)
Support	25	Describe sources of financial or non-financial support for the review, and the role of the funders or sponsors in the review.	Funding
Competing interests	26	Declare any competing interests of review authors.	Competing interest
Availability of data, code, and other materials	27	Report which of the following are publicly available and where they can be found template data collection forms; data extracted from included studies; data used for all analyses; analytic code; any other materials used in the review.	Supplementary files

7. Records excluded during full text review

Sl no.	Excluded articles	Exclusion reason
1.	Islam MA, Mamun AS, Hossain MM, Bharati P, Saw A, Lestrel PE, Hossain MG. Prevalence and factors associated with early initiation of breastfeeding among Bangladeshi mothers: a nationwide cross-sectional study. PloS one. 2019 Apr 25;14(4):e0215733.	Not relevant exposure
2.	Chowdhury T, Roy P, Huq O, Shaon KA. Infant and Young Child Feeding Practices among the selected Urban Working Lactating Mother, Bangladesh.	Not relevant analysis
3.	Zongrone AA, Menon P, Pelto GH, Habicht JP, Rasmussen KM, Constas MA, Vermeylen F, Khaled A, Saha KK, Stoltzfus RJ. The pathways from a behavior change communication intervention to infant and young child feeding in Bangladesh are mediated and potentiated by maternal self-efficacy. The Journal of Nutrition. 2018 Feb 1;148(2):259-66.	Not relevant analysis
4.	Rahman M, Yunus FM, Shah R, Jhohura FT, Mistry SK, Quayyum T, Aktar B, Afsana K. A controlled before-and-after perspective on the improving maternal, neonatal, and child survival program in rural Bangladesh: an impact analysis. PLoS One. 2016 Sep 1;11(9):e0161647.	Not relevant analysis
5.	Nahar B, Ahmed T, Brown KH, Hossain MI. Risk factors associated with severe underweight among young children reporting to a diarrhoea treatment facility in Bangladesh. Journal of health, population, and nutrition. 2010 Oct;28(5):476.	Not relevant outcomes
6.	Pagel C, Prost A, Hossen M, Azad K, Kuddus A, Roy SS, Nair N, Tripathy P, Saville N, Sen A, Sikorski C. Is essential newborn care provided by institutions and after home births? Analysis of prospective data from community trials in rural South Asia. BMC pregnancy and childbirth. 2014 Dec;14:1-9.	Not relevant exposure
7.	Giashuddin MS, Kabir M, Rahman A, Hannan MA. Exclusive breastfeeding and nutritional status in Bangladesh. The Indian Journal of Pediatrics. 2003 Jun;70:471-5.	Not relevant exposure
8.	Iqbal A. Knowledge and practices regarding infant and young child feeding among mothers working in readymade garments sector in bangladesh: A cross-sectional survey. Current Research in Nutrition and Food Science. 2021 Apr 1;9(1):190.	Not relevant analysis
9.	Senarath U, Agho KE, Akram DE, Godakandage SS, Hazir T, Jayawickrama H, Joshi N, Kabir I, Khanam M, Patel A, Pusdekar Y. Comparisons of complementary feeding indicators and associated factors in children aged 6–23 months across five South Asian countries. Maternal & child nutrition. 2012 Jan;8:89-106.	Not relevant analysis
10.	Islam MJ, Broidy L, Baird K, Rahman M, Zobair KM. Early exclusive breastfeeding cessation and postpartum depression: Assessing the mediating and moderating role of maternal stress and social support. PloS one. 2021 May 17;16(5):e0251419.	Not relevant outcomes
11.	Na M, Aguayo VM, Arimond M, Narayan A, Stewart CP. Stagnating trends in complementary feeding practices in Bangladesh: An analysis of national surveys from 2004-2014. Maternal & Child Nutrition. 2018 Nov;14:e12624.	Not relevant exposure

Sl no.	Excluded articles	Exclusion reason
12.	Ahmed S, Parveen SD, Islam A. Infant feeding practices in rural Bangladesh: policy implications. Journal of tropical pediatrics. 1999 Feb 1;45(1):37-41.	Not relevant exposure
13.	Mistry SK, Hossain MB, Irfan NM, Saha M, Saberin S, Shamim AA, Arora A. Trends in Complementary Feeding Indicators and Intake from Specific Food Groups among Children Aged 6–23 Months in Bangladesh. International Journal of Environmental Research and Public Health. 2022 Jan 4;19(1):550.	Not relevant analysis
14.	Islam M, Afroja S, Biswas A, Khan MS, Khandker S. Influence of socio-demographic factors on the breastfeeding period of women in Bangladesh: a polytomous logistic regression model. Family Medicine & Primary Care Review. 2019(3):223-9.	Not relevant exposure
15.	Raihana S, Alam A, Chad N, Huda TM, Dibley MJ. Delayed initiation of breastfeeding and role of mode and place of childbirth: evidence from health surveys in 58 low-and middle-income countries (2012–2017). International Journal of Environmental Research and Public Health. 2021 Jun 2;18(11):5976.	Not relevant exposure
16.	Komatsu H, Malapit HJ, Theis S. How does women's time in reproductive work and agriculture affect maternal and child nutrition? Evidence from Bangladesh, Cambodia, Ghana, Mozambique, and Nepal.	Not relevant exposure
17.	Guldan GS, Zeitlin MF, Beiser AS, Super CM, Gershoff SN, Datta S. Maternal education and child feeding practices in rural Bangladesh. Social science & medicine. 1993 Apr 1;36(7):925-35.	Not relevant exposure
18.	Khatun H, Comins CA, Shah R, Munirul Islam M, Choudhury N, Ahmed T. Uncovering the barriers to exclusive breastfeeding for mothers living in Dhaka's slums: a mixed method study. International breastfeeding journal. 2018 Dec;13:1-1.	Not relevant exposure
19.	Senarath U, Dibley MJ. Complementary feeding practices in South Asia: analyses of recent national survey data by the South Asia Infant Feeding Research Network. Maternal & child nutrition. 2012 Jan;8:5-10.	Not relevant analysis
20.	Nessa F, Rahman S. Breast feeding patterns of working women in the Dhaka metropolitan area. Bangladesh Med Res Counc Bull. 1988.	Studies not retrieved
21.	Sakib MS, Ripon Rouf AS, Tanny TF. Determinants of early initiation of breastfeeding practices of newborns in bangladesh: evidence from bangladesh demographic and health survey. Nutrition and Metabolic Insights. 2021 Oct;14:11786388211054677.	Not relevant exposure
22.	Karim F, Khan AN, Tasnim F, Chowdhury MA, Billah SM, Karim T, Arifeen SE, Garnett SP. Prevalence and determinants of initiation of breastfeeding within one hour of birth: An analysis of the Bangladesh Demographic and Health Survey, 2014. PloS one. 2019 Jul 25;14(7):e0220224.	Not relevant exposure
23.	Dintyala SS. A STUDY OF THE RELATIONSHIP BETWEEN MATERNAL ANTENATAL VISITATION AND ADHERENCE TO EXCLUSIVE BREASTFEEDING AT 6th MONTH.	Not relevant exposure
24.	Raihana S, Dibley MJ, Rahman MM, Tahsina T, Siddique MA, Rahman QS, Islam S, Alam A, Kelly PJ, Arifeen SE, Huda TM. Early initiation of breastfeeding and severe illness in the early newborn period: An observational study in rural Bangladesh. PLoS medicine. 2019 Aug 30;16(8):e1002904.	Not relevant exposure

Sl no.	Excluded articles	Exclusion reason
25.	Talukder S, Farhana D, Vitta B, Greiner T. In a rural area of Bangladesh, traditional birth attendant training improved early infant feeding practices: a pragmatic cluster randomized trial. Maternal & child nutrition. 2017 Jan;13(1):e12237.	Not relevant analysis
26.	Elaine AY, Thomas JS, Owais A, Tirmizi N, Faruque AS, Das SK, Rahman S, Schwartz B, Stein AD. Maternal prenatal attitudes and postnatal breast-feeding behaviours in rural Bangladesh. Public health nutrition. 2015 Mar;18(4):679-85.	Not relevant exposure
27.	Sen KK, Mallick TS, Bari W. Gender inequality in early initiation of breastfeeding in Bangladesh: a trend analysis. International breastfeeding journal. 2020 Dec;15:1-1.	Not relevant exposure
28.	Menon P, Nguyen PH, Saha KK, Khaled A, Sanghvi T, Baker J, Afsana K, Haque R, Frongillo EA, Ruel MT, Rawat R. Combining intensive counseling by frontline workers with a nationwide mass media campaign has large differential impacts on complementary feeding practices but not on child growth: results of a cluster-randomized program evaluation in Bangladesh. The Journal of nutrition. 2016 Oct 1;146(10):2075-84.	Not relevant analysis
29.	Al Mamun MA, Saha S, Li J, Binta A Ghani R, Al Hasan SM, Begum A. Child feeding practices of childbearing mothers and their household food insecurity in a coastal region of Bangladesh. INQUIRY: The Journal of Health Care Organization, Provision, and Financing. 2022 Apr 15;59:00469580221096277.	Not relevant exposure
30.	Komatsu H, Malapit HJ, Theis S. Does women's time in domestic work and agriculture affect women's and children's dietary diversity? Evidence from Bangladesh, Nepal, Cambodia, Ghana, and Mozambique. Food policy. 2018 Aug 1;79:256-70.	Not relevant analysis
31.	Oddo VM, Ickes SB. Maternal employment in low-and middle-income countries is associated with improved infant and young child feeding. The American journal of clinical nutrition. 2018 Mar 1;107(3):335-44.	Wrong study design
32.	Rahman A, Nomani D, Taneepanichskul S. Trends and determinants of EBF among adolescent children born to adolescent mothers in rural Bangladesh. International Journal of Environmental Research and Public Health. 2020 Nov;17(24):9315.	Not relevant exposure
33.	Rahman M, Haque SE, Zahan S, Islam O. Noninstitutional births and newborn care practices among adolescent mothers in Bangladesh. Journal of Obstetric, Gynecologic & Neonatal Nursing. 2011 May 1;40(3):262-73.	Not relevant exposure
34.	Giashuddin MS, Kabir M. Duration of breast-feeding in Bangladesh. Indian Journal of Medical Research. 2004 Jun 1;119:267-72.	Not relevant exposure
35.	Das DK, Talukder MQ, Sella GE. Infant feeding practices in rural Bangladesh. The Indian Journal of Pediatrics. 1992 Sep;59:573-7.	Not relevant analysis
36.	Tariqujjaman M, Hasan MM, Mahfuz M, Hossain M, Ahmed T. Association between mother's education and infant and young child feeding practices in South Asia. Nutrients. 2022 Apr 5;14(7):1514.	Not relevant exposure
37.	Haider R, Begum S. Working women, maternity entitlements, and breastfeeding: a report from Bangladesh. Journal of Human Lactation. 1995 Dec;11(4):273-7.	Not relevant analysis
38.	Ghosh R, Mascie-Taylor CN, Rosetta L. Longitudinal study of the frequency and duration of breastfeeding in rural Bangladeshi women. American Journal of Human Biology: The Official Journal of the Human Biology Association. 2006 Sep;18(5):630-8.	Not relevant analysis

Sl no.	Excluded articles	Exclusion reason
39.	Khan JR, Awan N, Sheikh MT. A multilevel and spatial analysis of the infant and young child feeding practices and associated factors among the under-2 aged children in Bangladesh. Child Care in Practice. 2022 Apr 3;28(2):178-95.	Not relevant analysis
40.	Campbell RK, Hurley KM, Shamim AA, Shaikh S, Chowdhury ZT, Mehra S, De Pee S, Ahmed T, West Jr KP, Christian P. Effect of complementary food supplementation on breastfeeding and home diet in rural Bangladeshi children. The American journal of clinical nutrition. 2016 Nov 1;104(5):1450-8.	Not relevant exposure
41.	Howlader H, Rahman A, Hasan M. Breastfeeding knowledge, attitudes and practice among rural women in Bangladesh: insights from Tungipara village. Family Medicine & Primary Care Review. 2020 Oct 1;22(4).	Not relevant analysis

8. Name of data extractor and date of data extraction

Sl no.	Article title	Data extractor	Date of data extraction
1	Ahmed MS, Whitfield KC, Yunus FM. Trends and predictors of early initiation, exclusive and continued breast-feeding in Bangladesh (2004-2018): A multilevel analysis of demographic and health survey data. British Journal of Nutrition. 2022 Nov 14;128(9):1857–67.	MAR, TA, PS	February 4, 2023
2	Ahmmed F, Hossain MdJ, Sutopa TS, Al-Mamun Md, Alam M, Islam MdR, et al. The trend in exclusive breastfeeding practice and its association with maternal employment in Bangladesh: A multilevel analysis. Frontiers in Public Health . 2022; 10(988016)	SSA, TA, MAR	February 4, 2023
3	Akter S, Rahman MdM. The Determinants of Early Cessation of Breastfeeding in Bangladesh. World Health & Population . 2010;11(4).	SSA, TA. MAR	February 5, 2023
4	Akter S, Rahman MM. Duration of Breastfeeding and Its Correlates in Bangladesh. Journal of Health, Population and Nutrition . 2010;28(6).	SSA, TA, MAR	February 6, 2023
5	Ali NB, Tahsina T, Emdadul Hoque DM, Hasan MM, Iqbal A, Huda TM, et al. Association of food security and other socioeconomic factors with dietary diversity and nutritional statuses of children aged 6-59 months in rural Bangladesh. PLoS One. 2019 Aug 1;14(8).	SSA, TA, MAR	February 7, 2023
6	Ayesha U, Mamun ASMA, Sayem MA, Hossain MG. Factors associated with duration of breastfeeding in Bangladesh: evidence from Bangladesh demographic and health survey 2014. BMC Public Health. 2021 Dec 1;21(1).	SSA, TA, MAR	February 8, 2023
7	Basnet S, Frongillo EA, Nguyen PH, Moore S, Arabi M. Associations of maternal resources with care behaviours differ by resource and behaviour. Matern Child Nutr. 2020 Jul 1;16(3).	SSA, TA, MAR	February 8, 2023
8	Blackstone S, Sanghvi T. A comparison of minimum dietary diversity in Bangladesh in 2011 and 2014. Matern Child Nutr. 2018 Oct 1;14(4).	PS, TA, MAR	February 8, 2023
9	Blackstone SR, Sanghvi T. Predictors of exclusive breastfeeding across three time points in Bangladesh: An examination of the 2007, 2011 and 2014 Demographic and Health Survey. Int Health. 2018 May 1;10(3):149–56.	SSA, TA, MAR	February 9, 2023
10	Haider R, Thorley V. Supporting Exclusive Breastfeeding Among Factory Workers and Their Unemployed Neighbors: Peer Counseling in Bangladesh. Journal of Human Lactation. 2019 Aug 1;36(3):414–25.	PS, MAR, TA	February 11, 2023
11	Hasan M, Hassan MN, Khan MSI, Tareq MA, Afroj MS. Prevalence, knowledge, attitudes and factors associated with exclusive breastfeeding among mothers in Dhaka, Bangladesh: A cross-sectional study. Popul Med. 2021 Sep 1;3:1–7.	PS, TA, MAR	February 12, 2023
12	Hasan M, Hassan MdN, Khan MSI, Al Banna MdH. Prevalence and Determinants of Early Initiation of Breastfeeding Among Mothers in Dhaka City, Bangladesh: a Cross-sectional Study. SN Compr Clin Med. 2020 Dec;2(12):2792–8.	SSA, TA, MAR	February 12, 2023
13	Hossain M, Islam A, Kamarul T, Hossain G. Exclusive breastfeeding practice during first six months of an infant's life in Bangladesh: A country based cross-sectional study. BMC Pediatr. 2018 Mar 2;18(1).	PS, TA, MAR	February 12, 2023

Sl no.	Article title	Data extractor	Date of data extraction
**14**	Kabir I, Khanam M, Agho KE, Mihrshahi S, Dibley MJ, Roy SK. Determinants of inappropriate complementary feeding practices in infant and young children in Bangladesh: Secondary data analysis of Demographic Health Survey 2007. Matern Child Nutr. 2012 Jan;8(SUPPL. 1):11–27.	**PS, MAR, TA**	**February 12, 2023**
**15**	Jain AK, Bongaarts J. Breastfeeding: Patterns, Correlates, and Fertility Effects. Family Planning [Internet]. 1981;12(3):79–99. Available from: http://www.jstor.orgURL:http://www.jstor.org/stable/1966370Accessed:07-12-201518:52UTC	**PS, MAR, TA**	**February 13, 2023**
**16**	Khan JR, Sheikh MT, Muurlink O. Breastfeeding termination and its determinants in Bangladesh: current status data modelling. Early Child Dev Care. 2019;190(16):2594–604.	**TA, SSA, MAR**	**February 13, 2023**
**17**	Kundu S, Sayeed A, Gedef Azene A, Rezyona H, Al Banna MH, Shafiqul M, et al. Exploring the factors associated with dietary diversity of children aged 6-59 months in some rural and slum areas of Bangladesh amid the COVID-19 pandemic: A mixed-effect regression analysis. Current Developments in Nutrition . 2022;6(8).	**PS, TA, MAR**	**February 14, 2023**
**18**	Mihrshahi S, Kabir I, Roy SK, Agho KE, Senarath U, Dibley MJ. Determinants of infant and young child feeding practices in Bangladesh: Secondary data analysis of Demographic and Health Survey 2004. Food Nutr Bull. 2010;31(2).	**PS, TA, MAR**	**February 14, 2023**
**19**	Nguyen PH, Avula R, Ruel MT, Saha KK, Ali D, Tran LM, et al. Maternal and child dietary diversity are associated in bangladesh, vietnam, and ethiopia. Journal of Nutrition. 2013 Jul 1;143(7):1176–83.	**PS, TA, MAR**	**February 15, 2023**
**20**	Rahman MA, Khan MN, Akter S, Rahman A, Alam MM, Khan MA, et al. Determinants of exclusive breastfeeding practice in Bangladesh: Evidence from nationally representative survey data. PLoS One. 2020 Jul 1;15(7).	**PS, TA, MAR**	**February 16, 2023**
**21**	Raihana S, Alam A, Huda TM, Dibley MJ. Factors associated with delayed initiation of breastfeeding in health facilities: secondary analysis of Bangladesh demographic and health survey 2014. Int Breastfeed J. 2021 Dec 1;16(1).	**SSA, TA, MAR**	**February 16, 2023**
**22**	Rana MM, Islam MR, Karim MR, Islam AZ, Haque MA, Shahiduzzaman M, et al. Knowledge and practices of exclusive breastfeeding among mothers in rural areas of Rajshahi district in Bangladesh: A community clinic based study. PLoS One. 2020 May 1;15(5).	**SSA, TA, MAR**	**February 16, 2023**
**23**	Rasheed S, Frongillo EA, Devine CM, Alam DS, Rasmussen KM. Maternal, infant, and household factors are associated with breast-feeding trajectories during infants' first 6 months of life in Matlab, Bangladesh. Journal of Nutrition. 2009 Aug;139(8):1582–7.	**TA, MAR, PS**	**February 18, 2023**
**24**	Sheikh N, Akram R, Ali N, Haque SR, Tisha S, Mahumud RA, et al. Infant and young child feeding practice, dietary diversity, associated predictors, and child health outcomes in Bangladesh. Journal of Child Health Care. 2019 Jun 1;24(2):260–73.	**SSA, TA, PS, MAR**	**February 19, 2023**

9. Dataset for meta-analysis

Table s4.1: Early initiation of breastfeeding

Study	Year of data	Components	Comparison group	Reference group	Outcome measure	Effect size	95% CI (lower bound)	95% CI (upper bound)	Sample size (N)
Ahmed, 2022	BDHS 2017-18	EIBF	Occupation	No occupation	OR of providing EIBF	0.93	0.78	1.10	2137
Hasan, 2020	Survey 2019	EIBF	Occupation	No occupation	OR of providing EIBF	0.35	0.22	0.56	422
Raihana, 2021	BDHS 2014	EIBF	Occupation	No occupation	OR of providing EIBF	1.09	0.81	1.47	1277
Mihrshahi, 2010	BDHS 2004	EIBF	Occupation	No occupation	OR of providing EIBF	0.97	0.75	1.26	2482

Table s4.2: Exclusive breastfeeding

Study	Year of data	Study level	Components	Comparison group	Reference group	Outcome measure	Effect size	95% CI (lower bound)	95% CI (upper bound)	Sample size (N)
Ahmed, 2022	BDHS 2017-18	National	EBF	Occupation	No occupation	OR of providing EBF	0.68	0.48	0.95	665
Blackstone, 2018	BDHS 2007	National	EBF	Occupation	No occupation	OR of providing EBF	0.75	0.38	1.46	515
Haider, 2019	Survey 2015-17	Sub-national	EBF	Occupation	No occupation	OR of providing EBF	0.34	0.11	0.87	304
Hasan, 2021	Survey 2019	Sub-national	EBF	Occupation	No occupation	OR of providing EBF	0.14	0.08	0.23	385

Study	Year of data	Study level	Components	Comparison group	Reference group	Outcome measure	Effect size	95% CI (lower bound)	95% CI (upper bound)	Sample size (N)
Rana, 2020	Survey 2015	Sub-national	EBF	Occupation	No occupation	OR of providing EBF	0.05	0.02	0.11	513
Rasheed, 2009	Survey 2002-2004	Sub-national	EBF	Occupation	No occupation	OR of providing EBF	0.49	0.19	1.32	810
Mihrshahi, 2010	BDHS 2004	National	EBF	Occupation	No occupation	OR of providing EBF	0.54	0.42	0.69	677

Table s4.3: Complementary feeding

Study	Year of data	Study level	Components	Comparison group	Reference group	Outcome measure	Effect size	95% CI (lower bound)	95% CI (upper bound)	Sample size (N)
Blackstone, 2018	BDHS 2011	National	MDD/MAD	Occupation	No occupation	OR of providing MDD	0.73	0.58	1.27	2264
Kabir, 2012	BDHS 2007	National	MDD/MAD	Occupation	No occupation	OR of providing MAD	1.26	0.99	1.60	1727
Kundu, 2022	Survey 2019	Sub-national	MDD/MAD	Occupation	No occupation	OR of providing MDD	1.67	0.88	3.39	1190
Nguyen, 2013	Survey 2010	Sub-national	MDD/MAD	Occupation	No occupation	OR of providing MDD	0.64	0.31	1.30	1211
Sheikh, 2019	BDHS 2014	National	MDD/MAD	Occupation	No occupation	OR of providing MAD	1.22	0.95	1.56	2331

10. Strategy for handling missing data

Not applicable

11. List of all articles found in databases searches for title and abstract and full text screening

SL	Articles excluded during title and abstract screening	Decision
1.	Mapping routine measles vaccination in low-and middle-income countries. Nature. 2021;589(7842):415-9.	Title and abstract not relevant- excluded
2.	Abdulla F, El-Raouf MA, Rahman A, Aldallal R, Mohamed MS, Hossain MM. Prevalence and determinants of wasting among under-5 Egyptian children: Application of quantile regression. Food Science & Nutrition. 2023;11(2):1073-83.	Title and abstract not relevant- excluded
3.	Abdulla F, Rahman A, Hossain MM. Prevalence and risk predictors of childhood stunting in Bangladesh. PLoS One. 2023;18(1):e0279901.	Title and abstract not relevant- excluded
4.	Abokyi E, Asante BO, Wongnaa CA. Women's role of caregiving for under-five children: Implications for dietary diversity and food security in Ghana. Cogent Food & Agriculture. 2023;9(1):2153415.	Title and abstract not relevant- excluded
5.	Adams KP, Ayifah E, Phiri TE, Mridha MK, Adu-Afarwuah S, Arimond M, et al. Maternal and child supplementation with lipid-based nutrient supplements, but not child supplementation alone, decreases self-reported household food insecurity in some settings. The Journal of nutrition. 2017;147(12):2309-18.	Title and abstract not relevant- excluded
6.	Adams MS, Khan N, Begum S, Wirz S, Hesketh T, Pring T. Feeding difficulties in children with cerebral palsy: low-cost caregiver training in Dhaka, Bangladesh. Child: care, health and development. 2012;38(6):878-88.	Title and abstract not relevant- excluded
7.	Adane M, Mengistie B, Mulat W, Kloos H, Medhin G. Utilization of health facilities and predictors of health-seeking behavior for under-five children with acute diarrhea in slums of Addis Ababa, Ethiopia: a community-based cross-sectional study. Journal of Health, Population and Nutrition. 2017;36:1-12.	Title and abstract not relevant- excluded
8.	Adebayo FA, Itkonen ST, Öhman T, Skaffari E, Saarnio EM, Erkkola M, et al. Vitamin D intake, serum 25-hydroxyvitamin D status and response to moderate vitamin D3 supplementation: a randomised controlled trial in East African and Finnish women. British Journal of Nutrition. 2018;119(4):431-41.	Title and abstract not relevant- excluded

SL	Articles excluded during title and abstract screening	Decision
9.	Afsana K, Haque MR, Sobhan S, Shahin SA. BRAC's experience in scaling-up MNP in Bangladesh. Asia Pacific journal of clinical nutrition. 2014;23(3):377-84.	Title and abstract not relevant- excluded
10.	Agho KE, Ezeh OK, Ferdous AJ, Mbugua I, Kamara JK. Factors associated with under-5 mortality in three disadvantaged East African districts. International health. 2020;12(5):417-28.	Title and abstract not relevant- excluded
11.	Aguayo VM, Badgaiyan N, Paintal K. Determinants of child stunting in the R oyal K ingdom of B hutan: an in-depth analysis of nationally representative data. Maternal & child nutrition. 2015;11(3):333-45.	Title and abstract not relevant- excluded
12.	Ahishakiye J, Bouwman L, Brouwer ID, Matsiko E, Armar-Klemesu M, Koelen M. Challenges and responses to infant and young child feeding in rural Rwanda: a qualitative study. Journal of Health, Population and Nutrition. 2019;38:1-10.	Title and abstract not relevant- excluded
13.	Ahmad SM, Raqib R, Qadri F, Stephensen CB. The effect of newborn vitamin A supplementation on infant immune functions: trial design, interventions, and baseline data. Contemporary Clinical Trials. 2014;39(2):269-79.	Title and abstract not relevant- excluded
14.	Ahmed B. Differential fertility in Bangladesh: a path analysis. Social Biology. 1981;28(1-2):102-10.	Title and abstract not relevant- excluded
15.	Ahmed F. Nutritional situation of Dhaka. The Southeast Asian Journal of Tropical Medicine and Public Health. 1992;23:59-64.	Title and abstract not relevant- excluded
16.	Ahmed F, Khan MR, Akhtaruzzaman M, Karim R, Williams G, Banu CP, et al. Effect of long-term intermittent supplementation with multiple micronutrients compared with iron-and-folic acid supplementation on Hb and micronutrient status of non-anaemic adolescent schoolgirls in rural Bangladesh. British journal of nutrition. 2012;108(8):1484-93.	Title and abstract not relevant- excluded
17.	Ahmed F, Khosravi-Boroujeni H, Khan MR, Roy AK, Raqib R. Prevalence and predictors of vitamin D deficiency and insufficiency among pregnant rural women in Bangladesh. Nutrients. 2021;13(2):449.	Title and abstract not relevant- excluded
18.	Ahmed M, Rashid M, Begum S. Diarrhoea and feeding practices of young children attending two selected urban clinics in Dhaka. Journal of diarrhoeal diseases research. 1992:217-20.	Title and abstract not relevant- excluded
19.	Ahmed T, Islam M, Choudhury N, Hossain I, Huq S, Mahfuz M, et al. Results with complementary food using local food ingredients. Complementary feeding: building the foundations for a healthy life. 87: Karger Publishers; 2017. p. 103-13.	Title and abstract not relevant- excluded
20.	Ahmed T, Mahfuz M, Islam MM, Mondal D, Hossain MI, Ahmed AS, et al. The MAL-ED cohort study in Mirpur, Bangladesh. Clinical Infectious Diseases. 2014;59(suppl_4):S280-S6.	Title and abstract not relevant- excluded
21.	Akbar M, Preston V. Entrepreneurial activities of Canadian Bangladeshi women in Toronto: a family perspective. Journal of Ethnic and Migration Studies. 2023;49(11):2817-36.	Title and abstract not relevant- excluded
22.	Akhtar E, Mily A, Haq A, Al-Mahmud A, El-Arifeen S, Hel Baqui A, et al. Prenatal high-dose vitamin D 3 supplementation has balanced effects on cord blood Th1 and Th2 responses. Nutrition journal. 2015;15:1-11.	Title and abstract not relevant- excluded
23.	Akhter S, Larson CP. Willingness to pay for zinc treatment of childhood diarrhoea in a rural population of Bangladesh. Health policy and planning. 2010;25(3):230-6.	Title and abstract not relevant- excluded

SL	Articles excluded during title and abstract screening	Decision
24.	Akpan E, Hossain SJ, Devine A, Braat S, Hasan MI, Tipu SMU, et al. Cost-effectiveness of universal iron supplementation and iron-containing micronutrient powders for anemia among young children in rural Bangladesh: analysis of a randomized, placebo-controlled trial. The American Journal of Clinical Nutrition. 2022;116(5):1303-13.	Title and abstract not relevant- excluded
25.	Akseer N, Keats EC, Thurairajah P, Cousens S, Bétran AP, Oaks BM, et al. Characteristics and birth outcomes of pregnant adolescents compared to older women: An analysis of individual level data from 140,000 mothers from 20 RCTs. EClinicalMedicine. 2022;45.	Title and abstract not relevant- excluded
26.	Akter F, Hossain MM, Shamim AA, Khan MSA, Hasan M, Hanif AAM, et al. Prevalence and socio-economic determinants of inadequate dietary diversity among adolescent girls and boys in Bangladesh: findings from a nationwide cross-sectional survey. Journal of Nutritional Science. 2021;10:e103.	Title and abstract not relevant- excluded
27.	Akter S, Rahman MM. The determinants of early cessation of breastfeeding in Bangladesh. World health & population. 2010;11(4):5-12.	Title and abstract not relevant- excluded
28.	Alam A, Rasheed S, Khan NU, Sharmin T, Huda TM, Arifeen SE, et al. How can formative research inform the design of an iron-folic acid supplementation intervention starting in first trimester of pregnancy in Bangladesh? BMC public health. 2015;15:1-9.	Title and abstract not relevant- excluded
29.	Alam MS. For Domestic Use Only: The Perception of Power and Powerlessness Among Rural Muslim Women in a Bangladesh Village: University of Arkansas, Fayetteville; 2006.	Title and abstract not relevant- excluded
30.	Ali H, Rahman H, Lee LC, Khan NZ, Wu LSF, Mehra S, et al. Autism spectrum disorder in a rural community in Bangladesh: a mid-childhood assessment. Autism Research. 2022;15(2):328-39.	Title and abstract not relevant- excluded
31.	Ali NB, Priyanka SS, Bhui BR, Herrera S, Azad MR, Karim A, et al. Prevalence and factors associated with skin-to-skin contact (SSC) practice: findings from a population-based cross-sectional survey in 10 selected districts of Bangladesh. BMC Pregnancy and Childbirth. 2021;21:1-13.	Title and abstract not relevant- excluded
32.	Amin R, Li Y. NGO-promoted women's credit program, immunization coverage, and child mortality in rural Bangladesh. Women & Health. 1997;25(1):71-87.	Title and abstract not relevant- excluded
33.	Anderson C, Hafen R, Sofrygin O, Ryan L, Community H. Comparing predictive abilities of longitudinal child growth models. Statistics in medicine. 2019;38(19):3555-70.	Title and abstract not relevant- excluded
34.	Anderson R, Williams A, Jess N, Read JM, Limmer M. The impact of professional midwives and mentoring on the quality and availability of maternity care in government sub-district hospitals in Bangladesh: a mixed-methods observational study. BMC Pregnancy and Childbirth. 2022;22(1):827.	Title and abstract not relevant- excluded
35.	Angdembe MR, Dulal BP, Bhattarai K, Karn S. Trends and predictors of inequality in childhood stunting in Nepal from 1996 to 2016. International journal for equity in health. 2019;18:1-17.	Title and abstract not relevant- excluded
36.	Arar NH. Cultural responses to water shortage among Palestinians in Jordan: the water crisis and its impact on child health. Human Organization. 1998;57(3):284-91.	Title and abstract not relevant- excluded
37.	Aremu O, Lawoko S, Dalal K. Childhood vitamin A capsule supplementation coverage in Nigeria: a multilevel analysis of geographic and socioeconomic inequities. The Scientific World Journal. 2010;10(1):1901-14.	Title and abstract not relevant- excluded

SL	Articles excluded during title and abstract screening	Decision
38.	Arnold BF, Null C, Luby SP, Unicomb L, Stewart CP, Dewey KG, et al. Cluster-randomised controlled trials of individual and combined water, sanitation, hygiene and nutritional interventions in rural Bangladesh and Kenya: the WASH Benefits study design and rationale. BMJ open. 2013;3(8):e003476.	Title and abstract not relevant- excluded
39.	Arsenault JE, Brown KH. Dietary protein intake in young children in selected low-income countries is generally adequate in relation to estimated requirements for healthy children, except when complementary food intake is low. The Journal of nutrition. 2017;147(5):932-9.	Title and abstract not relevant- excluded
40.	Asamane EA, Quinn L, Watson SI, Lilford RJ, Hemming K, Sidibe C, et al. Protocol for a parallel group, two-arm, superiority cluster randomised trial to evaluate a community-level complementary-food safety and hygiene and nutrition intervention in Mali: the MaaCiwara study (version 1.3; 10 November 2022). Trials. 2023;24(1):68.	Title and abstract not relevant- excluded
41.	Ashish K, Gurung R, Kinney MV, Sunny AK, Moinuddin M, Basnet O, et al. Effect of the COVID-19 pandemic response on intrapartum care, stillbirth, and neonatal mortality outcomes in Nepal: a prospective observational study. The lancet Global health. 2020;8(10):e1273-e81.	Title and abstract not relevant- excluded
42.	Ashraf S, Islam M, Unicomb L, Rahman M, Winch PJ, Arnold BF, et al. Effect of improved water quality, sanitation, hygiene and nutrition interventions on respiratory illness in young children in rural Bangladesh: a multi-arm cluster-randomized controlled trial. The American Journal of Tropical Medicine and Hygiene. 2020;102(5):1124.	Title and abstract not relevant- excluded
43.	Ashworth A, Shrimpton R, Jamil K. Growth monitoring and promotion: review of evidence of impact. Maternal & child nutrition. 2008;4:86-117.	Title and abstract not relevant- excluded
44.	Azage M, Kumie A, Worku A, Bagtzoglou AC. Childhood diarrhea in high and low hotspot districts of Amhara Region, northwest Ethiopia: a multilevel modeling. Journal of health, population and nutrition. 2016;35:1-14.	Title and abstract not relevant- excluded
45.	Azhar BS, Islam MS, Karim MR. Prevalence of anemia and associated risk factors among pregnant women attending antenatal care in Bangladesh: a cross-sectional study. Primary Health Care Research & Development. 2021;22:e61.	Title and abstract not relevant- excluded
46.	Ball HL, Moya E, Fairley L, Westman J, Oddie S, Wright J. Infant care practices related to sudden infant death syndrome in South Asian and White British families in the UK. Paediatric and perinatal epidemiology. 2012;26(1):3-12.	Title and abstract not relevant- excluded
47.	Barua P, Beeson JG, Maleta K, Ashorn P, Rogerson SJ. The impact of early life exposure to Plasmodium falciparum on the development of naturally acquired immunity to malaria in young Malawian children. Malaria Journal. 2019;18:1-12.	Title and abstract not relevant- excluded
48.	Bauserman M, Thorsten VR, Nolen TL, Patterson J, Lokangaka A, Tshefu A, et al. Maternal mortality in six low and lower-middle income countries from 2010 to 2018: risk factors and trends. Reproductive health. 2020;17:1-10.	Title and abstract not relevant- excluded
49.	Bbaale E. Determinants of early initiation, exclusiveness, and duration of breastfeeding in Uganda. Journal of health, population, and nutrition. 2014;32(2):249.	Title and abstract not relevant- excluded

SL	Articles excluded during title and abstract screening	Decision
50.	Begum HA, Moneesha SS, Sayem AM. Child care hygiene practices of women migrating from rural to urban areas of bangladesh. Asia Pacific Journal of Public Health. 2013;25(4):345-55.	Title and abstract not relevant- excluded
51.	Begum NNF. Novel facial characteristics in congenital rubella syndrome: a study of 115 cases in a cardiac hospital of Bangladesh. BMJ Paediatrics Open. 2020;4(1).	Title and abstract not relevant- excluded
52.	Begum S, Mahmud T, Rahman T, Zannat J, Khatun F, Nahar K, et al. Knowledge, attitude and practice of Bangladeshi women towards breast cancer: a cross sectional study. Mymensingh Med J. 2019;28(1):96-104.	Title and abstract not relevant- excluded
53.	Benfer KA, Novak I, Morgan C, Whittingham K, Khan NZ, Ware RS, et al. Community-based parent-delivered early detection and intervention programme for infants at high risk of cerebral palsy in a low-resource country (Learning through Everyday Activities with Parents (LEAP-CP): protocol for a randomised controlled trial. BMJ open. 2018;8(6):e021186.	Title and abstract not relevant- excluded
54.	Bhuiya A, Ahmed SM, Chowdhury M. Women focused development intervention reduces neonatal mortality in rural Bangladesh: a study of the pathways of influence. 2003.	Title and abstract not relevant- excluded
55.	Biks GA, Berhane Y, Worku A, Gete YK. Exclusive breast feeding is the strongest predictor of infant survival in Northwest Ethiopia: a longitudinal study. Journal of Health, Population and Nutrition. 2015;34:1-6.	Title and abstract not relevant- excluded
56.	Billah SM, Ali NB, Khan ANS, Raynes-Greenow C, Kelly PJ, Siraj MS, et al. Factors influencing quality nutrition service provision at antenatal care contacts: Findings from a public health facility-based observational study in 21 districts of Bangladesh. PloS one. 2022;17(1):e0262867.	Title and abstract not relevant- excluded
57.	Billah SM, Ferdous TE, Kelly P, Raynes-Greenow C, Siddique AB, Choudhury N, et al. Effect of nutrition counselling with a digital job aid on child dietary diversity: Analysis of secondary outcomes from a cluster randomised controlled trial in rural Bangladesh. Maternal & child nutrition. 2022;18(1):e13267.	Title and abstract not relevant- excluded
58.	Billah SM, Hoque DE, Rahman M, Christou A, Mugo NS, Begum K, et al. Feasibility of engaging “Village Doctors” in the Community-based Integrated Management of Childhood Illness (C-IMCI): experience from rural Bangladesh. Journal of global health. 2018;8(2).	Title and abstract not relevant- excluded
59.	Bizuneh AD, Azeze GG. Knowledge on anaemia and benefit of iron–folic acid supplementation among pregnant mothers attending antenatal care in Woldia town, Northeastern Ethiopia: a facility-based cross-sectional study. Journal of Health, Population and Nutrition. 2022;41(1):32.	Title and abstract not relevant- excluded
60.	Black MM, Baqui AH, Zaman K, Persson LA, El Arifeen S, Le K, et al. Iron and zinc supplementation promote motor development and exploratory behavior among Bangladeshi infants. The American journal of clinical nutrition. 2004;80(4):903-10.	Title and abstract not relevant- excluded
61.	Black R, Fontaine O, Lamberti L, Bhan M, Huicho L, El Arifeen S, et al. Drivers of the reduction in childhood diarrhea mortality 1980-2015 and interventions to eliminate preventable diarrhea deaths by 2030. Journal of global health. 2019;9(2).	Title and abstract not relevant- excluded
62.	Bland B. Commercial solutions to malnutrition. BMJ. 2009;339.	Title and abstract not relevant- excluded

SL	Articles excluded during title and abstract screening	Decision
63.	Brown LV, Rogers BL, Zeitlin MF, Gershoff SN, Huq N, Peterson KE. Comparison of the costs of compliance with nutrition education messages to improve the diets of Bangladeshi breastfeeding mothers and weaning-age children. Ecology of food and nutrition. 1993;30(2):99-126.	Title and abstract not relevant- excluded
64.	Bukachi SA, Ngutu M, Muthiru AW, Lépine A, Kadiyala S, Domínguez-Salas P. Gender and sociocultural factors in animal source foods (ASFs) access and consumption in lower-income households in urban informal settings of Nairobi, Kenya. Journal of Health, Population and Nutrition. 2022;41(1):30.	Title and abstract not relevant- excluded
65.	Callaghan-Koru JA, Seifu A, Tholandi M, de Graft-Johnson J, Daniel E, Rawlins B, et al. Newborn care practices at home and in health facilities in 4 regions of Ethiopia. BMC pediatrics. 2013;13:1-11.	Title and abstract not relevant- excluded
66.	Campbell C, Douglas A, Williams L, Cezard G, Brewster DH, Buchanan D, et al. Are there ethnic and religious variations in uptake of bowel cancer screening? A retrospective cohort study among 1.7 million people in Scotland. BMJ open. 2020;10(10):e037011.	Title and abstract not relevant- excluded
67.	Campbell RK, Aguayo VM, Kang Y, Dzed L, Joshi V, Waid JL, et al. Epidemiology of anaemia in children, adolescent girls, and women in Bhutan. Maternal & child nutrition. 2018;14:e12740.	Title and abstract not relevant- excluded
68.	Canagarajah S, Ashraf H. Multilingualism and education in South Asia: Resolving policy/practice dilemmas. Annual Review of Applied Linguistics. 2013;33:258-85.	Title and abstract not relevant- excluded
69.	Cezard GI, Bhopal RS, Ward HJ, Bansal N, Bhala N. Ethnic variations in upper gastrointestinal hospitalizations and deaths: the Scottish Health and Ethnicity Linkage Study. The European Journal of Public Health. 2016;26(2):254-60.	Title and abstract not relevant- excluded
70.	Chen LC, Ahmed S, Gesche M, Henry Mosley W. Prospective study of birth interval dynamics in rural Bangladesh. Population studies. 1974;28(2):277-97.	Title and abstract not relevant- excluded
71.	Chetley A. Marketing infant foods in developing countries. Lancet (London, England). 1979;2(8145):747-.	Title and abstract not relevant- excluded
72.	Choudhury S, Hossain S, Islam MZ, Akhter S, Arifa S, Hayat SMS-u. Evaluation of attributes to hyperbilirubinaemia in neonates in a tertiary care hospital in the Dhaka city. Bangladesh Journal of Medical Science. 2014;13(1):58-62.	Title and abstract not relevant- excluded
73.	Chow MYK, Khandaker G, McIntyre P. Global childhood deaths from pertussis: a historical review. Clinical Infectious Diseases. 2016;63(suppl_4):S134-S41.	Title and abstract not relevant- excluded
74.	Chowdhury AMR, Bhuiya A, Chowdhury ME, Rasheed S, Hussain Z, Chen LC. The Bangladesh paradox: exceptional health achievement despite economic poverty. The Lancet. 2013;382(9906):1734-45.	Title and abstract not relevant- excluded
75.	Chowdhury HA, Ahmed KR, Jebunessa F, Akter J, Hossain S, Shahjahan M. Factors associated with maternal anaemia among pregnant women in Dhaka city. BMC women's health. 2015;15:1-6.	Title and abstract not relevant- excluded
76.	Chowdhury S, Thow AM, Uddin MS, Akter S, Das S, Iqbal M, et al. Policy content and stakeholder network analysis for infant and young child feeding in Bangladesh. 2017.	Title and abstract not relevant- excluded
77.	Chowdhury TA. Applying and extending the sustainable livelihoods approach: Identifying the livelihood capitals and well-being achievements of indigenous people in Bangladesh. Journal of Social and Economic Development. 2021;23(2):302-20.	Title and abstract not relevant- excluded

SL	Articles excluded during title and abstract screening	Decision
78.	Chowdhury ZT, Hurley KM, Campbell RK, Shaikh S, Shamim AA, Mehra S, et al. Novel method for estimating nutrient intakes using a semistructured 24-hour diet recall for infants and young children in rural Bangladesh. Current Developments in Nutrition. 2020;4(9):nzaa123.	Title and abstract not relevant- excluded
79.	Christian P, Klemm R, Shamim AA, Ali H, Rashid M, Shaikh S, et al. Effects of vitamin A and β-carotene supplementation on birth size and length of gestation in rural Bangladesh: a cluster-randomized trial. The American journal of clinical nutrition. 2013;97(1):188-94.	Title and abstract not relevant- excluded
80.	Christian P, Lee SE, Donahue Angel M, Adair LS, Arifeen SE, Ashorn P, et al. Risk of childhood undernutrition related to small-for-gestational age and preterm birth in low-and middle-income countries. International journal of epidemiology. 2013;42(5):1340-55.	Title and abstract not relevant- excluded
81.	Combs G, Hassan N. The Chakaria food system study: household-level, case–control study to identify risk factor for rickets in Bangladesh. European journal of clinical nutrition. 2005;59(11):1291-301.	Title and abstract not relevant- excluded
82.	Connor NE, Islam MS, Arvay ML, Baqui AH, Zaidi AK, Soofi SB, et al. Methods employed in monitoring and evaluating field and laboratory systems in the ANISA study: ensuring quality. The Pediatric Infectious Disease Journal. 2016;35(5):S39-S44.	Title and abstract not relevant- excluded
83.	Cook EJ, Powell F, Ali N, Penn-Jones C, Ochieng B, Randhawa G. Improving support for breastfeeding mothers: a qualitative study on the experiences of breastfeeding among mothers who reside in a deprived and culturally diverse community. International Journal for Equity in Health. 2021;20:1-14.	Title and abstract not relevant- excluded
84.	Cormick G, Betran AP, Romero IB, García-Casal MN, Perez SM, Gibbons L, et al. Impact of flour fortification with calcium on calcium intake: a simulation study in seven countries. Annals of the New York Academy of Sciences. 2021;1493(1):59-74.	Title and abstract not relevant- excluded
85.	Cormick G, Gibbons L, Belizán JM. Impact of water fortification with calcium on calcium intake in different countries: a simulation study. Public Health Nutrition. 2022;25(2):344-57.	Title and abstract not relevant- excluded
86.	Cousens S, Nacro B, Curtis V, Kanki B, Tall F, Traore E, et al. Prolonged breast-feeding: no association with increased risk of clinical malnutrition in young children in Burkina Faso. Bulletin of the World Health Organization. 1993;71(6):713.	Title and abstract not relevant- excluded
87.	Cromwell EA, Osborne JC, Unnasch TR, Basáñez M-G, Gass KM, Barbre KA, et al. Predicting the environmental suitability for onchocerciasis in Africa as an aid to elimination planning. PLoS neglected tropical diseases. 2021;15(7):e0008824.	Title and abstract not relevant- excluded
88.	Darmstadt GL, Baqui AH, Choi Y, Bari S, Rahman SM, Mannan I, et al. Validation of community health workers' assessment of neonatal illness in rural Bangladesh. Bulletin of the World Health Organization. 2009;87:12-9.	Title and abstract not relevant- excluded
89.	Das S, Fahim SM, Alam MA, Mahfuz M, Bessong P, Mduma E, et al. Not water, sanitation and hygiene practice, but timing of stunting is associated with recovery from stunting at 24 months: results from a multi-country birth cohort study. Public health nutrition. 2021;24(6):1428-37.	Title and abstract not relevant- excluded
90.	Das S, Hossain M. Levels And Determinants Of Child Undernutrition In Bangladesh. Pakistan Journal of Statistics. 2008;24(4).	Title and abstract not relevant- excluded

SL	Articles excluded during title and abstract screening	Decision
91.	Davies-Adetugbo AA. Sociocultural factors and the promotion of exclusive breastfeeding in rural Yoruba communities of Osun State, Nigeria. Social science & medicine. 1997;45(1):113-25.	Title and abstract not relevant- excluded
92.	Davis MA, Higgins J, Li Z, Gilbert-Diamond D, Baker ER, Das A, et al. Preliminary analysis of in utero low-level arsenic exposure and fetal growth using biometric measurements extracted from fetal ultrasound reports. Environmental Health. 2015;14:1-11.	Title and abstract not relevant- excluded
93.	Davis MA, Li Z, Gilbert-Diamond D, Mackenzie TA, Cottingham KL, Jackson BP, et al. Infant toenails as a biomarker of in utero arsenic exposure. Journal of exposure science & environmental epidemiology. 2014;24(5):467-73.	Title and abstract not relevant- excluded
94.	de Balcazar YS, Balcazar FE. Child survival in the Third World: a functional analysis of oral rehydration therapy dissemination campaigns. Behaviour Change. 1991;8(1):26-34.	Title and abstract not relevant- excluded
95.	de Hoop T, Fallon S, Yunus FM, Munrat S, Jolly SP, Sehrin F, et al. Mothers' education and the effectiveness of nutrition programmes: evidence from a matched cross-sectional study in rural Bangladesh. Journal of Development Effectiveness. 2020;12(4):279-97.	Title and abstract not relevant- excluded
96.	De Moura FF, Moursi M, Angel MD, Angeles-Agdeppa I, Atmarita A, Gironella GM, et al. Biofortified β-carotene rice improves vitamin A intake and reduces the prevalence of inadequacy among women and young children in a simulated analysis in Bangladesh, Indonesia, and the Philippines. The American journal of clinical nutrition. 2016;104(3):769-75.	Title and abstract not relevant- excluded
97.	Devasenapathy N, Neogi SB, Soundararajan S, Ahmad D, Hazra A, Ahmad J, et al. Association of antenatal care and place of delivery with newborn care practices: evidence from a cross-sectional survey in rural Uttar Pradesh, India. Journal of Health, Population and Nutrition. 2017;36:1-12.	Title and abstract not relevant- excluded
98.	Dewey KG, Matias SL, Mridha MK, Arnold CD. Nutrient supplementation during the first 1000 days and growth of infants born to pregnant adolescents. Annals of the New York Academy of Sciences. 2020;1468(1):25-34.	Title and abstract not relevant- excluded
99.	Dewey KG, Mridha MK, Matias SL, Arnold CD, Cummins JR, Khan MSA, et al. Lipid-based nutrient supplementation in the first 1000 d improves child growth in Bangladesh: a cluster-randomized effectiveness trial. The American journal of clinical nutrition. 2017;105(4):944-57.	Title and abstract not relevant- excluded
100.	Dewey KG, Wessells KR, Arnold CD, Prado EL, Abbeddou S, Adu-Afarwuah S, et al. Characteristics that modify the effect of small-quantity lipid-based nutrient supplementation on child growth: an individual participant data meta-analysis of randomized controlled trials. The American journal of clinical nutrition. 2021;114:15S-42S.	Title and abstract not relevant- excluded
101.	Driezen P, Abdullah AS, Nargis N, Hussain AG, Fong GT, Thompson ME, et al. Awareness of tobacco-related health harms among vulnerable populations in Bangladesh: findings from the international tobacco control (ITC) Bangladesh survey. International journal of environmental research and public health. 2016;13(9):848.	Title and abstract not relevant- excluded
102.	Ehsan SMA, Jahan F. Analysing the impact of COVID-19 on the mothers of Bangladesh: hearing the unheard. Journal of Public Health. 2021:1-14.	Title and abstract not relevant- excluded

SL	Articles excluded during title and abstract screening	Decision
103.	Ekström E-C, Lindström E, Raqib R, El Arifeen S, Basu S, Brismar K, et al. Effects of prenatal micronutrient and early food supplementation on metabolic status of the offspring at 4.5 years of age. The MINIMat randomized trial in rural Bangladesh. International journal of epidemiology. 2016;45(5):1656-67.	Title and abstract not relevant- excluded
104.	Emran TB, Rahman MA, Uddin MMN, Rahman MM, Uddin MZ, Dash R, et al. Effects of organic extracts and their different fractions of five Bangladeshi plants on in vitro thrombolysis. BMC complementary and alternative medicine. 2015;15:1-8.	Title and abstract not relevant- excluded
105.	Engle PL, Castle S, Menon P. Child development: Vulnerability and resilience. Social science & medicine. 1996;43(5):621-35.	Title and abstract not relevant- excluded
106.	Engle-Stone R, Kumordzie SM, Meinzen-Dick L, Vosti SA. Replacing iron-folic acid with multiple micronutrient supplements among pregnant women in Bangladesh and Burkina Faso: costs, impacts, and cost-effectiveness. Annals of the New York Academy of Sciences. 2019;1444(1):35-51.	Title and abstract not relevant- excluded
107.	Entwistle F. Using qualitative research findings to analyse how breastfeeding public health recommendations can be tailored to meet the needs of women of Bangladeshi origin living in England. Journal of Research in Nursing. 2012;17(2):179-80.	Title and abstract not relevant- excluded
108.	Exposito ABP. Community intervention to assess the effects of orange-flesh sweet potatoes and vitamin A supplements on mineral absorption from rice-based meals and intestinal mucosal permeability in vitamin A-depleted Bangladeshi women: University of California, Davis; 2009.	Title and abstract not relevant- excluded
109.	Ezeh OK, Abir T, Zainol NR, Al Mamun A, Milton AH, Haque MR, et al. Trends of stunting prevalence and its associated factors among nigerian children aged 0–59 months residing in the northern nigeria, 2008–2018. Nutrients. 2021;13(12):4312.	Title and abstract not relevant- excluded
110.	Fall CH, Fisher DJ, Osmond C, Margetts BM. Multiple micronutrient supplementation during pregnancy in low-income countries: a meta-analysis of effects on birth size and length of gestation. Food and nutrition bulletin. 2009;30(4_suppl4):S533-S46.	Title and abstract not relevant- excluded
111.	Faruk A, Al Quddus I. COVID-19 vaccination: Willingness and practice in Bangladesh. Development Policy Review. 2023;41(1):e12645.	Title and abstract not relevant- excluded
112.	Farzana FD, Rahman AS, Sultana S, Raihan MJ, Haque MA, Waid JL, et al. Coping strategies related to food insecurity at the household level in Bangladesh. PloS one. 2017;12(4):e0171411.	Title and abstract not relevant- excluded
113.	Fauveau V, Wojtyniak B, Chakraborty J, Sarder AM, Briend A. The effect of maternal and child health and family planning services on mortality: Is prevention enough? British medical journal. 1990;301(6743):103-7.	Title and abstract not relevant- excluded
114.	Feigin VL, Nichols E, Alam T, Bannick MS, Beghi E, Blake N, et al. Global, regional, and national burden of neurological disorders, 1990–2016: a systematic analysis for the Global Burden of Disease Study 2016. The Lancet Neurology. 2019;18(5):459-80.	Title and abstract not relevant- excluded
115.	Ferdous J, Khatun S, Biswas SK, Pervin S, Akter L, Keya KA, et al. Low serum folate level and increased risk of invasive cervical cancer in Bangladeshi women. Bangladesh Journal of Obstetrics & Gynaecology. 2016;31(1):23-7.	Title and abstract not relevant- excluded

SL	Articles excluded during title and abstract screening	Decision
116.	Fiedler JL, Lividini K, Drummond E, Thilsted SH. Strengthening the contribution of aquaculture to food and nutrition security: The potential of a vitamin A-rich, small fish in Bangladesh. Aquaculture. 2016;452:291-303.	Title and abstract not relevant- excluded
117.	Figaroa MN, Bellizzi S, Delvaux T, Benova L. Lactational amenorrhoea among adolescent girls in low-income and middle-income countries: a systematic scoping review. BMJ global health. 2020;5(10):e002492.	Title and abstract not relevant- excluded
118.	Ford JL, Lopez-Teros V. Prediction of vitamin A stores in young children provides insights into the adequacy of current dietary reference intakes. Current Developments in Nutrition. 2020;4(8):nzaa119.	Title and abstract not relevant- excluded
119.	Ford K, Huffman SL, Chowdhury A, Becker S, Allen H, Menken J. Birth-interval dynamics in rural Bangladesh and maternal weight. Demography. 1989;26:425-37.	Title and abstract not relevant- excluded
120.	Frith AL, Ziaei S, Naved RT, Khan AI, Kabir I, Ekström E-C. Breast-feeding counselling mitigates the negative association of domestic violence on exclusive breast-feeding duration in rural Bangladesh. The MINIMat randomized trial. Public health nutrition. 2017;20(15):2810-8.	Title and abstract not relevant- excluded
121.	Gabida M, Chemhuru M, Tshimanga M, Gombe NT, Takundwa L, Bangure D. Effect of distribution of educational material to mothers on duration and severity of diarrhoea and pneumonia, Midlands Province, Zimbabwe: a cluster randomized controlled trial. International breastfeeding journal. 2015;10:1-12.	Title and abstract not relevant- excluded
122.	George AS, Jacobs T, Kinney MV, Haakenstad A, Singh NS, Rasanathan K, et al. Are rhetorical commitments to adolescents reflected in planning documents? An exploratory content analysis of adolescent sexual and reproductive health in Global Financing Facility country plans. Reproductive health. 2021;18:1-13.	Title and abstract not relevant- excluded
123.	Gernand AD, Paul RR, Ullah B, Taher MA, Witter FR, Wu L, et al. A home calendar and recall method of last menstrual period for estimating gestational age in rural Bangladesh: a validation study. Journal of Health, Population and Nutrition. 2016;35:1-9.	Title and abstract not relevant- excluded
124.	Ghafur S. Gender implications of space use in home-based work: evidences from slums in Bangladesh. Habitat international. 2002;26(1):33-50.	Title and abstract not relevant- excluded
125.	Ghosh S, Sen LC, Mali SK, Islam MM, Bakchi J. The role of rural women in household food security and nutrition management in Bangladesh. Asian Journal of Women's Studies. 2021;27(3):441-59.	Title and abstract not relevant- excluded
126.	Gitungwa H, Gustafson C, Jimenez E, Peterson E, Mwanzalila M, Makweta A, et al. Female and male-controlled livestock holdings impact pastoralist food security and women’s dietary diversity. One Health Outlook. 2021;3:1-13.	Title and abstract not relevant- excluded
127.	Goudet S, Murira Z, Torlesse H, Hatchard J, Busch-Hallen J. Effectiveness of programme approaches to improve the coverage of maternal nutrition interventions in South Asia. Maternal & child nutrition. 2018;14:e12699.	Title and abstract not relevant- excluded
128.	Goudet SM, Bogin BA, Madise NJ, Griffiths PL. Nutritional interventions for preventing stunting in children (birth to 59 months) living in urban slums in low-and middle-income countries (LMIC). Cochrane Database of Systematic Reviews. 2019(6).	Title and abstract not relevant- excluded

SL	Articles excluded during title and abstract screening	Decision
129.	Goudet SM, Griffiths PL, Bogin BA, Selim N. Impact of flooding on feeding practices of infants and young children in Dhaka, Bangladesh Slums: what are the coping strategies? Maternal & child nutrition. 2011;7(2):198-214.	Title and abstract not relevant- excluded
130.	Graham SM, Ahmed T, Amanullah F, Browning R, Cardenas V, Casenghi M, et al. Evaluation of tuberculosis diagnostics in children: 1. Proposed clinical case definitions for classification of intrathoracic tuberculosis disease. Consensus from an expert panel. Journal of Infectious Diseases. 2012;205(suppl_2):S199-S208.	Title and abstract not relevant- excluded
131.	Group NVASE. Early neonatal vitamin A supplementation and infant mortality: an individual participant data meta-analysis of randomised controlled trials. Archives of disease in childhood. 2019;104(3):217-26.	Title and abstract not relevant- excluded
132.	Haaland K, Sitaraman S. Increased breastfeeding; an educational exchange program between India and Norway improving newborn health in a low-and middle-income hospital population. Journal of Health, Population and Nutrition. 2022;41(1):16.	Title and abstract not relevant- excluded
133.	Haider R. ICDDR, B's creche promotes breastfeeding and supports mothers in the workplace. Glimpse (Dhaka, Bangladesh). 1999;21(1-2):6.	Title and abstract not relevant- excluded
134.	Haider R, Rasheed S, Sanghvi TG, Hassan N, Pachon H, Islam S, et al. Breastfeeding in infancy: identifying the program-relevant issues in Bangladesh. International Breastfeeding Journal. 2010;5:1-12.	Title and abstract not relevant- excluded
135.	Haider R, Saha KK. Breastfeeding and infant growth outcomes in the context of intensive peer counselling support in two communities in Bangladesh. International breastfeeding journal. 2016;11:1-10.	Title and abstract not relevant- excluded
136.	Haider R, Thorley V, Yourkavitch J. Breastfeeding practices after a counselling intervention for factory workers in Bangladesh. Maternal & Child Nutrition. 2021;17(2):e13113.	Title and abstract not relevant- excluded
137.	Hamadani JD, Mehrin SF, Tofail F, Hasan MI, Huda SN, Baker-Henningham H, et al. Integrating an early childhood development programme into Bangladeshi primary health-care services: an open-label, cluster-randomised controlled trial. The Lancet Global Health. 2019;7(3):e366-e75.	Title and abstract not relevant- excluded
138.	Hamadani JD, Nahar B, Huda SN, Tofail F. Integrating early child development programs into health and nutrition services in Bangladesh: benefits and challenges. Annals of the New York Academy of Sciences. 2014;1308(1):192-203.	Title and abstract not relevant- excluded
139.	Hamadani JD, Tofail F, Cole T, Grantham-McGregor S. The relation between age of attainment of motor milestones and future cognitive and motor development in B angladeshi children. Maternal & child nutrition. 2013;9:89-104.	Title and abstract not relevant- excluded
140.	Hamiduzzaman M, De-Bellis A, Abigail W, Fletcher A. Critical social framework on the determinants of primary healthcare access and utilisation. Family Medicine and Community Health. 2021;9(Suppl 1).	Title and abstract not relevant- excluded
141.	Hampel D, Shahab-Ferdows S, Domek JM, Siddiqua T, Raqib R, Allen LH. Competitive chemiluminescent enzyme immunoassay for vitamin B12 analysis in human milk. Food Chemistry. 2014;153:60-5.	Title and abstract not relevant- excluded
142.	Harris-Fry HA, Azad K, Younes L, Kuddus A, Shaha S, Nahar T, et al. Formative evaluation of a participatory women's group intervention to improve reproductive and women's health outcomes in rural Bangladesh: a controlled before and after study. J Epidemiol Community Health. 2016;70(7):663-70.	Title and abstract not relevant- excluded

SL	Articles excluded during title and abstract screening	Decision
143.	Harris-Fry HA, Paudel P, Harrisson T, Shrestha N, Jha S, Beard BJ, et al. Participatory women's groups with cash transfers can increase dietary diversity and micronutrient adequacy during pregnancy, whereas women's groups with food transfers can increase equity in intrahousehold energy allocation. The Journal of nutrition. 2018;148(9):1472-83.	Title and abstract not relevant- excluded
144.	Hasan AR, Smith G, Selim MA, Akter S, Khan NUZ, Sharmin T, et al. Work and breast milk feeding: a qualitative exploration of the experience of lactating mothers working in ready made garments factories in urban Bangladesh. International Breastfeeding Journal. 2020;15:1-11.	Title and abstract not relevant- excluded
145.	Hasan M, Islam MM, Mubarak E, Haque MA, Choudhury N, Ahmed T. Mother's dietary diversity and association with stunting among children< 2 years old in a low socio-economic environment: A case–control study in an urban care setting in Dhaka, Bangladesh. Maternal & child nutrition. 2019;15(2):e12665.	Title and abstract not relevant- excluded
146.	Hasan MMI, Hassan MM, Mohanta RC, Miah MAH, Harun-Or-Rashid M, Juyena NS. A comparative study on productive, reproductive and ovarian features of repeat breeder and normal cyclic cows in the selected areas of Bangladesh. Journal of Advanced Veterinary and Animal Research. 2018;5(3):324-31.	Title and abstract not relevant- excluded
147.	Hasan MT, Magalhaes RJS, Williams GM, Mamun AA. Long-term changes in childhood malnutrition are associated with long-term changes in maternal BMI: evidence from Bangladesh, 1996–2011. The American journal of clinical nutrition. 2016;104(4):1121-7.	Title and abstract not relevant- excluded
148.	Hasan MZ, Biswas NK, Aziz AM, Chowdhury J, Haider SS, Sarker M. Clinical profile and short-term outcomes of RT-PCR-positive patients with COVID-19: a cross-sectional study in a tertiary care hospital in Dhaka, Bangladesh. BMJ open. 2021;11(12):e055126.	Title and abstract not relevant- excluded
149.	Henderson J, Gao H, Redshaw M. Experiencing maternity care: the care received and perceptions of women from different ethnic groups. BMC pregnancy and childbirth. 2013;13:1-14.	Title and abstract not relevant- excluded
150.	Herlihy JM, Shaikh A, Mazimba A, Gagne N, Grogan C, Mpamba C, et al. Local perceptions, cultural beliefs and practices that shape umbilical cord care: a qualitative study in Southern Province, Zambia. PLoS One. 2013;8(11):e79191.	Title and abstract not relevant- excluded
151.	Hoddinott J, Ahmed A, Karachiwalla NI, Roy S. Nutrition behaviour change communication causes sustained effects on IYCN knowledge in two cluster-randomised trials in Bangladesh. Maternal & Child Nutrition. 2018;14(1):e12498.	Title and abstract not relevant- excluded
152.	Hoque M, Hosono M, Suzuki K. Direct and maternal genetic parameters for measures of feed consumption and feed efficiency in young male Japanese Black cattle. Livestock Science. 2009;122(2-3):333-8.	Title and abstract not relevant- excluded
153.	Hoque SF, Peters R, Whitehead P, Hope R, Hossain MA. River pollution and social inequalities in Dhaka, Bangladesh. Environmental Research Communications. 2021;3(9):095003.	Title and abstract not relevant- excluded
154.	Hossain A, Niroula B, Duwal S, Ahmed S, Kibria MG. Maternal profiles and social determinants of severe acute malnutrition among children under-five years of age: a case-control study in Nepal. Heliyon. 2020;6(5).	Title and abstract not relevant- excluded
155.	Hossain M, Khan M, Ababneh F, Shaw JEH. Identifying factors influencing contraceptive use in Bangladesh: evidence from BDHS 2014 data. BMC public health. 2018;18:1-14.	Title and abstract not relevant- excluded

SL	Articles excluded during title and abstract screening	Decision
156.	Hossain M, Sanin KI, Haque MA, Mbuya MN, Ghosh S, Aksari S, et al. Evaluating the impact of a countrywide, market-based roll-out of multiple micronutrient supplementation on low birth weight in Bangladesh: protocol for a two-arm, quasi-experimental and mixed-methods evaluation study. BMJ open. 2022;12(5):e060230.	Title and abstract not relevant- excluded
157.	Hossain ME, Hoque MA, Giorgi E, Fournié G, Das GB, Henning J. Impact of improved small-scale livestock farming on human nutrition. Scientific Reports. 2021;11(1):191.	Title and abstract not relevant- excluded
158.	Hossain SJ, Roy BR, Sujon HM, Tran T, Fisher J, Tofail F, et al. Effects of integrated psychosocial stimulation (PS) and Unconditional Cash Transfer (UCT) on Children's development in rural Bangladesh: A cluster randomized controlled trial. Social Science & Medicine. 2022;293:114657.	Title and abstract not relevant- excluded
159.	Hossen MR, Biswas S, Ali MA, Halim MA, Ullah MO. In silico peptide-based therapeutics against human colorectal cancer by the activation of TLR5 signaling pathways. Journal of Molecular Modeling. 2023;29(2):35.	Title and abstract not relevant- excluded
160.	Hossen Z, Abrar MA, Ara SR, Hasan MK. RATE-iPATH: On the design of integrated ultrasonic biomarkers for breast cancer detection. Biomedical Signal Processing and Control. 2020;62:102053.	Title and abstract not relevant- excluded
161.	Hotchkiss DR, Godha D, Do M. Expansion in the private sector provision of institutional delivery services and horizontal equity: evidence from Nepal and Bangladesh. Health Policy and Planning. 2014;29(suppl_1):i12-i9.	Title and abstract not relevant- excluded
162.	Huffman SL, Zehner ER, Victora C. Can improvements in breast-feeding practices reduce neonatal mortality in developing countries? Midwifery. 2001;17(2):80-92.	Title and abstract not relevant- excluded
163.	Huq MN, Sarwar KB. Growth Pattern of Exclusively Breastfed Babies in Urban Affluent of Bangladesh. Bangladesh Journal of Medical Science. 2014;13(4):466.	Title and abstract not relevant- excluded
164.	Injury G. Global, regional, and national burden of traumatic brain injury and spinal cord injury, 1990-2016: a systematic analysis for the Global Burden of Disease Study 2016. Lancet Neurol. 2019;18(1):56-87.	Title and abstract not relevant- excluded
165.	Islam A, Maitra C, Pakrashi D, Smyth R. Microcredit programme participation and household food security in rural Bangladesh. Journal of Agricultural Economics. 2016;67(2):448-70.	Title and abstract not relevant- excluded
166.	Islam AHMS, von Braun J, Thorne-Lyman AL, Ahmed AU. Farm diversification and food and nutrition security in Bangladesh: empirical evidence from nationally representative household panel data. Food security. 2018;10:701-20.	Title and abstract not relevant- excluded
167.	Islam Anne F, Akter SM, Sheikh SP, Ireen S, Escobar-DeMarco J, Kappos K, et al. Quality of nutrition services in primary health care facilities of Dhaka city: State of nutrition mainstreaming in urban Bangladesh. PLoS One. 2022;17(12):e0278621.	Title and abstract not relevant- excluded
168.	Islam F, Das Trisha A, Hafsa JM, Hasan A, Degen GH, Ali N. Occurrence of aflatoxin M1 in human breast milk in Bangladesh. Mycotoxin Research. 2021;37(3):241-8.	Title and abstract not relevant- excluded
169.	Islam MA, Nielsen C. Maternal and child health services: evaluating mothers' perceptions and participation. Public Health. 1993;107(4):243-9.	Title and abstract not relevant- excluded

SL	Articles excluded during title and abstract screening	Decision
170.	Islam MM, Brown KH. Zinc transferred through breast milk does not differ between appropriate-and small-for-gestational-age, predominantly breast-fed Bangladeshi infants. The Journal of nutrition. 2014;144(5):771-6.	Title and abstract not relevant- excluded
171.	Islam MM, McDonald CM, Krebs NF, Westcott J, Rahman AE, El Arifeen S, et al. Study protocol for a randomized, double-blind, community-based efficacy trial of various doses of zinc in micronutrient powders or tablets in young Bangladeshi children. Nutrients. 2018;10(2):132.	Title and abstract not relevant- excluded
172.	Islam MS, Mahmud ZH, Gope PS, Zaman RU, Hossain Z, Islam MS, et al. Hygiene intervention reduces contamination of weaning food in Bangladesh. Tropical medicine & international health. 2013;18(3):250-8.	Title and abstract not relevant- excluded
173.	Islam MS, Matsumoto M. Immunohistochemical localization of VEGFR-2 in mouse mammary gland during reproductive cycle. Journal of Advanced Veterinary and Animal Research. 2021;8(4):581.	Title and abstract not relevant- excluded
174.	Islam MS, Zafar Ullah AN, Mainali S, Imam MA, Hasan MI. Determinants of stunting during the first 1,000 days of life in Bangladesh: A review. Food Science & Nutrition. 2020;8(9):4685-95.	Title and abstract not relevant- excluded
175.	Islam MZ, Akhtaruzzaman M, Lamberg-Allardt C. Nutritional status of women in Bangladesh: comparison of energy intake and nutritional status of a low income rural group with a high income urban group. Asia Pacific journal of clinical nutrition. 2004;13(1).	Title and abstract not relevant- excluded
176.	Islam MZ, Shamim AA, Viljakainen HT, Akhtaruzzaman M, Jehan AH, Khan HU, et al. Effect of vitamin D, calcium and multiple micronutrient supplementation on vitamin D and bone status in Bangladeshi premenopausal garment factory workers with hypovitaminosis D: a double-blinded, randomised, placebo-controlled 1-year intervention. British journal of nutrition. 2010;104(2):241-7.	Title and abstract not relevant- excluded
177.	Islam S, Khan H, Khan H. Factors affecting fertility in a rural area of Bangladesh. The Bangladesh Journal of Scientific Research. 1993;11(1):21-6.	Title and abstract not relevant- excluded
178.	Islam SN, Ahmed L, Khan MNI, Huque S, Begum A, Yunus ABM. Immune components (IgA, IgM, IgG, immune cells) of colostrum of Bangladeshi mothers. Pediatrics international. 2006;48(6):543-8.	Title and abstract not relevant- excluded
179.	Islam T, Saha D, Bhowmik S, Nordin N, Islam S, Nur A-AU, et al. Nutritional properties of wild and fattening mud crab (Scylla serrata) in the south-eastern district of Bangladesh. Heliyon. 2022;8(6).	Title and abstract not relevant- excluded
180.	J. Limaye R, Ballard Sara A, Ahmed N, Ohkbuo S, Deka S, Mickish Gross C, et al. Enhancing the knowledge and behaviors of fieldworkers to promote family planning and maternal, newborn, and child health in Bangladesh through a digital health training package: results from a pilot study. International quarterly of community health education. 2020;40(2):143-9.	Title and abstract not relevant- excluded
181.	Jacobs B, Roberts E. Baseline assessment for addressing acute malnutrition by public-health staff in Cambodia. Journal of Health, Population and Nutrition. 2004:212-9.	Title and abstract not relevant- excluded
182.	Jahan N, Minuti A, Trevisi E. Assessment of immune response in periparturient dairy cows using ex vivo whole blood stimulation assay with lipopolysaccharides and carrageenan skin test. Veterinary Immunology and Immunopathology. 2015;165(3-4):119-26.	Title and abstract not relevant- excluded
183.	Jannat K, Luby SP, Unicomb L, Rahman M, Winch PJ, Hossain MI, et al. Snack food consumption among Bangladeshi children, supplementary data from a large RCT. Maternal & child nutrition. 2020;16(4):e12994.	Title and abstract not relevant- excluded

SL	Articles excluded during title and abstract screening	Decision
184.	Jiang NM, Tofail F, Moonah SN, Scharf RJ, Taniuchi M, Ma JZ, et al. Febrile illness and pro-inflammatory cytokines are associated with lower neurodevelopmental scores in Bangladeshi infants living in poverty. BMC pediatrics. 2014;14:1-9.	Title and abstract not relevant- excluded
185.	Jolly K, Ingram L, Khan KS, Deeks JJ, Freemantle N, MacArthur C. Systematic review of peer support for breastfeeding continuation: metaregression analysis of the effect of setting, intensity, and timing. Bmj. 2012;344.	Title and abstract not relevant- excluded
186.	Jukic AMZ, Zuchniak A, Qamar H, Ahmed T, Mahmud AA, Roth DE. Vitamin d treatment during pregnancy and maternal and neonatal cord blood metal concentrations at delivery: Results of a randomized controlled trial in bangladesh. Environmental health perspectives. 2020;128(11):117007.	Title and abstract not relevant- excluded
187.	Kabir A, Maitrot MRL. Factors influencing feeding practices of extreme poor infants and young children in families of working mothers in Dhaka slums: A qualitative study. PloS one. 2017;12(2):e0172119.	Title and abstract not relevant- excluded
188.	Kabir A, Rahman MJ, Shamim AA, Klemm RD, Labrique AB, Rashid M, et al. Identifying maternal and infant factors associated with newborn size in rural Bangladesh by partial least squares (PLS) regression analysis. PLoS one. 2017;12(12):e0189677.	Title and abstract not relevant- excluded
189.	Kabir ME, Miraz FH, Alam MH, Sarker MB, Hashem MA, Khandoker MY, et al. Dietary energy influences ovarian morphology and in vitro maturation of oocytes in goats. Journal of Applied Animal Research. 2022;50(1):47-53.	Title and abstract not relevant- excluded
190.	Kabir MR. How do traditional media access and mobile phone use affect maternal healthcare service use in Bangladesh? Moderated mediation effects of socioeconomic factors. PLoS One. 2022;17(4):e0266631.	Title and abstract not relevant- excluded
191.	Kalam MA, Asif CA, Stormer A, Bishop T, Jackson-deGraffenried M, Talukder A. Use of designing for behaviour change framework in identifying and addressing barriers to and enablers of animal source feeding to children ages 8–23 months in Bandarban Hill District in Bangladesh: Implications for a nutrition-sensitive agriculture programme. Maternal & Child Nutrition. 2023;19(2):e13472.	Title and abstract not relevant- excluded
192.	Kang Y, Hurley KM, Ruel-Bergeron J, Monclus AB, Oemcke R, Wu LSF, et al. Household food insecurity is associated with low dietary diversity among pregnant and lactating women in rural Malawi. Public health nutrition. 2019;22(4):697-705.	Title and abstract not relevant- excluded
193.	Kar BK. Multi-stakeholder partnership in nutrition: an experience from Bangladesh. Indian Journal of Community Health. 2014;26(Supp 1):15-21.	Title and abstract not relevant- excluded
194.	Karim KMR, Tasnim T. Impact of lockdown due to COVID-19 on nutrition and food security of the selected low-income households in Bangladesh. Heliyon. 2022;8(5).	Title and abstract not relevant- excluded
195.	Karlsson O, Kim R, Bogin B, SV S. Maternal height-standardized prevalence of stunting in 67 low-and middle-income countries. Journal of Epidemiology. 2022;32(7):337-44.	Title and abstract not relevant- excluded
196.	Kenea D, Jisha H. Urban-rural disparity and determinants of delivery care utilization in Oromia region, Ethiopia: Community-based cross-sectional study. International Journal of Nursing Practice. 2017;23(1).	Title and abstract not relevant- excluded
197.	Khan JR, Faisal ASM, Das S, Awan N. Identifying overnutrition risk groups in Bangladeshi married women based on sociodemographic factors: A classification and regression tree model. Obesity Medicine. 2022;33:100425.	Title and abstract not relevant- excluded

SL	Articles excluded during title and abstract screening	Decision
198.	Khan JR, Gulshan J. Heterogeneous effects of factors on child nutritional status in Bangladesh using linear quantile mixed model. Biostatistics & Epidemiology. 2020;4(1):265-81.	Title and abstract not relevant- excluded
199.	Khan NA, Khisa SK. Sustainable land management with rubber-based agroforestry: a Bangladeshi example of uplands community development 1. Sustainable Development. 2000;8(1):1-10.	Title and abstract not relevant- excluded
200.	Khan R, MacQuarrie KL, Sultana M, Nahar Q. Intermittent needs for family planning among women with an internal migrant husband in Bangladesh: a qualitative study. Sexual and Reproductive Health Matters. 2022;29(2):2097044.	Title and abstract not relevant- excluded
201.	Khanam R, Fleischer TC, Boghossian NS, Nisar I, Dhingra U, Rahman S, et al. Performance of a validated spontaneous preterm delivery predictor in South Asian and Sub-Saharan African women: a nested case control study. The Journal of Maternal-Fetal & Neonatal Medicine. 2022;35(25):8878-86.	Title and abstract not relevant- excluded
202.	Klemm RD, Merrill RD, Wu L, Shamim AA, Ali H, Labrique A, et al. Low-birthweight rates higher among B angladeshi neonates measured during active birth surveillance compared to national survey data. Maternal & child nutrition. 2015;11(4):583-94.	Title and abstract not relevant- excluded
203.	Kocarnik JM, Compton K, Dean FE, Fu W, Gaw BL, Harvey JD, et al. Cancer incidence, mortality, years of life lost, years lived with disability, and disability-adjusted life years for 29 cancer groups from 2010 to 2019: a systematic analysis for the global burden of disease study 2019. JAMA oncology. 2022;8(3):420-44.	Title and abstract not relevant- excluded
204.	Korpe PS, Haque R, Gilchrist C, Valencia C, Niu F, Lu M, et al. Natural history of cryptosporidiosis in a longitudinal study of slum-dwelling Bangladeshi children: association with severe malnutrition. PLoS neglected tropical diseases. 2016;10(5):e0004564.	Title and abstract not relevant- excluded
205.	Kundu RN, Hossain MG, Haque MA, Biswas S, Huq MM, Pasa MK, et al. Factor associated with anthropometric failure among under-five Bengali children: A comparative study between Bangladesh and India. Plos one. 2022;17(8):e0272634.	Title and abstract not relevant- excluded
206.	Kundu S, Azene AG, Kundu S, Banna MHA, Mahbub T, Alshahrani NZ, et al. Prevalence of and factors associated with early initiation of breastfeeding in Bangladesh: a multilevel modelling. International Health. 2023;15(4):403-13.	Title and abstract not relevant- excluded
207.	Kundu S, Jharna DE, Banna MHA, Khan MSI. Factors associated with dietary diversity and physical activity of pregnant women in Bangladesh: a cross-sectional study at an antenatal care setting. Lifestyle Medicine. 2021;2(3):e41.	Title and abstract not relevant- excluded
208.	Kunjumen T, Okech M, Deki, Asamani JA, Mohamed N, Nuruzzaman M. Multi-country case studies on planning RMNCH services using WISN methodology: Bangladesh, Ghana, Kenya, Sultanate of Oman and Papua New Guinea. Human Resources for Health. 2022;19(Suppl 1):155.	Title and abstract not relevant- excluded
209.	Kurzawa Z, Cotton CS, Mazurkewich N, Verney A, Busch-Hallen J, Kashi B. Training healthcare workers increases IFA use and adherence: Evidence and cost-effectiveness analysis from Bangladesh. Maternal & child nutrition. 2021;17(2):e13124.	Title and abstract not relevant- excluded
210.	L Sloan N, Ahmed S, Islam M, N Mitra S. Experiences with community kangaroo mother care in very low-income settings. Current Women's Health Reviews. 2011;7(3):310-6.	Title and abstract not relevant- excluded

SL	Articles excluded during title and abstract screening	Decision
211.	Lakhanpaul M, Benton L, Lloyd-Houldey O, Manikam L, Rosenthal DM, Allaham S, et al. Nurture Early for Optimal Nutrition (NEON) programme: qualitative study of drivers of infant feeding and care practices in a British-Bangladeshi population. BMJ open. 2020;10(6):e035347.	Title and abstract not relevant- excluded
212.	Lassi ZS, Kedzior SG, Bhutta ZA. Community-based maternal and newborn educational care packages for improving neonatal health and survival in low-and middle-income countries. Cochrane Database of Systematic Reviews. 2019(11).	Title and abstract not relevant- excluded
213.	Lazzerini M, Rubert L, Pani P. Specially formulated foods for treating children with moderate acute malnutrition in low-and middle-income countries. Cochrane Database of Systematic Reviews. 2013(6).	Title and abstract not relevant- excluded
214.	Lee AC, Katz J, Blencowe H, Cousens S, Kozuki N, Vogel JP, et al. National and regional estimates of term and preterm babies born small for gestational age in 138 low-income and middle-income countries in 2010. The Lancet global health. 2013;1(1):e26-e36.	Title and abstract not relevant- excluded
215.	Lee AC, Kozuki N, Cousens S, Stevens GA, Blencowe H, Silveira MF, et al. Estimates of burden and consequences of infants born small for gestational age in low and middle income countries with INTERGROWTH-21st standard: analysis of CHERG datasets. bmj. 2017;358.	Title and abstract not relevant- excluded
216.	Lee B, Carmolli M, Dickson DM, Colgate ER, Diehl SA, Uddin MI, et al. Rotavirus-specific immunoglobulin A responses are impaired and serve as a suboptimal correlate of protection among infants in Bangladesh. Clinical Infectious Diseases. 2018;67(2):186-92.	Title and abstract not relevant- excluded
217.	Lenguerrand E, Harding S. P46 Ethnic differences in pace of growth between birth and 5 years: results from the millennium cohort study. Journal of Epidemiology & Community Health. 2010;64(Suppl 1):A51-A.	Title and abstract not relevant- excluded
218.	Leong C, Gibson RS, Diana A, Haszard JJ, Rahmannia S, Ansari MB, et al. Differences in micronutrient intakes of exclusive and partially breastfed Indonesian infants from resource-poor households are not accompanied by differences in micronutrient status, morbidity, or growth. The Journal of Nutrition. 2021;151(3):705-15.	Title and abstract not relevant- excluded
219.	Li H, Kim Y, Park C, Kang M, Kang Y. Gender-common and gender-specific determinants of child dietary diversity in eight Asia Pacific countries. Journal of global health. 2022;12.	Title and abstract not relevant- excluded
220.	Lin A, Ali S, Arnold BF, Rahman MZ, Alauddin M, Grembi J, et al. Effects of water, sanitation, handwashing, and nutritional interventions on environmental enteric dysfunction in young children: a cluster-randomized, controlled trial in rural Bangladesh. Clinical Infectious Diseases. 2020;70(5):738-47.	Title and abstract not relevant- excluded
221.	Lin A, Arnold BF, Mertens AN, Lin J, Benjamin-Chung J, Ali S, et al. Effects of water, sanitation, handwashing, and nutritional interventions on telomere length among children in a cluster-randomized controlled trial in rural Bangladesh. Elife. 2017;6:e29365.	Title and abstract not relevant- excluded
222.	Lindholm L, Streatfield PK, Ahmed S, Persson LÅ, Shaheen R. Cost-effectiveness of invitation to food supplementation early in pregnancy combined with multiple micronutrients on infant survival: analysis of data from MINIMat randomized trial, Bangladesh. 2015.	Title and abstract not relevant- excluded

SL	Articles excluded during title and abstract screening	Decision
223.	Liu G, Segrè J, Gülmezoglu AM, Mathai M, Smith JM, Hermida J, et al. Antenatal corticosteroids for management of preterm birth: a multi-country analysis of health system bottlenecks and potential solutions. BMC pregnancy and childbirth. 2015;15:1-16.	Title and abstract not relevant- excluded
224.	Liu JZ, Deng W, Lee J, Lin P-iD, Valeri L, Christiani DC, et al. A cross-validated ensemble approach to robust hypothesis testing of continuous nonlinear interactions: application to nutrition-environment studies. Journal of the American Statistical Association. 2022;117(538):561-73.	Title and abstract not relevant- excluded
225.	Luby SP, Rahman M, Arnold BF, Unicomb L, Ashraf S, Winch PJ, et al. Effects of water quality, sanitation, handwashing, and nutritional interventions on diarrhoea and child growth in rural Bangladesh: a cluster randomised controlled trial. The Lancet Global Health. 2018;6(3):e302-e15.	Title and abstract not relevant- excluded
226.	Mahmud MZ, Islam MT, Misran N, Almutairi AF, Cho M. Ultra-wideband (UWB) antenna sensor based microwave breast imaging: A review. Sensors. 2018;18(9):2951.	Title and abstract not relevant- excluded
227.	Mahmud R. Mixed implications of private supplementary tutoring for students' learning: Urban and rural disparities in Bangladesh. International Journal of Comparative Education and Development. 2019;21(1):61-75.	Title and abstract not relevant- excluded
228.	Malin Igra A, Vahter M, Raqib R, Kippler M. Early-life cadmium exposure and bone-related biomarkers: a longitudinal study in children. Environmental Health Perspectives. 2019;127(3):037003.	Title and abstract not relevant- excluded
229.	Mangwi Ayiasi R, Kolsteren P, Batwala V, Criel B, Orach CG. Effect of village health team home visits and mobile phone consultations on maternal and newborn care practices in Masindi and Kiryandongo, Uganda: a community-intervention trial. PloS one. 2016;11(4):e0153051.	Title and abstract not relevant- excluded
230.	Mannan I, Rahman SM, Sania A, Seraji HR, Arifeen S, Winch P, et al. Can early postpartum home visits by trained community health workers improve breastfeeding of newborns? Journal of Perinatology. 2008;28(9):632-40.	Title and abstract not relevant- excluded
231.	Maternal Af, Group NHIGAS, Maternal Af, Group NHIGS. Simplified models to assess newborn gestational age in low-middle income countries: findings from a multicountry, prospective cohort study. BMJ Global Health. 2021;6(9):e005688.	Title and abstract not relevant- excluded
232.	MAUNG U K, KHIN M, WAI NN, HMAN NW, MYINT TT, BUTLER T. Risk factors for the development of persistent diarrhoea and malnutrition in Burmese children. International Journal of Epidemiology. 1992;21(5):1021-9.	Title and abstract not relevant- excluded
233.	Mazumder S, Taneja S, Bahl R, Mohan P, Strand TA, Sommerfelt H, et al. Effect of implementation of integrated management of neonatal and childhood illness programme on treatment seeking practices for morbidities in infants: cluster randomised trial. BMJ. 2014;349.	Title and abstract not relevant- excluded
234.	McFadden A, Atkin K, Renfrew MJ. The impact of transnational migration on intergenerational transmission of knowledge and practice related to breast feeding. Midwifery. 2014;30(4):439-46.	Title and abstract not relevant- excluded
235.	McFadden A, Renfrew MJ, Atkin K. Does cultural context make a difference to women's experiences of maternity care? A qualitative study comparing the perspectives of breast-feeding women of Bangladeshi origin and health practitioners. Health Expectations. 2013;16(4):e124-e35.	Title and abstract not relevant- excluded

SL	Articles excluded during title and abstract screening	Decision
236.	McKerricher L, Petrucka P. Maternal nutritional supplement delivery in developing countries: a scoping review. BMC nutrition. 2019;5:1-6.	Title and abstract not relevant- excluded
237.	Mehrin SF, Hasan MI, Tofail F, Shiraji S, Ridout D, Grantham-McGregor S, et al. Integrating a group-based, early childhood parenting intervention into primary health care services in rural Bangladesh: a cluster-randomized controlled trial. Frontiers in Pediatrics. 2022;10:886542.	Title and abstract not relevant- excluded
238.	Mekonnen GK, Mengistie B, Sahilu G, Mulat W, Kloos H. Caregivers' knowledge and attitudes about childhood diarrhea among refugee and host communities in Gambella Region, Ethiopia. Journal of Health, Population and Nutrition. 2018;37:1-11.	Title and abstract not relevant- excluded
239.	Mekonnen TC, Workie SB, Yimer TM, Mersha WF. Meal frequency and dietary diversity feeding practices among children 6–23 months of age in Wolaita Sodo town, Southern Ethiopia. Journal of Health, Population and Nutrition. 2017;36:1-8.	Title and abstract not relevant- excluded
240.	Mhajabin S, Hossain AT, Nusrat N, Jabeen S, Ameen S, Banik G, et al. Indirect effects of the early phase of the COVID-19 pandemic on the coverage of essential maternal and newborn health services in a rural subdistrict in Bangladesh: results from a cross-sectional household survey. BMJ open. 2022;12(2):e056951.	Title and abstract not relevant- excluded
241.	Mihrshahi S, Ara G, Khanam M, Rasheed S, Agho KE, Kabir AI, et al. The Shishu Pushti Trial–Extended Peer Counseling for Improving Feeding Practices and Reducing Undernutrition in Children Aged 0-48 Months in Urban Bangladesh: Protocol for a Cluster-Randomized Controlled Trial. JMIR Research Protocols. 2022;11(2):e31475.	Title and abstract not relevant- excluded
242.	Milner KM, Bhopal S, Black M, Dua T, Gladstone M, Hamadani J, et al. Counting outcomes, coverage and quality for early child development programmes. Archives of Disease in Childhood. 2019;104(Suppl 1):S13-S21.	Title and abstract not relevant- excluded
243.	Milner KM, Salazar RB, Bhopal S, Brentani A, Britto PR, Dua T, et al. Contextual design choices and partnerships for scaling early child development programmes. Archives of disease in childhood. 2019;104(Suppl 1):S3-S12.	Title and abstract not relevant- excluded
244.	Mon MR, Parvin MN, Kemp J, Joya SS, Pedersen C, Byrskog U, et al. Midwives' associations' dual role in supporting members and driving the profession forward: A qualitative interview study from Bangladesh. Journal of Asian Midwives (JAM). 2022;9(1):15-33.	Title and abstract not relevant- excluded
245.	Monangi N, Xu H, Khanam R, Khan W, Deb S, Pervin J, et al. Association of maternal prenatal selenium concentration and preterm birth: a multicountry meta-analysis. BMJ global health. 2021;6(9):e005856.	Title and abstract not relevant- excluded
246.	Mondal SI, Akter A, Koga R, Hosokawa T, Dayi M, Murase K, et al. Reduced genome of the gut symbiotic bacterium "Candidatus Benitsuchiphilus tojoi" provides insight into its possible roles in ecology and adaptation of the host insect. Frontiers in Microbiology. 2020;11:840.	Title and abstract not relevant- excluded
247.	Moran AC, Choudhury N, Uz Zaman Khan N, Ahsan Karar Z, Wahed T, Faiz Rashid S, et al. Newborn care practices among slum dwellers in Dhaka, Bangladesh: a quantitative and qualitative exploratory study. BMC pregnancy and childbirth. 2009;9:1-8.	Title and abstract not relevant- excluded

SL	Articles excluded during title and abstract screening	Decision
248.	Morris SK, Pell LG, Rahman MZ, Mahmud AA, Shi J, Ahmed T, et al. Effects of maternal vitamin D supplementation during pregnancy and lactation on infant acute respiratory infections: follow-up of a randomized trial in Bangladesh. Journal of the Pediatric Infectious Diseases Society. 2021;10(9):901-9.	Title and abstract not relevant- excluded
249.	Morseth MS, Grewal NK, Kaasa IS, Hatloy A, Barikmo I, Henjum S. Dietary diversity is related to socioeconomic status among adult Saharawi refugees living in Algeria. BMC public health. 2017;17:1-9.	Title and abstract not relevant- excluded
250.	Mostafa I, Islam SF, Mondal P, Faruque A, Ahmed T, Hossain MI. Factors affecting low coverage of the vitamin A supplementation program among young children admitted in an urban diarrheal treatment facility in Bangladesh. Global Health Action. 2019;12(1):1588513.	Title and abstract not relevant- excluded
251.	Mostafa I, Naila NN, Mahfuz M, Roy M, Faruque AS, Ahmed T. Children living in the slums of Bangladesh face risks from unsafe food and water and stunted growth is common. Acta Paediatrica. 2018;107(7):1230-9.	Title and abstract not relevant- excluded
252.	Müller-Hauser AA, Sobhan S, Huda TMN, Waid JL, Wendt AS, Islam MA, et al. Key food hygiene behaviors to reduce microbial contamination of complementary foods in rural Bangladesh. The American Journal of Tropical Medicine and Hygiene. 2022;107(3):709.	Title and abstract not relevant- excluded
253.	Munirul Islam M, Arafat Y, Connell N, Mothabbir G, McGrath M, Berkley JA, et al. Severe malnutrition in infants aged< 6 months—Outcomes and risk factors in Bangladesh: A prospective cohort study. Maternal & child nutrition. 2019;15(1):e12642.	Title and abstract not relevant- excluded
254.	Muraduzzaman A, Islam SR-U, Siddiqui MMR. Use of drugs and treatment cost in acute watery diarrhoea of under-2 children attending a tertiary hospital of Bogra. Journal of Medicine. 2013;14(2):149.	Title and abstract not relevant- excluded
255.	Murray-Kolb LE, Rasmussen ZA, Scharf RJ, Rasheed MA, Svensen E, Seidman JC, et al. The MAL-ED cohort study: methods and lessons learned when assessing early child development and caregiving mediators in infants and young children in 8 low-and middle-income countries. Clinical Infectious Diseases. 2014;59(suppl_4):S261-S72.	Title and abstract not relevant- excluded
256.	Nachtnebel M, O'Mahony A, Pillai N, Hort K. Effectively engaging the private sector through vouchers and contracting–A case for analysing health governance and context. Social Science & Medicine. 2015;145:193-200.	Title and abstract not relevant- excluded
257.	Nadia KN, Das A, Karmakar P, Banik S, Rahman KA, Hossain MM, et al. Exploring women's awareness about breastfeeding and health benefits using a cross-sectional survey in Dhaka City, Bangladesh. Int J Pharm Sci Res. 2016;7(6):2410-5.	Title and abstract not relevant- excluded
258.	Nahar B, Hossain I, Hamadani J, Ahmed T, Grantham-McGregor S, Persson LA. Effect of a food supplementation and psychosocial stimulation trial for severely malnourished children on the level of maternal depressive symptoms in B angladesh. Child: Care, Health and Development. 2015;41(3):483-93.	Title and abstract not relevant- excluded
259.	Naila N, Nahar B, Lazarus M, Ritter G, Hossain M, Mahfuz M, et al. "Those who care much, understand much." Maternal perceptions of children's appetite: Perspectives from urban and rural caregivers of diverse parenting experience in Bangladesh. Maternal & child nutrition. 2018;14(1):e12473.	Title and abstract not relevant- excluded

SL	Articles excluded during title and abstract screening	Decision
260.	Nair H, Simões EA, Rudan I, Gessner BD, Azziz-Baumgartner E, Zhang JSF, et al. Global and regional burden of hospital admissions for severe acute lower respiratory infections in young children in 2010: a systematic analysis. The Lancet. 2013;381(9875):1380-90.	Title and abstract not relevant- excluded
261.	Namayengo FM, Antonides G, Cecchi F. Microcredit and food security: Evidence from rural households in Uganda. Journal of African Economies. 2018;27(4):457-82.	Title and abstract not relevant- excluded
262.	Naz S, Page A, Agho KE. Household air pollution from use of cooking fuel and under-five mortality: The role of breastfeeding status and kitchen location in Pakistan. PLoS One. 2017;12(3):e0173256.	Title and abstract not relevant- excluded
263.	Nguyen PH, Martin-Prevel Y, Moursi M, Tran LM, Menon P, Ruel MT, et al. Assessing dietary diversity in pregnant women: relative validity of the list-based and open recall methods. Current Developments in Nutrition. 2020;4(1):nzz134.	Title and abstract not relevant- excluded
264.	Nguyen PH, Sanghvi T, Kim SS, Tran LM, Afsana K, Mahmud Z, et al. Factors influencing maternal nutrition practices in a large scale maternal, newborn and child health program in Bangladesh. PloS one. 2017;12(7):e0179873.	Title and abstract not relevant- excluded
265.	Noor SZ, Rousham EK. Breast-feeding and maternal mental well-being among Bangladeshi and Pakistani women in north-east England. Public health nutrition. 2008;11(5):486-92.	Title and abstract not relevant- excluded
266.	Nor B, Ahlberg BM, Doherty T, Zembe Y, Jackson D, Ekström EC, et al. Mother's perceptions and experiences of infant feeding within a community-based peer counselling intervention in South Africa. Maternal & child nutrition. 2012;8(4):448-58.	Title and abstract not relevant- excluded
267.	Omer A, Hailu D, Whiting SJ. Effect of a child-owned poultry intervention providing eggs on nutrition status and motor skills of young children in southern Ethiopia: A cluster randomized and controlled community trial. International Journal of Environmental Research and Public Health. 2022;19(22):15305.	Title and abstract not relevant- excluded
268.	Osendarp SJ, Van Raaij JM, Arifeen SE, Wahed M, Baqui AH, Fuchs GJ. A randomized, placebo-controlled trial of the effect of zinc supplementation during pregnancy on pregnancy outcome in Bangladeshi urban poor. The American journal of clinical nutrition. 2000;71(1):114-9.	Title and abstract not relevant- excluded
269.	Padhani ZA, Moazzam Z, Ashraf A, Bilal H, Salam RA, Das JK, et al. Vitamin C supplementation for prevention and treatment of pneumonia. Cochrane Database of Systematic Reviews. 2020(4).	Title and abstract not relevant- excluded
270.	Palis FG, Malabayabas AJ, Singleton GR, Mazid MA, Johnson DE. Early harvest of monsoon rice to address seasonal hunger in northwest Bangladesh. Food Security. 2016;8:443-57.	Title and abstract not relevant- excluded
271.	Palwala M, Sharma S, Udipi SA, Ghugre PS, Kothari G, Sawardekar P. Nutritional quality of diets fed to young children in urban slums can be improved by intensive nutrition education. Food and Nutrition bulletin. 2009;30(4):317-26.	Title and abstract not relevant- excluded
272.	Pandey S, Lin Y, Collier-Tenison S, Bodden J. Social factors determining the experience of blindness among pregnant women in developing countries: the case of India. Health & social work. 2012;37(3):157-69.	Title and abstract not relevant- excluded
273.	Panter-Brick C. Seasonal growth patterns in rural Nepali children. Annals of human biology. 1997;24(1):1-18.	Title and abstract not relevant- excluded

SL	Articles excluded during title and abstract screening	Decision
274.	Park JJ, Fang ML, Harari O, Dron L, Siden EG, Majzoub R, et al. Association of early interventions with birth outcomes and child linear growth in low-income and middle-income countries: Bayesian network meta-analyses of randomized clinical trials. JAMA network open. 2019;2(7):e197871-e.	Title and abstract not relevant- excluded
275.	Parvez SM, Azad R, Rahman M, Unicomb L, Ram PK, Naser AM, et al. Achieving optimal technology and behavioral uptake of single and combined interventions of water, sanitation hygiene and nutrition, in an efficacy trial (WASH benefits) in rural Bangladesh. Trials. 2018;19:1-16.	Title and abstract not relevant- excluded
276.	Patel A, Badhoniya N, Khadse S, Senarath U, Agho KE, Dibley MJ, et al. Infant and young child feeding indicators and determinants of poor feeding practices in India: secondary data analysis of National Family Health Survey 2005–06. Food and nutrition bulletin. 2010;31(2):314-33.	Title and abstract not relevant- excluded
277.	Batal M, Boulghourjian C, Abdallah A, Afifi R. Breast-feeding and feeding practices of infants in a developing country: a national survey in Lebanon. Public health nutrition. 2006;9(3):313-9.	Title and abstract not relevant- excluded
278.	Bbaale E. Determinants of early initiation, exclusiveness, and duration of breastfeeding in Uganda. Journal of health, population, and nutrition. 2014;32(2):249.	Title and abstract not relevant- excluded
279.	Benedict RK, Craig HC, Torlesse H, Stoltzfus RJ. Trends and predictors of optimal breastfeeding among children 0–23 months, South Asia: Analysis of national survey data. Maternal & child nutrition. 2018;14:e12698.	Title and abstract not relevant- excluded
280.	Bhandari S, Thorne-Lyman AL, Shrestha B, Neupane S, Nonyane BAS, Manohar S, et al. Determinants of infant breastfeeding practices in Nepal: a national study. International breastfeeding journal. 2019;14:1-17.	Title and abstract not relevant- excluded
281.	Chandrashekhar T, Joshi H, Binu V, Shankar P, Rana M, Ramachandran U. Breast-feeding initiation and determinants of exclusive breast-feeding–a questionnaire survey in an urban population of western Nepal. Public health nutrition. 2007;10(2):192-7.	Title and abstract not relevant- excluded
282.	Chaudhary R, Shah T, Raja S. Knowledge and practice of mothers regarding breast feeding: a hospital based study. Health Renaissance. 2011;9(3):194-200.	Title and abstract not relevant- excluded
283.	Chipojola R, Lee GT, Chiu H-Y, Chang P-C, Kuo S-Y. Determinants of breastfeeding practices among mothers in Malawi: a population-based survey. International health. 2020;12(2):132-41.	Title and abstract not relevant- excluded
284.	Chye JK, Zain Z, Lim WL, Lim CT. Breastfeeding at 6 weeks and predictive factors. Journal of tropical pediatrics. 1997;43(5):287-92.	Title and abstract not relevant- excluded
285.	Dashti M, Scott JA, Edwards CA, Al-Sughayer M. Determinants of breastfeeding initiation among mothers in Kuwait. International breastfeeding journal. 2010;5:1-9.	Title and abstract not relevant- excluded
286.	Derso T, Biks GA, Tariku A, Tebeje NB, Gizaw Z, Muchie KF, et al. Correlates of early neonatal feeding practice in Dabat HDSS site, northwest Ethiopia. International breastfeeding journal. 2017;12:1-7.	Title and abstract not relevant- excluded
287.	Dharel D, Dhungana R, Basnet S, Gautam S, Dhungana A, Dudani R, et al. Breastfeeding practices within the first six months of age in mid-western and eastern regions of Nepal: a health facility-based cross-sectional study. BMC pregnancy and childbirth. 2020;20:1-9.	Title and abstract not relevant- excluded

SL	Articles excluded during title and abstract screening	Decision
288.	Rahman A, Hossain MM. Quantile regression approach to estimating prevalence and determinants of child malnutrition. Journal of Public Health. 2022:1-17.	Title and abstract not relevant- excluded
289.	Rahman M, Kabir M. Do adolescents support early marriage in Bangladesh? Evidence from study. Journal of the Nepal Medical Association. 2005;44(159).	Title and abstract not relevant- excluded
290.	Rahman MM. Micronutrient profile of children and women in rural Bangladesh: study on available data for iron and vitamin A supplementation. East African journal of public health. 2009;6(1).	Title and abstract not relevant- excluded
291.	Rahman MO, Yamaji N, Sasayama K, Yoneoka D, Ota E. Technology-based innovative healthcare solutions for improving maternal and child health outcomes in low-and middle-income countries: A network meta-analysis protocol. Nursing Open. 2023;10(1):367-76.	Title and abstract not relevant- excluded
292.	Rahman QS-u, Islam MS, Hossain B, Hossain T, Connor NE, Jaman MJ, et al. Centralized data management in a multicountry, multisite population-based study. The Pediatric infectious disease journal. 2016;35(5):S23-S8.	Title and abstract not relevant- excluded
293.	Rahman S, Rahman AS, Alam N, Ahmed AS, Ireen S, Chowdhury IA, et al. Vitamin A deficiency and determinants of vitamin A status in Bangladeshi children and women: findings of a national survey. Public health nutrition. 2017;20(6):1114-25.	Title and abstract not relevant- excluded
294.	Rai A, Khan MN, Thapa S. Trends and determinants of anaemia in women of Nepal: a multilevel analysis. Maternal & Child Nutrition. 2020;16(4):e13044.	Title and abstract not relevant- excluded
295.	Ramakrishnan U, Martorell R, Latham MC, Abel R. Dietary vitamin A intakes of preschool-age children in South India. The Journal of nutrition. 1999;129(11):2021-7.	Title and abstract not relevant- excluded
296.	Razzaque S, Elias SM, Haque T, Biswas S, Jewel GNA, Rahman S, et al. Gene expression analysis associated with salt stress in a reciprocally crossed rice population. Scientific reports. 2019;9(1):8249.	Title and abstract not relevant- excluded
297.	Rianon N, Britt R, Faisel A, Shahidullah S, LeBlanc A. Is poor nutrition masking the effects of depomedroxyprogesterone acetate on bones in adolescent users? Singapore Med J. 2010;51(11):876-82.	Title and abstract not relevant- excluded
298.	Rogawski McQuade ET, Shaheen F, Kabir F, Rizvi A, Platts-Mills JA, Aziz F, et al. Epidemiology of Shigella infections and diarrhea in the first two years of life using culture-independent diagnostics in 8 low-resource settings. PLoS neglected tropical diseases. 2020;14(8):e0008536.	Title and abstract not relevant- excluded
299.	Roni RA, Sani MNH, Munira S, Wazed MA, Siddiquee S. Nutritional composition and sensory evaluation of cake fortified with moringa oleifera leaf powder and ripe banana flour. Applied Sciences. 2021;11(18):8474.	Title and abstract not relevant- excluded
300.	Ronsmans C, Fisher DJ, Osmond C, Margetts BM, Fall CH. Multiple micronutrient supplementation during pregnancy in low-income countries: a meta-analysis of effects on stillbirths and on early and late neonatal mortality. Food and nutrition bulletin. 2009;30(4_suppl4):S547-S55.	Title and abstract not relevant- excluded
301.	Rosetta L, Kurpad A, Mascie-Taylor C, Shetty P. Total energy expenditure (H218O), physical activity level and milk output of lactating rural Bangladeshi tea workers and nontea workers. European journal of clinical nutrition. 2005;59(5):632-8.	Title and abstract not relevant- excluded
302.	Rosetta L, Mascie-Taylor C. Factors in the regulation of fertility in deprived populations. Annals of Human Biology. 2009;36(5):642-52.	Title and abstract not relevant- excluded

SL	Articles excluded during title and abstract screening	Decision
303.	Ross L, Simkhada P, Smith WCS. Evaluating effectiveness of complex interventions aimed at reducing maternal mortality in developing countries. Journal of Public Health. 2005;27(4):331-7.	Title and abstract not relevant- excluded
304.	Roth DE, Gernand AD, Morris SK, Pezzack B, Islam MM, Dimitris MC, et al. Maternal vitamin D supplementation during pregnancy and lactation to promote infant growth in Dhaka, Bangladesh (MDIG trial): study protocol for a randomized controlled trial. Trials. 2015;16:1-16.	Title and abstract not relevant- excluded
305.	Routh S. An economic appraisal of alternative strategies for the delivery of MCH–FP services in urban Dhaka, Bangladesh. The International Journal of Health Planning and Management. 2000;15(2):115-32.	Title and abstract not relevant- excluded
306.	Roy A, Hossain MM, Ullah MB, Mridha MK. Maternal and neonatal peripartum factors associated with late initiation of breast feeding in Bangladesh: a secondary analysis. BMJ open. 2022;12(5):e051004.	Title and abstract not relevant- excluded
307.	Roy D, Zulfiqar F, Tsusaka TW, Datta A. Household food insecurity and dietary diversity of women of reproductive age among smallholder farming households in northwest Bangladesh. Ecology of Food and Nutrition. 2022;61(4):460-83.	Title and abstract not relevant- excluded
308.	Roy S, Islam A, Molla A, Akramuzzaman S, Jahan F, Fuchs G. Impact of a single megadose of vitamin A at delivery on breastmilk of mothers and morbidity of their infants. European Journal of Clinical Nutrition. 1997;51(5):302-7.	Title and abstract not relevant- excluded
309.	Roy SK, de Groot S, Shafique S, Afroz A. Perceptions of mothers and use of breastmilk substitutes in Dhaka, Bangladesh. Journal of Health, Population and Nutrition. 2002:264-70.	Title and abstract not relevant- excluded
310.	Rubayet S, Shahidullah M, Hossain A, Corbett E, Moran AC, Mannan I, et al. Newborn survival in Bangladesh: a decade of change and future implications. Health Policy and Planning. 2012;27(suppl_3):iii40-iii56.	Title and abstract not relevant- excluded
311.	Rydbeck F, Bottai M, Tofail F, Persson L-Å, Kippler M. Urinary iodine concentrations of pregnant women in rural Bangladesh: a longitudinal study. Journal of exposure science & environmental epidemiology. 2014;24(5):504-9.	Title and abstract not relevant- excluded
312.	Saeedi R, Sultana A, Rahman K, Belal Bin Heyat M, Kamal MA, Ishawu M. Efficacy of Acacia nilotica Linn. Pod's Sitz Bath plus vaginal pessary in syndromic management of abnormal vaginal discharge: A randomized controlled trial. Evidence-Based Complementary and Alternative Medicine. 2022;2022(1):5769555.	Title and abstract not relevant- excluded
313.	Saha KK, Frongillo EA, Alam DS, Arifeen SE, Persson LÅ, Rasmussen KM. Appropriate infant feeding practices result in better growth of infants and young children in rural Bangladesh. The American journal of clinical nutrition. 2008;87(6):1852-9.	Title and abstract not relevant- excluded
314.	Saha KK, Tofail F, Frongillo E, Rasmussen K, Arifeen S, Persson L-Å, et al. Household food security is associated with early childhood language development: results from a longitudinal study in rural Bangladesh. Child: Care, health and development. 2010;36(3):309-16.	Title and abstract not relevant- excluded
315.	Saleem AF, Mahmud S, Baig-Ansari N, Zaidi AK. Impact of maternal education about complementary feeding on their infants' nutritional outcomes in low-and middle-income households: a community-based randomized interventional study in Karachi, Pakistan. Journal of Health, Population, and Nutrition. 2014;32(4):623.	Title and abstract not relevant- excluded

SL	Articles excluded during title and abstract screening	Decision
316.	Sand A, Kumar R, Shaikh BT, Somrongthong R, Hafeez A, Rai D. Determinants of severe acute malnutrition among children under five years in a rural remote setting: A hospital based study from district Tharparkar-Sindh, Pakistan. Pakistan journal of medical sciences. 2018;34(2):260.	Title and abstract not relevant- excluded
317.	Sanghvi T, Haque R, Roy S, Afsana K, Seidel R, Islam S, et al. Achieving behaviour change at scale: Alive & Thrive's infant and young child feeding programme in Bangladesh. Maternal & child nutrition. 2016;12:141-54.	Title and abstract not relevant- excluded
318.	Sanghvi T, Martin L, Hajeebhoy N, Abrha TH, Abebe Y, Haque R, et al. Strengthening systems to support mothers in infant and young child feeding at scale. Food and nutrition bulletin. 2013;34(3_suppl2):S156-S68.	Title and abstract not relevant- excluded
319.	Sanghvi T, Nguyen PH, Tharaney M, Ghosh S, Escobar-Alegria J, Mahmud Z, et al. Gaps in the implementation and uptake of maternal nutrition interventions in antenatal care services in Bangladesh, Burkina Faso, Ethiopia and India. Maternal & child nutrition. 2022;18(2):e13293.	Title and abstract not relevant- excluded
320.	Sanghvi TG, Homan R, Forissier T, Preware P, Kawu A, Nguyen TT, et al. The financial costs of mass media interventions used for improving breastfeeding practices in Bangladesh, Burkina Faso, Nigeria, and Vietnam. International Journal of Environmental Research and Public Health. 2022;19(24):16923.	Title and abstract not relevant- excluded
321.	Sanin KI, Islam MM, Mahfuz M, Ahmed AS, Mondal D, Haque R, et al. Micronutrient adequacy is poor, but not associated with stunting between 12-24 months of age: A cohort study findings from a slum area of Bangladesh. PloS one. 2018;13(3):e0195072.	Title and abstract not relevant- excluded
322.	Sarkar P, Rifat M, Bakshi P, Talukdar IH, Pechtl SM, Lindström Battle T, et al. How is parental education associated with infant and young child feeding in Bangladesh? a systematic literature review. BMC public health. 2023;23(1):510.	Title and abstract not relevant- excluded
323.	Sartorius B, VanderHeide JD, Yang M, Goosmann EA, Hon J, Haeuser E, et al. Subnational mapping of HIV incidence and mortality among individuals aged 15–49 years in sub-Saharan Africa, 2000–18: a modelling study. The Lancet HIV. 2021;8(6):e363-e75.	Title and abstract not relevant- excluded
324.	Sathar ZA, Siddiqui KA. Child Survival and Changing Fertility Patterns in Pakistan [with Comments]. The Pakistan Development Review. 1992;31(4):699-713.	Title and abstract not relevant- excluded
325.	Saville NM, Shrestha BP, Style S, Harris-Fry H, Beard BJ, Sengupta A, et al. Protocol of the Low Birth Weight South Asia Trial (LBWSAT), a cluster-randomised controlled trial testing impact on birth weight and infant nutrition of Participatory Learning and Action through women's groups, with and without unconditional transfers of fortified food or cash during pregnancy in Nepal. BMC pregnancy and childbirth. 2016;16:1-19.	Title and abstract not relevant- excluded
326.	Scherer E, Hagaman A, Chung E, Rahman A, O'Donnell K, Maselko J. The relationship between responsive caregiving and child outcomes: evidence from direct observations of mother-child dyads in Pakistan. BMC public health. 2019;19:1-10.	Title and abstract not relevant- excluded
327.	Schnefke CH, Lutter CK, Thuita F, Webale A, Flax VL, Bentley ME. Is it possible to promote egg consumption during pregnancy? Findings from a study on knowledge, perceptions, and practices in Kenya. Food and nutrition bulletin. 2019;40(2):151-70.	Title and abstract not relevant- excluded

SL	Articles excluded during title and abstract screening	Decision
328.	Seiermann AU, Al-Mufti H, Waid JL, Wendt AS, Sobhan S, Gabrysch S. Women's fasting habits and dietary diversity during Ramadan in rural Bangladesh. Maternal & child nutrition. 2021;17(3):e13135.	Title and abstract not relevant- excluded
329.	Senarath U, Dibley MJ, Godakandage SS, Jayawickrama H, Wickramasinghe A, Agho KE, et al. Determinants of infant and young child feeding practices in Sri Lanka: secondary data analysis of Demographic and Health Survey 2000. Food and nutrition bulletin. 2010;31(2):352-65.	Title and abstract not relevant- excluded
330.	Sethi V, Choedon T, Chowdhury R, Bhatia N, Dinachandra K, Murira Z, et al. Screening and management options for severe thinness during pregnancy in India. International Journal of Gynecology & Obstetrics. 2021;155(3):357-79.	Title and abstract not relevant- excluded
331.	Shah NM, Kazi SFA. Attitudes, contraceptive practice and children ever born among currently married women exposed to different types of communication media, Pakistan. The Pakistan Development Review. 1977;16(4):405-23.	Title and abstract not relevant- excluded
332.	Shah R, Mullany LC, Darmstadt GL, Mannan I, Rahman SM, Talukder RR, et al. Incidence and risk factors of preterm birth in a rural Bangladeshi cohort. BMC pediatrics. 2014;14:1-11.	Title and abstract not relevant- excluded
333.	Shaheen R, Streatfield PK, Naved RT, Lindholm L, Persson LÅ. Equity in adherence to and effect of prenatal food and micronutrient supplementation on child mortality: results from the MINIMat randomized trial, Bangladesh. BMC Public Health. 2014;14:1-9.	Title and abstract not relevant- excluded
334.	Shahjalal M, Chakma SK, Ahmed T, Yasmin I, Mahumud RA, Hossain A. Prevalence and determinants of using complementary and alternative medicine for the treatment of chronic illnesses: a multicenter study in Bangladesh. PLoS One. 2022;17(1):e0262221.	Title and abstract not relevant- excluded
335.	Shahrier MA, Wada H. Effects of ethanol exposure during lactation on ultrasonic vocalizations of rat pups upon their isolation: Increase in pup distress calls. Brain Sciences. 2021;11(9):1249.	Title and abstract not relevant- excluded
336.	Shaikh S, Schulze KJ, Kurpad A, Ali H, Shamim AA, Mehra S, et al. Development of bioelectrical impedance analysis-based equations for estimation of body composition in postpartum rural Bangladeshi women. British journal of nutrition. 2013;109(4):639-47.	Title and abstract not relevant- excluded
337.	Shamba D, Day LT, Zaman SB, Sunny AK, Tarimo MN, Peven K, et al. Barriers and enablers to routine register data collection for newborns and mothers: EN-BIRTH multi-country validation study. BMC pregnancy and childbirth. 2021;21:1-14.	Title and abstract not relevant- excluded
338.	Shamim AA, Hanif AA, Merrill RD, Campbell RK, Kumkum MA, Shaikh S, et al. Preferred delivery method and acceptability of wheat-soy blend (WSB++) as a daily complementary food supplement in Northwest Bangladesh. Ecology of food and nutrition. 2015;54(1):74-92.	Title and abstract not relevant- excluded
339.	Sharma G, Mathai M, Dickson KE, Weeks A, Hofmeyr GJ, Lavender T, et al. Quality care during labour and birth: a multi-country analysis of health system bottlenecks and potential solutions. BMC pregnancy and childbirth. 2015;15:1-19.	Title and abstract not relevant- excluded
340.	Shaun MMA, Nizum MWR, Shuvo MA, Fayeza F, Faruk MO, Alam MF, et al. Determinants of minimum dietary diversity of lactating mothers in rural northern region of Bangladesh: A community-based cross-sectional study. Heliyon. 2023;9(1).	Title and abstract not relevant- excluded

SL	Articles excluded during title and abstract screening	Decision
341.	Sheikh SP, Akter SM, Anne FI, Ireen S, Escobar-Alegria J, Kappos K, et al. Violations of International Code of Breast-milk Substitutes (BMS) in commercial settings and media in Bangladesh. Maternal & Child Nutrition. 2022;18:e13351.	Title and abstract not relevant- excluded
342.	Shih Y-H, Bryan MS, Parvez F, Uesugi KH, Shahriar M, Ahmed A, et al. Gravidity, parity, blood pressure and mortality among women in Bangladesh from the HEALS cohort. BMJ open. 2020;10(8):e037244.	Title and abstract not relevant- excluded
343.	Simen-Kapeu A, Seale AC, Wall S, Nyange C, Qazi SA, Moxon SG, et al. Treatment of neonatal infections: a multi-country analysis of health system bottlenecks and potential solutions. BMC pregnancy and childbirth. 2015;15:1-15.	Title and abstract not relevant- excluded
344.	Singha S, Koop G, Persson Y, Hossain D, Scanlon L, Derks M, et al. Incidence, etiology, and risk factors of clinical mastitis in dairy cows under semi-tropical circumstances in Chattogram, Bangladesh. Animals. 2021;11(8):2255.	Title and abstract not relevant- excluded
345.	Sinha B, Chowdhury R, Upadhyay RP, Taneja S, Martines J, Bahl R, et al. Integrated interventions delivered in health systems, home, and community have the highest impact on breastfeeding outcomes in low-and middle-income countries. The Journal of nutrition. 2017;147(11):2179S-87S.	Title and abstract not relevant- excluded
346.	Sinha S, Curtis K, Jayakody A, Viner R, Roberts H. Family and peer networks in intimate and sexual relationships amongst teenagers in a multicultural area of East London. Sociological Research Online. 2006;11(1):74-86.	Title and abstract not relevant- excluded
347.	Sinharoy SS, Waid JL, Haardörfer R, Wendt A, Gabrysch S, Yount KM. Women's dietary diversity in rural Bangladesh: Pathways through women's empowerment. Maternal & child nutrition. 2018;14(1):e12489.	Title and abstract not relevant- excluded
348.	Sitrin D, Guenther T, Waiswa P, Namutamba S, Namazzi G, Sharma S, et al. Improving newborn care practices through home visits: lessons from Malawi, Nepal, Bangladesh, and Uganda. Global health action. 2015;8(1):23963.	Title and abstract not relevant- excluded
349.	Skröder H, Kippler M, Tofail F, Vahter M. Early-life selenium status and cognitive function at 5 and 10 years of age in Bangladeshi children. Environmental Health Perspectives. 2017;125(11):117003.	Title and abstract not relevant- excluded
350.	Solmi M, Estradé A, Thompson T, Agorastos A, Radua J, Cortese S, et al. Physical and mental health impact of COVID-19 on children, adolescents, and their families: The Collaborative Outcomes study on Health and Functioning during Infection Times-Children and Adolescents (COH-FIT-C&A). Journal of affective disorders. 2022;299:367-76.	Title and abstract not relevant- excluded
351.	Somé JW, Jones AD. The influence of crop production and socioeconomic factors on seasonal household dietary diversity in Burkina Faso. PloS one. 2018;13(5):e0195685.	Title and abstract not relevant- excluded
352.	Sparling TM, Waid JL, Wendt AS, Gabrysch S. Depression among women of reproductive age in rural Bangladesh is linked to food security, diets and nutrition. Public health nutrition. 2020;23(4):660-73.	Title and abstract not relevant- excluded
353.	Stanaway JD, Afshin A, Gakidou E, Lim SS, Abate D, Abate KH, et al. Global, regional, and national comparative risk assessment of 84 behavioural, environmental and occupational, and metabolic risks or clusters of risks for 195 countries and territories, 1990–2017: a systematic analysis for the Global Burden of Disease Study 2017. The Lancet. 2018;392(10159):1923-94.	Title and abstract not relevant- excluded

SL	Articles excluded during title and abstract screening	Decision
354.	Stevens B, Watt K, Brimbecombe J, Clough A, Judd J, Lindsay D. The role of seasonality on the diet and household food security of pregnant women living in rural Bangladesh: a cross-sectional study. Public health nutrition. 2017;20(1):121-9.	Title and abstract not relevant- excluded
355.	Stevens B, Watt K, Brimbecombe J, Clough A, Judd JA, Lindsay D. A village-matched evaluation of providing a local supplemental food during pregnancy in rural Bangladesh: a preliminary study. BMC pregnancy and childbirth. 2018;18:1-11.	Title and abstract not relevant- excluded
356.	Strathdee SA, West BS, Reed E, Moazan B, Azim T, Dolan K. Substance use and HIV among female sex workers and female prisoners: risk environments and implications for prevention, treatment, and policies. JAIDS Journal of Acquired Immune Deficiency Syndromes. 2015;69:S110-S7.	Title and abstract not relevant- excluded
357.	Strong J, Lattof SR, Maliqi B, Yaqub N. Experiences of private sector quality care amongst mothers, newborns, and children in low-and middle-income countries: a systematic review. BMC health services research. 2021;21:1-15.	Title and abstract not relevant- excluded
358.	Sultanov M, Zeeuw Jd, Koot J, der Schans Jv, Beltman JJ, Fouw Md, et al. Investigating feasibility of 2021 WHO protocol for cervical cancer screening in underscreened populations: PREvention and SCReening Innovation Project Toward Elimination of Cervical Cancer (PRESCRIP-TEC). BMC public health. 2022;22(1):1356.	Title and abstract not relevant- excluded
359.	Sundaram ME, Ali H, Mehra S, Shamim AA, Ullah B, Rashid M, et al. Early newborn ritual foods correlate with delayed breastfeeding initiation in rural Bangladesh. International breastfeeding journal. 2016;11:1-6.	Title and abstract not relevant- excluded
360.	Sunuwar DR, Singh DR, Chaudhary NK, Pradhan PMS, Rai P, Tiwari K. Prevalence and factors associated with anemia among women of reproductive age in seven South and Southeast Asian countries: Evidence from nationally representative surveys. PloS one. 2020;15(8):e0236449.	Title and abstract not relevant- excluded
361.	Susilowati D. Hormonal images related nursing mothers breast cancer risk in Semarang. Bangladesh Journal of Medical Science. 2017;16(3):413.	Title and abstract not relevant- excluded
362.	Taghivand M, Pell LG, Rahman MZ, Mahmud AA, Ohuma EO, Pullangyeum EM, et al. Effect of maternal vitamin D supplementation on nasal pneumococcal acquisition, carriage dynamics and carriage density in infants in Dhaka, Bangladesh. BMC infectious diseases. 2022;22:1-11.	Title and abstract not relevant- excluded
363.	Talukder MQ-e-K, Shahidullah M, Chowdhury A, Khatoon S, Talukder K. Health professional associations and industry funding—reply from Talukder et al. The Lancet. 2017;389(10080):1696-7.	Title and abstract not relevant- excluded
364.	Thairu L, Pelto G. Newborn care practices in Pemba Island (Tanzania) and their implications for newborn health and survival. Maternal & child nutrition. 2008;4(3):194-208.	Title and abstract not relevant- excluded
365.	Thorne-Lyman AL, Valpiani N, Akter R, Baten MA, Genschick S, Karim M, et al. Fish and meat are often withheld from the diets of infants 6 to 12 months in fish-farming households in rural Bangladesh. Food and Nutrition Bulletin. 2017;38(3):354-68.	Title and abstract not relevant- excluded
366.	Tiranti D. The small miracle. New internationalist. 1979(79):19-21.	Title and abstract not relevant- excluded

SL	Articles excluded during title and abstract screening	Decision
367.	Titaley CR, Dibley MJ, Roberts CL, Hall J, Agho K. Iron and folic acid supplements and reduced early neonatal deaths in Indonesia. Bulletin of the World Health Organization. 2010;88(7):500-8.	Title and abstract not relevant- excluded
368.	Todd CS, Chowdhury Z, Mahmud Z, Islam N, Shabnam S, Parvin M, et al. Maternal nutrition intervention and maternal complications in 4 districts of Bangladesh: A nested cross-sectional study. PLoS Medicine. 2019;16(10):e1002927.	Title and abstract not relevant- excluded
369.	Tofail F, Hamadani J, Ahmed A, Mehrin F, Hakim M, Huda S. The mental development and behavior of low-birth-weight Bangladeshi infants from an urban low-income community. European journal of clinical nutrition. 2012;66(2):237-43.	Title and abstract not relevant- excluded
370.	Tran NT, Greer A, Kini B, Abdi H, Rajeh K, Cortier H, et al. Integrating sexual and reproductive health into health system strengthening in humanitarian settings: a planning workshop toolkit to transition from minimum to comprehensive services in the Democratic Republic of Congo, Bangladesh, and Yemen. Conflict and health. 2020;14:1-12.	Title and abstract not relevant- excluded
371.	Twamley K, Puthussery S, Harding S, Baron M, Macfarlane A. UK-born ethnic minority women and their experiences of feeding their newborn infant. Midwifery. 2011;27(5):595-602.	Title and abstract not relevant- excluded
372.	Uddin J, Hossain Z. Predictors of infant mortality in a developing country. Asian Journal of Epidemiology. 2008;1(1):1-16.	Title and abstract not relevant- excluded
373.	Uddin ME, George J, Jahan S, Shams Z, Haque N, Perry HB. Learnings from a pilot study to strengthen primary health care services: the community-clinic-centered health service model in Barishal District, Bangladesh. Global Health: Science and Practice. 2021;9(Supplement 1):S179-S89.	Title and abstract not relevant- excluded
374.	Uddin MF, Molyneux S, Muraya K, Jemutai J, Berkley JA, Walson JL, et al. Treatment-seeking and recovery among young undernourished children post-hospital discharge in Bangladesh: A qualitative study. Plos one. 2022;17(9):e0274996.	Title and abstract not relevant- excluded
375.	Uddin MSG, Islam MS, Methun MIH. Low birth weight baby and its associated factors among rural women in Bangladesh: a decision curve analysis. Bangladesh Medical Research Council Bulletin. 2021;47(1):42-9.	Title and abstract not relevant- excluded
376.	Uddin S, Mahmood H, Senarath U, Zahiruddin Q, Karn S, Rasheed S, et al. Analysis of stakeholders networks of infant and young child nutrition programmes in Sri Lanka, India, Nepal, Bangladesh and Pakistan. BMC public health. 2017;17:15-25.	Title and abstract not relevant- excluded
377.	Vahter M, Skröder H, Rahman SM, Levi M, Hamadani JD, Kippler M. Prenatal and childhood arsenic exposure through drinking water and food and cognitive abilities at 10 years of age: A prospective cohort study. Environment international. 2020;139:105723.	Title and abstract not relevant- excluded
378.	van Liere MJ, Tarlton D, Menon R, Yellamanda M, Reerink I. Harnessing private sector expertise to improve complementary feeding within a regulatory framework: Where is the evidence? Maternal & child nutrition. 2017;13:e12429.	Title and abstract not relevant- excluded
379.	Vinoy S, Rosetta L, Mascie-Taylor C. Repeated measurements of energy intake, energy expenditure and energy balance in lactating Bangladeshi mothers. European journal of clinical nutrition. 2000;54(7):579-85.	Title and abstract not relevant- excluded

SL	Articles excluded during title and abstract screening	Decision
380.	Vos T, Lim SS, Abbafati C, Abbas KM, Abbasi M, Abbasifard M, et al. Global burden of 369 diseases and injuries in 204 countries and territories, 1990–2019: a systematic analysis for the Global Burden of Disease Study 2019. The lancet. 2020;396(10258):1204-22.	Title and abstract not relevant- excluded
381.	Wable Grandner G, Dickin K, Kanbur R, Menon P, Rasmussen KM, Hoddinott J. Assessing statistical similarity in dietary intakes of women of reproductive age in Bangladesh. Maternal & Child Nutrition. 2021;17(2):e13086.	Title and abstract not relevant- excluded
382.	Walker CLF, Black RE. Zinc for the treatment of diarrhoea: effect on diarrhoea morbidity, mortality and incidence of future episodes. International journal of epidemiology. 2010;39(Suppl 1):i63.	Title and abstract not relevant- excluded
383.	Wang H, Abbas KM, Abbasifard M, Abbasi-Kangevari M, Abbastabar H, Abd-Allah F, et al. Global age-sex-specific fertility, mortality, healthy life expectancy (HALE), and population estimates in 204 countries and territories, 1950–2019: a comprehensive demographic analysis for the Global Burden of Disease Study 2019. The Lancet. 2020;396(10258):1160-203.	Title and abstract not relevant- excluded
384.	Waswa LM, Jordan I, Krawinkel MB, Keding GB. Seasonal variations in dietary diversity and nutrient intakes of women and their children (6–23 months) in Western Kenya. Frontiers in Nutrition. 2021;8:636872.	Title and abstract not relevant- excluded
385.	Weber AM, Rubio-Codina M, Walker SP, Van Buuren S, Eekhout I, Grantham-McGregor SM, et al. The D-score: a metric for interpreting the early development of infants and toddlers across global settings. BMJ global health. 2019;4(6):e001724.	Title and abstract not relevant- excluded
386.	Weingarten SE, Dearden KA, Crookston BT, Penny ME, Behrman JR, Humphries DL. Are Household Expenditures on Food Groups Associated with Children's Future Heights in Ethiopia, India, Peru, and Vietnam? International Journal of Environmental Research and Public Health. 2020;17(13):4739.	Title and abstract not relevant- excluded
387.	Weiss M, Somma D, Karim F, Abouihia A, Auer C, Kemp J, et al. Cultural epidemiology of TB with reference to gender in Bangladesh, India and Malawi [Special section on gender and TB]. The International Journal of Tuberculosis and Lung Disease. 2008;12(7):837-47.	Title and abstract not relevant- excluded
388.	Wendt AS, Sparling TM, Waid JL, Mueller AA, Gabrysch S. Food and Agricultural Approaches to Reducing Malnutrition (FAARM): protocol for a cluster-randomised controlled trial to evaluate the impact of a Homestead Food Production programme on undernutrition in rural Bangladesh. BMJ open. 2019;9(7):e031037.	Title and abstract not relevant- excluded
389.	West KP, Shamim AA, Mehra S, Labrique AB, Ali H, Shaikh S, et al. Effect of maternal multiple micronutrient vs iron–folic acid supplementation on infant mortality and adverse birth outcomes in rural Bangladesh: the JiVitA-3 randomized trial. Jama. 2014;312(24):2649-58.	Title and abstract not relevant- excluded
390.	Wiens KE, Lindstedt PA, Blacker BF, Johnson KB, Baumann MM, Schaeffer LE, et al. Mapping geographical inequalities in oral rehydration therapy coverage in low-income and middle-income countries, 2000–17. The Lancet Global Health. 2020;8(8):e1038-e60.	Title and abstract not relevant- excluded
391.	Wilson A, Gallos ID, Plana N, Lissauer D, Khan KS, Zamora J, et al. Effectiveness of strategies incorporating training and support of traditional birth attendants on perinatal and maternal mortality: meta-analysis. Bmj. 2011;343.	Title and abstract not relevant- excluded

SL	Articles excluded during title and abstract screening	Decision
392.	Winch PJ, Gilroy KE, Doumbia S, Patterson AE, Daou Z, Diawara A, et al. Operational issues and trends associated with the pilot introduction of zinc for childhood diarrhoea in Bougouni district, Mali. Journal of health, population, and nutrition. 2008;26(2):151.	Title and abstract not relevant- excluded
393.	Wondafrash M, Amsalu T, Woldie M. Feeding styles of caregivers of children 6-23 months of age in Derashe special district, Southern Ethiopia. BMC Public Health. 2012;12:1-8.	Title and abstract not relevant- excluded
394.	Wu Q, Zhang Y, Chang S, Wang W, Van Velthoven MH, Han H, et al. Monitoring and evaluating the adherence to a complementary food supplement (Ying Yang Bao) among young children in rural Qinghai, China: a mixed methods evaluation study. Journal of global health. 2017;7(1).	Title and abstract not relevant- excluded
395.	Yamasaki S, Tomihara T, Kimura G, Ueno Y, Ketema RM, Sato S, et al. Long-term effects of maternal resveratrol intake during lactation on cholesterol metabolism in male rat offspring. International journal of food sciences and nutrition. 2020;71(2):226-34.	Title and abstract not relevant- excluded
396.	Yimer NB, Liben ML. Effects of home delivery on colostrum avoidance practices in North Wollo zone, an urban setting, Ethiopia: a cross sectional study. Journal of Health, Population and Nutrition. 2018;37:1-7.	Title and abstract not relevant- excluded
397.	Yonzon KK, Dehingia N, Alwadhi V, Singh K, Kumar H, Bhat AA, et al. An Assessment of home-based newborn care plus innovation in six districts of Rajasthan: A cross sectional comparative analysis. Indian Journal of Community Health. 2019;31(3):338-46.	Title and abstract not relevant- excluded
398.	Yosef S, Jones AD, Chakraborty B, Gillespie S. Agriculture and nutrition in Bangladesh: Mapping evidence to pathways. Food and nutrition Bulletin. 2015;36(4):387-404.	Title and abstract not relevant- excluded
399.	Young MF, Nguyen P, Kachwaha S, Tran Mai L, Ghosh S, Agrawal R, et al. It takes a village: An empirical analysis of how husbands, mothers-in-law, health workers, and mothers influence breastfeeding practices in Uttar Pradesh, India. Maternal & child nutrition. 2020;16(2):e12892.	Title and abstract not relevant- excluded
400.	Zelka MA, Yalew AW, Debelew GT. The effects of completion of continuum of care in maternal health services on adverse birth outcomes in Northwestern Ethiopia: a prospective follow-up study. Reproductive Health. 2022;19(1):200.	Title and abstract not relevant- excluded
401.	A double-blind placebo-controlled trial of azithromycin to reduce mortality and improve growth in high-risk young children with non-bloody diarrhoea in low resource settings: the Antibiotics for Children with Diarrhoea (ABCD) trial protocol. Trials. 2020;21(1):71.	Title and abstract not relevant- excluded
402.	Abdullah AA, Rifat M, Hasan MT, Manir MZ, Khan MMM, Azad F. Infant and Young Child Feeding (IYCF) Practices, Household Food Security and Nutritional Status of Under-Five Children in Cox's Bazar, Bangladesh. Current research in nutrition and food science journal. 2018;6(3):789-97.	Title and abstract not relevant- excluded
403.	Aboud FE, Singla DR, Nahil MI, Borisova I. Effectiveness of a parenting program in Bangladesh to address early childhood health, growth and development. Social Science & Medicine. 2013;97:250-8.	Title and abstract not relevant- excluded
404.	Adams AM, Nababan HY, Hanifi SMA. Building social networks for maternal and newborn health in poor urban settlements: a cross-sectional study in Bangladesh. PLoS One. 2015;10(4):e0123817.	Title and abstract not relevant- excluded
405.	Afrose L, Banu B, Ahmed KR, Khanom K. Factors associated with knowledge about breastfeeding among female garment workers in Dhaka city. WHO South-East Asia Journal of Public Health. 2012;1(3):249-55.	Title and abstract not relevant- excluded

SL	Articles excluded during title and abstract screening	Decision
406.	Aguayo VM, Nair R, Badgaiyan N, Krishna V. Determinants of stunting and poor linear growth in children under 2 years of age in India: An in-depth analysis of Maharashtra's comprehensive nutrition survey. Maternal & child nutrition. 2016;12:121-40.	Title and abstract not relevant- excluded
407.	Ahmad R, Akhter QS, Haque M. Occupational cement dust exposure and inflammatory nemesis: Bangladesh relevance. Journal of Inflammation Research. 2021:2425-44.	Title and abstract not relevant- excluded
408.	Ahmad S, Abid J, Muhammad N, Wasila H, Zaitoun M, Awudi DA. Prevalence and factors associated with undernutrition among 6-59 months children in Tehsil Battagram, Pakistan. JPMA. 2022;72(1535).	Title and abstract not relevant- excluded
409.	Ahmed F. Vitamin A deficiency in Bangladesh: a review and recommendations for improvement. Public Health Nutrition. 1999;2(1):1-14.	Title and abstract not relevant- excluded
410.	Ahmed F, Mahmuda I, Sattar A, Akhtaruzzaman M. Anaemia and vitamin A deficiency in poor urban pregnant women of Bangladesh. Asia Pacific journal of clinical nutrition. 2003;12(4):460-6.	Title and abstract not relevant- excluded
411.	Ahmed JU, Gazi MA, Iqbal R, Islam QT, Talukder N. Value co-creation through social innovation in healthcare: a case of WE CARE Solar. World Journal of Entrepreneurship, Management and Sustainable Development. 2020;16(4):341-57.	Title and abstract not relevant- excluded
412.	Ahmed JU, Mozahid MN, Dhar AR, Alamgir MS, Jannat A, Islam MM. Food security and dietary diversity of tea workers of two tea gardens in greater Sylhet district of Bangladesh. GeoJournal. 2021;86:1015-27.	Title and abstract not relevant- excluded
413.	Ahmed L, Islam SN, Khan M, Huque S, Ahsan M. Antioxidant micronutrient profile (vitamin E, C, A, copper, zinc, iron) of colostrum: association with maternal characteristics. Journal of tropical pediatrics. 2004;50(6):357-8.	Title and abstract not relevant- excluded
414.	Ahmed S, Macfarlane A, Naylor J, Hastings J. Evaluating bilingual peer support for breastfeeding in a local sure start. British Journal of Midwifery. 2006;14(8):467-70.	Title and abstract not relevant- excluded
415.	Ahmed S, Mitra S, Chowdhury A, Camacho L, Winikoff B, Sloan N. Community Kangaroo Mother Care: implementation and potential for neonatal survival and health in very low-income settings. Journal of Perinatology. 2011;31(5):361-7.	Title and abstract not relevant- excluded
416.	Ahmed S, Mofijur M, Nuzhat S, Chowdhury AT, Rafa N, Uddin MA, et al. Recent developments in physical, biological, chemical, and hybrid treatment techniques for removing emerging contaminants from wastewater. Journal of hazardous materials. 2021;416:125912.	Title and abstract not relevant- excluded
417.	Ahmed S, Norton M, Williams E, Ahmed S, Shah R, Begum N, et al. Operations research to add postpartum family planning to maternal and neonatal health to improve birth spacing in Sylhet District, Bangladesh. Global Health: Science and Practice. 2013;1(2):262-76.	Title and abstract not relevant- excluded
418.	Ahmed SI, Hasan ST, Khan MA, Ahmed T. Effect of Maternal Exposure to Seasons during the Second and Third Trimesters of Pregnancy on Infant Birth Weight in Rural Bangladesh. Current Developments in Nutrition. 2020;4(3):nzaa016.	Title and abstract not relevant- excluded
419.	Akhtar S, Ismail T, Atukorala S, Arlappa N. Micronutrient deficiencies in South Asia–current status and strategies. Trends in food science & technology. 2013;31(1):55-62.	Title and abstract not relevant- excluded

SL	Articles excluded during title and abstract screening	Decision
420.	Akhter H, Aziz F, Ullah FR, Ahsan M, Islam SN. Immunoglobulins content in colostrum, transitional and mature milk of Bangladeshi mothers: Influence of parity and sociodemographic characteristics. Journal of Mother and Child. 2021;24(3):8-15.	Title and abstract not relevant- excluded
421.	Akter F, Rahman M, Pitchik HO, Winch PJ, Fernald LC, Nurul Huda TM, et al. Adaptation and integration of psychosocial stimulation, maternal mental health and nutritional interventions for pregnant and lactating women in rural Bangladesh. International journal of environmental research and public health. 2020;17(17):6233.	Title and abstract not relevant- excluded
422.	Akter S, Rahman J, Rahman MM, Abedin S. The influence of birth spacing on child survival in Bangladesh: a life table approach. World health & population. 2010;12(1):42-56.	Title and abstract not relevant- excluded
423.	Alam A, Chowdhury M, Dibley M, Raynes-Greenow C. Making a balanced plate for pregnant women to improve birthweight of infants: a study protocol for a cluster randomised controlled trial in rural Bangladesh. 2017.	Title and abstract not relevant- excluded
424.	Alam DS. Prevention of low birthweight. Emerging Societies-Coexistence of Childhood Malnutrition and Obesity. 63: Karger Publishers; 2009. p. 209-25.	Title and abstract not relevant- excluded
425.	Alauddin M, Rob U. Meeting needs of newly-married youth: the Bangladesh case. International Quarterly of Community Health Education. 1999;18(4):459-69.	Title and abstract not relevant- excluded
426.	Allen LH, Hampel D, Shahab-Ferdows S, Andersson M, Barros E, Doel AM, et al. The Mothers, Infants, and Lactation Quality (MILQ) study: a multi-center collaboration. Current developments in nutrition. 2021;5(10):nzab116.	Title and abstract not relevant- excluded
427.	Alom J, Quddus MA, Islam MA. Nutritional status of under-five children in Bangladesh: a multilevel analysis. Journal of biosocial science. 2012;44(5):525-35.	Title and abstract not relevant- excluded
428.	Ambikapathi R, Kosek MN, Lee GO, Mahopo C, Patil CL, Maciel BL, et al. How multiple episodes of exclusive breastfeeding impact estimates of exclusive breastfeeding duration: report from the eight-site MAL-ED birth cohort study. Maternal & child nutrition. 2016;12(4):740-56.	Title and abstract not relevant- excluded
429.	Ameen S, Siddique AB, Peven K, Rahman QS-u, Day LT, Shabani J, et al. Survey of women's report for 33 maternal and newborn indicators: EN-BIRTH multi-country validation study. BMC pregnancy and childbirth. 2021;21:1-17.	Title and abstract not relevant- excluded
430.	Amin S, Islam M, Choudhury F, Ahmad F, Fatematuzzaman F, Hussain F, et al. Risk Factors of Neonatal Sepsis in Neonatal Intensive Care Unit (NICU) of Mymensingh Medical College Hospital. Mymensingh medical journal: MMJ. 2021;30(3):671-7.	Title and abstract not relevant- excluded
431.	Anik AI, Rahman MM, Rahman MM, Tareque MI, Khan MN, Alam MM. Double burden of malnutrition at household level: A comparative study among Bangladesh, Nepal, Pakistan, and Myanmar. PloS one. 2019;14(8):e0221274.	Title and abstract not relevant- excluded
432.	Ara G, Khanam M, Papri N, Nahar B, Haque MA, Kabir I, et al. Peer counselling improves breastfeeding practices: A cluster randomized controlled trial in urban Bangladesh. Maternal & Child Nutrition. 2018;14(3):e12605.	Title and abstract not relevant- excluded

SL	Articles excluded during title and abstract screening	Decision
433.	Aruldas K, Kant A, Mohanan P. Care-seeking behaviors for maternal and newborn illnesses among self-help group households in Uttar Pradesh, India. Journal of Health, Population and Nutrition. 2017;36:25-34.	Title and abstract not relevant- excluded
434.	Ashraf A, Quaiyum M, Ng N, Van Minh H, Razzaque A, Masud Ahmed S, et al. Self-reported use of tobacco products in nine rural INDEPTH Health and Demographic Surveillance Systems in Asia. Global Health Action. 2009;2(1):1997.	Title and abstract not relevant- excluded
435.	Ashworth A, Khanum S. Cost-effective treatment for severely malnourished children: what is the best approach? Health Policy and Planning. 1997;12(2):115-21.	Title and abstract not relevant- excluded
436.	Association AB. Research summaries September 2013. PLoS One. 2013;8(8):e70867.	Title and abstract not relevant- excluded
437.	Azad K, Mohsin F, Zargar AH, Zabeen B, Ahmad J, Raza SA, et al. Fasting guidelines for diabetic children and adolescents. Indian journal of endocrinology and metabolism. 2012;16(4):516-8.	Title and abstract not relevant- excluded
438.	Bairagi R. Is income the only constraint on child nutrition in rural Bangladesh? Bulletin of the World Health Organization. 1980;58(5):767.	Title and abstract not relevant- excluded
439.	Baird S, Murphy M, Seager J, Jones N, Malhotra A, Alheiwidi S, et al. Intersecting disadvantages for married adolescents: life after marriage pre-and post-COVID-19 in contexts of displacement. Journal of Adolescent Health. 2022;70(3):S86-S96.	Title and abstract not relevant- excluded
440.	Baker D, Garrow A, Shiels C. Inequalities in immunisation and breast feeding in an ethnically diverse urban area: cross-sectional study in Manchester, UK. Journal of Epidemiology & Community Health. 2011;65(4):346-52.	Title and abstract not relevant- excluded
441.	Baker EJ, Sanei LC, Franklin N. Early initiation of and exclusive breastfeeding in large-scale community-based programmes in Bolivia and Madagascar. Journal of health, population, and nutrition. 2006;24(4):530.	Title and abstract not relevant- excluded
442.	Baker J, Sanghvi T, Hajeebhoy N, Abrha TH. Learning from the design and implementation of large-scale programs to improve infant and young child feeding. Food and nutrition bulletin. 2013;34(3_suppl2):S226-S30.	Title and abstract not relevant- excluded
443.	Banerjee S. Major determinants of infant mortality: District-level evidences from Odisha. Journal of Health Management. 2018;20(3):345-62.	Title and abstract not relevant- excluded
444.	Baqui AH, Williams E, Darmstadt G, Kumar V, Kiran T, Panwar D, et al. Newborn care in rural Uttar Pradesh. The Indian Journal of Pediatrics. 2007;74:241-7.	Title and abstract not relevant- excluded
445.	Barac R, Als D, Radhakrishnan A, Gaffey MF, Bhutta ZA, Barwick M. Implementation of interventions for the control of typhoid fever in low-and middle-income countries. The American journal of tropical medicine and hygiene. 2018;99(3 Suppl):79.	Title and abstract not relevant- excluded
446.	Bari W, Chowdhury RI, Islam M, Chakraborty N, Akhter H. The differentials and determinants of perinatal mortality in rural Bangladesh. The European Journal of Contraception & Reproductive Health Care. 2002;7(4):216-22.	Title and abstract not relevant- excluded
447.	Barn R, Sidhu K. Dealing with difference: professional conceptualizations of health and social care needs of Bangladeshi women in London. Journal of Social Work Research and Evaluation. 2002;3(2):145-58.	Title and abstract not relevant- excluded

SL	Articles excluded during title and abstract screening	Decision
448.	Barratt MJ, Nuzhat S, Ahsan K, Frese SA, Arzamasov AA, Sarker SA, et al. Bifidobacterium infantis treatment promotes weight gain in Bangladeshi infants with severe acute malnutrition. Science Translational Medicine. 2022;14(640):eabk1107.	Title and abstract not relevant- excluded
449.	Barua S, Tarannum S, Nahar L, Mohiduzzaman M. Retinol and alpha-tocopherol content in breast milk of Bangladeshi mothers under low socio-economic status. International journal of food sciences and nutrition. 1997;48(1):13-8.	Title and abstract not relevant- excluded
450.	Bashed M, Gazi Mahabubul Alam GMA, Kabir M, Abul Quasem Al-Amin AQA-A. Male infertility in Bangladesh: what serve better-pharmacological help or awareness programme? 2012.	Title and abstract not relevant- excluded
451.	Basnet S. Role of Resources for Care in Improving Care Behaviors, Children's Nutritional Status and Early Childhood Development in Low-and Middle-Income Countries. 2019.	Title and abstract not relevant- excluded
452.	Bauer B, Hedlund C. Nurture project international: lactation work in crisis. Journal of Human Lactation. 2018;34(3):503-6.	Title and abstract not relevant- excluded
453.	Bégin F, Aguayo VM. First foods: Why improving young children's diets matter. Maternal & Child Nutrition. 2017;13:e12528.	Title and abstract not relevant- excluded
454.	Bergkvist C, Aune M, Nilsson I, Sandanger TM, Hamadani JD, Tofail F, et al. Occurrence and levels of organochlorine compounds in human breast milk in Bangladesh. Chemosphere. 2012;88(7):784-90.	Title and abstract not relevant- excluded
455.	Bhattacharya S. History of development of oral rehydration therapy. Indian journal of public health. 1994;38(2):39-43.	Title and abstract not relevant- excluded
456.	Bhowmik B, Siddiquee T, Mdala I, Nesa LQ, Shelly SJ, Hassan Z, et al. Vitamin D3 and B12 supplementation in pregnancy. diabetes research and clinical practice. 2021;174:108728.	Title and abstract not relevant- excluded
457.	Bhuiya A, Streatfield K. Mothers' education and survival of female children in a rural area of Bangladesh. Population studies. 1991;45(2):253-64.	Title and abstract not relevant- excluded
458.	Biks GA, Blencowe H, Hardy VP, Geremew BM, Angaw DA, Wagnew A, et al. Birthweight data completeness and quality in population-based surveys: EN-INDEPTH study. Population Health Metrics. 2021;19:1-16.	Title and abstract not relevant- excluded
459.	Bilal SM, Moser A, Blanco R, Spigt M, Dinant GJ. Practices and challenges of growth monitoring and promotion in Ethiopia: a qualitative study. Journal of health, population, and nutrition. 2014;32(3):441.	Title and abstract not relevant- excluded
460.	Bilic M, Qamar H, Onoyovwi A, Korsiak J, Papp E, Al Mahmud A, et al. Prenatal vitamin D and cord blood insulin-like growth factors in Dhaka, Bangladesh. Endocrine Connections. 2019;8(6):745-53.	Title and abstract not relevant- excluded
461.	Billah SM, Ferdous TE, Karim MA, Dibley MJ, Raihana S, Moinuddin M, et al. A community-based cluster randomised controlled trial to evaluate the effectiveness of different bundles of nutrition-specific interventions in improving mean length-for-age z score among children at 24 months of age in rural Bangladesh: study protocol. BMC Public Health. 2017;17:1-12.	Title and abstract not relevant- excluded
462.	Billah SM, Ferdous TE, Siddique AB, Raynes-Greenow C, Kelly P, Choudhury N, et al. BILLAH et al. Maternal and Child Nutrition. 2022;18(3).	Title and abstract not relevant- excluded

SL	Articles excluded during title and abstract screening	Decision
463.	Black MM, Baqui AH, Zaman K, McNary SW, Le K, Arifeen SE, et al. Depressive symptoms among rural Bangladeshi mothers: implications for infant development. Journal of Child Psychology and Psychiatry. 2007;48(8):764-72.	Title and abstract not relevant- excluded
464.	Black MM, Tofail F, Hodges EA, Bann CM, Hamadani JD, Aktar S, et al. Rethinking responsive feeding: Insights from Bangladesh. Nutrients. 2022;14(15):3156.	Title and abstract not relevant- excluded
465.	Bogard JR, Marks GC, Mamun A, Thilsted SH. Non-farmed fish contribute to greater micronutrient intakes than farmed fish: results from an intra-household survey in rural Bangladesh. Public Health Nutrition. 2017;20(4):702-11.	Title and abstract not relevant- excluded
466.	Boggs D, Milner KM, Chandna J, Black M, Cavallera V, Dua T, et al. Rating early child development outcome measurement tools for routine health programme use. Archives of disease in childhood. 2019;104(Suppl 1):S22-S33.	Title and abstract not relevant- excluded
467.	Bourassa MW, Osendarp SJ, Adu-Afarwuah S, Ahmed S, Ajello C, Bergeron G, et al. Review of the evidence regarding the use of antenatal multiple micronutrient supplementation in low-and middle-income countries. Annals of the new York Academy of Sciences. 2019;1444(1):6-21.	Title and abstract not relevant- excluded
468.	Bowler IM. Stereotypes of women of Asian descent in midwifery: some evidence. Midwifery. 1993;9(1):7-16.	Title and abstract not relevant- excluded
469.	Breuer A, Asiedu E. Can gender-targeted employment interventions help enhance community participation? Evidence from urban Togo. World development. 2017;96:390-407.	Title and abstract not relevant- excluded
470.	Bromage S, Ahmed T, Fawzi WW. Calcium deficiency in Bangladesh: burden and proposed solutions for the first 1000 days. Food and nutrition bulletin. 2016;37(4):475-93.	Title and abstract not relevant- excluded
471.	Burkart K, Causey K, Cohen AJ, Wozniak SS, Salvi DD, Abbafati C, et al. Estimates, trends, and drivers of the global burden of type 2 diabetes attributable to PM2· 5 air pollution, 1990–2019: an analysis of data from the Global Burden of Disease Study 2019. The Lancet Planetary Health. 2022;6(7):e586-e600.	Title and abstract not relevant- excluded
472.	Burrell A, Kueter AM, Ariful S, Rahaman H, Iellamo A, Mothabbir G. Appropriate infant and young child feeding practices in an emergency for non-breastfed infants under six months: The Rohingya experience. Journal of Human Lactation. 2020;36(3):510-8.	Title and abstract not relevant- excluded
473.	Busert LK, Neuman M, Rehfuess EA, Dulal S, Harthan J, Chaube SS, et al. Dietary diversity is positively associated with deviation from expected height in rural Nepal. The Journal of nutrition. 2016;146(7):1387-93.	Title and abstract not relevant- excluded
474.	Cairncross S, Hunt C, Boisson S, Bostoen K, Curtis V, Fung IC, et al. Water, sanitation and hygiene for the prevention of diarrhoea. International journal of epidemiology. 2010;39(suppl_1):i193-i205.	Title and abstract not relevant- excluded
475.	Campbell RK, Hurley KM, Shamim AA, Shaikh S, Chowdhury ZT, Mehra S, et al. Complementary food supplements increase dietary nutrient adequacy and do not replace home food consumption in children 6–18 months old in a randomized controlled trial in rural Bangladesh. The Journal of nutrition. 2018;148(9):1484-92.	Title and abstract not relevant- excluded
476.	Castine SA, Bogard JR, Barman BK, Karim M, Mokarrom Hossain M, Kunda M, et al. Homestead pond polyculture can improve access to nutritious small fish. Food Security. 2017;9:785-801.	Title and abstract not relevant- excluded

SL	Articles excluded during title and abstract screening	Decision
477.	Catala-Lopez F, Padron-Monedero A, Collaborators GAYAC. The global burden of adolescent and young adult cancer in 2019: a systematic analysis for the Global Burden of Disease Study 2019. 2022.	Title and abstract not relevant- excluded
478.	Chakravarty S, Kamal M. MUC1 and E-cadherin immunohistochemistry of endometrium cannot predict the outcome of in vitro fertilization: a case-control study. F1000Research. 2019;8:162.	Title and abstract not relevant- excluded
479.	Chandrashekhar T, Joshi H, Binu V, Shankar P, Rana M, Ramachandran U. Breast-feeding initiation and determinants of exclusive breast-feeding–a questionnaire survey in an urban population of western Nepal. Public health nutrition. 2007;10(2):192-7.	Title and abstract not relevant- excluded
480.	Chaudhury RH. Determinants of nutrient adequacy for lactating and pregnant mothers in a rural area of Bangladesh. Food and Nutrition Bulletin. 1985;7(1):1-7.	Title and abstract not relevant- excluded
481.	Cheang HK, Yeung CY, Cheah I, Tjipta GD, Lubis BM, Garza-Bulnes R, et al. A survey among healthcare professionals from seven countries reported diverse nutritional practices of late preterm infants. Acta Paediatrica. 2022;111(7):1362-71.	Title and abstract not relevant- excluded
482.	Choudhury S, Headey DD. Household dairy production and child growth: Evidence from Bangladesh. Economics & Human Biology. 2018;30:150-61.	Title and abstract not relevant- excluded
483.	Chowdhury AMR, Karim F, Sarkar S, Cash RA, Bhuiya A. The status of ORT in Bangladesh: how widely is it used? Health Policy and Planning. 1997;12(1):58-66.	Title and abstract not relevant- excluded
484.	Chowdhury M, Raynes-Greenow C, Kelly P, Alam NA, Afsana K, Billah SM, et al. The impact of antenatal balanced plate nutrition education for pregnant women on birth weight: a cluster randomised controlled trial in rural Bangladesh. Nutrients. 2022;14(21):4687.	Title and abstract not relevant- excluded
485.	Chowdhury RI, Islam MA, Chakraborty N, Akhter HH. Determinants of antenatal morbidity: a multivariate analysis. World health & population. 2007;9(3):9-18.	Title and abstract not relevant- excluded
486.	Chowdhury S, Sarkar N, Roy SK. Impact of lactational performance on bone mineral density in marginally-nourished Bangladeshi women. Journal of Health, Population and Nutrition. 2002:26-30.	Title and abstract not relevant- excluded
487.	Christian P, Kim J, Mehra S, Shaikh S, Ali H, Shamim AA, et al. Effects of prenatal multiple micronutrient supplementation on growth and cognition through 2 y of age in rural Bangladesh: the JiVitA-3 Trial. The American journal of clinical nutrition. 2016;104(4):1175-82.	Title and abstract not relevant- excluded
488.	Clemens JD, Stanton B, Stoll B, Shahid NS, Banu H, CHOWDHURY AA. Breast feeding as a determinant of severity in shigellosis: evidence for protection throughout the first three years of life in Bangladeshi children. American journal of epidemiology. 1986;123(4):710-20.	Title and abstract not relevant- excluded
489.	Colgate ER, Haque R, Dickson DM, Carmolli MP, Mychaleckyj JC, Nayak U, et al. Delayed dosing of oral rotavirus vaccine demonstrates decreased risk of rotavirus gastroenteritis associated with serum zinc: a randomized controlled trial. Clinical Infectious Diseases. 2016;63(5):634-41.	Title and abstract not relevant- excluded
490.	Colombara DV, Cowgill KD, Faruque AS. Risk factors for severe cholera among children under five in rural and urban Bangladesh, 2000–2008: a hospital-based surveillance study. PLoS One. 2013;8(1):e54395.	Title and abstract not relevant- excluded
491.	Cooper CM, Ogutu A, Matiri E, Tappis H, Mackenzie D, Pfitzer A, et al. Maximizing opportunities: family planning and maternal, infant, and young child nutrition integration in Bondo Sub-County, Kenya. Maternal and child health journal. 2017;21(10):1880-9.	Title and abstract not relevant- excluded

SL	Articles excluded during title and abstract screening	Decision
492.	Cromwell EA, Schmidt CA, Kwong KT, Pigott DM, Mupfasoni D, Biswas G, et al. The global distribution of lymphatic filariasis, 2000–18: a geospatial analysis. The Lancet Global Health. 2020;8(9):e1186-e94.	Title and abstract not relevant- excluded
493.	Cruz-Rivera E, Friedlander M. Effects of algal phenotype on mesograzer feeding. Marine Ecology Progress Series. 2013;490:69-78.	Title and abstract not relevant- excluded
494.	Dalal K. Causes and consequences of violence against child labour and women in developing countries: Karolinska Institutet (Sweden); 2008.	Title and abstract not relevant- excluded
495.	Dam Lam R, Barman BK, Lozano Lazo DP, Khatun Z, Parvin L, Choudhury A, et al. Sustainability impacts of ecosystem approaches to small-scale aquaculture in Bangladesh. Sustainability Science. 2022;17(1):295-313.	Title and abstract not relevant- excluded
496.	Darj E, Newaz MS, Zaman MH. Pharmacists’ perception of their challenges at work, focusing on antimicrobial resistance: a qualitative study from Bangladesh. Global health action. 2019;12(sup1):1735126.	Title and abstract not relevant- excluded
497.	Darling AL, Blackbourn DJ, Ahmadi KR, Lanham-New SA. Vitamin D supplement use and associated demographic, dietary and lifestyle factors in 8024 South Asians aged 40–69 years: analysis of the UK Biobank cohort. Public health nutrition. 2018;21(14):2678-88.	Title and abstract not relevant- excluded
498.	Darmstadt GL, Munar W, Henry SK. Newborn health: Everybody's business. Global Public Health. 2014;9(7):752-9.	Title and abstract not relevant- excluded
499.	Daru J, Moores R, Dodds J, Rayment J, Allard S, Khan K. Non-anaemic iron deficiency in pregnancy: the views of health service users and health care professionals. Transfusion Medicine. 2015;25(1):27-32.	Title and abstract not relevant- excluded
500.	Das JK, Hoodbhoy Z, Salam RA, Bhutta AZ, Valenzuela-Rubio NG, Prinzo ZW, et al. Lipid-based nutrient supplements for maternal, birth, and infant developmental outcomes. Cochrane Database of Systematic Reviews. 2018(8).	Title and abstract not relevant- excluded
501.	Dasgupta S, Mustafa G, Paul T, Wheeler D. The socioeconomics of fish consumption and child health: An observational cohort study from Bangladesh. World Development. 2021;137:105201.	Title and abstract not relevant- excluded
502.	Dash M, Misra P, Subudhi K. Utilization of the prevention of parent-to-child transmission of HIV (PPTCT) services in a tertiary care hospital, Odisha, India. Bangladesh Journal of Medical Science. 2014;13(2):163.	Title and abstract not relevant- excluded
503.	Daviaud E, Nkonki L, Ijumba P, Doherty T, Lawn JE, Owen H, et al. South-Africa (Goodstart III) trial: community-based maternal and newborn care economic analysis. Health policy and planning. 2017;32(suppl_1):i53-i63.	Title and abstract not relevant- excluded
504.	Devkota S, Panda B. Socioeconomic gradients in early childhood health: evidence from Bangladesh and Nepal. International Journal for Equity in Health. 2016;15:1-16.	Title and abstract not relevant- excluded
505.	Dewey KG, Oaks BM. U-shaped curve for risk associated with maternal hemoglobin, iron status, or iron supplementation. The American journal of clinical nutrition. 2017;106:1694S-702S.	Title and abstract not relevant- excluded
506.	Di Stefano L, Bottecchia M, Yargawa J, Akuze J, Haider MM, Galiwango E, et al. Stillbirth maternity care measurement and associated factors in population-based surveys: EN-INDEPTH study. Population health metrics. 2021;19:1-16.	Title and abstract not relevant- excluded

SL	Articles excluded during title and abstract screening	Decision
507.	Donowitz JR, Cook H, Alam M, Tofail F, Kabir M, Colgate ER, et al. Role of maternal health and infant inflammation in nutritional and neurodevelopmental outcomes of two-year-old Bangladeshi children. PLoS neglected tropical diseases. 2018;12(5):e0006363.	Title and abstract not relevant- excluded
508.	Douglas N. Befriending breastfeeding: a home-based antenatal pilot for South Asian families. Community Practitioner. 2012;85(6).	Title and abstract not relevant- excluded
509.	Edmonston B. Human reproduction in Bangladesh: a microanalytic simulation model. Janasamkhya. 1983;1(1):61-73.	Title and abstract not relevant- excluded
510.	Walsh V, Brown JVE, Copperthwaite BR, Oddie SJ, McGuire W. Early full enteral feeding for preterm or low birth weight infants. Cochrane Database of Systematic Reviews. 2020(12).	Title and abstract not relevant- excluded
511.	Xiong T, Maheshwari A, Neu J, Ei-Saie A, Pammi M. An overview of systematic reviews of randomized-controlled trials for preventing necrotizing enterocolitis in preterm infants. Neonatology. 2020;117(1):46-56.	Title and abstract not relevant- excluded
512.	Acharya P, Khanal V. The effect of mother's educational status on early initiation of breastfeeding: further analysis of three consecutive Nepal Demographic and Health Surveys. BMC Public Health. 2015;15:1-12.	Title and abstract not relevant- excluded
513.	Adewuyi EO, Adefemi K. Breastfeeding in Nigeria: a systematic review. 2016.	Title and abstract not relevant- excluded
514.	Adhikari M, Khanal V, Karkee R, Gavidia T. Factors associated with early initiation of breastfeeding among Nepalese mothers: further analysis of Nepal Demographic and Health Survey, 2011. International breastfeeding journal. 2014;9:1-9.	Title and abstract not relevant- excluded
515.	Adhikari N, Acharya K, Upadhya DP, Pathak S, Pokharel S, Pradhan PMS. Infant and young child feeding practices and its associated factors among mothers of under two years children in a western hilly region of Nepal. PloS one. 2021;16(12):e0261301.	Title and abstract not relevant- excluded
516.	Agampodi SB, Agampodi TC, Piyaseeli UKD. Breastfeeding practices in a public health field practice area in Sri Lanka: a survival analysis. International Breastfeeding Journal. 2007;2:1-7.	Title and abstract not relevant- excluded
517.	Agnarsson I, Mpello A, Gunnlaugsson G, Hofvander Y, Greiner T. Infant feeding practices during the first six month of life in the rural areas of Tanzania. East African medical journal. 2001;78(1):9-13.	Title and abstract not relevant- excluded
518.	Ahmed AE, Salih OA. Determinants of the early initiation of breastfeeding in the Kingdom of Saudi Arabia. International breastfeeding journal. 2019;14:1-13.	Title and abstract not relevant- excluded
519.	Al Juaid DA, Binns CW, Giglia RC. Breastfeeding in Saudi Arabia: a review. International Breastfeeding Journal. 2014;9:1-9.	Title and abstract not relevant- excluded
520.	Ali F, Mgongo M, Mamseri R, George JM, Mboya IB, Msuya SE. Prevalence of and factors associated with early initiation of breastfeeding among women with children aged< 24 months in Kilimanjaro region, northern Tanzania: a community-based cross-sectional study. International Breastfeeding Journal. 2020;15:1-10.	Title and abstract not relevant- excluded

SL	Articles excluded during title and abstract screening	Decision
521.	Frith AL, Naved RT, Persson LA, Frongillo EA. Early prenatal food supplementation ameliorates the negative association of maternal stress with birth size in a randomised trial. Maternal & child nutrition. 2015;11(4):537-49.	Title and abstract not relevant- excluded
522.	Frith AL, Naved RT, Persson LA, Rasmussen KM, Frongillo EA. Early participation in a prenatal food supplementation program ameliorates the negative association of food insecurity with quality of maternal-infant interaction. The Journal of nutrition. 2012;142(6):1095-101.	Title and abstract not relevant- excluded
523.	Fuchs C, Sultana T, Ahmed T, Iqbal Hossain M. Factors associated with acute malnutrition among children admitted to a diarrhoea treatment facility in Bangladesh. International journal of pediatrics. 2014;2014(1):267806.	Title and abstract not relevant- excluded
524.	Gaillard C, Verger EO, Dury S, Dop MC, El Ati J, Group MS. Farm production diversity and women's dietary diversity: Evidence from central Tunisia. PLoS One. 2022;17(2):e0263276.	Title and abstract not relevant- excluded
525.	Galasso E, Weber AM, Stewart CP, Ratsifandrihamanana L, Fernald LC. Effects of nutritional supplementation and home visiting on growth and development in young children in Madagascar: a cluster-randomised controlled trial. The Lancet Global Health. 2019;7(9):e1257-e68.	Title and abstract not relevant- excluded
526.	Gao Y, Kc A, Chen C, Huang Y, Wang Y, Zou S, et al. Inequality in measles vaccination coverage in the "big six" countries of the WHO South-East Asia region. Human vaccines & immunotherapeutics. 2020;16(7):1485-97.	Title and abstract not relevant- excluded
527.	Garcia R, Ali N, Griffiths M, Randhawa G. Understanding the consumption of folic acid during preconception, among Pakistani, Bangladeshi and white British mothers in Luton, UK: a qualitative study. BMC pregnancy and childbirth. 2018;18:1-9.	Title and abstract not relevant- excluded
528.	Garg A, Chadha R. Index for measuring the quality of complementary feeding practices in rural India. Journal of health, population, and nutrition. 2009;27(6):763.	Title and abstract not relevant- excluded
529.	Gebreselassie SG, Gase FE, Deressa MU. Prevalence and correlates of prenatal vitamin A deficiency in rural Sidama, Southern Ethiopia. Journal of health, population, and nutrition. 2013;31(2):185.	Title and abstract not relevant- excluded
530.	Gera T, Shah D, Garner P, Richardson M, Sachdev HS. Integrated management of childhood illness (IMCI) strategy for children under five. Cochrane Database of Systematic Reviews. 2016(6).	Title and abstract not relevant- excluded
531.	Giashuddin M, Kabir M, Hasan M. Economic disparity and child nutrition in Bangladesh. The Indian Journal of Pediatrics. 2005;72:481-7.	Title and abstract not relevant- excluded
532.	Gibson E, Stacey N, Sunderland TC, Adhuri DS. Dietary diversity and fish consumption of mothers and their children in fisher households in Komodo District, eastern Indonesia. PloS one. 2020;15(4):e0230777.	Title and abstract not relevant- excluded
533.	Gibson E, Stacey N, Sunderland TC, Adhuri DS. Coping or adapting? Experiences of food and nutrition insecurity in specialised fishing households in Komodo District, eastern Indonesia. BMC Public Health. 2021;21:1-17.	Title and abstract not relevant- excluded
534.	Gilgen D, Mascie-Taylor C, Rosetta L. Intestinal helminth infections, anaemia and labour productivity of female tea pluckers in Bangladesh. Tropical medicine & international health. 2001;6(6):449-57.	Title and abstract not relevant- excluded

SL	Articles excluded during title and abstract screening	Decision
535.	Giovino GA, Mirza SA, Samet JM, Gupta PC, Jarvis MJ, Bhala N, et al. Tobacco use in 3 billion individuals from 16 countries: an analysis of nationally representative cross-sectional household surveys. The Lancet. 2012;380(9842):668-79.	Title and abstract not relevant- excluded
536.	Giovino GA, Mirza SA, Samet JM, Gupta PC, Jarvis MJ, Bhala N, et al. Department of error:" Tobacco use in 3 billion individuals from 16 countries: An analysis of nationally representative cross-sectional household surveys.". 2013.	Title and abstract not relevant- excluded
537.	Gomes F, Agustina R, Black RE, Christian P, Dewey KG, Kraemer K, et al. Multiple micronutrient supplements versus iron-folic acid supplements and maternal anemia outcomes: An iron dose analysis. Annals of the New York Academy of Sciences. 2022;1512(1):114-25.	Title and abstract not relevant- excluded
538.	Greenhalgh T, Clinch M, Afsar N, Choudhury Y, Sudra R, Campbell-Richards D, et al. Socio-cultural influences on the behaviour of South Asian women with diabetes in pregnancy: qualitative study using a multi-level theoretical approach. BMC medicine. 2015;13:1-15.	Title and abstract not relevant- excluded
539.	Greiner T, Mitra S. Evaluation of the effect of a breastfeeding message integrated into a larger communication project. Journal of tropical pediatrics. 1999;45(6):351-7.	Title and abstract not relevant- excluded
540.	Groopman JD, Egner PA, Schulze KJ, Wu LS-F, Merrill R, Mehra S, et al. Aflatoxin exposure during the first 1000 days of life in rural South Asia assessed by aflatoxin B1-lysine albumin biomarkers. Food and Chemical Toxicology. 2014;74:184-9.	Title and abstract not relevant- excluded
541.	Gupta DN, Rajendran K, Mondal SK, Ghosh S, Bhattacharya SK. Operational feasibility of implementing community-based zinc supplementation: impact on childhood diarrheal morbidity. The Pediatric infectious disease journal. 2007;26(4):306-10.	Title and abstract not relevant- excluded
542.	Gustin K, Tofail F, Vahter M, Kippler M. Cadmium exposure and cognitive abilities and behavior at 10 years of age: a prospective cohort study. Environment international. 2018;113:259-68.	Title and abstract not relevant- excluded
543.	Habib AA, Hasan MK, Islam S, Ahmed MM, Aman AHM, Bagwari A, et al. Voltage equalization circuit for retired batteries for energy storage applications. Energy Reports. 2022;8:367-74.	Title and abstract not relevant- excluded
544.	Habtewold TD, Mohammed SH, Endalamaw A, Mulugeta H, Dessie G, Berhe DF, et al. Higher educational and economic status are key factors for the timely initiation of breastfeeding in Ethiopia: a review and meta-analysis. Acta Paediatrica. 2020;109(11):2208-18.	Title and abstract not relevant- excluded
545.	Haider MM, Mahmud K, Blencowe H, Ahmed T, Akuze J, Cousens S, et al. Gestational age data completeness, quality and validity in population-based surveys: EN-INDEPTH study. Population health metrics. 2021;19:1-18.	Title and abstract not relevant- excluded
546.	Haider R, Kabir I, Huttly SR, Ashworth A. Training peer counselors to promote and support exclusive breastfeeding in Bangladesh. Journal of Human Lactation. 2002;18(1):7-12.	Title and abstract not relevant- excluded
547.	Haile D, Belachew T, Berhanu G, Setegn T, Biadgilign S. Complementary feeding practices and associated factors among HIV positive mothers in Southern Ethiopia. Journal of Health, Population and Nutrition. 2015;34:1-9.	Title and abstract not relevant- excluded

SL	Articles excluded during title and abstract screening	Decision
548.	Hailu S, Woldemichael B. Dietary diversity and associated factors among pregnant women attending antenatal care at public health facilities in Bale Zone, Southeast Ethiopia. Nutrition and Dietary Supplements. 2019:1-8.	Title and abstract not relevant- excluded
549.	Halim A, Utz B, Biswas A, Rahman F, Van Den Broek N. Cause of and contributing factors to maternal deaths; a cross-sectional study using verbal autopsy in four districts in Bangladesh. BJOG: An International Journal of Obstetrics & Gynaecology. 2014;121:86-94.	Title and abstract not relevant- excluded
550.	Halim MR, Saha S, Haque IU, Jesmin S, Nishat RJ, Islam AA, et al. ABO blood group and outcomes in patients with COVID-19 admitted in the intensive care unit (ICU): a retrospective study in a tertiary-level hospital in Bangladesh. Journal of multidisciplinary healthcare. 2021:2429-36.	Title and abstract not relevant- excluded
551.	Hamadani JD, Tofail F, Huda SN, Alam DS, Ridout DA, Attanasio O, et al. Cognitive deficit and poverty in the first 5 years of childhood in Bangladesh. Pediatrics. 2014;134(4):e1001-e8.	Title and abstract not relevant- excluded
552.	Haq AM, Giasuddin A, Huque MM. Serum total homocysteine and lipoprotein (a) levels in acute myocardial infarction and their response to treatment with vitamins. J Coll Physicians Surg Pak. 2011;21(5):266-70.	Title and abstract not relevant- excluded
553.	Haque MA, Choudhury N, Ahmed ST, Farzana FD, Ali M, Naz F, et al. The large-scale community-based programme 'suchana'improved maternal healthcare practices in north-eastern Bangladesh: Findings from a cluster randomized pre-post study. Maternal & child nutrition. 2022;18(1):e13258.	Title and abstract not relevant- excluded
554.	Haque MM, Alam MR, Alam MM, Basak B, Sumi KR, Belton B, et al. Integrated floating cage aquageoponics system (IFCAS): An innovation in fish and vegetable production for shaded ponds in Bangladesh. Aquaculture Reports. 2015;2:1-9.	Title and abstract not relevant- excluded
555.	Harding KL, Matias SL, Mridha MK, Moniruzzaman M, Vosti SA, Hussain S, et al. Adherence to recommendations on lipid-based nutrient supplement and iron and folic acid tablet consumption among pregnant and lactating women participating in a community health programme in northwest Bangladesh. Maternal & child nutrition. 2017;13(1):e12252.	Title and abstract not relevant- excluded
556.	Harohau D, Sulu RJ, Phillips MJ, Sukulu M, Pickering T, Schwarz AM. Improving household tilapia (Oreochromis mossambicus) aquaculture through participatory action research. Aquaculture. 2016;465:272-86.	Title and abstract not relevant- excluded
557.	Harris J, Frongillo EA, Nguyen PH, Kim SS, Menon P. Changes in the policy environment for infant and young child feeding in Vietnam, Bangladesh, and Ethiopia, and the role of targeted advocacy. BMC Public Health. 2017;17:107-23.	Title and abstract not relevant- excluded
558.	Harris-Fry H, Azad K, Kuddus A, Shaha S, Nahar B, Hossen M, et al. Socio-economic determinants of household food security and women's dietary diversity in rural Bangladesh: a cross-sectional study. Journal of Health, Population and Nutrition. 2015;33:1-12.	Title and abstract not relevant- excluded
559.	Harris-Fry H, Shrestha N, Costello A, Saville NM. Determinants of intra-household food allocation between adults in South Asia–a systematic review. International journal for equity in health. 2017;16:1-21.	Title and abstract not relevant- excluded

SL	Articles excluded during title and abstract screening	Decision
560.	Harun-Or-Rashid M, Khatun UF, Yoshida Y, Morita S, Chowdhury N, Sakamoto J. Iron and iodine deficiencies among under-2 children, adolescent girls, and pregnant women of Bangladesh: association with common diseases. Nagoya journal of medical science. 2009;71(1-2):39.	Title and abstract not relevant- excluded
561.	Harun-Or-Rashid M, Sarkar AK, Hasan MMI, Hasan M, Juyena NS. Productive, reproductive, and estrus characteristics of different breeds of buffalo cows in Bangladesh. Journal of advanced veterinary and animal research. 2019;6(4):553.	Title and abstract not relevant- excluded
562.	Haselow NJ, Stormer A, Pries A. Evidence-based evolution of an integrated nutrition-focused agriculture approach to address the underlying determinants of stunting. Maternal & child nutrition. 2016;12:155-68.	Title and abstract not relevant- excluded
563.	Hasnat Milton A, Smith W, Rahman B, Ahmed B, Shahidullah S, Hossain Z, et al. Prevalence and determinants of malnutrition among reproductive aged women of rural Bangladesh. Asia Pacific Journal of Public Health. 2010;22(1):110-7.	Title and abstract not relevant- excluded
564.	Head SK, Yount KM, Sibley LM. Delays in recognition of and care-seeking response to prolonged labor in Bangladesh. Social science & medicine. 2011;72(7):1157-68.	Title and abstract not relevant- excluded
565.	Headey D, Hoddinott J, Park S. Drivers of nutritional change in four South Asian countries: a dynamic observational analysis. Maternal & child nutrition. 2016;12:210-8.	Title and abstract not relevant- excluded
566.	Heaney CD, Kmush B, Navas-Acien A, Francesconi K, Gössler W, Schulze K, et al. Arsenic exposure and hepatitis E virus infection during pregnancy. Environmental research. 2015;142:273-80.	Title and abstract not relevant- excluded
567.	Hezam IM, Nayeem MK, Foul A, Alrasheedi AF. COVID-19 Vaccine: A neutrosophic MCDM approach for determining the priority groups. Results in physics. 2021;20:103654.	Title and abstract not relevant- excluded
568.	Highet G, Ritchie D, Platt S, Amos A, Hargreaves K, Martin C, et al. The re-shaping of the life-world: male British Bangladeshi smokers and the English smoke-free legislation. Ethnicity & health. 2011;16(6):519-33.	Title and abstract not relevant- excluded
569.	Hjertholm KG, Holmboe-Ottesen G, Iversen PO, Mdala I, Munthali A, Maleta K, et al. Seasonality in associations between dietary diversity scores and nutrient adequacy ratios among pregnant women in rural Malawi–a cross-sectional study. Food & nutrition research. 2019;63.	Title and abstract not relevant- excluded
570.	Hoddinott J, Karachiwalla NI, Ledlie NA, Roy S. Adolescent girls' infant and young child nutrition knowledge levels and sources differ among rural and urban samples in Bangladesh. Maternal & child nutrition. 2016;12(4):885-97.	Title and abstract not relevant- excluded
571.	Holman DJ, Grimes MA, Achterberg JT, Brindle E, O'Connor KA. Distribution of postpartum amenorrhea in rural Bangladeshi women. American Journal of Physical Anthropology: The Official Publication of the American Association of Physical Anthropologists. 2006;129(4):609-19.	Title and abstract not relevant- excluded
572.	Holme F, Kapambwe S, Nessa A, Basu P, Murillo R, Jeronimo J. Scaling up proven innovative cervical cancer screening strategies: challenges and opportunities in implementation at the population level in low- and lower-middle-income countries. International Journal of Gynecology & Obstetrics. 2017;138:63-8.	Title and abstract not relevant- excluded
573.	Hoque A, Selwyn BJ. Birth practice patterns in urban slums of Dhaka, Bangladesh. Women & health. 1996;24(1):41-58.	Title and abstract not relevant- excluded

SL	Articles excluded during title and abstract screening	Decision
574.	Hoque M, Annur B, Sayeed M, Mamun M. Risk Factors of Malnutrition in Under-5 Children of Slum Area in Dhaka City. Mymensingh medical journal: MMJ. 2021;30(1):196-201.	Title and abstract not relevant- excluded
575.	Hoque MN, Istiaq A, Rahman MS, Islam MR, Anwar A, Siddiki AZ, et al. Microbiome dynamics and genomic determinants of bovine mastitis. Genomics. 2020;112(6):5188-203.	Title and abstract not relevant- excluded
576.	Hose I, Durham J, Phengsavanh A, Sychareun V, Vongxay V, Xaysomphou D, et al. Perceptions and management of postpartum haemorrhage among remote communities in Lao PDR. Rural and Remote Health. 2020;20(1):156-65.	Title and abstract not relevant- excluded
577.	Hossain M, Islam Z, Sultana S, Rahman AS, Hotz C, Haque MA, et al. Effectiveness of workplace nutrition programs on anemia status among female readymade garment workers in Bangladesh: a program evaluation. Nutrients. 2019;11(6):1259.	Title and abstract not relevant- excluded
578.	Hossain S, Hussain J, Bhowmick S, Sarkar M, Basunia M, Al Mamun A, et al. Docosahexaenoic Acid (DHA, C22: 6, ω-3) composition of milk and mammary gland tissues of lactating mother rats is severely affected by lead (Pb) exposure. Biological trace element research. 2020;195:525-34.	Title and abstract not relevant- excluded
579.	Hossain SJ, Roy BR, Salveen N-E, Hasan MI, Tipu SMU, Shiraji S, et al. Effects of adding psychosocial stimulation for children of lactating mothers using an unconditional cash transfer platform on neurocognitive behavior of children in rural Bangladesh: protocol for a cluster randomized controlled trial. BMC psychology. 2019;7:1-6.	Title and abstract not relevant- excluded
580.	Howell SR, Barnett AG, Underwood MR. The use of pre-conceptional folic acid as an indicator of uptake of a health message amongst white and Bangladeshi women in Tower Hamlets, east London. Family Practice. 2001;18(3):300-3.	Title and abstract not relevant- excluded
581.	Huda TMN, Jahir T, Sarker S, Yeasmin F, Masud AA, Sultana J, et al. Formative research to design a child-friendly latrine in bangladesh. International journal of environmental research and public health. 2021;18(21):11092.	Title and abstract not relevant- excluded
582.	Hui CY, Abdulla A, Ahmed Z, Goel H, Habib GM, Hock TT, et al. Mapping national information and communication technology (ICT) infrastructure to the requirements of potential digital health interventions in low-and middle-income countries. Journal of global health. 2022;12.	Title and abstract not relevant- excluded
583.	Huq NL, Ahmed A, Haque Na, Hossaine M, Uddin J, Ahmed F, et al. Effect of an integrated maternal health intervention on skilled provider’s care for maternal health in remote rural areas of Bangladesh: a pre and post study. BMC pregnancy and childbirth. 2015;15:1-15.	Title and abstract not relevant- excluded
584.	Hussain AZ, Rafiquzzaman M. Determinants of weaning age in rural Bangladesh. Social biology. 1994;41(1-2):78-82.	Title and abstract not relevant- excluded
585.	Idris F, Murad ZN, Gan BC, Noh KB, Yi YX, Ming OF, et al. Complicated Paediatric Bronchial Foreign Body: A Novel Extraction Technique. Bangladesh Journal of Otorhinolaryngology. 2021;27(2):177-83.	Title and abstract not relevant- excluded
586.	Investigators M-EN. Childhood stunting in relation to the pre-and postnatal environment during the first 2 years of life: the MAL-ED longitudinal birth cohort study. PLoS medicine. 2017;14(10):e1002408.	Title and abstract not relevant- excluded
587.	Islam FB, Sharma M. Socio-economic determinants of women’s livelihood time use in rural Bangladesh. GeoJournal. 2022;87(Suppl 4):439-51.	Title and abstract not relevant- excluded

SL	Articles excluded during title and abstract screening	Decision
588.	Islam M, Lamberg-Allardt C, Bhuyan M, Salamatullah Q. Iron status of premenopausal women in two regions of Bangladesh: prevalence of deficiency in high and low socio-economic groups. European journal of clinical nutrition. 2001;55(7):598-604.	Title and abstract not relevant- excluded
589.	Islam M, Lamberg-Allardt C, Kärkkäinen M, Outila T, Salamatullah Q, Shamim A. Vitamin D deficiency: a concern in premenopausal Bangladeshi women of two socio-economic groups in rural and urban region. European Journal of Clinical Nutrition. 2002;56(1):51-6.	Title and abstract not relevant- excluded
590.	Islam M, Rahman S, Islam M, Samad A. Effect of maternal status and breastfeeding practices on infant nutritional status-a cross sectional study in the south-west region of Bangladesh. The Pan African Medical Journal. 2013;16.	Title and abstract not relevant- excluded
591.	Islam MA, Khan MNA, Raihan H, Barna SD. Exploring the Influencing Factors for Contraceptive Use among Women: A Meta-Analysis of Demographic and Health Survey Data from 18 Developing Countries. International Journal of Reproductive Medicine. 2022;2022(1):6942438.	Title and abstract not relevant- excluded
592.	Islam MA, Rahman MM, Mahalanabis D. Maternal and socioeconomic factors and the risk of severe malnutrition in a child: a case-control study. European journal of clinical nutrition. 1994;48(6):416-24.	Title and abstract not relevant- excluded
593.	Islam MS, Rahman QS-u, Hossain T, Connor NE, Hossain B, Rahman MM, et al. Using text messages for critical real-time data capture in the ANISA study. The Pediatric Infectious Disease Journal. 2016;35(5):S35-S8.	Title and abstract not relevant- excluded
594.	Islam MZ, Shamim AA, Ahmed A, Akhtaruzzaman M, Kärkkäinen M, Lamberg-Allardt C. Effect of vitamin D, calcium and multiple micronutrients supplementation on lipid profile in pre-menopausal Bangladeshi garment factory workers with hypovitaminosis D. Journal of health, population, and nutrition. 2014;32(4):687.	Title and abstract not relevant- excluded
595.	Islam MZ, Shamim AA, Kemi V, Nevanlinna A, Akhtaruzzaman M, Laaksonen M, et al. Vitamin D deficiency and low bone status in adult female garment factory workers in Bangladesh. British Journal of Nutrition. 2008;99(6):1322-9.	Title and abstract not relevant- excluded
596.	Islam S, Jubayer A, Nayan MM, Islam MH, Nowar A. Assessment of nutrient adequacy and associated factors among lactating women of rural Bangladesh using observed intake: Findings from Bangladesh integrated household survey 2018–2019. Food Science & Nutrition. 2023;11(1):126-36.	Title and abstract not relevant- excluded
597.	Jahan Y, Rahman S, Shamsi T, Sm-Rahman A. Attitudes and views concerning human milk banking among mothers residing in a rural region of Bangladesh. Journal of Human Lactation. 2022;38(1):108-17.	Title and abstract not relevant- excluded
598.	Jahir T, Winch PJ, Leontsini E, Hwang ST, Yeasmin F, Hossain K, et al. Success factors for community health workers in implementing an integrated group-based child development intervention in rural Bangladesh. International journal of environmental research and public health. 2021;18(15):7891.	Title and abstract not relevant- excluded
599.	Jannat K, Luby SP, Unicomb L, Rahman M, Winch PJ, Parvez SM, et al. Complementary feeding practices among rural Bangladeshi mothers: Results from WASH Benefits study. Maternal & Child Nutrition. 2019;15(1):e12654.	Title and abstract not relevant- excluded

SL	Articles excluded during title and abstract screening	Decision
600.	Jeemon P, Mini G, Thankappan K, Harikrishnan S, Sylaja P. GBD 2017 SDG Collaborators. Measuring progress from 1990 to 2017 and projecting attainment to 2030 of the health-related Sustainable Development Goals for 195 countries and territories: a systematic analysis for the Global Burden. 2018.	Title and abstract not relevant- excluded
601.	Jenkins C, Rahman H. Rapidly changing conditions in the brothels of Bangladesh: impact on HIV/STD. AIDS Education and Prevention. 2002;14(3 Supplement):97-106.	Title and abstract not relevant- excluded
602.	Jeong J-H, Korsiak J, Papp E, Shi J, Gernand AD, Al Mahmud A, et al. Determinants of Vitamin D Status of Women of Reproductive Age in Dhaka, Bangladesh: Insights from Husband–Wife Comparisons. Current developments in nutrition. 2019;3(11):nzz112.	Title and abstract not relevant- excluded
603.	Jewkes R, Fulu E, Tabassam Naved R, Chirwa E, Dunkle K, Haardörfer R, et al. Women's and men's reports of past-year prevalence of intimate partner violence and rape and women's risk factors for intimate partner violence: A multicountry cross-sectional study in Asia and the Pacific. PLoS medicine. 2017;14(9):e1002381.	Title and abstract not relevant- excluded
604.	John JR, Mistry SK, Kebede G, Manohar N, Arora A. Determinants of early initiation of breastfeeding in Ethiopia: a population-based study using the 2016 demographic and health survey data. BMC pregnancy and childbirth. 2019;19:1-10.	Title and abstract not relevant- excluded
605.	Kabir A, Merrill RD, Shamim AA, Klemn RD, Labrique AB, Christian P, et al. Canonical correlation analysis of infant's size at birth and maternal factors: a study in rural Northwest Bangladesh. PloS one. 2014;9(4):e94243.	Title and abstract not relevant- excluded
606.	Kabir M. Breastfeeding supplements in urban and rural areas of Bangladesh. Rural demography. 1986;13(1-2):1-11.	Title and abstract not relevant- excluded
607.	Kabir MA, Rahman MM, Khan MN. Maternal anemia and risk of adverse maternal health and birth outcomes in Bangladesh: A nationwide population-based survey. PloS one. 2022;17(12):e0277654.	Title and abstract not relevant- excluded
608.	Kabwijamu L, Waiswa P, Kawooya V, Nalwadda CK, Okuga M, Nabiwemba EL. Newborn care practices among adolescent mothers in Hoima District, Western Uganda. PloS one. 2016;11(11):e0166405.	Title and abstract not relevant- excluded
609.	Kac G, Arnold CD, Matias SL, Mridha MK, Dewey KG. Gestational weight gain and newborn anthropometric outcomes in rural Bangladesh. Maternal & Child Nutrition. 2019;15(4):e12816.	Title and abstract not relevant- excluded
610.	Kadiyala S, Prost A, Harris-Fry H, O'Hearn M, Pradhan R, Pradhan S, et al. Upscaling Participatory Action and Videos for Agriculture and Nutrition (UPAVAN) trial comparing three variants of a nutrition-sensitive agricultural extension intervention to improve maternal and child nutritional outcomes in rural Odisha, India: study protocol for a cluster randomised controlled trial. Trials. 2018;19:1-16.	Title and abstract not relevant- excluded
611.	Kalra S, Ghosh S, Aamir A, Ahmed MT, Amin MF, Bajaj S, et al. Safe and pragmatic use of sodium–glucose co-transporter 2 inhibitors in type 2 diabetes mellitus: South Asian Federation of Endocrine Societies consensus statement. Indian journal of endocrinology and metabolism. 2017;21(1):210-30.	Title and abstract not relevant- excluded
612.	Kampouri M, Tofail F, Rahman SM, Gustin K, Vahter M, Kippler M. Gestational and childhood urinary iodine concentrations and children's cognitive function in a longitudinal mother-child cohort in rural Bangladesh. International Journal of Epidemiology. 2023;52(1):144-55.	Title and abstract not relevant- excluded

SL	Articles excluded during title and abstract screening	Decision
613.	Kamruzzaman M, Rabbani MG, Saw A, Sayem MA, Hossain MG. Differentials in the prevalence of anemia among non-pregnant, ever-married women in Bangladesh: multilevel logistic regression analysis of data from the 2011 Bangladesh Demographic and Health Survey. BMC women's health. 2015;15:1-8.	Title and abstract not relevant- excluded
614.	Kang Y, Prihartono I, Hossain MI, Min S, Kim H, Cho Y, et al. Impact evaluation of a community nutrition and livelihood program on child nutrition in rural Bangladesh. Maternal & Child Nutrition. 2023;19(2):e13461.	Title and abstract not relevant- excluded
615.	Kanungsukkasem U, Ng N, Van Minh H, Razzaque A, Ashraf A, Juvekar S, et al. Fruit and vegetable consumption in rural adults population in INDEPTH HDSS sites in Asia. Global Health Action. 2009;2(1):1988.	Title and abstract not relevant- excluded
616.	Karasz A, Patel V, Ranasinghe S, Chaudhuri K, McKee D. Preventing caries in young children of immigrant Bangladeshi families in New York: perspectives of mothers and paediatricians. Community Dent Health. 2014;31(2):80-4.	Title and abstract not relevant- excluded
617.	Karmaker S, Lahiry S, Roy D, Singha B. Determinants of Infant And Child Mortality in Bangladesh: Time Trends and Comparisons across South Asia. Bangladesh Journal of Medical Science. 2014;13(4).	Title and abstract not relevant- excluded
618.	Karyadi E, Reddy J, Dearden KA, Purwanti T, Mardewi, Asri E, et al. Antenatal care is associated with adherence to iron supplementation among pregnant women in selected low-middle-income-countries of Asia, Africa, and Latin America & the Caribbean regions: Insights from Demographic and Health Surveys. Maternal & child nutrition. 2023;19(2):e13477.	Title and abstract not relevant- excluded
619.	Kasasa S, Natukwatsa D, Galiwango E, Nareeba T, Gyezaho C, Fisker AB, et al. Birth, stillbirth and death registration data completeness, quality and utility in population-based surveys: EN-INDEPTH study. Population Health Metrics. 2021;19:1-15.	Title and abstract not relevant- excluded
620.	Kashi B, Godin CM, Kurzawa ZA, Verney AM, Busch-Hallen JF, De-Regil LM. Multiple micronutrient supplements are more cost-effective than iron and folic acid: modeling results from 3 high-burden Asian countries. The Journal of Nutrition. 2019;149(7):1222-9.	Title and abstract not relevant- excluded
621.	Katoch OR. Determinants of malnutrition among children: A systematic review. Nutrition. 2022;96:111565.	Title and abstract not relevant- excluded
622.	Kc A, Peven K, Ameen S, Msemo G, Basnet O, Ruysen H, et al. Neonatal resuscitation: EN-BIRTH multi-country validation study. BMC Pregnancy and Childbirth. 2021;21:1-19.	Title and abstract not relevant- excluded
623.	Keller S. Good Reproductive health involves many services. Network (Research Triangle Park, NC). 1995;16(1):19-22.	Title and abstract not relevant- excluded
624.	Kerac M, Mwangome M, McGrath M, Haider R, Berkley JA. Management of acute malnutrition in infants aged under 6 months (MAMI): current issues and future directions in policy and research. Food and nutrition bulletin. 2015;36(1_suppl1):S30-S4.	Title and abstract not relevant- excluded
625.	Khan ANS, Karim F, Chowdhury MAK, Zaka N, Manu A, El Arifeen S, et al. Competence of healthcare professionals in diagnosing and managing obstetric complications and conducting neonatal care: a clinical vignette-based assessment in district and subdistrict hospitals in northern Bangladesh. BMJ open. 2019;9(8):e028670.	Title and abstract not relevant- excluded

SL	Articles excluded during title and abstract screening	Decision
626.	Khan GN, Ariff S, Khan U, Habib A, Umer M, Suhag Z, et al. Determinants of infant and young child feeding practices by mothers in two rural districts of Sindh, Pakistan: a cross-sectional survey. International breastfeeding journal. 2017;12:1-8.	Title and abstract not relevant- excluded
627.	Khan NUZ, Rasheed S, Sharmin T, Siddique A, Dibley M, Alam A. How can mobile phones be used to improve nutrition service delivery in rural Bangladesh? BMC health services research. 2018;18:1-10.	Title and abstract not relevant- excluded
628.	Khan SM, Speizer IS, Singh K, Angeles G, Twum–Danso NA, Barker P. Does postnatal care have a role in improving newborn feeding? A study in 15 sub–Saharan African countries. Journal of Global Health. 2017;7(2).	Title and abstract not relevant- excluded
629.	Khanam F, Hossain B, Mistry SK, Mitra DK, Raza WA, Rifat M, et al. The association between daily 500 mg calcium supplementation and lower pregnancy-induced hypertension risk in Bangladesh. BMC pregnancy and childbirth. 2018;18:1-9.	Title and abstract not relevant- excluded
630.	Khanam M, Ara G, Rahman AS, Islam Z, Farhad S, Khan SS, et al. Factors affecting food security in women enrolled in a program for vulnerable group development. Current Developments in Nutrition. 2020;4(4):nzaa037.	Title and abstract not relevant- excluded
631.	Kim ET, Singh K. The state of essential newborn care by delivery location in Bangladesh. Maternal and child health journal. 2017;21:2078-85.	Title and abstract not relevant- excluded
632.	Kim JM, Labrique A, West KP, Rashid M, Shamim AA, Ali H, et al. Maternal morbidity in early pregnancy in rural northern Bangladesh. International Journal of Gynecology & Obstetrics. 2012;119(3):227-33.	Title and abstract not relevant- excluded
633.	Kim R, Mejía-Guevara I, Corsi DJ, Aguayo VM, Subramanian S. Relative importance of 13 correlates of child stunting in South Asia: Insights from nationally representative data from Afghanistan, Bangladesh, India, Nepal, and Pakistan. Social Science & Medicine. 2017;187:144-54.	Title and abstract not relevant- excluded
634.	Kim SS, Roopnaraine T, Nguyen PH, Saha KK, Bhuiyan MI, Menon P. Factors influencing the uptake of a mass media intervention to improve child feeding in Bangladesh. Maternal & child nutrition. 2018;14(3):e12603.	Title and abstract not relevant- excluded
635.	Kinyoki D, Osgood-Zimmerman AE, Bhattacharjee NV, Kassebaum NJ, Hay SI. Anemia prevalence in women of reproductive age in low-and middle-income countries between 2000 and 2018. Nature medicine. 2021;27(10):1761-82.	Title and abstract not relevant- excluded
636.	Kippler M, Tofail F, Hamadani JD, Gardner RM, Grantham-McGregor SM, Bottai M, et al. Early-life cadmium exposure and child development in 5-year-old girls and boys: a cohort study in rural Bangladesh. Environmental health perspectives. 2012;120(10):1462-8.	Title and abstract not relevant- excluded
637.	Kjolhede CL, Stallings RY, Dibley MJ, Sadjimin T, Dawiesah S, Padmawati S. Serum retinol levels among preschool children in Central Java: demographic and socioeconomic determinants. International journal of epidemiology. 1995;24(2):399-403.	Title and abstract not relevant- excluded
638.	Kmush BL, Labrique A, Li W, Klein SL, Schulze K, Shaikh S, et al. The association of cytokines and micronutrients with hepatitis E virus infection during pregnancy and the postpartum period in rural Bangladesh. The American journal of tropical medicine and hygiene. 2016;94(1):203.	Title and abstract not relevant- excluded

SL	Articles excluded during title and abstract screening	Decision
639.	Kouyaté RA, Ahmed S, Haver J, McKaig C, Akter N, Nash-Mercado A, et al. Transition from the Lactational Amenorrhea Method to other modern family planning methods in rural Bangladesh: Barrier analysis and implications for behavior change communication program intervention design. Evaluation and Program Planning. 2015;50:10-7.	Title and abstract not relevant- excluded
640.	Kozuki N, Katz J, Christian P, Lee AC, Liu L, Silveira MF, et al. Comparison of US birth weight references and the international fetal and newborn growth consortium for the 21st century standard. JAMA pediatrics. 2015;169(7):e151438-e.	Title and abstract not relevant- excluded
641.	Kram N, Melgen S, Kedera E, Collison DK, Colton J, Blount W, et al. The acceptability of dietary tools to improve maternal and child nutrition in Western Kenya. Public Health Nutrition. 2016;19(10):1823-33.	Title and abstract not relevant- excluded
642.	Kramer E, Peterson K, Rogers B, Hughes M. Intrahousehold allocation of energy intake among children under five years and their parents in rural Bangladesh. European journal of clinical nutrition. 1997;51(11):750-6.	Title and abstract not relevant- excluded
643.	Kramer MS, Kakuma R. Optimal duration of exclusive breastfeeding. Cochrane database of systematic reviews. 2012(8).	Title and abstract not relevant- excluded
644.	Kumar A, Rajpal S, Alambusha R, Sharma S, Joe W. Can Anganwadi services strengthening improve the association between maternal and child dietary diversity? Evidence from Project Spotlight implemented in tribal dominated Gadchiroli and Chandrapur districts of Maharashtra, India. Plos one. 2022;17(3):e0264567.	Title and abstract not relevant- excluded
645.	Kutlu R, Kara F, Durduran Y, Marakoglu K, Çivi S. Assessment of effects of pre-and post-training programme for healthcare professionals about breastfeeding. Journal of health, population, and nutrition. 2007;25(3):382.	Title and abstract not relevant- excluded
646.	Kwesiga D, Tawiah C, Imam MA, Tesega AK, Nareeba T, Enuameh YA, et al. Barriers and enablers to reporting pregnancy and adverse pregnancy outcomes in population-based surveys: EN-INDEPTH study. Population health metrics. 2021;19:1-14.	Title and abstract not relevant- excluded
647.	Kyu HH, Abate D, Abate KH, Abay SM, Abbafati C, Abbasi N, et al. Global, regional, and national disability-adjusted life-years (DALYs) for 359 diseases and injuries and healthy life expectancy (HALE) for 195 countries and territories, 1990–2017: a systematic analysis for the Global Burden of Disease Study 2017. The Lancet. 2018;392(10159):1859-922.	Title and abstract not relevant- excluded
648.	Labrique A, Sikder S, Wu L, Rashid M, Ali H, Ullah B, et al. Beyond pregnancy–the neglected burden of mortality in young women of reproductive age in Bangladesh: a prospective cohort study. BJOG: An International Journal of Obstetrics & Gynaecology. 2013;120(9):1085-9.	Title and abstract not relevant- excluded
649.	Labrique AB, Christian P, Klemm RD, Rashid M, Shamim AA, Massie A, et al. A cluster-randomized, placebo-controlled, maternal vitamin A or beta-carotene supplementation trial in Bangladesh: design and methods. Trials. 2011;12:1-18.	Title and abstract not relevant- excluded
650.	Labrique AB, Palmer AC, Healy K, Mehra S, Sauer TC, West KP, et al. A novel device for assessing dark adaptation in field settings. BMC ophthalmology. 2015;15:1-9.	Title and abstract not relevant- excluded

SL	Articles excluded during title and abstract screening	Decision
651.	Labrique AB, Pereira S, Christian P, Murthy N, Bartlett L, Mehl G. Pregnancy registration systems can enhance health systems, increase accountability and reduce mortality. Reproductive health matters. 2012;20(39):113-7.	Title and abstract not relevant- excluded
652.	Lama TP, Khatry SK, Katz J, LeClerq SC, Mullany LC. Illness recognition, decision-making, and care-seeking for maternal and newborn complications: a qualitative study in Sarlahi District, Nepal. Journal of Health, Population and Nutrition. 2017;36:45-58.	Title and abstract not relevant- excluded
653.	Lamstein SA. Differential impact of a community-based nutrition program on the poor and the non-poor: analysis from the Bangladesh Integrated Nutrition Project. (No Title).	Title and abstract not relevant- excluded
654.	Lazarus JV, Romero D, Kopka CJ, Karim SA, Abu-Raddad LJ, Almeida G, et al. A multinational Delphi consensus to end the COVID-19 public health threat. Nature. 2022;611(7935):332-45.	Title and abstract not relevant- excluded
655.	Lee C-F, Amin R. Socioeconomic factors, intermediate variables and fertility in Bangladesh. Journal of Biosocial Science. 1981;13(2):179-88.	Title and abstract not relevant- excluded
656.	Leung DT, Das SK, Malek M, Qadri F, Faruque A, Chisti MJ, et al. Concurrent pneumonia in children under 5 years of age presenting to a diarrheal hospital in Dhaka, Bangladesh. The American Journal of Tropical Medicine and Hygiene. 2015;93(4):831.	Title and abstract not relevant- excluded
657.	Levay AV, Mumtaz Z, Faiz Rashid S, Willows N. Influence of gender roles and rising food prices on poor, pregnant women’s eating and food provisioning practices in Dhaka, Bangladesh. Reproductive Health. 2013;10:1-11.	Title and abstract not relevant- excluded
658.	Lindsey LL. Sharp right turn: Globalization and gender equity. The Sociological Quarterly. 2014;55(1):1-22.	Title and abstract not relevant- excluded
659.	Liu E, Wang D, Darling AM, Perumal N, Wang M, Ahmed T, et al. Effects of prenatal nutritional supplements on gestational weight gain in low-and middle-income countries: a meta-analysis of individual participant data. The American journal of clinical nutrition. 2022;116(6):1864-76.	Title and abstract not relevant- excluded
660.	Lozano R, Fullman N, Mumford JE, Knight M, Barthelemy CM, Abbafati C, et al. Measuring universal health coverage based on an index of effective coverage of health services in 204 countries and territories, 1990–2019: a systematic analysis for the Global Burden of Disease Study 2019. The Lancet. 2020;396(10258):1250-84.	Title and abstract not relevant- excluded
661.	Ly C, Diallo A, Simondon F, Simondon K. Early short-term infant food supplementation, maternal weight loss and duration of breast-feeding: a randomised controlled trial in rural Senegal. European journal of clinical nutrition. 2006;60(2):265-71.	Title and abstract not relevant- excluded
662.	Lyngdoh T, Neogi SB, Ahmad D, Soundararajan S, Mavalankar D. Intensity of contact with frontline workers and its influence on maternal and newborn health behaviors: cross-sectional survey in rural Uttar Pradesh, India. Journal of Health, Population and Nutrition. 2018;37:1-11.	Title and abstract not relevant- excluded
663.	Makasi RR, Humphrey JH. Summarizing the child growth and diarrhea findings of the water, sanitation, and hygiene benefits and sanitation hygiene infant nutrition efficacy trials. Global Landscape of Nutrition Challenges in Infants and Children. 2020;93:153-66.	Title and abstract not relevant- excluded

SL	Articles excluded during title and abstract screening	Decision
664.	Mallard SR, Houghton LA, Filteau S, Mullen A, Nieuwelink J, Chisenga M, et al. Dietary diversity at 6 months of age is associated with subsequent growth and mediates the effect of maternal education on infant growth in urban Zambia. The Journal of nutrition. 2014;144(11):1818-25.	Title and abstract not relevant- excluded
665.	Manikam L, Allaham S, Demel IC, Bello UA, Naman M, Heys M, et al. Developing a community facilitator-led participatory learning and action women's group intervention to improve infant feeding, care and dental hygiene practices in South Asian infants: NEON programme. Health Expectations. 2022;25(5):2416-30.	Title and abstract not relevant- excluded
666.	Mannan HR, Islam MN. Breast-feeding in Bangladesh: patterns and impact on fertility. Asia-Pacific Population Journal. 1995;10(4):23-38.	Title and abstract not relevant- excluded
667.	Mannan T, Ahmed S, Akhtar E, Roy AK, Haq MA, Roy A, et al. Maternal micronutrient supplementation and long term health impact in children in rural Bangladesh. PLoS One. 2016;11(8):e0161294.	Title and abstract not relevant- excluded
668.	Manzione LC, Kriser H, Gamboa EG, Hanson CM, Mulokozi G, Mwaipape O, et al. Maternal employment status and minimum meal frequency in children 6-23 months in Tanzania. International journal of environmental research and public health. 2019;16(7):1137.	Title and abstract not relevant- excluded
669.	Marjan N, Rahman A, Rois R, Rahman A. Factors associated with coverage of vitamin a supplementation among Bangladeshi children: mixed modelling approach. BMC Public Health. 2021;21:1-11.	Title and abstract not relevant- excluded
670.	Marriott H. In-depth study of breastfeeding structure: New data from Mali. American Journal of Human Biology: The Official Journal of the Human Biology Association. 1998;10(2):179-90.	Title and abstract not relevant- excluded
671.	Masuku SK, Lan S-JJ. Nutritional knowledge, attitude, and practices among pregnant and lactating women living with HIV in the Manzini region of Swaziland. Journal of health, population, and nutrition. 2014;32(2):261.	Title and abstract not relevant- excluded
672.	Matias SL, Mridha MK, Paul RR, Hussain S, Vosti SA, Arnold CD, et al. Prenatal lipid-based nutrient supplements affect maternal anthropometric indicators only in certain subgroups of rural Bangladeshi women. The Journal of nutrition. 2016;146(9):1775-82.	Title and abstract not relevant- excluded
673.	Matias SL, Mridha MK, Young RT, Hussain S, Dewey KG. Daily maternal lipid-based nutrient supplementation with 20 mg iron, compared with iron and folic acid with 60 mg iron, resulted in lower iron status in late pregnancy but not at 6 months postpartum in either the mothers or their infants in Bangladesh. The Journal of Nutrition. 2018;148(10):1615-24.	Title and abstract not relevant- excluded
674.	Mazur NI, Löwensteyn YN, Willemsen JE, Gill CJ, Forman L, Mwananyanda LM, et al. Global respiratory syncytial virus–related infant community deaths. Clinical Infectious Diseases. 2021;73(Supplement_3):S229-S37.	Title and abstract not relevant- excluded
675.	McClure EM, Garces AL, Hibberd PL, Moore JL, Goudar SS, Saleem S, et al. The Global Network Maternal Newborn Health Registry: a multi-country, community-based registry of pregnancy outcomes. Reproductive health. 2020;17:1-11.	Title and abstract not relevant- excluded
676.	McCormick BJ, Caulfield LE, Richard SA, Pendergast L, Seidman JC, Maphula A, et al. Early life experiences and trajectories of cognitive development. Pediatrics. 2020;146(3).	Title and abstract not relevant- excluded

SL	Articles excluded during title and abstract screening	Decision
677.	McCormick BJ, Richard SA, Caulfield LE, Pendergast LL, Seidman JC, Koshy B, et al. Early life child micronutrient status, maternal reasoning, and a nurturing household environment have persistent influences on child cognitive development at age 5 years: results from MAL-ED. The Journal of nutrition. 2019;149(8):1460-9.	Title and abstract not relevant- excluded
678.	McFadden A, Renfrew MJ, Atkin K. Using qualitative research findings to analyse how breastfeeding public health recommendations can be tailored to meet the needs of women of Bangladeshi origin living in England. Journal of Research in Nursing. 2012;17(2):159-78.	Title and abstract not relevant- excluded
679.	McNamara K, Wood E. Food taboos, health beliefs, and gender: understanding household food choice and nutrition in rural Tajikistan. Journal of Health, Population and Nutrition. 2019;38(1):17.	Title and abstract not relevant- excluded
680.	Mena Alberico AP, Valeria da Veiga G, Ribeiro Baião M, Antonieta de Souza Santos MM, Buongermino de Souza S, Cornbluth Szarfarc S. Iron deficiency anaemia in infants attended at municipal primary health care centres in Rio de Janeiro–Brazil. Nutrition & Food Science. 2003;33(2):50-5.	Title and abstract not relevant- excluded
681.	Menon P, Nguyen PH, Saha KK, Khaled A, Kennedy A, Tran LM, et al. Impacts on breastfeeding practices of at-scale strategies that combine intensive interpersonal counseling, mass media, and community mobilization: results of cluster-randomized program evaluations in Bangladesh and Viet Nam. PLoS medicine. 2016;13(10):e1002159.	Title and abstract not relevant- excluded
682.	Miller S, Belizán JM. The true cost of maternal death: individual tragedy impacts family, community and nations. Reproductive health. 2015;12:1-4.	Title and abstract not relevant- excluded
683.	Mistry SK, Hossain MB, Arora A. Maternal nutrition counselling is associated with reduced stunting prevalence and improved feeding practices in early childhood: a post-program comparison study. Nutrition Journal. 2019;18:1-9.	Title and abstract not relevant- excluded
684.	Mitchell S, Cockcroft A, Andersson N. Population weighted raster maps can communicate findings of social audits: examples from three continents. BMC health services research. 2011;11:1-21.	Title and abstract not relevant- excluded
685.	Mitra DK, Mahmud A, Begum N, Rafiqullah I, Roy A, Moin SMI, et al. Implementation of the ANISA protocol in Sylhet, Bangladesh: Challenges and Solutions. The Pediatric Infectious Disease Journal. 2016;35(5):S55-S9.	Title and abstract not relevant- excluded
686.	Mitu MMP, Islam K, Sarwar S, Ali M, Amin MR. Spatial differences in diet quality and economic vulnerability to food insecurity in Bangladesh: results from the 2016 household income and expenditure survey. Sustainability. 2022;14(9):5643.	Title and abstract not relevant- excluded
687.	Mohite R, Mohite V, Kakade S. Knowledge of breast feeding among primigravida mothers. Bangladesh Journal of Medical Science. 2012;11(4):312.	Title and abstract not relevant- excluded
688.	Mohite VR, Pratinidhi AK, Mohite RV. Reproductive risk factors and breast cancer: a case control study from rural India. Bangladesh Journal of Medical Science. 2015;14(3):258.	Title and abstract not relevant- excluded
689.	Monterrosa EC, Beesabathuni K, van Zutphen KG, Steiger G, Kupka R, Fleet A, et al. Situation analysis of procurement and production of multiple micronutrient supplements in 12 lower and upper middle-income countries. Maternal & Child Nutrition. 2018;14:e12500.	Title and abstract not relevant- excluded

SL	Articles excluded during title and abstract screening	Decision
690.	Alauddin M. Maternal mortality in rural Bangladesh: the Tangail district. Studies in family planning. 1986;17(1):13-21.	Title and abstract not relevant- excluded
691.	Jafree SR, Momina A, Muazzam A, Wajid R, Calib G. Factors affecting delivery health service satisfaction of women and fear of COVID− 19: implications for maternal and child health in Pakistan. Maternal and child health journal. 2021;25(6):881-91.	Title and abstract not relevant- excluded
692.	Bhan G, Bhandari N, Taneja S, Mazumder S, Bahl R, Group ZS. The effect of maternal education on gender bias in care-seeking for common childhood illnesses. Social science & medicine. 2005;60(4):715-24.	Title and abstract not relevant- excluded
693.	Avula R. Understanding Maternal Demands, Capabilities, and Ability to Feed Children in Bangladesh. 2011.	Title and abstract not relevant- excluded
694.	Rana EA, Fazal MA, Alim MA. Frequently used therapeutic antimicrobials and their resistance patterns on Staphylococcus aureus and Escherichia coli in mastitis affected lactating cows. International Journal of Veterinary Science and Medicine. 2022;10(1):1-10.	Title and abstract not relevant- excluded
695.	Kurian K, Lakiang T, Sinha RK, Kathuria N, Krishnan P, Mehra D, et al. Scoping review of intervention strategies for improving coverage and uptake of maternal nutrition services in Southeast Asia. International Journal of Environmental Research and Public Health. 2021;18(24):13292.	Title and abstract not relevant- excluded
696.	Akter T, Dawson A, Sibbritt D. The determinants of essential newborn care for home births in Bangladesh. Public health. 2016;141:7-16.	Title and abstract not relevant- excluded
697.	Dibley M, Thow AM, Devkota M, Gaidhane A, Godakandage S, Hazir T, et al. Opportunities for strengthening infant and young child feeding policies in South Asia: Insights from the SAIFRN policy analysis project. 2017.	Title and abstract not relevant- excluded
698.	Marriott BP, White A, Hadden L, Davies JC, Wallingford JC. World Health Organization (WHO) infant and young child feeding indicators: associations with growth measures in 14 low-income countries. Maternal & child nutrition. 2012;8(3):354-70.	Title and abstract not relevant- excluded
699.	Rahman MM, Islam MA, Mahalanabis D, Chowdhury S, Biswas E. Impact of health education on the feeding of green leafy vegetables at home to children of the urban poor mothers of Bangladesh. Public Health. 1994;108(3):211-8.	Title and abstract not relevant- excluded
700.	Athavale P, Hoeft K, Dalal RM, Bondre AP, Mukherjee P, Sokal-Gutierrez K. A qualitative assessment of barriers and facilitators to implementing recommended infant nutrition practices in Mumbai, India. Journal of Health, Population and Nutrition. 2020;39:1-12.	Title and abstract not relevant- excluded
701.	Na M, Shamim AA, Mehra S, Labrique A, Ali H, Wu LS-F, et al. Maternal nutritional status mediates the linkage between household food insecurity and mid-infancy size in rural Bangladesh. British Journal of Nutrition. 2020;123(12):1415-25.	Title and abstract not relevant- excluded
702.	Naila NN, Mahfuz M, Hossain M, Arndt M, Walson JL, Nahar B, et al. Improvement in appetite among stunted children receiving nutritional intervention in Bangladesh: results from a community-based study. European journal of clinical nutrition. 2021;75(9):1359-67.	Title and abstract not relevant- excluded

SL	Articles excluded during title and abstract screening	Decision
703.	Nareeba T, Dzabeng F, Alam N, Biks GA, Thysen SM, Akuze J, et al. Neonatal and child mortality data in retrospective population-based surveys compared with prospective demographic surveillance: EN-INDEPTH study. Population health metrics. 2021;19:1-17.	Title and abstract not relevant- excluded
704.	Nath DC, Singh KK, Land KC, Talukdar PK. Breastfeeding and postpartum amenorrhea in a traditional society: a hazards model analysis. Social biology. 1993;40(1-2):74-86.	Title and abstract not relevant- excluded
705.	Nayak U, Kanungo S, Zhang D, Ross Colgate E, Carmolli MP, Dey A, et al. Influence of maternal and socioeconomic factors on breast milk fatty acid composition in urban, low-income families. Maternal & child nutrition. 2017;13(4):e12423.	Title and abstract not relevant- excluded
706.	Neupane D, McLachlan CS, Sharma R, Gyawali B, Khanal V, Mishra SR, et al. Prevalence of hypertension in member countries of South Asian Association for Regional Cooperation (SAARC): systematic review and meta-analysis. Medicine. 2014;93(13):e74.	Title and abstract not relevant- excluded
707.	Ng N, Hakimi M, Van Minh H, Juvekar S, Razzaque A, Ashraf A, et al. Prevalence of physical inactivity in nine rural INDEPTH Health and Demographic Surveillance Systems in five Asian countries. Global health action. 2009;2(1):1985.	Title and abstract not relevant- excluded
708.	Ng N, Kowal P, Kahn K, Naidoo N, Abdullah S, Bawah A, et al. Health inequalities among older men and women in Africa and Asia: evidence from eight Health and Demographic Surveillance System sites in the INDEPTH WHO-SAGE Study. Global Health Action. 2010;3(1):5420.	Title and abstract not relevant- excluded
709.	Nguyen PH, Frongillo EA, Kim SS, Zongrone AA, Jilani A, Tran LM, et al. Information diffusion and social norms are associated with infant and young child feeding practices in Bangladesh. The Journal of Nutrition. 2019;149(11):2034-45.	Title and abstract not relevant- excluded
710.	Nguyen PH, Frongillo EA, Sanghvi T, Wable G, Mahmud Z, Tran LM, et al. Engagement of husbands in a maternal nutrition program substantially contributed to greater intake of micronutrient supplements and dietary diversity during pregnancy: results of a cluster-randomized program evaluation in Bangladesh. The Journal of nutrition. 2018;148(8):1352-63.	Title and abstract not relevant- excluded
711.	Nguyen PH, Headey D, Frongillo EA, Tran LM, Rawat R, Ruel MT, et al. Changes in underlying determinants explain rapid increases in child linear growth in Alive & Thrive study areas between 2010 and 2014 in Bangladesh and Vietnam. The Journal of Nutrition. 2017;147(3):462-9.	Title and abstract not relevant- excluded
712.	Nguyen PH, Kim SS, Sanghvi T, Mahmud Z, Tran LM, Shabnam S, et al. Integrating nutrition interventions into an existing maternal, neonatal, and child health program increased maternal dietary diversity, micronutrient intake, and exclusive breastfeeding practices in Bangladesh: results of a cluster-randomized program evaluation. The Journal of Nutrition. 2017;147(12):2326-37.	Title and abstract not relevant- excluded
713.	Nguyen PH, Kim SS, Tran LM, Menon P, Frongillo EA. Intervention design elements are associated with frontline health workers' performance to deliver infant and young child nutrition services in Bangladesh and Vietnam. Current developments in nutrition. 2019;3(8):nzz070.	Title and abstract not relevant- excluded
714.	Nguyen VH, Mouquet-Rivier C, Eymard-Duvernay S, Treche S. Effect of extrusion cooking and amylase addition to gruels to increase energy density and nutrient intakes by Vietnamese infants. Asia Pacific Journal of Clinical Nutrition. 2010;19(3):308-15.	Title and abstract not relevant- excluded

SL	Articles excluded during title and abstract screening	Decision
715.	Niger MT, Shafia Khatun SK, Marufa Sultana MS, Nurul Islam NI, Ohtani Kazuhiro OK. Determinants of malnutrition among the children under 2 years of age. 2010.	Title and abstract not relevant- excluded
716.	Niraula K, Kohrt BA, Flora MS, Thapa N, Mumu SJ, Pathak R, et al. Prevalence of depression and associated risk factors among persons with type-2 diabetes mellitus without a prior psychiatric history: a cross-sectional study in clinical settings in urban Nepal. BMC psychiatry. 2013;13:1-12.	Title and abstract not relevant- excluded
717.	Nisha MK, Raynes-Greenow C, Rahman A, Alam A. Perceptions and practices related to birthweight in rural Bangladesh: implications for neonatal health programs in low-and middle-income settings. PloS one. 2019;14(12):e0221691.	Title and abstract not relevant- excluded
718.	Nugent R, Brower E, Cravioto A, Koehlmoos T. A cost-benefit analysis of a National Hypertension Treatment Program in Bangladesh. Preventive medicine. 2017;105:S56-S61.	Title and abstract not relevant- excluded
719.	NUR O. THE EFFECT OF INFANT MORTALITY ON REPRODUCTIVE BEHAVIOR: THE EXPERIENCE OF SUDAN. 2000.	Title and abstract not relevant- excluded
720.	Nuri RP, Ghahari S, Aldersey HM, Huque AS. Exploring access to government-led support for children with disabilities in Bangladesh. Plos one. 2020;15(7):e0235439.	Title and abstract not relevant- excluded
721.	Oddo VM, Rah JH, Semba RD, Sun K, Akhter N, Sari M, et al. Predictors of maternal and child double burden of malnutrition in rural Indonesia and Bangladesh. The American journal of clinical nutrition. 2012;95(4):951-8.	Title and abstract not relevant- excluded
722.	Ogunlesi TA, Ogunfowora OB, Ogundeyi MM. Prevalence and risk factors for hypothermia on admission in Nigerian babies< 72 h of age. 2009.	Title and abstract not relevant- excluded
723.	Okuga M, Waiswa P, Mandu R, Wachira J, Hanson C, Manzi F. Illness recognition and care-seeking for maternal and newborn complications in rural eastern Uganda. Journal of Health, Population and Nutrition. 2017;36:75-84.	Title and abstract not relevant- excluded
724.	Olney DK, Leroy J, Bliznashka L, Ruel MT. PROCOMIDA, a food-assisted maternal and child health and nutrition program, reduces child stunting in Guatemala: a cluster-randomized controlled intervention trial. The Journal of nutrition. 2018;148(9):1493-505.	Title and abstract not relevant- excluded
725.	Olsen J, Nøhr EA, Thomsen RW, Støvring H. Non-communicable disease epidemic: epidemiology in action (EuroEpi 2013 and NordicEpi 2013) Aarhus, Denmark from 11 August to 14 August 2013. European Journal of Epidemiology. 2013;28:S1-S270.	Title and abstract not relevant- excluded
726.	Orderud H, Härkönen J, Hårsaker CT, Bogren M. Floods and maternal healthcare utilisation in Bangladesh. Population and environment. 2022;44(3):193-225.	Title and abstract not relevant- excluded
727.	Ouyang Y-Q, Nasrin L, editors. Father's knowledge, attitude and support to mother's exclusive breastfeeding practices in Bangladesh: a multi-group structural equations model analysis. Healthcare; 2021: MDPI.	Title and abstract not relevant- excluded
728.	Owais A, Kleinbaum DG, Suchdev PS, Faruque A, Das SK, Schwartz B, et al. Household food security and infant feeding practices in rural Bangladesh. Public health nutrition. 2016;19(10):1875-81.	Title and abstract not relevant- excluded

SL	Articles excluded during title and abstract screening	Decision
729.	Owais A, Schwartz B, Kleinbaum DG, Suchdev PS, Faruque A, Das SK, et al. Minimum acceptable diet at 9 months but not exclusive breastfeeding at 3 months or timely complementary feeding initiation is predictive of infant growth in rural Bangladesh. PLoS One. 2016;11(10):e0165128.	Title and abstract not relevant- excluded
730.	Owais A, Schwartz B, Kleinbaum DG, Suchdev PS, Faruque ASG, Das SK, et al. A nutrition education program in rural Bangladesh was associated with improved feeding practices but not with child growth. The Journal of Nutrition. 2017;147(5):948-54.	Title and abstract not relevant- excluded
731.	Pagel C, Prost A, Lewycka S, Das S, Colbourn T, Mahapatra R, et al. Intracluster correlation coefficients and coefficients of variation for perinatal outcomes from five cluster-randomised controlled trials in low and middle-income countries: results and methodological implications. Trials. 2011;12:1-12.	Title and abstract not relevant- excluded
732.	Pan WK, Seidman JC, Ali A, Hoest C, Mason C, Mondal D, et al. Oral polio vaccine response in the MAL-ED birth cohort study: considerations for polio eradication strategies. Vaccine. 2019;37(2):352-65.	Title and abstract not relevant- excluded
733.	Pasricha S-R, Hasan MI, Braat S, Larson LM, Tipu SM-U, Hossain SJ, et al. Benefits and risks of iron interventions in infants in rural Bangladesh. New England Journal of Medicine. 2021;385(11):982-95.	Title and abstract not relevant- excluded
734.	Patel A, Pusdekar Y, Badhoniya N, Borkar J, Agho KE, Dibley MJ. Determinants of inappropriate complementary feeding practices in young children in India: secondary analysis of National Family Health Survey 2005–2006. Maternal & child nutrition. 2012;8:28-44.	Title and abstract not relevant- excluded
735.	Paulson KR, Kamath AM, Alam T, Bienhoff K, Abady GG, Abbas J, et al. Global, regional, and national progress towards Sustainable Development Goal 3.2 for neonatal and child health: all-cause and cause-specific mortality findings from the Global Burden of Disease Study 2019. The Lancet. 2021;398(10303):870-905.	Title and abstract not relevant- excluded
736.	Penfold S, Manzi F, Mkumbo E, Temu S, Jaribu J, Shamba DD, et al. Effect of home-based counselling on newborn care practices in southern Tanzania one year after implementation: a cluster-randomised controlled trial. BMC pediatrics. 2014;14:1-12.	Title and abstract not relevant- excluded
737.	Perry G. Alloparental care and assistance in a normatively patrilocal society. Current Anthropology. 2017;58(1):114-23.	Title and abstract not relevant- excluded
738.	Perry HB, Chowdhury M, Were M, LeBan K, Crigler L, Lewin S, et al. Community health workers at the dawn of a new era: 11. CHWs leading the way to “Health for All”. Health Research Policy and Systems. 2021;19:1-21.	Title and abstract not relevant- excluded
739.	Pervin J, Gustafsson FE, Moran AC, Roy S, Persson LÅ, Rahman A. Implementing K angaroo mother care in a resource-limited setting in rural B angladesh. Acta Paediatrica. 2015;104(5):458-65.	Title and abstract not relevant- excluded
740.	Peven K, Day LT, Ruysen H, Tahsina T, Kc A, Shabani J, et al. Stillbirths including intrapartum timing: EN-BIRTH multi-country validation study. BMC Pregnancy and Childbirth. 2021;21:1-18.	Title and abstract not relevant- excluded
741.	Pickering AJ, Null C, Winch PJ, Mangwadu G, Arnold BF, Prendergast AJ, et al. The WASH Benefits and SHINE trials: interpretation of WASH intervention effects on linear growth and diarrhoea. The Lancet Global Health. 2019;7(8):e1139-e46.	Title and abstract not relevant- excluded
742.	Piperata BA, Mattern LMG. Longitudinal study of breastfeeding structure and women's work in the Brazilian Amazon. American Journal of Physical Anthropology. 2011;144(2):226-37.	Title and abstract not relevant- excluded

SL	Articles excluded during title and abstract screening	Decision
743.	Pitchik HO, Tofail F, Rahman M, Akter F, Sultana J, Shoab AK, et al. A holistic approach to promoting early child development: a cluster randomised trial of a group-based, multicomponent intervention in rural Bangladesh. BMJ Global Health. 2021;6(3):e004307.	Title and abstract not relevant- excluded
744.	Popkin BM, Guilkey DK, Akin JS, Adair LS, Richard Udry J, Flieger W. Nutrition, lactation, and birth spacing in Filipino women. Demography. 1993;30:333-52.	Title and abstract not relevant- excluded
745.	Qiu L, Binns CW, Zhao Y, Lee AH, Xie X. Breastfeeding practice in Zhejiang province, PR China, in the context of melamine-contaminated formula milk. Journal of health, population, and nutrition. 2010;28(2):189.	Title and abstract not relevant- excluded
746.	Rah JH, Christian P, Shamim AA, Arju UT, Labrique AB, Rashid M. Predictors of stunting and thinness in post-menarcheal adolescent girls in rural Bangladesh. Public health nutrition. 2009;12(12):2400-9.	Title and abstract not relevant- excluded
747.	Rahman A, Akter F. Reasons for formula feeding among rural Bangladeshi mothers: A qualitative exploration. PloS one. 2019;14(2):e0211761.	Title and abstract not relevant- excluded
748.	Rahman A, Bhuiyan MB, Das SK. Effect of short-term educational intervention on complementary feeding index among infants in rural Bangladesh: a randomized control trial. BMC nutrition. 2022;8(1):73.	Title and abstract not relevant- excluded
749.	Rahman M, Chen LC, Chakraborty J, Yunus M, Chowdhury A, Sarder A, et al. Use of tetanus toxoid for the prevention of neonatal tetanus. 1. Reduction of neonatal mortality by immunization of non-pregnant and pregnant women in rural Bangladesh. Bulletin of the World Health Organization. 1982;60(2):261.	Title and abstract not relevant- excluded
750.	Rahman M, Haider MM, Curtis SL, Lance PM. The Mayer Hashi large-scale program to increase use of long-acting reversible contraceptives and permanent methods in Bangladesh: explaining the disappointing results. An outcome and process evaluation. Global Health: Science and Practice. 2016;4(Supplement 2):S122-S39.	Title and abstract not relevant- excluded
751.	Rahman S, Ahmed T, Rahman AS, Alam N, Ahmed AS, Ireen S, et al. Status of zinc nutrition in Bangladesh: the underlying associations. Journal of Nutritional Science. 2016;5:e25.	Title and abstract not relevant- excluded
752.	Rahman S, Shaheen N. Phytate-iron molar ratio and bioavailability of iron in Bangladesh. Tropical Medicine & International Health. 2022;27(5):509-14.	Title and abstract not relevant- excluded
753.	Rahman SR, Islam MN, Harun-ur-Rashid M, Siddiki MSR, Islam MA. Dairy buffalo production under intensive system in semi arid area of Bangladesh. Buffalo Bulletin. 2019;38(1):83-98.	Title and abstract not relevant- excluded
754.	Rajpal S, Kumar A, Alambusha R, Sharma S, Joe W. Maternal dietary diversity during lactation and associated factors in Palghar district, Maharashtra, India. PLoS One. 2021;16(12):e0261700.	Title and abstract not relevant- excluded
755.	Rashid AM, Rashid AS, Rahman A. Prevalence of intestinal parasitoses in urban and rural children of a developing country. Asian Pacific Journal of Tropical Biomedicine. 2011;1(2):S268-S70.	Title and abstract not relevant- excluded
756.	Rashid MB, Marey MA, Fukuda K, Haneda S, Kusama K, Shimada M, et al. Intrauterine infusion of low levels of interferon-tau on day-8 post-estrus stimulates the bovine endometrium to secrete apolipoprotein-A1: A possible implication for early embryo tolerance. American Journal of Reproductive Immunology. 2022;88(3):e13592.	Title and abstract not relevant- excluded
757.	Rasmi A, Purnima M, Islam BM, Saiqa S, Raisul H, SB JC, et al. A program impact pathway analysis identifies critical steps in the implementation and utilization of a behavior change communication	Title and abstract not relevant- excluded

SL	Articles excluded during title and abstract screening	Decision
	intervention promoting infant and child feeding practices in Bangladesh. The Journal of nutrition. 2013;143(12):2029-37.	
758.	Rasul C, Kabir A, Rashid A, Mahboob A, Hassan M. Role of antibiotic in the outcome of bronchiolitis. group. 2008;6:1.	Title and abstract not relevant- excluded
759.	Rauyajin O, Pasandhanatorn V, Rauyajin V, Na-nakorn S, Ngarmyithayapong J, Varothai C. Mothers' hygiene behaviours and their determinants in Suphanburi, Thailand. Journal of Diarrhoeal Diseases Research. 1994:25-34.	Title and abstract not relevant- excluded
760.	Rayment J, McCourt C, Vaughan L, Christie J, Trenchard-Mabere E. B angladeshi women's experiences of infant feeding in the L ondon B orough of T ower H amlets. Maternal & Child Nutrition. 2016;12(3):484-99.	Title and abstract not relevant- excluded
761.	Raza WA, Van de Poel E, Van Ourti T. Impact and spill-over effects of an asset transfer program on child undernutrition: Evidence from a randomized control trial in Bangladesh. Journal of Health Economics. 2018;62:105-20.	Title and abstract not relevant- excluded
762.	Reerink I, Namaste SM, Poonawala A, Nyhus Dhillon C, Aburto N, Chaudhery D, et al. Experiences and lessons learned for delivery of micronutrient powders interventions. Maternal & Child Nutrition. 2017;13:e12495.	Title and abstract not relevant- excluded
763.	Richard SA, McCormick BJ, Murray-Kolb LE, Patil CL, Chandyo RK, Mahopo C, et al. Characteristics associated with the transition to partial breastfeeding prior to 6 months of age: Data from seven sites in a birth cohort study. Maternal & child nutrition. 2021;17(3):e13166.	Title and abstract not relevant- excluded
764.	Roba KT, O'Connor TP, O'Brien NM, Aweke CS, Kahsay ZA, Chisholm N, et al. Seasonal variations in household food insecurity and dietary diversity and their association with maternal and child nutritional status in rural Ethiopia. Food Security. 2019;11:651-64.	Title and abstract not relevant- excluded
765.	Robert RC, Bartolini RM, Creed-Kanashiro HM, Verney Sward A. Using formative research to design context-specific animal source food and multiple micronutrient powder interventions to improve the consumption of micronutrients by infants and young children in Tanzania, Kenya, Bangladesh and Pakistan. Maternal & child nutrition. 2021;17(2):e13084.	Title and abstract not relevant- excluded
766.	Robey B. Policies for fertility reduction: focus on Asia. Asia-Pacific population & policy. 1989(9):1-4.	Title and abstract not relevant- excluded
767.	Rosales A, Sulistyo S, Miko O, Hairani LK, Ilyana M, Thomas J, et al. Recognition of and care-seeking for maternal and newborn complications in Jayawijaya district, Papua province, Indonesia: a qualitative study. Journal of Health, Population and Nutrition. 2017;36:35-44.	Title and abstract not relevant- excluded
768.	Ross JL, Laston SL, Pelto PJ, Muna L. Exploring explanatory models of women's reproductive health in rural Bangladesh. Culture, Health & Sexuality. 2002;4(2):173-90.	Title and abstract not relevant- excluded
769.	Roth DE, Pezzack B, Al Mahmud A, Abrams SA, Islam M, Phillips AA, et al. Bioavailability of enteric-coated microencapsulated calcium during pregnancy: a randomized crossover trial in Bangladesh. The American Journal of Clinical Nutrition. 2014;100(6):1587-95.	Title and abstract not relevant- excluded

SL	Articles excluded during title and abstract screening	Decision
770.	Roth GA, Abate D, Abate KH, Abay SM, Abbafati C, Abbasi N, et al. Global, regional, and national age-sex-specific mortality for 282 causes of death in 195 countries and territories, 1980–2017: a systematic analysis for the Global Burden of Disease Study 2017. The lancet. 2018;392(10159):1736-88.	Title and abstract not relevant- excluded
771.	Roy S, Tasnim S, Jahan MK, Nazmeen S, Debnath SC, Islam AM. Difficulties in breastfeeding: Easy solution by Oketani breast massage. Bangladesh Medical Research Council Bulletin. 2019;45(3):149-54.	Title and abstract not relevant- excluded
772.	Russell LB, Pentakota SR, Toscano CM, Cosgriff B, Sinha A. What pertussis mortality rates make maternal acellular pertussis immunization cost-effective in low-and middle-income countries? a decision analysis. Clinical Infectious Diseases. 2016;63(suppl_4):S227-S35.	Title and abstract not relevant- excluded
773.	Russell NJ, Seale AC, O'Driscoll M, O'Sullivan C, Bianchi-Jassir F, Gonzalez-Guarin J, et al. Maternal colonization with group B Streptococcus and serotype distribution worldwide: systematic review and meta-analyses. Clinical infectious diseases. 2017;65(suppl_2):S100-S11.	Title and abstract not relevant- excluded
774.	Ruysen H, Rahman AE, Gordeev VS, Hossain T, Basnet O, Shirima K, et al. Electronic data collection for multi-country, hospital-based, clinical observation of maternal and newborn care: EN-BIRTH study experiences. BMC pregnancy and childbirth. 2021;21:1-15.	Title and abstract not relevant- excluded
775.	Saggurti N, Atmavilas Y, Porwal A, Schooley J, Das R, Kande N, et al. Effect of health intervention integration within women's self-help groups on collectivization and healthy practices around reproductive, maternal, neonatal and child health in rural India. PLoS One. 2018;13(8):e0202562.	Title and abstract not relevant- excluded
776.	Saha KK, Frongillo EA, Alam DS, Arifeen SE, Persson LÅ, Rasmussen KM. Household food security is associated with infant feeding practices in rural Bangladesh. The Journal of nutrition. 2008;138(7):1383-90.	Title and abstract not relevant- excluded
777.	Saha UR, Khan MA, Begum M, Bairagi R. Determinants of pill failure in rural Bangladesh. Journal of biosocial science. 2004;36(1):39-50.	Title and abstract not relevant- excluded
778.	Salam M. Clinical research and service centre: an institutional profile and programmes. Glimpse (Dhaka, Bangladesh). 1997;19(3):4-5.	Title and abstract not relevant- excluded
779.	Salasibew MM, Filteau S, Marchant T. A qualitative study exploring newborn care behaviours after home births in rural Ethiopia: implications for adoption of essential interventions for saving newborn lives. BMC pregnancy and childbirth. 2014;14:1-7.	Title and abstract not relevant- excluded
780.	Salasibew MM, Moss C, Ayana G, Kuche D, Eshetu S, Dangour AD. The fidelity and dose of message delivery on infant and young child feeding practice and nutrition sensitive agriculture in Ethiopia: a qualitative study from the Sustainable Undernutrition Reduction in Ethiopia (SURE) programme. Journal of Health, Population and Nutrition. 2019;38:1-11.	Title and abstract not relevant- excluded
781.	Salem MK, Pitchik HO, Sultana J, Rahman M, Jannat KK, Luby SP, et al. Prevalence of Sugar-Sweetened Food Consumption in Rural Bangladeshi Children Aged 6–24 Months. The Journal of nutrition. 2022;152(9):2155-64.	Title and abstract not relevant- excluded
782.	Salim N, Shabani J, Peven K, Rahman QS-u, Kc A, Shamba D, et al. Kangaroo mother care: EN-BIRTH multi-country validation study. BMC pregnancy and childbirth. 2021;21:1-16.	Title and abstract not relevant- excluded

SL	Articles excluded during title and abstract screening	Decision
783.	Salzberg NT, Sivalogan K, Bassat Q, Taylor AW, Adedini S, El Arifeen S, et al. Mortality surveillance methods to identify and characterize deaths in child health and mortality prevention surveillance network sites. Clinical infectious diseases. 2019;69(Supplement_4):S262-S73.	Title and abstract not relevant- excluded
784.	Sanghvi T, Jimerson A, Hajeebhoy N, Zewale M, Nguyen GH. Tailoring communication strategies to improve infant and young child feeding practices in different country settings. Food and nutrition bulletin. 2013;34(3_suppl2):S169-S80.	Title and abstract not relevant- excluded
785.	Sanghvi T, Nguyen PH, Ghosh S, Zafimanjaka M, Walissa T, Karama R, et al. Process of developing models of maternal nutrition interventions integrated into antenatal care services in Bangladesh, Burkina Faso, Ethiopia and India. Maternal & child nutrition. 2022;18(4):e13379.	Title and abstract not relevant- excluded
786.	Sarker AR, Sultana M, Sheikh N, Akram R, Ali N, Mahumud RA, et al. Inequality of childhood undernutrition in Bangladesh: A decomposition approach. The International Journal of Health Planning and Management. 2020;35(2):441-68.	Title and abstract not relevant- excluded
787.	Sarker M, Ko S, Lee S, Kim G, Choi J, Yang C. Effect of different feed additives on growth performance and blood profiles of Korean Hanwoo calves. Asian-Australasian Journal of Animal Sciences. 2010;23(1):52-60.	Title and abstract not relevant- excluded
788.	Sarker M, Yang C. Propolis and illite as feed additives on performance and blood profiles of post-weaning Hanwoo calves. 2010.	Title and abstract not relevant- excluded
789.	Sarker SC, Parvin MS, Rahman AA, Islam MT. Prevalence and risk factors of subclinical mastitis in lactating dairy cows in north and south regions of Bangladesh. Tropical Animal Health and Production. 2013;45:1171-6.	Title and abstract not relevant- excluded
790.	Sazawal S, Bhan M, Bhandari N. Type of milk feeding during acute diarrhoea and the risk of persistent diarrhoea: a case control study. Acta paediatrica. 1992;81:93-7.	Title and abstract not relevant- excluded
791.	Senarath U, Siriwardena I, Godakandage SS, Jayawickrama H, Fernando DN, Dibley MJ. Determinants of breastfeeding practices: an analysis of the Sri Lanka Demographic and Health Survey 2006–2007. Maternal & child nutrition. 2012;8(3):315-29.	Title and abstract not relevant- excluded
792.	Sethi V, Bhanot A, Bhalla S, Bhattacharjee S, Daniel A, Sharma DM, et al. Partnering with women collectives for delivering essential women's nutrition interventions in tribal areas of eastern India: a scoping study. Journal of Health, Population and Nutrition. 2017;36:1-10.	Title and abstract not relevant- excluded
793.	Shah R, Mullany LC, Darmstadt GL, Talukder RR, Rahman SM, Mannan I, et al. Neonatal mortality risks among preterm births in a rural B angladeshi cohort. Paediatric and perinatal epidemiology. 2014;28(6):510-20.	Title and abstract not relevant- excluded
794.	Shahjahan M, Ahmed MR, Rahman MM, Afroz A. Factors affecting newborn care practices in Bangladesh. Paediatric and perinatal epidemiology. 2012;26(1):13-8.	Title and abstract not relevant- excluded
795.	Shamim AA, Christian P, Schulze KJ, Ali H, Kabir A, Rashid M, et al. Iodine status in pregnancy and household salt iodine content in rural Bangladesh. Maternal & child nutrition. 2012;8(2):162-73.	Title and abstract not relevant- excluded

SL	Articles excluded during title and abstract screening	Decision
796.	Shamim AA, Kabir A, Merrill RD, Ali H, Rashid M, Schulze K, et al. Plasma zinc, vitamin B12 and α-tocopherol are positively and plasma γ-tocopherol is negatively associated with Hb concentration in early pregnancy in north-west Bangladesh. Public health nutrition. 2013;16(8):1354-61.	Title and abstract not relevant- excluded
797.	Shamim AA, Mashreky SR, Ferdous T, Tegenfeldt K, Roy S, Rahman AF, et al. Pregnant women diet quality and its sociodemographic determinants in southwestern Bangladesh. Food and nutrition bulletin. 2016;37(1):14-26.	Title and abstract not relevant- excluded
798.	Shamsuddin M, Alam M, Hossein M, Goodger W, Bari F, Ahmed T, et al. Participatory rural appraisal to identify needs and prospects of market-oriented dairy industries in Bangladesh. Tropical Animal Health and Production. 2007;39:567-81.	Title and abstract not relevant- excluded
799.	Shamsuddin M, Goodger W, Hossein M, Azizunnesa, Bennett T, Nordlund K. A survey to identify economic opportunities for smallholder dairy farms in Bangladesh. Tropical Animal Health and Production. 2006;38:131-40.	Title and abstract not relevant- excluded
800.	Shankar B, Zanello G, Srinivasan CS. Rural-urban disparities in child nutrition in Bangladesh and Nepal. 2013.	Title and abstract not relevant- excluded
801.	Sharma IK, Byrne A. Early initiation of breastfeeding: a systematic literature review of factors and barriers in South Asia. International breastfeeding journal. 2016;11:1-12.	Title and abstract not relevant- excluded
802.	Sharma V, Leight J, AbdulAziz F, Giroux N, Nyqvist MB. Illness recognition, decision-making, and care-seeking for maternal and newborn complications: a qualitative study in Jigawa State, Northern Nigeria. Journal of Health, Population and Nutrition. 2017;36:59-74.	Title and abstract not relevant- excluded
803.	Sharmin KN, Sarwar N, Mumu SJ, Taleb DA, Flora MS. Postnatal depression and infant growth in an urban area of Bangladesh. Midwifery. 2019;74:57-67.	Title and abstract not relevant- excluded
804.	Shaw RL, Wallace LM, Bansal M. Is breast best? Perceptions of infant feeding. Community Practitioner. 2003;76:299-303.	Title and abstract not relevant- excluded
805.	Shillcutt SD, Lefevre AE, Lee AC, Baqui AH, Black RE, Darmstadt GL. Forecasting burden of long-term disability from neonatal conditions: results from the Projahnmo I trial, Sylhet, Bangladesh. Health policy and planning. 2013;28(4):435-52.	Title and abstract not relevant- excluded
806.	Sibley LM, Amare Y, Abebe ST, Belew ML, Shiffra K, Barry D. Appropriateness and timeliness of care-seeking for complications of pregnancy and childbirth in rural Ethiopia: a case study of the Maternal and Newborn Health in Ethiopia Partnership. Journal of Health, Population and Nutrition. 2017;36:9-24.	Title and abstract not relevant- excluded
807.	Simondon KB, Delaunay V, Diallo A, Elguero E, Simondon F. Lactational amenorrhea is associated with child age at the time of introduction of complementary food: a prospective cohort study in rural Senegal, West Africa. The American journal of clinical nutrition. 2003;78(1):154-61.	Title and abstract not relevant- excluded
808.	Simondon KB, Simondon F. Mothers prolong breastfeeding of undernourished children in rural Senegal. International Journal of Epidemiology. 1998;27(3):490-4.	Title and abstract not relevant- excluded
809.	Singh NS, Singh NS. Determinants of duration of breastfeeding amongst women in Manipur. Bangladesh Journal of Medical Science. 2011;10(4):235.	Title and abstract not relevant- excluded

SL	Articles excluded during title and abstract screening	Decision
810.	Singh R, Tripathi V, Kalaivani M, Singh K, Dwivedi S. Determinants of birth intervals in Tamil Nadu in India: developing Cox hazard models with validations and predictions. Revista Colombiana de Estadística. 2012;35(2):289-307.	Title and abstract not relevant- excluded
811.	Sinharoy SS. Women's empowerment and women's dietary diversity in urban and rural Bangladesh 2017.	Title and abstract not relevant- excluded
812.	Skröder H, Kippler M, De Loma J, Raqib R, Vahter M. Predictors of selenium biomarker kinetics in 4–9-year-old Bangladeshi children. Environment international. 2018;121:842-51.	Title and abstract not relevant- excluded
813.	Skröder HM, Hamadani JD, Tofail F, Persson LÅ, Vahter ME, Kippler MJ. Selenium status in pregnancy influences children's cognitive function at 1.5 years of age. Clinical Nutrition. 2015;34(5):923-30.	Title and abstract not relevant- excluded
814.	Sloan NL, Ahmed S, Mitra SN, Choudhury N, Chowdhury M, Rob U, et al. Community-based kangaroo mother care to prevent neonatal and infant mortality: a randomized, controlled cluster trial. Pediatrics. 2008;121(5):e1047-e59.	Title and abstract not relevant- excluded
815.	SM Jr MK, Rosliza A, Aynul M. Effects of Wealth on Nutritional Status of Pre-school Children in Bangladesh. Malaysian journal of nutrition. 2010;16(2):219-32.	Title and abstract not relevant- excluded
816.	Smith ER, Shankar AH, Wu LS, Aboud S, Adu-Afarwuah S, Ali H, et al. Modifiers of the effect of maternal multiple micronutrient supplementation on stillbirth, birth outcomes, and infant mortality: a meta-analysis of individual patient data from 17 randomised trials in low-income and middle-income countries. The Lancet Global Health. 2017;5(11):e1090-e100.	Title and abstract not relevant- excluded
817.	Smith N, Sievert LL, Muttukrishna S, Begum K, Murphy L, Sharmeen T, et al. Mismatch: a comparative study of vitamin D status in British-Bangladeshi migrants. Evolution, medicine, and public health. 2021;9(1):164-73.	Title and abstract not relevant- excluded
818.	Sobhan S, Müller-Hauser AA, Huda TMN, Waid JL, Gautam OP, Gon G, et al. Design, delivery, and determinants of uptake: findings from a food hygiene behavior change intervention in rural Bangladesh. BMC Public Health. 2022;22(1):887.	Title and abstract not relevant- excluded
819.	Solmi M, Thompson T, Estradé A, Agorastos A, Radua J, Cortese S, et al. Validation of the Collaborative Outcomes study on Health and Functioning during Infection Times (COH-FIT) questionnaire for adults. Journal of affective disorders. 2023;326:249-61.	Title and abstract not relevant- excluded
820.	Sotoudeh G, Kabiri S, Yeganeh HS, Koohdani F, Khajehnasiri F, Khosravi S. Predictors of dietary supplement usage among medical interns of Tehran university of medical sciences. Journal of health, population, and nutrition. 2015;33(1):68.	Title and abstract not relevant- excluded
821.	Srivastava A, Bhattacharyya S, Gautham M, Schellenberg J, Avan BI. Linkages between public and non-government sectors in healthcare: a case study from Uttar Pradesh, India. Global public health. 2016;11(10):1216-30.	Title and abstract not relevant- excluded
822.	Starkweather KE. Shodagor Family Strategies: Balancing work and family on the water. Human Nature. 2017;28:138-66.	Title and abstract not relevant- excluded

SL	Articles excluded during title and abstract screening	Decision
823.	Stevens B, Watt K, Brimblecombe J, Clough A, Judd J. Development of a locally produced, balanced protein–energy food-based supplement and its acceptance by undernourished pregnant women in Northern Bangladesh. Journal of Hunger & Environmental Nutrition. 2018;13(1):100-15.	Title and abstract not relevant- excluded
824.	Stewart CP, Iannotti L, Dewey KG, Michaelsen KF, Onyango AW. Contextualising complementary feeding in a broader framework for stunting prevention. Maternal & child nutrition. 2013;9:27-45.	Title and abstract not relevant- excluded
825.	Stewart CP, Wessells KR, Arnold CD, Huybregts L, Ashorn P, Becquey E, et al. Lipid-based nutrient supplements and all-cause mortality in children 6–24 months of age: a meta-analysis of randomized controlled trials. The American journal of clinical nutrition. 2020;111(1):207-18.	Title and abstract not relevant- excluded
826.	Sundaram ME, Labrique AB, Mehra S, Ali H, Shamim AA, Klemm RD, et al. Early Neonatal Feeding Is Common and Associated with Subsequent Breastfeeding Behavior in Rural Bangladesh1–3. The Journal of nutrition. 2013;143(7):1161-7.	Title and abstract not relevant- excluded
827.	Svefors P, Selling KE, Shaheen R, Khan AI, Persson L-Å, Lindholm L. Cost-effectiveness of prenatal food and micronutrient interventions on under-five mortality and stunting: Analysis of data from the MINIMat randomized trial, Bangladesh. PLoS One. 2018;13(2):e0191260.	Title and abstract not relevant- excluded
828.	Svefors P, Sysoev O, Ekstrom E-C, Persson LA, Arifeen SE, Naved RT, et al. Relative importance of prenatal and postnatal determinants of stunting: data mining approaches to the MINIMat cohort, Bangladesh. BMJ open. 2019;9(8):e025154.	Title and abstract not relevant- excluded
829.	Syed U, Asiruddin S, Helal MS, Mannan II, Murray J. Immediate and early postnatal care for mothers and newborns in rural Bangladesh. Journal of health, population, and nutrition. 2006;24(4):508.	Title and abstract not relevant- excluded
830.	Tablante EC, Pachón H, Guetterman HM, Finkelstein JL. Fortification of wheat and maize flour with folic acid for population health outcomes. Cochrane Database of Systematic Reviews. 2019(7).	Title and abstract not relevant- excluded
831.	Tahsina T, Hossain AT, Ruysen H, Rahman AE, Day LT, Peven K, et al. Immediate newborn care and breastfeeding: EN-BIRTH multi-country validation study. BMC pregnancy and childbirth. 2021;21:1-17.	Title and abstract not relevant- excluded
832.	Talukder A. Risk factors associated with wasting among under-5 children residing in urban areas of Bangladesh: a multilevel modelling approach. Journal of Public Health. 2021;29:525-31.	Title and abstract not relevant- excluded
833.	Tariqujjaman M, Rahman M, Luies SK, Karmakar G, Ahmed T, Sarma H. Unintended consequences of programmatic changes to infant and young child feeding practices in Bangladesh. Maternal & Child Nutrition. 2021;17(2):e13077.	Title and abstract not relevant- excluded
834.	Taylor A. Violations of the international code of marketing of breast milk substitutes: prevalence in four countries. Bmj. 1998;316(7138):1117-22.	Title and abstract not relevant- excluded
835.	Teka T, Faruque A, Fuchs G. Risk factors for deaths in under-age-five children attending a diarrhoea treatment centre. Acta Paediatrica. 1996;85(9):1070-5.	Title and abstract not relevant- excluded
836.	Tesfau YB, Gebrehiwot TG, Godefay H, Kahsay AB. Effect of health facility linkage with community using postnatal card on postnatal home visit coverage and newborn care practices in rural Ethiopia: A controlled quasi-experimental study design. Plos one. 2022;17(5):e0267686.	Title and abstract not relevant- excluded

SL	Articles excluded during title and abstract screening	Decision
837.	Thapa K, Adhikary P, Faruquee MH, Suwal BR. Associated factors for dropout of first vs third doses of diphtheria tetanus pertussis (DPT) vaccination in Nepal. Advances in Preventive Medicine. 2021;2021(1):1319090.	Title and abstract not relevant- excluded
838.	Thayyil S, Pant S, Montaldo P, Shukla D, Oliveira V, Ivain P, et al. Hypothermia for moderate or severe neonatal encephalopathy in low-income and middle-income countries (HELIX): a randomised controlled trial in India, Sri Lanka, and Bangladesh. The Lancet Global Health. 2021;9(9):e1273-e85.	Title and abstract not relevant- excluded
839.	Thornburg J, Islam S, Billah SM, Chan B, McCombs M, Abbott M, et al. Pregnant women's exposure to household air pollution in rural Bangladesh: a feasibility study for Poriborton: the CHANge trial. International Journal of Environmental Research and Public Health. 2022;19(1):482.	Title and abstract not relevant- excluded
840.	Thorne-Lyman AL, Shaikh S, Mehra S, Wu LS, Ali H, Alland K, et al. Dietary patterns of> 30,000 adolescents 9–15 years of age in rural Bangladesh. Annals of the new York Academy of Sciences. 2020;1468(1):3-15.	Title and abstract not relevant- excluded
841.	Tikadar KK, Islam MJ, Saha SM, Alam MM, Barman SK, Rahman MA. Livelihood status of small-scale fishermen and determinants of their income: Insights from north-eastern floodplains of Bangladesh. Geography and Sustainability. 2022;3(3):204-13.	Title and abstract not relevant- excluded
842.	Tran MC, Labrique AB, Mehra S, Ali H, Shaikh S, Mitra M, et al. Analyzing the mobile "digital divide": changing determinants of household phone ownership over time in rural Bangladesh. JMIR mHealth and uHealth. 2015;3(1):e3663.	Title and abstract not relevant- excluded
843.	Trevisi E, Jahan N, Bertoni G, Ferrari A, Minuti A. Pro-inflammatory cytokine profile in dairy cows: consequences for new lactation. Italian Journal of Animal Science. 2015;14(3):3862.	Title and abstract not relevant- excluded
844.	Uddin MF, Jabeen I, Islam MA, Rahman M, Chisti MJ, Ahmed T, et al. Barriers to breastfeeding are shaped by sociocultural context: an exploratory qualitative study in Bangladesh. Journal of Health, Population and Nutrition. 2022;41(1):34.	Title and abstract not relevant- excluded
845.	Uddin MF, Molyneux S, Muraya K, Hossain MA, Islam MA, Shahid ASMSB, et al. Gender-related influences on adherence to advice and treatment-seeking guidance for infants and young children post-hospital discharge in Bangladesh. International journal for equity in health. 2021;20:1-19.	Title and abstract not relevant- excluded
846.	Udo H, Hermans C, Dawood F. Comparison of two cattle production systems in Pabna district Bangladesh. Trop Anim Heal Prod. 1990;22:247-59.	Title and abstract not relevant- excluded
847.	Ulijaszek SJ, Leighton D. Maternal employment and child nutritional status in a very poor population of residents and migrants from Bangladesh in Calcutta, India. Anthropological Science. 1998;106(3):253-63.	Title and abstract not relevant- excluded
848.	Uwitonze AM, Uwambaye P, Isyagi M, Mumena CH, Hudder A, Haq A, et al. Periodontal diseases and adverse pregnancy outcomes: Is there a role for vitamin D? The Journal of steroid biochemistry and molecular biology. 2018;180:65-72.	Title and abstract not relevant- excluded
849.	Van Roosmalen J. World population, family planning and development. Nederlands tijdschrift voor geneeskunde. 1992;136(12):589-90.	Title and abstract not relevant- excluded
850.	Vasudevan L, Labrique AB, Mehra S, Wu L, Levine O, Feikin D, et al. Maternal determinants of timely vaccination coverage among infants in rural Bangladesh. Vaccine. 2014;32(42):5514-9.	Title and abstract not relevant- excluded

SL	Articles excluded during title and abstract screening	Decision
851.	Verguet S, Nandi A, Filippi V, Bundy DA. Postponing adolescent parity in developing countries through education: an extended cost-effectiveness analysis. 2018.	Title and abstract not relevant- excluded
852.	Wable Grandner G, Rasmussen KM, Dickin KL, Menon P, Yeh T, Hoddinott J. Storytelling for persuasion: Insights from community health workers on how they engage family members to improve adoption of recommended maternal nutrition and breastfeeding behaviours in rural Bangladesh. Maternal & Child Nutrition. 2022;18(4):e13408.	Title and abstract not relevant- excluded
853.	Waid JL, Nielsen JN, Afroz S, Lindsey D, Sinharoy SS. Use of the Essential Nutrition Actions framework improved child growth in Bangladesh. Maternal & Child Nutrition. 2019;15(2):e12691.	Title and abstract not relevant- excluded
854.	Waiswa P, Pariyo G, Kallander K, Akuze J, Namazzi G, Ekirapa-Kiracho E, et al. Effect of the Uganda Newborn Study on care-seeking and care practices: a cluster-randomised controlled trial. Global health action. 2015;8(1):24584.	Title and abstract not relevant- excluded
855.	Ward JL, Azzopardi PS, Francis KL, Santelli JS, Skirbekk V, Sawyer SM, et al. Global, regional, and national mortality among young people aged 10–24 years, 1950–2019: a systematic analysis for the Global Burden of Disease Study 2019. The Lancet. 2021;398(10311):1593-618.	Title and abstract not relevant- excluded
856.	Warren AM, Frongillo EA, Nguyen PH, Menon P. Nutrition intervention using behavioral change communication without additional material inputs increased expenditures on key food groups in Bangladesh. The Journal of Nutrition. 2020;150(5):1284-90.	Title and abstract not relevant- excluded
857.	Wessells KR, Arnold CD, Stewart CP, Prado EL, Abbeddou S, Adu-Afarwuah S, et al. Characteristics that modify the effect of small-quantity lipid-based nutrient supplementation on child anemia and micronutrient status: an individual participant data meta-analysis of randomized controlled trials. The American journal of clinical nutrition. 2021;114:68S-94S.	Title and abstract not relevant- excluded
858.	West KP, Christian P, Labrique AB, Rashid M, Shamim AA, Klemm RD, et al. Effects of vitamin A or beta carotene supplementation on pregnancy-related mortality and infant mortality in rural Bangladesh: a cluster randomized trial. Jama. 2011;305(19):1986-95.	Title and abstract not relevant- excluded
859.	Wilkins E, Wickramasinghe K, Pullar J, Demaio AR, Roberts N, Perez-Blanco K-M, et al. Maternal nutrition and its intergenerational links to non-communicable disease metabolic risk factors: a systematic review and narrative synthesis. Journal of Health, Population and Nutrition. 2021;40:1-11.	Title and abstract not relevant- excluded
860.	Win H, Shafique S, Mizan S, Wallenborn J, Probst-Hensch N, Fink G. Association between mother’s work status and child stunting in urban slums: a cross-sectional assessment of 346 child-mother dyads in Dhaka, Bangladesh (2020). Archives of Public Health. 2022;80(1):192.	Title and abstract not relevant- excluded
861.	Wise J. Baby milk companies accused of breaching marketing code. BMJ: British Medical Journal. 1997;314(7075):167.	Title and abstract not relevant- excluded
862.	Woograsingh S. A single flavour of motherhood: An emerging identity in a young Bangladeshi woman. Infant Observation. 2007;10(3):267-79.	Title and abstract not relevant- excluded
863.	Wrigley-Asante C. Out of the dark but not out of the cage: women's empowerment and gender relations in the Dangme West district of Ghana. Gender, Place & Culture. 2012;19(3):344-63.	Title and abstract not relevant- excluded

SL	Articles excluded during title and abstract screening	Decision
864.	Yapo YV. Breastfeeding and child survival from 0 to 5 years in Côte d'Ivoire. Journal of Health, Population and Nutrition. 2020;39:1-9.	Title and abstract not relevant- excluded
865.	Yargawa J, Machiyama K, Ponce Hardy V, Enuameh Y, Galiwango E, Gelaye K, et al. Pregnancy intention data completeness, quality and utility in population-based surveys: EN-INDEPTH study. Population health metrics. 2021;19:1-18.	Title and abstract not relevant- excluded
866.	Yeasmin F, Winch PJ, Hwang ST, Leontsini E, Jahir T, Das JB, et al. Exploration of attendance, active participation, and behavior change in a group-based responsive stimulation, maternal and child health, and nutrition intervention. The American journal of tropical medicine and hygiene. 2021;104(4):1586.	Title and abstract not relevant- excluded
867.	Zakayo SM, Njeru RW, Sanga G, Kimani MN, Charo A, Muraya K, et al. Vulnerability and agency across treatment-seeking journeys for acutely ill children: how family members navigate complex healthcare before, during and after hospitalisation in a rural Kenyan setting. International Journal for Equity in Health. 2020;19:1-17.	Title and abstract not relevant- excluded
868.	Zaman M, Bhuiyan M, Huq S, Rahman M, Sinha D, Fernando T. Dual use of tobacco among Bangladeshi men. Indian journal of cancer. 2014;51(Suppl 1):S46-S9.	Title and abstract not relevant- excluded
869.	Zaman S, Ashraf RN, Martines J. Training in complementary feeding counselling of healthcare workers and its influence on maternal behaviours and child growth: a cluster-randomized controlled trial in Lahore, Pakistan. Journal of health, population, and nutrition. 2008;26(2):210.	Title and abstract not relevant- excluded
870.	Zarei P, Rezvanfar MR, Khosrowbeygi A. Coenzyme Q10 supplementation effects on lipid ratios in women with type 2 diabetes mellitus: A randomized, double-blind clinical trial study. Bangladesh Journal of Medical Science. 2020;19(1):152.	Title and abstract not relevant- excluded
871.	Zeitlyn S, Rowshan R. Privileged knowledge and mothers'"perceptions": The case of breast-feeding and insufficient milk in Bangladesh. Medical Anthropology Quarterly. 1997;11(1):56-68.	Title and abstract not relevant- excluded
872.	Zerfu TA, Umeta M, Baye K. Dietary habits, food taboos, and perceptions towards weight gain during pregnancy in Arsi, rural central Ethiopia: a qualitative cross-sectional study. Journal of Health, Population and Nutrition. 2016;35:1-7.	Title and abstract not relevant- excluded
873.	Zhang S, Sammon PM, King I, Andrade AL, Toscano CM, Araujo SN, et al. Cost of management of severe pneumonia in young children: systematic analysis. Journal of global health. 2016;6(1).	Title and abstract not relevant- excluded
874.	Zhang Y, Zhou J, Niu F, Donowitz JR, Haque R, Petri WA, et al. Characterizing early child growth patterns of height-for-age in an urban slum cohort of Bangladesh with functional principal component analysis. BMC pediatrics. 2017;17:1-11.	Title and abstract not relevant- excluded
875.	Dibley M, Arifeen S, Braat S. Benefits and risks of Iron interventions in children (BRISC): protocol for a three-arm parallel-group randomised controlled field trial in Bangladesh. 2017.	Title and abstract not relevant- excluded
876.	Bogard JR, Hother A-L, Saha M, Bose S, Kabir H, Marks GC, et al. Inclusion of small indigenous fish improves nutritional quality during the first 1000 days. Food and nutrition bulletin. 2015;36(3):276-89.	Title and abstract not relevant- excluded
877.	Morseth MS, Torheim LE, Chandyo RK, Ulak M, Shrestha SK, Shrestha B, et al. Severely inadequate micronutrient intake among children 9–24 months in Nepal—The MAL-ED birth cohort study. Maternal & Child Nutrition. 2018;14(2):e12552.	Title and abstract not relevant- excluded

SL	Articles excluded during title and abstract screening	Decision
878.	Enuameh YAK, Dzabeng F, Blencowe H, Thysen SM, Abebe SM, Asante KP, et al. Termination of pregnancy data completeness and feasibility in population-based surveys: EN-INDEPTH study. Population health metrics. 2021;19:1-16.	Title and abstract not relevant- excluded
879.	Islam MA, Sharma A, Ahsan S, Mazumdar S, Rudra K, Phillips CJ. Welfare assessment of dairy cows in small farms in Bangladesh. Animals. 2020;10(3):394.	Title and abstract not relevant- excluded
880.	Fischer Walker CL, Walker N. The Lives Saved Tool (LiST) as a model for diarrhea mortality reduction. BMC medicine. 2014;12:1-13.	Title and abstract not relevant- excluded
881.	Williams FB, Kader A, Colgate ER, Dickson DM, Carmolli M, Uddin MI, et al. Maternal secretor status affects oral rotavirus vaccine response in breastfed infants in Bangladesh. The Journal of infectious diseases. 2021;224(7):1147-51.	Title and abstract not relevant- excluded
882.	Mahmud M, Gutierrez IA, Kumar KB, Nataraj S. What aspects of formality do Workers value? Evidence from a choice experiment in Bangladesh. The World Bank Economic Review. 2021;35(2):303-27.	Title and abstract not relevant- excluded
883.	Prata N, Passano P, Rowen T, Bell S, Walsh J, Potts M. Where there are (few) skilled birth attendants. Journal of health, population, and nutrition. 2011;29(2):81.	Title and abstract not relevant- excluded
884.	Islam MR, Rahman SM, Tarafder C, Rahman MM, Rahman A, Ekström E-C. Exploring rural adolescents' dietary diversity and its socioeconomic correlates: a cross-sectional study from Matlab, Bangladesh. Nutrients. 2020;12(8):2230.	Title and abstract not relevant- excluded
885.	Sathi NJ, Ahammed B, Alam K, Hashmi R, Lee KY, Keramat SA. Socioeconomic inequalities in low birth weight in South Asia: A comparative analysis using Demographic and Health Surveys. SSM-Population Health. 2022;20:101248.	Title and abstract not relevant- excluded
886.	Lokonon JH, Hounkpatin WA, Bodjrenou SU, Sokadjo MY, Dossou NI, Atchadé MN, et al. The role of exclusive breastfeeding and water sources in the reduction of stunting: Mediation and moderation analysis of cross-sectional data among Beninese children aged 6 months. PROGRESS IN NUTRITION. 2022;24(2).	Title and abstract not relevant- excluded
887.	Naz S, Page A, Agho KE. Potential impacts of modifiable behavioral and environmental exposures on reducing burden of under-five mortality associated with household air pollution in Nepal. Maternal and child health journal. 2018;22:59-70.	Title and abstract not relevant- excluded
888.	Bisimwa G, Owino VO, Bahwere P, Dramaix M, Donnen P, Dibari F, et al. Randomized controlled trial of the effectiveness of a soybean-maize-sorghum–based ready-to-use complementary food paste on infant growth in South Kivu, Democratic Republic of Congo. The American journal of clinical nutrition. 2012;95(5):1157-64.	Title and abstract not relevant- excluded
889.	Panter-Brick C. Lactation, birth spacing and maternal work-loads among two castes in rural Nepal. Journal of Biosocial Science. 1991;23(2):137-54.	Title and abstract not relevant- excluded
890.	Moore Z, Pfitzer A, Gubin R, Charurat E, Elliott L, Croft T. Missed opportunities for family planning: an analysis of pregnancy risk and contraceptive method use among postpartum women in 21 low-and middle-income countries. Contraception. 2015;92(1):31-9.	Title and abstract not relevant- excluded
891.	Morris SK, Pell LG, Rahman MZ, Dimitris MC, Mahmud A, Islam MM, et al. Maternal vitamin D supplementation during pregnancy and lactation to prevent acute respiratory infections in infancy	Title and abstract not relevant- excluded

SL	Articles excluded during title and abstract screening	Decision
	in Dhaka, Bangladesh (MDARI trial): protocol for a prospective cohort study nested within a randomized controlled trial. BMC pregnancy and childbirth. 2016;16:1-10.	
892.	Moxon SG, Ruysen H, Kerber KJ, Amouzou A, Fournier S, Grove J, et al. Count every newborn; a measurement improvement roadmap for coverage data. BMC pregnancy and childbirth. 2015;15:1-23.	Title and abstract not relevant- excluded
893.	Mulmi P, Masters WA, Ghosh S, Namirembe G, Rajbhandary R, Manohar S, et al. Household food production is positively associated with dietary diversity and intake of nutrient-dense foods for older preschool children in poorer families: Results from a nationally-representative survey in Nepal. PloS one. 2017;12(11):e0186765.	Title and abstract not relevant- excluded
894.	Murray CJ, Callender CS, Kulikoff XR, Srinivasan V, Abate D, Abate KH, et al. Population and fertility by age and sex for 195 countries and territories, 1950–2017: a systematic analysis for the Global Burden of Disease Study 2017. The Lancet. 2018;392(10159):1995-2051.	Title and abstract not relevant- excluded
895.	Murray SF, Hunter BM, Bisht R, Ensor T, Bick D. Demand-side financing measures to increase maternal health service utilisation and improve health outcomes: a systematic review of evidence from low-and middle-income countries. JBI Evidence Synthesis. 2012;10(58):4165-567.	Title and abstract not relevant- excluded
896.	Mutoro AN, Garcia AL, Kimani-Murage EW, Wright CM. Prevalence and overlap of known undernutrition risk factors in children in Nairobi Kenya. Maternal & child nutrition. 2022;18(1):e13261.	Title and abstract not relevant- excluded
897.	Mychaleckyj JC, Nayak U, Colgate ER, Zhang D, Carstensen T, Ahmed S, et al. Multiplex genomewide association analysis of breast milk fatty acid composition extends the phenotypic association and potential selection of FADS1 variants to arachidonic acid, a critical infant micronutrient. Journal of Medical Genetics. 2018;55(7):459-68.	Title and abstract not relevant- excluded
898.	Mychaleckyj JC, Zhang D, Nayak U, Ross Colgate E, Carmolli M, Dickson D, et al. Association of breast milk gamma-linolenic acid with infant anthropometric outcomes in urban, low-income Bangladeshi families: a prospective, birth cohort study. European journal of clinical nutrition. 2020;74(5):698-707.	Title and abstract not relevant- excluded
899.	Na M, Aguayo VM, Arimond M, Mustaphi P, Stewart CP. Predictors of complementary feeding practices in Afghanistan: Analysis of the 2015 Demographic and Health Survey. Maternal & child nutrition. 2018;14:e12696.	Title and abstract not relevant- excluded
900.	Na M, Mehra S, Christian P, Ali H, Shaikh S, Shamim AA, et al. Maternal dietary diversity decreases with household food insecurity in rural Bangladesh: a longitudinal analysis. The Journal of nutrition. 2016;146(10):2109-16.	Title and abstract not relevant- excluded
901.	Ganpule-Rao A, Bhat D, Yajnik C, Rush E. Dietary diversity scores, nutrient intakes and biomarkers vitamin B12, folate and Hb in rural youth from the Pune Maternal Nutrition Study. British Journal of Nutrition. 2021;126(2):236-43.	Title and abstract not relevant- excluded

SL	Articles excluded during title and abstract screening	Decision
902.	Barger-Kamate B, Deloria Knoll M, Kagucia EW, Prosperi C, Baggett HC, Brooks WA, et al. Pertussis-associated pneumonia in infants and children from low-and middle-income countries participating in the PERCH study. Clinical Infectious Diseases. 2016;63(suppl_4):S187-S96.	Title and abstract not relevant- excluded
903.	Gewa CA, Leslie TF. Distribution and determinants of young child feeding practices in the East African region: demographic health survey data analysis from 2008-2011. Journal of Health, Population and Nutrition. 2015;34:1-14.	Title and abstract not relevant- excluded
904.	Zumrawi F. Effects of the relative importance of different factors and their degree of interactions on child growth. Journal of tropical pediatrics. 1991;37(3):131-5.	Title and abstract not relevant- excluded
905.	Diana A, Mallard SR, Haszard JJ, Purnamasari DM, Nurulazmi I, Herliani PD, et al. Consumption of fortified infant foods reduces dietary diversity but has a positive effect on subsequent growth in infants from Sumedang district, Indonesia. Plos one. 2017;12(4):e0175952.	Title and abstract not relevant- excluded
906.	Moni NN, Haider MZ, Al Masud MM. Institutional practices and vulnerability of shrimp fry catchers in the south-west region of Bangladesh. International Journal of Social Economics. 2018;45(11):1533-49.	Title and abstract not relevant- excluded
907.	Tiruneh SA, Zeleke EG, Animut Y. Time to death and its associated factors among infants in sub-Saharan Africa using the recent demographic and health surveys: shared frailty survival analysis. BMC pediatrics. 2021;21:1-13.	Title and abstract not relevant- excluded
908.	Akter T, Dawson A, Sibbritt D. What impact does antenatal and postnatal care have on neonatal deaths in low-and lower-middle-income countries? Evidence from Bangladesh. Health Care for Women International. 2017;38(8):848-60.	Title and abstract not relevant- excluded
909.	Munos MK, Mullany LC, Maïga A, Baya B, Bryce J. Coverage and determinants of newborn feeding practices in rural Burkina Faso. Journal of Perinatology. 2014;34(5):369-74.	Title and abstract not relevant- excluded
910.	Rahman MM. James D Shelton. Bull World Health Organ. 2013;91:449-58.	Title and abstract not relevant- excluded
911.	Hussain M, Sikder Z, Wahed M, Haque A, Jahan F. Zinc concentration of breast milk and its diurnal variation in Bangladeshi mothers. Bangladesh Medical Research Council bulletin. 1996;22(2):70-3.	Title and abstract not relevant- excluded
912.	Boone K, Morris SK, Doshi S, Black J, Mohsin M, Ahmed T, et al. Antimicrobial Prescribing during Infant Hospital Admissions in a Birth Cohort in Dhaka, Bangladesh. Journal of Tropical Pediatrics. 2021;67(3):fmaa093.	Title and abstract not relevant- excluded
913.	Almroth S, Mohale M, Latham MC. Grandma ahead of her time: traditional ways of diarrhoea management in Lesotho. Journal of Diarrhoeal Diseases Research. 1997:167-72.	Title and abstract not relevant- excluded
914.	Coles CL, Labrique A, Saha SK, Ali H, Al-Emran H, Rashid M, et al. Newborn vitamin A supplementation does not affect nasopharyngeal carriage of Streptococcus pneumoniae in Bangladeshi infants at age 3 months. The Journal of nutrition. 2011;141(10):1907-11.	Title and abstract not relevant- excluded
915.	Memon ZA, Khan GN, Soofi SB, Baig IY, Bhutta ZA. Impact of a community-based perinatal and newborn preventive care package on perinatal and neonatal mortality in a remote mountainous district in Northern Pakistan. BMC pregnancy and childbirth. 2015;15:1-9.	Title and abstract not relevant- excluded

SL	Articles excluded during title and abstract screening	Decision
916.	Habtewold TD, Mohammed SH, Endalamaw A, Akibu M, Sharew NT, Alemu YM, et al. Breast and complementary feeding in Ethiopia: new national evidence from systematic review and meta-analyses of studies in the past 10 years. European journal of nutrition. 2019;58:2565-95.	Title and abstract not relevant- excluded
917.	Mohsin F, Azad K, Zabeen B, Tayyeb S, Baki A, Nahar N. Should type 1 diabetics fast in Ramadan. J Pak Med Assoc. 2015;65(5):S26-S9.	Title and abstract not relevant- excluded
918.	Peña-Rosas JP, Mithra P, Unnikrishnan B, Kumar N, De-Regil LM, Nair NS, et al. Fortification of rice with vitamins and minerals for addressing micronutrient malnutrition. Cochrane Database of Systematic Reviews. 2019(10).	Title and abstract not relevant- excluded
919.	Dicker D, Nguyen G, Abate D, Abate KH, Abay SM, Abbafati C, et al. Global, regional, and national age-sex-specific mortality and life expectancy, 1950–2017: a systematic analysis for the Global Burden of Disease Study 2017. The lancet. 2018;392(10159):1684-735.	Title and abstract not relevant- excluded
920.	Lo SW, Mellor K, Cohen R, Alonso AR, Belman S, Kumar N, et al. Emergence of a multidrug-resistant and virulent Streptococcus pneumoniae lineage mediates serotype replacement after PCV13: an international whole-genome sequencing study. The Lancet Microbe. 2022;3(10):e735-e43.	Title and abstract not relevant- excluded
921.	Shamim AA, Schulze K, Merrill RD, Kabir A, Christian P, Shaikh S, et al. First-trimester plasma tocopherols are associated with risk of miscarriage in rural Bangladesh. The American journal of clinical nutrition. 2015;101(2):294-301.	Title and abstract not relevant- excluded
922.	Razzaque A, Nahar L, Van Minh H, Ng N, Juvekar S, Ashraf A, et al. Social factors and overweight: evidence from nine Asian INDEPTH Network sites. Global health action. 2009;2(1):1991.	Title and abstract not relevant- excluded
923.	Persson LÅ, Arifeen S, Ekström E-C, Rasmussen KM, Frongillo EA, Team MS. Effects of prenatal micronutrient and early food supplementation on maternal hemoglobin, birth weight, and infant mortality among children in Bangladesh: the MINIMat randomized trial. Jama. 2012;307(19):2050-9.	Title and abstract not relevant- excluded
924.	Saleem S, McClure EM, Goudar SS, Patel A, Esamai F, Garces A, et al. A prospective study of maternal, fetal and neonatal deaths in low-and middle-income countries. Bulletin of the World Health Organization. 2014;92:605-12.	Title and abstract not relevant- excluded
925.	Van Minh H, Soonthornthada K, Ng N, Juvekar S, Razzaque A, Ashraf A, et al. Blood pressure in adult rural INDEPTH population in Asia. Global health action. 2009;2(1):2010.	Title and abstract not relevant- excluded
926.	Sania A, Sudfeld CR, Danaei G, Fink G, McCoy DC, Zhu Z, et al. Early life risk factors of motor, cognitive and language development: a pooled analysis of studies from low/middle-income countries. BMJ open. 2019;9(10):e026449.	Title and abstract not relevant- excluded
927.	Vaag A. Low birth weight and early weight gain in the metabolic syndrome: consequences for infant nutrition. International journal of gynecology & obstetrics. 2009;104:S32-S4.	Title and abstract not relevant- excluded
928.	Kamiya Y, Yoshimura Y, Islam MT. An impact evaluation of the safe motherhood promotion project in Bangladesh: evidence from Japanese aid-funded technical cooperation. Social science & medicine. 2013;83:34-41.	Title and abstract not relevant- excluded
929.	Ahmed T, Mahfuz M, Ireen S, Ahmed AS, Rahman S, Islam MM, et al. Nutrition of children and women in Bangladesh: trends and directions for the future. Journal of health, population, and nutrition. 2012;30(1):1.	Title and abstract not relevant- excluded

SL	Articles excluded during title and abstract screening	Decision
930.	Black MM, Baqui AH, Zaman K, El Arifeen S, Black RE. Maternal depressive symptoms and infant growth in rural Bangladesh. The American journal of clinical nutrition. 2009;89(3):951S-7S.	Title and abstract not relevant- excluded
931.	Nguyen PH, Frongillo EA, Sanghvi T, Kim SS, Alayon S, Tran LM, et al. Importance of coverage and quality for impact of nutrition interventions delivered through an existing health programme in Bangladesh. Maternal & child nutrition. 2018;14(4):e12613.	Title and abstract not relevant- excluded
932.	Talukder AK, Rashid MB, Yousef MS, Kusama K, Shimizu T, Shimada M, et al. Oviduct epithelium induces interferon-tau in bovine Day-4 embryos, which generates an anti-inflammatory response in immune cells. Scientific Reports. 2018;8(1):7850.	Title and abstract not relevant- excluded
933.	Islam S, Hasan M, Ghosh N, Islam M. Prospects and problems of indigenous sheep production in south-western coastal regions of Bangladesh. Journal of Agricultural Sciences–Sri Lanka. 2021;16(1).	Title and abstract not relevant- excluded
934.	Gardner RM, Kippler M, Tofail F, Bottai M, Hamadani J, Grandér M, et al. Environmental exposure to metals and children's growth to age 5 years: a prospective cohort study. American journal of epidemiology. 2013;177(12):1356-67.	Title and abstract not relevant- excluded
935.	Huq A, Delaney A, Debney B. Challenging the entrepreneurial discourse around women home-based workers' empowerment. The Economic and Labour Relations Review. 2022;33(2):308-28.	Title and abstract not relevant- excluded
936.	Singh PN, Kheam T, Lopez J, Job JS, Yel D. Patterns of maternal tobacco use among Cambodian women: findings from a nationwide sample. Asia Pacific Journal of Public Health. 2013;25(5_suppl):54S-63S.	Title and abstract not relevant- excluded
937.	Watson F, Ngesa A, Onyang'o J, Alnwick D, Tomkins A. Fermentation-a traditional anti-diarrhoeal practice lost? The use of fermented foods in urban and rural Kenya. International journal of food sciences and nutrition. 1996;47(2):171-9.	Title and abstract not relevant- excluded
938.	Yousafzai AK, Rasheed MA, Rizvi A, Armstrong R, Bhutta ZA. Effect of integrated responsive stimulation and nutrition interventions in the Lady Health Worker programme in Pakistan on child development, growth, and health outcomes: a cluster-randomised factorial effectiveness trial. The Lancet. 2014;384(9950):1282-93.	Title and abstract not relevant- excluded
939.	Reiner Jr RC, Hay SI. The overlapping burden of the three leading causes of disability and death in sub-Saharan African children. Nature communications. 2022;13(1):7457.	Title and abstract not relevant- excluded
940.	Namazzi G, Okuga M, Tetui M, Muhumuza Kananura R, Kakaire A, Namutamba S, et al. Working with community health workers to improve maternal and newborn health outcomes: implementation and scale-up lessons from eastern Uganda. Global health action. 2017;10(sup4):1345495.	Title and abstract not relevant- excluded
941.	Nadim M. Reinterpreting the relation between motherhood and paid work: second-generation immigrant women in Norway. The Sociological Review. 2014;62(3):494-511.	Title and abstract not relevant- excluded
942.	Seale AC, Bianchi-Jassir F, Russell NJ, Kohli-Lynch M, Tann CJ, Hall J, et al. Estimates of the burden of group B streptococcal disease worldwide for pregnant women, stillbirths, and children. Clinical infectious diseases. 2017;65(suppl_2):S200-S19.	Title and abstract not relevant- excluded
943.	Nahar S, Mascie-Taylor CN, Begum HA. Impact of targeted food supplementation on pregnancy weight gain and birth weight in rural Bangladesh: an assessment of the Bangladesh Integrated Nutrition Program (BINP). Public health nutrition. 2009;12(8):1205-12.	Title and abstract not relevant- excluded

SL	Articles excluded during title and abstract screening	Decision
944.	Ahirwar R, Mondal PR. Prevalence of obesity in India: A systematic review. Diabetes & Metabolic Syndrome: Clinical Research & Reviews. 2019;13(1):318-21.	Title and abstract not relevant- excluded
945.	Ahmed I, Rabbi MB, Sultana S. Antibiotic resistance in Bangladesh: A systematic review. International Journal of Infectious Diseases. 2019;80:54-61.	Title and abstract not relevant- excluded
946.	Akter S, Rahman MM. Duration of breastfeeding and its correlates in Bangladesh. Journal of health, population, and nutrition. 2010;28(6):595.	Title and abstract not relevant- excluded
947.	Arugula P, Paramasivam SK, Kanuri N, Srirangam A, Vemuluri M. Perception on use of sanitary napkins among students in Khammam locality: a survey. Indian Journal of Pharmacy Practice. 2017;10(2).	Title and abstract not relevant- excluded
948.	Aylward GP. Neurodevelopmental outcomes of infants born prematurely. Journal of Developmental & Behavioral Pediatrics. 2014;35(6):394-407.	Title and abstract not relevant- excluded
949.	Aylward GP. Update on neurodevelopmental outcomes of infants born prematurely. Journal of Developmental & Behavioral Pediatrics. 2014;35(6):392-3.	Title and abstract not relevant- excluded
950.	Belachew A, Tewabe T. Neonatal sepsis and its association with birth weight and gestational age among admitted neonates in Ethiopia: systematic review and meta-analysis. BMC pediatrics. 2020;20:1-7.	Title and abstract not relevant- excluded
951.	Blencowe H, Krasevec J, De Onis M, Black RE, An X, Stevens GA, et al. National, regional, and worldwide estimates of low birthweight in 2015, with trends from 2000: a systematic analysis. The Lancet global health. 2019;7(7):e849-e60.	Title and abstract not relevant- excluded
952.	Bohler E, Bergström S. Premature weaning in East Bhutan: only if mother is pregnant again. Journal of biosocial science. 1995;27(3):253-65.	Title and abstract not relevant- excluded
953.	Bøhler E, Ingstad B. The struggle of weaning: factors determining breastfeeding duration in East Bhutan. Social Science & Medicine. 1996;43(12):1805-15.	Title and abstract not relevant- excluded
954.	Breeman LD, Jaekel J, Baumann N, Bartmann P, Wolke D. Preterm cognitive function into adulthood. Pediatrics. 2015;136(3):415-23.	Title and abstract not relevant- excluded
955.	Cannavò L, Perrone S, Viola V, Marseglia L, Di Rosa G, Gitto E. Oxidative stress and respiratory diseases in preterm newborns. International journal of molecular sciences. 2021;22(22):12504.	Title and abstract not relevant- excluded
956.	Cortez J, Makker K, Kraemer D, Neu J, Sharma R, Hudak M. Maternal milk feedings reduce sepsis, necrotizing enterocolitis and improve outcomes of premature infants. Journal of Perinatology. 2018;38(1):71-4.	Title and abstract not relevant- excluded
957.	D'Onofrio BM, Class QA, Rickert ME, Larsson H, Långström N, Lichtenstein P. Preterm birth and mortality and morbidity: a population-based quasi-experimental study. JAMA psychiatry. 2013;70(11):1231-40.	Title and abstract not relevant- excluded
958.	Dewey K. Guiding principles for complementary feeding of the breastfed child. 2003.	Title and abstract not relevant- excluded

SL	Articles excluded during title and abstract screening	Decision
959.	Dong D, Ru X, Huang X, Sang T, Li S, Wang Y, et al. A prospective cohort study on lactation status and breastfeeding challenges in mothers giving birth to preterm infants. International Breastfeeding Journal. 2022;17:1-13.	Title and abstract not relevant- excluded
960.	Doyle LW, Anderson PJ. Adult outcome of extremely preterm infants. Pediatrics. 2010;126(2):342-51.	Title and abstract not relevant- excluded
961.	Edmond KM, Yousufi K, Anwari Z, Sadat SM, Staniczai SM, Higgins-Steele A, et al. Can community health worker home visiting improve care-seeking and maternal and newborn care practices in fragile states such as Afghanistan? A population-based intervention study. BMC medicine. 2018;16:1-13.	Title and abstract not relevant- excluded
962.	Ganjoo C, Rowlands R. Breast feeding and weaning practices of urban housewives in Srinagar. 1988.	Title and abstract not relevant- excluded
963.	Gao C-J, Wang F, Shen H-M, Kannan K, Guo Y. Feminine hygiene products—a neglected source of phthalate exposure in women. Environmental Science & Technology. 2019;54(2):930-7.	Title and abstract not relevant- excluded
964.	Group WIKS. Immediate “kangaroo mother care” and survival of infants with low birth weight. New England Journal of Medicine. 2021;384(21):2028-38.	Title and abstract not relevant- excluded
965.	Haider R, Rasheed S, Sanghvi TG, Hassan N, Pachon H, Islam S, et al. Breastfeeding in infancy: identifying the program-relevant issues in Bangladesh. International Breastfeeding Journal. 2010;5:1-12.	Title and abstract not relevant- excluded
966.	Ishii S, Katagiri R, Kataoka T, Wada M, Imai S, Yamasaki K. Risk assessment study of dioxins in sanitary napkins produced in Japan. Regulatory Toxicology and Pharmacology. 2014;70(1):357-62.	Title and abstract not relevant- excluded
967.	Jaekel J, Baumann N, Bartmann P, Wolke D. Mood and anxiety disorders in very preterm/very low–birth weight individuals from 6 to 26 years. Journal of Child Psychology and Psychiatry. 2018;59(1):88-95.	Title and abstract not relevant- excluded
968.	Jana A, Dey D, Ghosh R. Contribution of low birth weight to childhood undernutrition in India: evidence from the national family health survey 2019–2021. BMC Public Health. 2023;23(1):1336.	Title and abstract not relevant- excluded
969.	Jenabi E, Khazaei S, Bashirian S, Aghababaei S, Matinnia N. Reasons for elective cesarean section on maternal request: a systematic review. The Journal of Maternal-Fetal & Neonatal Medicine. 2020;33(22):3867-72.	Title and abstract not relevant- excluded
970.	KC A, Basel PL, Singh S. Low birth weight and its associated risk factors: Health facility-based case-control study. PloS one. 2020;15(6):e0234907.	Title and abstract not relevant- excluded
971.	Kim HY, Lee JD, Kim J-Y, Lee JY, Bae O-N, Choi Y-K, et al. Risk assessment of volatile organic compounds (VOCs) detected in sanitary pads. Journal of Toxicology and Environmental Health, Part A. 2019;82(11):678-95.	Title and abstract not relevant- excluded

SL	Articles excluded during title and abstract screening	Decision
972.	Kovachy VN, Adams JN, Tamaresis JS, Feldman HM. Reading abilities in school-aged preterm children: a review and meta-analysis. Developmental Medicine & Child Neurology. 2015;57(5):410-9.	Title and abstract not relevant- excluded
973.	Leon DA, Moser KA. Low birth weight persists in South Asian babies born in England and Wales regardless of maternal country of birth. Slow pace of acculturation, physiological constraint or both? Analysis of routine data. J Epidemiol Community Health. 2012;66(6):544-51.	Title and abstract not relevant- excluded
974.	Ma X, Chen Q, Pu Y, Guo M, Jiang Z, Huang W, et al. Skipping breakfast is associated with overweight and obesity: A systematic review and meta-analysis. Obesity research & clinical practice. 2020;14(1):1-8.	Title and abstract not relevant- excluded
975.	Misra A, Jayawardena R, Anoop S. Obesity in South Asia: phenotype, morbidities, and mitigation. Current obesity reports. 2019;8:43-52.	Title and abstract not relevant- excluded
976.	Moonajilin MS, Rahman ME, Islam MS. Relationship between overweight/obesity and mental health disorders among Bangladeshi adolescents: a cross-sectional survey. Obesity Medicine. 2020;18:100216.	Title and abstract not relevant- excluded
977.	Nasir AA. Factors influencing exclusive breastfeeding for the first six months among infants aged 0-12 months in Male'city, Maldives: Faculty of Health Sciences; 2015.	Title and abstract not relevant- excluded
978.	North K, Gao M, Allen G, Lee AC. Breastfeeding in a global context: epidemiology, impact, and future directions. Clinical Therapeutics. 2022;44(2):228-44.	Title and abstract not relevant- excluded
979.	Ogbo FA, Dhami MV, Awosemo AO, Olusanya BO, Olusanya J, Osuagwu UL, et al. Regional prevalence and determinants of exclusive breastfeeding in India. International breastfeeding journal. 2019;14:1-12.	Title and abstract not relevant- excluded
980.	Pokhrel HP, Pavadhgul P, Srisorrachatr S. Factors associated with exclusive breastfeeding practices in western Bhutan. Bhutan Health Journal. 2018;4(1):13-22.	Title and abstract not relevant- excluded
981.	Prentice AM. Breastfeeding in the modern world. Annals of Nutrition and Metabolism. 2022;78(Suppl. 2):29-38.	Title and abstract not relevant- excluded
982.	Putri TA, Salsabilla DA, Saputra RK. The effect of low birth weight on stunting in children under five: a meta analysis. 2021.	Title and abstract not relevant- excluded
983.	Pyhälä R, Wolford E, Kautiainen H, Andersson S, Bartmann P, Baumann N, et al. Self-reported mental health problems among adults born preterm: a meta-analysis. Pediatrics. 2017;139(4).	Title and abstract not relevant- excluded
984.	R. MP, S. P. Disclosure of Ingredients Used in Drugs, Cosmetics and Medical Products with Special Reference to Sanitary Napkins. Supremo Amicus. 2022;31:162.	Title and abstract not relevant- excluded
985.	Raheem R. A cohort study of postnatal depression, infant feeding practices and infant growth in Male', the Republic of Maldives. Acedido em https://espace curtin edu au/handle/20500. 2014;11937:1591.	Title and abstract not relevant- excluded
986.	Rahimi BA, Mohammadi E, Stanikzai MH, Wasiq AW. Determinants of exclusive breastfeeding practices in Kandahar, Afghanistan: A cross-sectional analytical study. Journal of Pediatric Perspectives. 2020;8(4):11125-40.	Title and abstract not relevant- excluded

SL	Articles excluded during title and abstract screening	Decision
987.	Roy A, Hossain MM, Hanif AAM, Khan MSA, Hasan M, Hossaine M, et al. Prevalence of infant and young child feeding practices and differences in estimates of minimum dietary diversity using 2008 and 2021 definitions: evidence from Bangladesh. Current Developments in Nutrition. 2022;6(4):nzac026.	Title and abstract not relevant- excluded
988.	Saigal S, Day KL, Van Lieshout RJ, Schmidt LA, Morrison KM, Boyle MH. Health, wealth, social integration, and sexuality of extremely low-birth-weight prematurely born adults in the fourth decade of life. JAMA pediatrics. 2016;170(7):678-86.	Title and abstract not relevant- excluded
989.	Serrao F, Papacci P, Costa S, Giannantonio C, Cota F, Vento G, et al. Effect of early expressed human milk on insulin-like growth factor 1 and short-term outcomes in preterm infants. PloS one. 2016;11(12):e0168139.	Title and abstract not relevant- excluded
990.	Shammi M, Rahman MM, Bondad SE, Bodrud-Doza M, editors. Impacts of salinity intrusion in community health: a review of experiences on drinking water sodium from coastal areas of Bangladesh. Healthcare; 2019: MDPI.	Title and abstract not relevant- excluded
991.	Sharma A, Thakur PS, Tiwari R, Kasar PK, Sharma R, Kabirpanthi V. Factors associated with early initiation of breastfeeding among mothers of tribal area of Madhya Pradesh, India: a community based cross sectional study. Int J Community Med Public Heal. 2016;3(1):194-9.	Title and abstract not relevant- excluded
992.	Shinwari I, Aminee AW, Warvadekar K, Gupta P, Chaudhery DN, Raut MK. Infant and young child feeding practices in two provinces of Afghanistan: results from two rounds of large country-lot quality assurance sampling surveys. International Journal of Community Medicine and Public Health. 2018;5(11):4761.	Title and abstract not relevant- excluded
993.	Siffel C, Kistler KD, Lewis JF, Sarda SP. Global incidence of bronchopulmonary dysplasia among extremely preterm infants: a systematic literature review. The Journal of Maternal-Fetal & Neonatal Medicine. 2021;34(11):1721-31.	Title and abstract not relevant- excluded
994.	Tariqujjaman M, Hasan MM, Mahfuz M, Ahmed T, Hossain M. Between and within-country variations in infant and young child feeding practices in South Asia. International Journal of Environmental Research and Public Health. 2022;19(7):4350.	Title and abstract not relevant- excluded
995.	Taylor SN, editor Solely human milk diets for preterm infants. Seminars in perinatology; 2019: Elsevier.	Title and abstract not relevant- excluded
996.	Tessema ZT, Tamirat KS, Teshale AB, Tesema GA. Prevalence of low birth weight and its associated factor at birth in Sub-Saharan Africa: A generalized linear mixed model. PloS one. 2021;16(3):e0248417.	Title and abstract not relevant- excluded
997.	THAN NBLTM. The global cost of not breastfeeding. 2022.	Title and abstract not relevant- excluded
998.	Tshering D, Gurung MS, Wangmo N, Pelzom D, Tejativaddhana P, Dzed L. Prevalence of exclusive breastfeeding and factors associated with exclusive breastfeeding of children in Trongsa District, Bhutan. Asia Pacific Journal of Public Health. 2018;30(4):369-77.	Title and abstract not relevant- excluded

SL	Articles excluded during title and abstract screening	Decision
999.	Victora CG, Bahl R, Barros AJ, França GV, Horton S, Krasevec J, et al. Breastfeeding in the 21st century: epidemiology, mechanisms, and lifelong effect. The lancet. 2016;387(10017):475-90.	Title and abstract not relevant- excluded
1000.	Engle-Stone R, Sununtnasuk C, Fiedler JL. Investigating the significance of the data collection period of household consumption and expenditures surveys for food and nutrition policymaking: Analysis of the 2010 Bangladesh household income and expenditure survey. Food policy. 2017;72:72-80.	Title and abstract not relevant- excluded
1001.	Engström K, Rydbeck F, Kippler M, Wojdacz TK, Arifeen S, Vahter M, et al. Prenatal lead exposure is associated with decreased cord blood DNA methylation of the glycoprotein VI gene involved in platelet activation and thrombus formation. Environmental epigenetics. 2015;1(1):dvv007.	Title and abstract not relevant- excluded
1002.	Fathima FN, Raju M, Varadharajan KS, Krishnamurthy A, Ananthkumar S, Mony PK. Assessment of 'accredited social health activists'—a national community health volunteer scheme in Karnataka State, India. Journal of health, population, and nutrition. 2015;33(1):137.	Title and abstract not relevant- excluded
1003.	Feigin VL, Stark BA, Johnson CO, Roth GA, Bisignano C, Abady GG, et al. Global, regional, and national burden of stroke and its risk factors, 1990–2019: a systematic analysis for the Global Burden of Disease Study 2019. The Lancet Neurology. 2021;20(10):795-820.	Title and abstract not relevant- excluded
1004.	Fentahun N, Mulu Y, Feleke T, Tamirat A. Nearly one in three children is suffering from sub-optimal feeding practice in Gibe District, Hadiya zone, South Ethiopia. Journal of Health, Population and Nutrition. 2020;39:1-9.	Title and abstract not relevant- excluded
1005.	Ferdous F, Das SK, Ahmed S, Farzana FD, Malek MA, Das J, et al. Diarrhoea in slum children: observation from a large diarrhoeal disease hospital in D haka, B angladesh. Tropical Medicine & International Health. 2014;19(10):1170-6.	Title and abstract not relevant- excluded
1006.	Fernandez-Concha D, Gilman RH, Gilman JB. A home nutritional rehabilitation programme in a Peruvian peri-urban shanty town (pueblo joven). Transactions of the Royal Society of Tropical Medicine and Hygiene. 1991;85(6):809-13.	Title and abstract not relevant- excluded
1007.	Filteau S, Sullivan K, Anwar U, Anwar Z, Tomkins A. Iodine deficiency alone cannot account for goitre prevalence among pregnant women in Modhupur, Bangladesh. European journal of clinical nutrition. 1994;48(4):293-302.	Title and abstract not relevant- excluded
1008.	Finigan V, Long T. Skin-to-skin contact: multicultural perspectives on birth fluids and birth 'dirt'. International nursing review. 2014;61(2):270-7.	Title and abstract not relevant- excluded
1009.	Foraita R, Klasen S, Pigeot I. Using graphical chain models to analyze differences in structural correlates of undernutrition in Benin and Bangladesh. Economics & Human Biology. 2008;6(3):398-419.	Title and abstract not relevant- excluded
1010.	Ford K. Correlation between subsequent lengths of postpartum amenorrhoea in a prospective study of breast-feeding women in rural Bangladesh. Journal of biosocial science. 1992;24(1):89-96.	Title and abstract not relevant- excluded

SL	Articles excluded during title and abstract screening	Decision
1011.	Ali S, Ali SF, Imam AM, Ayub S, Billoo AG. Perception and practices of breastfeeding of infants 0-6 months in an urban and a semi-urban community in Pakistan: a cross-sectional study. Journal of the Pakistan Medical Association. 2011;61(1):99.	Title and abstract not relevant- excluded
1012.	Al-Sahab B, Tamim H, Mumtaz G, Khawaja M, Khogali M, Afifi R, et al. Predictors of breast-feeding in a developing country: results of a prospective cohort study. Public health nutrition. 2008;11(12):1350-6.	Title and abstract not relevant- excluded
1013.	Alzaheb RA. Factors associated with the initiation of breastfeeding within the first 48 hours of life in Tabuk, Saudi Arabia. International breastfeeding journal. 2016;11:1-6.	Title and abstract not relevant- excluded
1014.	Alzaheb RA. A review of the factors associated with the timely initiation of breastfeeding and exclusive breastfeeding in the Middle East. Clinical medicine insights: pediatrics. 2017;11:1179556517748912.	Title and abstract not relevant- excluded
1015.	Amin T, Hablas H, Al Qader AA. Determinants of initiation and exclusivity of breastfeeding in Al Hassa, Saudi Arabia. Breastfeeding medicine. 2011;6(2):59-68.	Title and abstract not relevant- excluded
1016.	Asare BY-A, Preko JV, Baafi D, Dwumfour-Asare B. Breastfeeding practices and determinants of exclusive breastfeeding in a cross-sectional study at a child welfare clinic in Tema Manhean, Ghana. International breastfeeding journal. 2018;13:1-9.	Title and abstract not relevant- excluded
1017.	Asim M, Ahmed ZH, Hayward MD, Widen EM. Prelacteal feeding practices in Pakistan: a mixed-methods study. International Breastfeeding Journal. 2020;15:1-11.	Title and abstract not relevant- excluded
1018.	Atimati A, Adam V. Breastfeeding practices among mothers of children aged 1–24 months in Egor Local Government Area of Edo State, Nigeria. South African Journal of Clinical Nutrition. 2020;33(1):10-6.	Title and abstract not relevant- excluded
1019.	Banapurmath C, Nagaraj M, Banapurmath S, Kesaree N. Breastfeeding practices in villages of central Karnataka. Indian pediatrics. 1996;33:477-80.	Title and abstract not relevant- excluded
1020.	Patel A, Bucher S, Pusdekar Y, Esamai F, Krebs NF, Goudar SS, et al. Rates and determinants of early initiation of breastfeeding and exclusive breast feeding at 42 days postnatal in six low and middle-income countries: a prospective cohort study. Reproductive health. 2015;12:1-11.	Title and abstract not relevant- excluded
1021.	Pattinson R, Kerber K, Waiswa P, Day LT, Mussell F, Asiruddin S, et al. Perinatal mortality audit: counting, accountability, and overcoming challenges in scaling up in low-and middle-income countries. International Journal of Gynecology & Obstetrics. 2009;107:S113-S22.	Title and abstract not relevant- excluded
1022.	Paudel M, Javanparast S, Newman L, Dasvarma G. Health system barriers influencing perinatal survival in mountain villages of Nepal: implications for future policies and practices. Journal of Health, Population and Nutrition. 2018;37:1-19.	Title and abstract not relevant- excluded
1023.	Penafiel D, Cevallos-Valdiviezo H, Espinel R, Van Damme P. Local traditional foods contribute to diversity and species richness of rural women's diet in Ecuador. Public Health Nutrition. 2019;22(16):2962-71.	Title and abstract not relevant- excluded

SL	Articles excluded during title and abstract screening	Decision
1024.	Phillips JF, Stinson WS, Bhatia S, Rahman M, Chakraborty J. The demographic impact of the family planning--health services project in Matlab, Bangladesh. Studies in family planning. 1982:131-40.	Title and abstract not relevant- excluded
1025.	Prata N, Quaiyum MA, Passano P, Bell S, Bohl DD, Hossain S, et al. Training traditional birth attendants to use misoprostol and an absorbent delivery mat in home births. Social Science & Medicine. 2012;75(11):2021-7.	Title and abstract not relevant- excluded
1026.	Prentice A. Nutritional rickets around the world. The Journal of steroid biochemistry and molecular biology. 2013;136:201-6.	Title and abstract not relevant- excluded
1027.	Program NSotWF. Micronutrient fortification: WFP experiences and ways forward. Food and nutrition bulletin. 2006;27(1):67-75.	Title and abstract not relevant- excluded
1028.	Quaiyum A, Tunon C, Hel Baqui A, Yum ZQ, Khatun J. Impact of national immunization days on polio-related knowledge and practice of urban women in Bangladesh. Health Policy and Planning. 1997;12(4):363-71.	Title and abstract not relevant- excluded
1029.	Rah JH, Depee S, Kraemer K, Steiger G, Bloem MW, Spiegel P, et al. Program experience with micronutrient powders and current evidence. The Journal of Nutrition. 2012;142(1):191S-6S.	Title and abstract not relevant- excluded
1030.	Rah JH, Shamim AA, Arju UT, Labrique AB, Klemm RD, Rashid M, et al. Difference in ponderal growth and body composition among pregnant vs. never-pregnant adolescents varies by birth outcomes. Maternal & Child Nutrition. 2010;6(1):27-37.	Title and abstract not relevant- excluded
1031.	Islam MA, Mamun AS, Hossain MM, Bharati P, Saw A, Lestrel PE, Hossain MG. Prevalence and factors associated with early initiation of breastfeeding among Bangladeshi mothers: a nationwide cross-sectional study. PloS one. 2019 Apr 25;14(4):e0215733.	Not relevant exposure- full text excluded
1032.	Chowdhury T, Roy P, Huq O, Shaon KA. Infant and Young Child Feeding Practices among the selected Urban Working Lactating Mother, Bangladesh.	Not relevant analysis- full text excluded
1033.	Zongrone AA, Menon P, Pelto GH, Habicht JP, Rasmussen KM, Constas MA, Vermeylen F, Khaled A, Saha KK, Stoltzfus RJ. The pathways from a behavior change communication intervention to infant and young child feeding in Bangladesh are mediated and potentiated by maternal self-efficacy. The Journal of Nutrition. 2018 Feb 1;148(2):259-66.	Not relevant analysis- full text excluded
1034.	Rahman M, Yunus FM, Shah R, Jhohura FT, Mistry SK, Quayyum T, Aktar B, Afsana K. A controlled before-and-after perspective on the improving maternal, neonatal, and child survival program in rural Bangladesh: an impact analysis. PLoS One. 2016 Sep 1;11(9):e0161647.	Not relevant analysis- full text excluded
1035.	Nahar B, Ahmed T, Brown KH, Hossain MI. Risk factors associated with severe underweight among young children reporting to a diarrhoea treatment facility in Bangladesh. Journal of health, population, and nutrition. 2010 Oct;28(5):476.	Not relevant outcomes- full text excluded

SL	Articles excluded during title and abstract screening	Decision
1036.	Pagel C, Prost A, Hossen M, Azad K, Kuddus A, Roy SS, Nair N, Tripathy P, Saville N, Sen A, Sikorski C. Is essential newborn care provided by institutions and after home births? Analysis of prospective data from community trials in rural South Asia. BMC pregnancy and childbirth. 2014 Dec;14:1-9.	Not relevant exposure- full text excluded
1037.	Giashuddin MS, Kabir M, Rahman A, Hannan MA. Exclusive breastfeeding and nutritional status in Bangladesh. The Indian Journal of Pediatrics. 2003 Jun;70:471-5.	Not relevant exposure- full text excluded
1038.	Iqbal A. Knowledge and practices regarding infant and young child feeding among mothers working in readymade garments sector in bangladesh: A cross-sectional survey. Current Research in Nutrition and Food Science. 2021 Apr 1;9(1):190.	Not relevant analysis- full text excluded
1039.	Senarath U, Agho KE, Akram DE, Godakandage SS, Hazir T, Jayawickrama H, Joshi N, Kabir I, Khanam M, Patel A, Pusdekar Y. Comparisons of complementary feeding indicators and associated factors in children aged 6–23 months across five South Asian countries. Maternal & child nutrition. 2012 Jan;8:89-106.	Not relevant analysis- full text excluded
1040.	Islam MJ, Broidy L, Baird K, Rahman M, Zobair KM. Early exclusive breastfeeding cessation and postpartum depression: Assessing the mediating and moderating role of maternal stress and social support. PloS one. 2021 May 17;16(5):e0251419.	Not relevant outcomes- full text excluded
1041.	Na M, Aguayo VM, Arimond M, Narayan A, Stewart CP. Stagnating trends in complementary feeding practices in Bangladesh: An analysis of national surveys from 2004-2014. Maternal & Child Nutrition. 2018 Nov;14:e12624.	Not relevant exposure- full text excluded
1042.	Ahmed S, Parveen SD, Islam A. Infant feeding practices in rural Bangladesh: policy implications. Journal of tropical pediatrics. 1999 Feb 1;45(1):37-41.	Not relevant exposure- full text excluded
1043.	Mistry SK, Hossain MB, Irfan NM, Saha M, Saberin S, Shamim AA, Arora A. Trends in Complementary Feeding Indicators and Intake from Specific Food Groups among Children Aged 6–23 Months in Bangladesh. International Journal of Environmental Research and Public Health. 2022 Jan 4;19(1):550.	Not relevant analysis- full text excluded
1044.	Islam M, Afroja S, Biswas A, Khan MS, Khandker S. Influence of socio-demographic factors on the breastfeeding period of women in Bangladesh: a polytomous logistic regression model. Family Medicine & Primary Care Review. 2019(3):223-9.	Not relevant exposure- full text excluded
1045.	Raihana S, Alam A, Chad N, Huda TM, Dibley MJ. Delayed initiation of breastfeeding and role of mode and place of childbirth: evidence from health surveys in 58 low-and middle-income countries (2012–2017). International Journal of Environmental Research and Public Health. 2021 Jun 2;18(11):5976.	Not relevant exposure- full text excluded

SL	Articles excluded during title and abstract screening	Decision
1046.	Komatsu H, Malapit HJ, Theis S. How does women's time in reproductive work and agriculture affect maternal and child nutrition? Evidence from Bangladesh, Cambodia, Ghana, Mozambique, and Nepal.	Not relevant exposure- full text excluded
1047.	Guldan GS, Zeitlin MF, Beiser AS, Super CM, Gershoff SN, Datta S. Maternal education and child feeding practices in rural Bangladesh. Social science & medicine. 1993 Apr 1;36(7):925-35.	Not relevant exposure- full text excluded
1048.	Khatun H, Comins CA, Shah R, Munirul Islam M, Choudhury N, Ahmed T. Uncovering the barriers to exclusive breastfeeding for mothers living in Dhaka's slums: a mixed method study. International breastfeeding journal. 2018 Dec;13:1-1.	Not relevant exposure- full text excluded
1049.	Senarath U, Dibley MJ. Complementary feeding practices in South Asia: analyses of recent national survey data by the South Asia Infant Feeding Research Network. Maternal & child nutrition. 2012 Jan;8:5-10.	Not relevant analysis- full text excluded
1050.	Nessa F, Rahman S. Breast feeding patterns of working women in the Dhaka metropolitan area. Bangladesh Med Res Counc Bull. 1988.	Studies not retrieved- full text excluded
1051.	Sakib MS, Ripon Rouf AS, Tanny TF. Determinants of early initiation of breastfeeding practices of newborns in bangladesh: evidence from bangladesh demographic and health survey. Nutrition and Metabolic Insights. 2021 Oct;14:11786388211054677.	Not relevant exposure- full text excluded
1052.	Karim F, Khan AN, Tasnim F, Chowdhury MA, Billah SM, Karim T, Arifeen SE, Garnett SP. Prevalence and determinants of initiation of breastfeeding within one hour of birth: An analysis of the Bangladesh Demographic and Health Survey, 2014. PloS one. 2019 Jul 25;14(7):e0220224.	Not relevant exposure- full text excluded
1053.	Dintyala SS. A STUDY OF THE RELATIONSHIP BETWEEN MATERNAL ANTENATAL VISITATION AND ADHERENCE TO EXCLUSIVE BREASTFEEDING AT 6th MONTH.	Not relevant exposure- full text excluded
1054.	Raihana S, Dibley MJ, Rahman MM, Tahsina T, Siddique MA, Rahman QS, Islam S, Alam A, Kelly PJ, Arifeen SE, Huda TM. Early initiation of breastfeeding and severe illness in the early newborn period: An observational study in rural Bangladesh. PLoS medicine. 2019 Aug 30;16(8):e1002904.	Not relevant exposure- full text excluded
1055.	Talukder S, Farhana D, Vitta B, Greiner T. In a rural area of Bangladesh, traditional birth attendant training improved early infant feeding practices: a pragmatic cluster randomized trial. Maternal & child nutrition. 2017 Jan;13(1):e12237.	Not relevant analysis- full text excluded
1056.	Elaine AY, Thomas JS, Owais A, Tirmizi N, Faruque AS, Das SK, Rahman S, Schwartz B, Stein AD. Maternal prenatal attitudes and postnatal breast-feeding behaviours in rural Bangladesh. Public health nutrition. 2015 Mar;18(4):679-85.	Not relevant exposure- full text excluded

SL	Articles excluded during title and abstract screening	Decision
1057.	Sen KK, Mallick TS, Bari W. Gender inequality in early initiation of breastfeeding in Bangladesh: a trend analysis. International breastfeeding journal. 2020 Dec;15:1-1.	Not relevant exposure- full text excluded
1058.	Menon P, Nguyen PH, Saha KK, Khaled A, Sanghvi T, Baker J, Afsana K, Haque R, Frongillo EA, Ruel MT, Rawat R. Combining intensive counseling by frontline workers with a nationwide mass media campaign has large differential impacts on complementary feeding practices but not on child growth: results of a cluster-randomized program evaluation in Bangladesh. The Journal of nutrition. 2016 Oct 1;146(10):2075-84.	Not relevant analysis- full text excluded
1059.	Al Mamun MA, Saha S, Li J, Binta A Ghani R, Al Hasan SM, Begum A. Child feeding practices of childbearing mothers and their household food insecurity in a coastal region of Bangladesh. INQUIRY: The Journal of Health Care Organization, Provision, and Financing. 2022 Apr 15;59:00469580221096277.	Not relevant exposure- full text excluded
1060.	Komatsu H, Malapit HJ, Theis S. Does women's time in domestic work and agriculture affect women's and children's dietary diversity? Evidence from Bangladesh, Nepal, Cambodia, Ghana, and Mozambique. Food policy. 2018 Aug 1;79:256-70.	Not relevant analysis- full text excluded
1061.	Oddo VM, Ickes SB. Maternal employment in low-and middle-income countries is associated with improved infant and young child feeding. The American journal of clinical nutrition. 2018 Mar 1;107(3):335-44.	Wrong study design- full text excluded
1062.	Rahman A, Nomani D, Taneepanichskul S. Trends and determinants of EBF among adolescent children born to adolescent mothers in rural Bangladesh. International Journal of Environmental Research and Public Health. 2020 Nov;17(24):9315.	Not relevant exposure- full text excluded
1063.	Rahman M, Haque SE, Zahan S, Islam O. Noninstitutional births and newborn care practices among adolescent mothers in Bangladesh. Journal of Obstetric, Gynecologic & Neonatal Nursing. 2011 May 1;40(3):262-73.	Not relevant exposure- full text excluded
1064.	Giashuddin MS, Kabir M. Duration of breast-feeding in Bangladesh. Indian Journal of Medical Research. 2004 Jun 1;119:267-72.	Not relevant exposure- full text excluded
1065.	Das DK, Talukder MQ, Sella GE. Infant feeding practices in rural Bangladesh. The Indian Journal of Pediatrics. 1992 Sep;59:573-7.	Not relevant analysis- full text excluded
1066.	Tariqujjaman M, Hasan MM, Mahfuz M, Hossain M, Ahmed T. Association between mother's education and infant and young child feeding practices in South Asia. Nutrients. 2022 Apr 5;14(7):1514.	Not relevant exposure- full text excluded
1067.	Haider R, Begum S. Working women, maternity entitlements, and breastfeeding: a report from Bangladesh. Journal of Human Lactation. 1995 Dec;11(4):273-7.	Not relevant analysis- full text excluded

SL	Articles excluded during title and abstract screening	Decision
1068.	Ghosh R, Mascie-Taylor CN, Rosetta L. Longitudinal study of the frequency and duration of breastfeeding in rural Bangladeshi women. American Journal of Human Biology: The Official Journal of the Human Biology Association. 2006 Sep;18(5):630-8.	Not relevant analysis- full text excluded
1069.	Khan JR, Awan N, Sheikh MT. A multilevel and spatial analysis of the infant and young child feeding practices and associated factors among the under-2 aged children in Bangladesh. Child Care in Practice. 2022 Apr 3;28(2):178-95.	Not relevant analysis- full text excluded
1070.	Campbell RK, Hurley KM, Shamim AA, Shaikh S, Chowdhury ZT, Mehra S, De Pee S, Ahmed T, West Jr KP, Christian P. Effect of complementary food supplementation on breastfeeding and home diet in rural Bangladeshi children. The American journal of clinical nutrition. 2016 Nov 1;104(5):1450-8.	Not relevant exposure- full text excluded
1071.	Howlader H, Rahman A, Hasan M. Breastfeeding knowledge, attitudes and practice among rural women in Bangladesh: insights from Tungipara village. Family Medicine & Primary Care Review. 2020 Oct 1;22(4).	Not relevant analysis- full text excluded
1072.	Ahmed MS, Whitfield KC, Yunus FM. Trends and predictors of early initiation, exclusive and continued breast-feeding in Bangladesh (2004-2018): A multilevel analysis of demographic and health survey data. British Journal of Nutrition. 2022 Nov 14;128(9):1857–67.	Included
1073.	Ahmmed F, Hossain MdJ, Sutopa TS, Al-Mamun Md, Alam M, Islam MdR, et al. The trend in exclusive breastfeeding practice and its association with maternal employment in Bangladesh: A multilevel analysis. Frontiers in Public Health . 2022; 10(988016)	Included
1074.	Akter S, Rahman MdM. The Determinants of Early Cessation of Breastfeeding in Bangladesh. World Health & Population . 2010;11(4).	Included
1075.	Akter S, Rahman MM. Duration of Breastfeeding and Its Correlates in Bangladesh. Journal of Health, Population and Nutrition . 2010;28(6).	Included
1076.	Ali NB, Tahsina T, Emdadul Hoque DM, Hasan MM, Iqbal A, Huda TM, et al. Association of food security and other socioeconomic factors with dietary diversity and nutritional statuses of children aged 6-59 months in rural Bangladesh. PLoS One. 2019 Aug 1;14(8).	Included
1077.	Ayesha U, Mamun ASMA, Sayem MA, Hossain MG. Factors associated with duration of breastfeeding in Bangladesh: evidence from Bangladesh demographic and health survey 2014. BMC Public Health. 2021 Dec 1;21(1).	Included
1078.	Basnet S, Frongillo EA, Nguyen PH, Moore S, Arabi M. Associations of maternal resources with care behaviours differ by resource and behaviour. Matern Child Nutr. 2020 Jul 1;16(3).	Included
1079.	Blackstone S, Sanghvi T. A comparison of minimum dietary diversity in Bangladesh in 2011 and 2014. Matern Child Nutr. 2018 Oct 1;14(4).	Included
1080.	Blackstone SR, Sanghvi T. Predictors of exclusive breastfeeding across three time points in Bangladesh: An examination of the 2007, 2011 and 2014 Demographic and Health Survey. Int Health. 2018 May 1;10(3):149–56.	Included

SL	Articles excluded during title and abstract screening	Decision
1081.	Haider R, Thorley V. Supporting Exclusive Breastfeeding Among Factory Workers and Their Unemployed Neighbors: Peer Counseling in Bangladesh. Journal of Human Lactation. 2019 Aug 1;36(3):414–25.	Included
1082.	Hasan M, Hassan MN, Khan MSI, Tareq MA, Afroj MS. Prevalence, knowledge, attitudes and factors associated with exclusive breastfeeding among mothers in Dhaka, Bangladesh: A cross-sectional study. Popul Med. 2021 Sep 1;3:1–7.	Included
1083.	Hasan M, Hassan MdN, Khan MSI, Al Banna MdH. Prevalence and Determinants of Early Initiation of Breastfeeding Among Mothers in Dhaka City, Bangladesh: a Cross-sectional Study. SN Compr Clin Med. 2020 Dec;2(12):2792–8.	Included
1084.	Hossain M, Islam A, Kamarul T, Hossain G. Exclusive breastfeeding practice during first six months of an infant's life in Bangladesh: A country based cross-sectional study. BMC Pediatr. 2018 Mar 2;18(1).	Included
1085.	Kabir I, Khanam M, Agho KE, Mihrshahi S, Dibley MJ, Roy SK. Determinants of inappropriate complementary feeding practices in infant and young children in Bangladesh: Secondary data analysis of Demographic Health Survey 2007. Matern Child Nutr. 2012 Jan;8(SUPPL. 1):11–27.	Included
1086.	Jain AK, Bongaarts J. Breastfeeding: Patterns, Correlates, and Fertility Effects. Family Planning [Internet]. 1981;12(3):79–99. Available from: http://www.jstor.orgURL:http://www.jstor.org/stable/1966370Accessed:07-12-201518:52UTC	Included
1087.	Khan JR, Sheikh MT, Muurlink O. Breastfeeding termination and its determinants in Bangladesh: current status data modelling. Early Child Dev Care. 2019;190(16):2594–604.	Included
1088.	Kundu S, Sayeed A, Gedef Azene A, Rezyona H, Al Banna MH, Shafiqul M, et al. Exploring the factors associated with dietary diversity of children aged 6-59 months in some rural and slum areas of Bangladesh amid the COVID-19 pandemic: A mixed-effect regression analysis. Current Developments in Nutrition . 2022;6(8).	Included
1089.	Mihrshahi S, Kabir I, Roy SK, Agho KE, Senarath U, Dibley MJ. Determinants of infant and young child feeding practices in Bangladesh: Secondary data analysis of Demographic and Health Survey 2004. Food Nutr Bull. 2010;31(2).	Included
1090.	Nguyen PH, Avula R, Ruel MT, Saha KK, Ali D, Tran LM, et al. Maternal and child dietary diversity are associated in bangladesh, vietnam, and ethiopia. Journal of Nutrition. 2013 Jul 1;143(7):1176–83.	Included
1091.	Rahman MA, Khan MN, Akter S, Rahman A, Alam MM, Khan MA, et al. Determinants of exclusive breastfeeding practice in Bangladesh: Evidence from nationally representative survey data. PLoS One. 2020 Jul 1;15(7).	Included
1092.	Raihana S, Alam A, Huda TM, Dibley MJ. Factors associated with delayed initiation of breastfeeding in health facilities: secondary analysis of Bangladesh demographic and health survey 2014. Int Breastfeed J. 2021 Dec 1;16(1).	Included

SL	Articles excluded during title and abstract screening	Decision
1093.	Rana MM, Islam MR, Karim MR, Islam AZ, Haque MA, Shahiduzzaman M, et al. Knowledge and practices of exclusive breastfeeding among mothers in rural areas of Rajshahi district in Bangladesh: A community clinic based study. PLoS One. 2020 May 1;15(5).	Included
1094.	Rasheed S, Frongillo EA, Devine CM, Alam DS, Rasmussen KM. Maternal, infant, and household factors are associated with breast-feeding trajectories during infants' first 6 months of life in Matlab, Bangladesh. Journal of Nutrition. 2009 Aug;139(8):1582–7.	Included
1095.	Sheikh N, Akram R, Ali N, Haque SR, Tisha S, Mahumud RA, et al. Infant and young child feeding practice, dietary diversity, associated predictors, and child health outcomes in Bangladesh. Journal of Child Health Care. 2019 Jun 1;24(2):260–73.	Included
