# Supplementary material for: How is maternal employment associated with infant and young child feeding in Bangladesh? A systematic literature review and meta-analysis
Source: PLoS One. 2025 Jan 24;20(1):e0316436. doi: 10.1371/journal.pone.0316436 (PMC11759378; doi:10.1371/journal.pone.0316436)
Supplement: Attachment — Submitted filename: Response to reviewers.docx [file pone.0316436.s002.docx]

**Response to editor and reviewers’ comments**

Highlighted phrases refer to the text in the revised manuscript.

**Response to editor’s comments**

| **1** | **Editor’s comment:** Line 45, add space between 0.32 and (  **Authors’ response:** Thank you.  **Action taken:** Edited accordingly (L45). “0.32 (95% CI:0.16, 0.67; p = 0.002)” |
| --- | --- |
| **2** | **Editor’s comment:** Line 163, add reference to the Newcastle Ottawa Scale.  **Authors’ response:** Thank you.  **Action taken:** Reference provided (L156). “The Newcastle-Ottawa Scale (NOS) was used to assess the quality of the included articles (26).” |
| **3** | **Editor’s comment:** Line 186, explain rationale for publication period.  **Authors’ response:** This publication period refers to the years of publication of the included studies.  **Action taken:** We did not put any time frame for the published articles when selecting studies, which we mentioned accordingly (L109).  “(3) publication time: no limit on publication period was considered since no similar study was conducted in Bangladesh context” |
| **4** | **Editor’s comment:** Line 237, no need to include year of publication when using Vancouver. The same is true later in manuscript, e.g., lines 267, 296, 353. Please check throughout and remove the blue hyperlinks.  **Authors’ response:** Thanks for the suggestions.  **Action taken:** The correction has been done throughout the manuscript. Blue color letters are not hyperlink texts and were applied to easily identify the references used. However, only dark font was used in the revised version. |
| **5** | **Editor’s comment:** Line 279, mention that, of the 3 figures, only one was statistically significant.  **Authors’ response:** Thank you.  **Action taken:** These 3 figures represent pooled OR of 3 infant and young child feeding indicators among employed mothers compared to their counterparts. The significance values have been mentioned as suggested (L252).  “0.79 (95% CI: 0.49, 1.27; p = 0.33), 0.32 (95% CI: 0.16, 0.67; p = 0.002), 1.07 (95% CI: 0.81, 1.42; p = 0.63), respectively (Fig 2-4)” |
| **6** | **Editor’s comment:** Line 361, the UNICEF and WHO recommendations are up to 2 years and beyond. The 'and beyond' is inclusive of these time periods. In other words, longer breastfeeding should not be construed as potentially negative. I understand you were pointing to what the authors deemed 'adequate,' but I suggest looking at how this sentence is written.  **Authors’ response:** Thanks for the suggestion.  **Action taken:** The sentence has been rephrased with proper justification (L337-339). “Therefore, the effect estimate could have been different if minimum recommended duration by the WHO and UNICEF was considered, as commonly used in other studies (66).” |
| **7** | **Editor’s comment:** Lines 383-4, specify what current maternity policies are in Bangladesh (this can be presented earlier if desired).  **Authors’ response:** Thank you.  **Action taken:** The major policy concerns have been described (L377-380). “In brief, those policies emphasize on enabling environment for breastfeeding at workplace, promotion and protection of breastfeeding and complementary feeding practices, quality implementation of baby friendly hospital initiatives, increase the duration of maternity leave, and mandate to mainstream IYCF (67).” |
| **8** | **Editor’s comment:** Line 288, provide further background on existing nutrition efforts (can be presented earlier in background).  **Authors’ response:** Thank you.  **Action taken:** According to PDF (initial submission), Line 288 in refers to Figure 4. The figure has been described accordingly in the revised manuscript (L246-256). Furthermore, in the background section the relationship between maternal employment and complementary feeding practice has been mentioned. However, as our aim was to observe the overall association, individual nutrition programs targeting complementary feeding were not elaborated.  “In Bangladesh, child feeding is usually performed by mothers (8), whereas family food choice is primarily decided by fathers (9). Therefore, child feeding at the family level, especially complementary feeding, is mostly dependent on paternal food preference (9). However, this may be different where mothers are in employment. Employed mothers can independently spend earnings on food for their children and therefore diversify food intake, regardless of their husbands’ income (10).” |

**Response to comments from Reviewer #1**

| **1** | **Reviewer’s comment:** Introduction, Line 60: Could the authors provide some context on how the statistics quoted compared to other similar countries? This will help readers understand the position of Bangladesh within the global context.  **Authors’ response:** Thank you.  **Action taken:** Some statistics from neighboring countries are provided (L60-61). “Meanwhile, 31% of children aged under five years are stunted, and 22% are underweight, which are similar with neighboring countries, including India (36% stunting and 32% underweight) and Nepal (25% stunting and 19% underweight) (3,4).” |
| --- | --- |
| **2** | **Reviewer’s comments:** Introduction, Line 72: The reference used (#9) is about Ethiopia, not Bangladesh. Can the authors replace this with a reference relevant to the study population? If not, perhaps rework the sentence, for example, “Researchers have shown in LMIC employed mothers …”  **Authors’ response:** Thank you.  **Action taken:** The sentence has been re-phrased accordingly (L71-72). “Studies conducted in lower middle-income countries (LMICs) have shown that employed mothers have better knowledge of nutrition and child feeding, can access and interpret nutritional information (11).” |
| **3** | **Reviewer’s comments:** Methods, Lines 126-129: These two sentences seem to contradict each other on the data extraction process. Can the authors please review and edit to ensure clarity?  **Authors’ response:** Thank you.  **Action taken:** The sentences have been edited accordingly (L121-123). “For each of the included records, data was extracted by two of the five reviewers working independently (TA, IJR, SSA, PS, and MAR). One reviewer (MAR) conducted cross-checking of the extracted data and resolved conflicts through mutual discussion.” |
| **4** | **Reviewer’s comments:** Methods, Lines 139-140: The authors introduce MDD and MAD with little explanation of this terminology. Can the authors please provide more information about this terminology?  **Authors’ response:** Thank you.  **Action taken:** A brief description and reference to the MDD and MAD has been provided (L131-135). “Both minimum dietary diversity (MDD), an indicator to understand the diet quality, referring to the presence of food items at least from five different food groups out of eight food groups according to latest definition, and minimum acceptable diet (MAD), MDD plus recommended daily frequency of the diet, were accepted as complementary feeding indicators (23).” |
| **5** | **Reviewer’s comments:** Methods: Can the authors clarify whether the included papers defined the duration of exclusive breastfeeding in line with WHO recommendations?  **Authors’ response:** Thank you.  **Action taken:** Definition of exclusive breastfeeding according to WHO has been clarified (L130). “2) exclusive breastfeeding until six months of age as recommended by WHO.” |
| **6** | **Reviewer’s comments:** Results, Line 208: I cannot see a Table 3. Does Table 4 need to be renumbered as Table 3?  **Authors’ response:** Thank you for noticing the misreporting.  **Action taken:** The three tables have been numbered accordingly. |
| **7** | **Reviewer’s comments:** Results, Lines 218-271: Much of this 2.5 pages of written content repeats the results presented in the forest plots. Can the authors condense the results by briefly commenting on the frequently contradictory nature of the included papers’ results, and then focusing on the pooled estimates in the written text, rather than repeating results for individual studies?  **Authors’ response:** Thank you for the useful suggestion. The narrative part of the result section has been condensed accordingly.  **Action taken:** The explanation of the pooled estimate was elaborated (L211-258). “In total, 4 effect sizes for early initiation of breastfeeding, 7 effect sizes for exclusive breastfeeding, and 5 effect sizes for complementary feeding were considered for meta-analysis. The majority of effects were calculated as OR. Analysis found the pooled odds ratios for providing early initiation of breastfeeding, exclusive breastfeeding, and complementary feeding among employed mothers to be 0.79 (95% CI: 0.49, 1.27; p = 0.33), 0.32 (95% CI: 0.16, 0.67; p = 0.002), 1.07 (95% CI: 0.81, 1.42; p = 0.63), respectively (Fig 2-4). A high level of heterogeneity was found across all three meta-analyses (I^2^ = > 80%). Furthermore, publication bias was also detected (S2 Appendix). As identified by leave-one-out meta-analysis, the stability of the overall effect of maternal occupation on EIBF and CF were found to be affected by individual effect, indicating a week stability of the pooled effect size; however, such effect was not observed for EBF (S2 Appendix).” |
| **8** | **Reviewer’s comments:** Discussion, Lines 318-9: The authors state ‘working mothers were found to have more control over food choices due to improved access through income” however it is not clear to me that the data presented in the Results section supports this statement. Can the authors please provide a clear link between the results and this statement?  **Authors’ response:** We explained why maternal employment could play as both protective and risk factor of complementary feeding, providing circumstantial evidence. Although the pooled effect provides a summary of estimates, we believe it will be good describe when the situation differed.  **Action taken:** The phrases have been edited accordingly (L300-306). “However, a positive association between maternal employment and better child feeding practices is not always guaranteed. Mothers who were employed but with a low salary and engaged in informal and poor working conditions were at higher risk of failing to secure adequate childcare (62). Therefore, children whose mothers were working in unsuitable workplace environments, were paid low salaries, or were engaged in the informal sectors experienced poor complementary feeding (31,32,42,63). These justifications comply with findings from studies further afield, such as in South Africa (64).” |
| **9** | **Reviewer’s comments:** Discussion, Lines 322-25: It is unclear whether the authors are referring to their own results or results from other papers. The authors need to reframe their writing to make this clear to the author and use correct tense (were vs. are). For example, “In contrast, it has been shown mothers who are employed …. are at higher risk …”  **Authors’ response:** Thanks for the suggestion. The interpretation refers to Bangladesh context, so evidence from included studies has been brought up because a systematic review contains all relevant evidence. However, we have brought up evidence from another country as well.  **Action taken:** The grammatical issue has been corrected accordingly (L301-304). “Mothers who were employed but with a low salary and engaged in informal and poor working conditions were at higher risk of failing to secure adequate childcare (62). Therefore, children whose mothers were working in unsuitable workplace environments, were paid low salaries, or were engaged in the informal sectors experienced poor complementary feeding (31,32,42,63). These justifications comply with findings from studies further afield, such as in South Africa (64).” |
| **10** | **Reviewer’s comments:** Discussion, Line 368: Can the authors clarify this potential calculation error in more detail and how they came to determine this potential error explanation may have occurred?  **Authors’ response:** Thanks, the issue has been outlined.  **Action taken:** The reason behind the calculation error has been outlined (L345-348). “This could be a result of including a mistaken number of children under six months of age, which was due to excluding mothers who had children less than six months of age, as the denominator when calculating the rate of exclusive breastfeeding.” |
| **11** | **Reviewer’s comments:** Discussion, Line 370: The Policy Implications Section reads as if it should come at the end of the paper, leading into the Conclusions. I would recommend the authors consider moving the Limitations Section to after the Study Quality but before the Policy Implications Section. This will improve the flow of the paper.  **Authors’ response:** Thanks.  **Action taken:** The sections have been re-ordered accordingly. |
| **12** | **Reviewer’s comments:** Discussion, Line 408: The authors introduce abbreviations (EIBF, EBF, CF) which have not been previously defined or used elsewhere in the paper. Can the authors either write the abbreviations out in full, or define these earlier and use throughout?  **Authors’ response:** Thank you for pointing out these.  **Action taken:** The abbreviations have been elaborated when they first appeared in the manuscript (L212, L216, L233). |
| **13** | **Reviewer’s comment:** General grammar feedback:  - Numbers under 10 should be written as words, not numerals.  - Blue font appears in some sections of the paper.  **Authors’ response:** Thanks for the suggestions!  **Action taken:** The manuscript has been thoroughly crosschecked, and the issues have been addressed. Blue font has been changed to dark font. |

**Response to comments from Reviewer #2**

| **1** | **Reviewer’s comment:** The data processing seems in order, the conclusion can be re-written as the current conclusion is too broad and weak, given the strength of the evidence presented. Strengthen to conclusion to speak directly to the findings. Employed women were found to be less likely to initiate breastfeeding, this calls for improved maternity protection and support for employed women. Employed women were less likely to exclusively breastfeed, this calls for stronger workplace support and breastfeeding protection. CF support needs to be implemented for all children, irrespective of the mother's employment status.  **Authors’ response:** Thank you for very constructive feedback and direction how to restructure the conclusion.  **Action taken:** The conclusion has been narrowed down and re-written according to the findings from meta-analysis in the abstract and conclusion section of the revised manuscript.  “Conclusion in abstract: Maternal employment appears not to be a protective factor for some important breastfeeding indicators in Bangladeshi context; therefore, this issue should be taken into consideration when formulating relevant policies.  Conclusion section: The association between maternal employment and IYCF indicators in Bangladesh not appears to be homogenous, maternal employment being a protective factor for complementary feeding practices but a risk factor for early breastfeeding initiation and exclusive breastfeeding. Support through policy and its implementation should be provided to employed mothers to allow them to practice exclusive breastfeeding during the first six months. Similarly, support should be given to unemployed mothers to allow for improved complementary feeding practices.” |
| --- | --- |

**Response to comments from Reviewer #3**

| **1** | **Reviewer’s comment:** The paper is well designed and very well written. There is a consistency between the study aim with sections of the manuscript. The sections of the manuscript are consistent to the study aim and written with clarity and concision. The systematic review process has been followed and realized. However, the manuscript should be edited and more details on the results sections are needed.  **Authors’ response:** Thanks for the nice comments.  **Action taken:** The manuscript has been revised and edited after taking your suggestions into careful consideration. |
| --- | --- |
| **2** | **Reviewer’s comment:** Methods section should be strengthened by in-text citations. The meaning of effects size estimates and their significance should be verified in the whole text.  **Authors’ response:** Thank you.  **Action taken:** We have provided additional references to strengthen the justification of methods, especially for leave-one-out meta-analysis, publication bias, variable selection for meta-analysis, and PRISMA guideline, considered in this study.  “This systematic review was registered to PROSPERO with registration ID CRD42023408507. Preferred Reporting Items for Systematic Review and Meta-Analysis (PRISMA) was considered as the reporting guideline (S1 Checklist) (22).  Minimum acceptable diet (MAD), MDD plus recommended daily frequency of the diet, were accepted as complementary feeding indicators (23).  Furthermore, leave-one-out meta-analysis was carried out to observe how the pooled effect size was affected due to the omission of any of the included studies (24) . Meta funnel plot was depicted to observe the small study effect or publication bias (25) . The statistical software package STATA (version: 17.0 BE-Basic Edition) was used for conducting the meta-analysis.  The Newcastle-Ottawa Scale (NOS) was used to assess the quality of the included articles (26).” |
| **3** | **Reviewer’s comment:** Abstract: Lines 29-30. The authors should pay attention to the accuracy at citing the variables in the study aim. Please, rephrase that section of the sentence: ‘…to investigate the association between these variables.  **Authors’ response:** Thank you.  **Action taken:** The sentence has been rephrased (L29-30). “The aim of this systematic review and meta-analysis is to investigate the association between these variables.” |
| **4** | **Reviewer’s comment:** Line 34. This sentence ‘Findings were presented in a summary table’ doesn’t add any value to the abstract, I will suggest the authors delete it.  **Authors’ response:** Thank you.  **Action taken:** The sentence has been deleted. |
| **5** | **Reviewer’s comment:** Many studies have been conducted on the topic. The novelty of the manuscript might be to mention the study period and the study setting to show the specificity of the study.  **Authors’ response:** Thank you for the suggestions. All these issues are addressed in different sections.  **Action taken:** Rationale is provided in the introduction section (L83-92), included studies publication period is mentioned in the inclusion and exclusion criteria (L106-109), settings are presented in Table 1, and methodical strength is presented in the strengths and limitation section (L348-355). |
| **6** | **Reviewer’s comment:** Introduction  • Lines 58-60. Replace the in-text citation to the end of the sentence to support the whole sentence.  **Authors’ response:** Thank you.  **Action taken:** The correction has been made accordingly (L59). “In Bangladesh, 65% of infants aged less than 6 months are exclusively breastfed and only 34% of children aged 6-23 months receive recommended complementary feeding (2).” |
| **7** | **Reviewer’s comment:** Line 85. The authors should rephrase this sentence: ‘…between increases in maternal employment and IYCF practices has yet to be reviewed.’ I suggest: ‘…between in maternal employment status and IYCF practices has yet to be reviewed.’  **Authors’ response:** Thank you.  **Action taken:** The editing has been done according to suggestion (L86-87). “However, the association between maternal employment status and IYCF practices has yet to be reviewed.” |
| **8** | **Reviewer’s comment:** Line 87. The metanalysis provides the consensus on the associated pooled data and not a spurious idea on the relationship. The authors can use the term “consensus” instead of “an idea”.  **Authors’ response:** Thanks for the suggestion.  **Action taken:** The more appropriate word has been used (L89). “Meta-analysis will provide a consensus of the individual associations between maternal employment and IYCF practices over the years.” |
| **9** | **Reviewer’s comment:**  Methodology • Selection process section (Lines 114-123). The manuscript seems to have two eligibility criteria: the first one on lines 103-111 and the second on line 118-123. We should recommend the authors to use one eligibility criteria based on the PECO acronym for the whole text. Also, this eligibility criteria should include the period where data has been collected.  **Authors’ response:** Thanks!  **Action taken:** The eligibility criteria have been presented in one paragraph (L106-111). “In addition to the PECO framework, the following inclusion criteria were implemented: (1) study design: observational because the study aim is to observe the association between the variables of interest in usual settings; (2) study objective: investigating factors associated with IYCF practices in Bangladesh; (3) publication time: no limit on publication period was considered since no similar study was conducted in Bangladesh context; (4) publication language: English; (5) association: maternal employment and IYCF practices.” |
| **10** | **Reviewer’s comment:** Please, edit the format of the sentence: ‘… title and abstract were…’ instead of ‘… title and abstract was…’  **Authors’ response:** Thank you.  **Action taken:** The format of the sentence has been edited accordingly (L115). “The title and abstract were screened by at least two of the six reviewers (FA, TA, IJR, SSA, PS, and MAR), and conflicts were independently resolved by a third reviewer (MAR, PS, and SS).” |
| **11** | **Reviewer’s comment:** Data extraction section. The are many resources (citations) that can support the data extraction and very interesting o be cited. Among the variables that have been extracted ion the data extraction table, the title of each primary article doesn’t need to display.  **Authors’ response:** The PRISMA guidelines were followed during screening and selection of the articles. We followed the same strategy while data extraction, at least two reviewers being involved in data extraction and cross-checking.  **Action taken:** We have cited the PRISMA guideline accordingly (L96). “This systematic review was registered to PROSPERO with registration ID CRD42023408507. Preferred Reporting Items for Systematic Review and Meta-Analysis (PRISMA) was considered as the reporting guideline (S1 Checklist) (22).” |
| **12** | **Reviewer’s comment:** Data synthesis and metanalysis section.   - The section should need more in-text citations to support the review process particularly for data synthesis, meta-analysis, funnel plot. - Criteria for including primary articles in the meta-analysis needs to be clearly defined in this section. For instance, the definition of variables were they the same for all primary articles that have been included in the metanalysis?   **Authors’ response:** Thanks for the important suggestions.  **Action taken:**   - The appropriate references to funnel plot and leave-one-out meta-analysis have been provided (L152-153) - Three variables were considered for meta-analysis. These are early initiation of breastfeeding, exclusive breastfeeding, and complementary feeding. All three variables were measured following standard guidelines provided by WHO, which we mentioned accordingly (L129-154)   “Furthermore, leave-one-out meta-analysis was carried out to observe how the pooled effect size was affected due to the omission of any of the included studies (24) . Meta funnel plot was depicted to observe the small study effect or publication bias (25) . The statistical software package STATA (version: 17.0 BE-Basic Edition) was used for conducting the meta-analysis.  Both minimum dietary diversity (MDD), an indicator to understand the diet quality, referring to the presence of food items at least from five different food groups out of eight food groups according to latest definition, and minimum acceptable diet (MAD), MDD plus recommended daily frequency of the diet, were accepted as complementary feeding indicators (23).” |
| **13** | **Reviewer’s comment:**  • Quality assessment and risk assessment.  o This section should chronologically display after the data synthesis before the metanalysis. The quality of data is one of the criteria for review.  **Authors’ response:** We have combined data synthesis and meta-analysis into one paragraph/section; therefore, quality assessment and assessing the risk of bias section was placed after that.  **Action taken:** Quality assessment and assessing the risk of bias section was placed after the data synthesis and meta-analysis section. |
| **14** | **Reviewer’s comment:**  Results Figure 1:   - The computation of numbers in the whole flow diagram needs more correctitude and consistency. All databases have searched for 1865 primary articles instead of 1971 that have been mentioned in the flow diagram. The authors should spell out the search strategy in the manuscript for transparency and reproducibility of the methods. Also, the citation for the flow diagram can be inserted in the figure comments.   **Authors’ response:** Thanks for the very useful feedback.  **Action taken:** We have cross-checked the digits and calculations in figure 1 and made necessary corrections. |
| **15** | **Reviewer’s comment:** The cell ‘Studies for retrieval’ should display 67 articles instead of 65 as reflected in the flow diagram.  **Authors’ response:** Thank you.  **Action taken:** The correction was made accordingly. |
| **16** | **Reviewer’s comment:**  The reasons of exclusion of primary articles need to be mentioned for more accuracy in the review process. ‘wrong exposure’ or ‘wrong outcomes’ seem to be inappropriate terms to express the exclusion criteria. Terms as ‘not relevant exposure or not relevant outcomes’ refer to the eligibility criteria and are a bit more appropriate.  **Authors’ response:** Thank you.  **Action taken:** Figure 1 has been edited accordingly. The suggested phrases have been used. |
| **17** | **Reviewer’s comment:** Figure 2 to 4:  o Only one study (Hans, 2020) has demonstrated a protective relationship between maternal employment status and the IYCF.  o the summary of the review should better explore these sections. The pooled data should better be summarized in the first paragraph of the discussion instead of the summary from primary articles. For illustration, data can show the positive association between exclusive breastfeeding, complementary feeding and maternal employment status while no significant relationship between maternal employment and initiative breastfeeding.  **Authors’ response:** Thanks for the useful suggestions.  **Action taken:**  Figures 2 to 4 have been cross checked and significance values have been mentioned in the text (L252-253).  The summary of the meta-analysis has been put in the first paragraph of the discussion and individual study effects were removed accordingly.  “Analysis found the pooled odds ratios for providing early initiation of breastfeeding, exclusive breastfeeding, and complementary feeding among employed mothers to be 0.79 (95% CI: 0.49, 1.27; p = 0.33), 0.32 (95% CI: 0.16, 0.67; p = 0.002), 1.07 (95% CI: 0.81, 1.42; p = 0.63), respectively (Fig 2-4).  While the pooled association demonstrated maternal employment to be negatively associated with recommended breastfeeding practices, maternal employment was also found to be a protective factor for recommended complementary feeding practices.” |
| **18** | **Reviewer’s comment:**  • Table 1:   - The heading in table 1, column 2 need to be edited. Please, can the authors mention ‘Author, year’ instead of ‘study’? - For better understanding of the table 1, I would recommend the authors to add another column beside of the entitled column ‘geographical area variable’. It will better sound to segregate data based on ‘National vs subnational’ for the first column, and the second column where ‘rurality variable’ should include ‘rural vs urban vs urbano-rural’. - Demographic data for participants (mother/caregiver, children) might add value to the review.   **Authors’ response:** Thanks for the suggestion.  **Action taken:**  The heading of column 2 has been changed to ‘author, year’ (Table 1).  Considering geographical area variable, all national and multi-district level studies covered both rural and urban areas. For studies covering a single district or similar cluster, we have specified if the area was rural, urban, or both. Therefore, another column for demographic area variable was not inserted (Table 1).  Demographic information (age and education level) of mothers or caregivers has been incorporated in Table 1. Educational attainments have been mentioned in the footnote. |
| **19** | **Reviewer’s comment:**  o On line 264, how confident are the authors to the results? Please, check for the confidence interval? Thew authors should check for the significance of the relationship between maternal employment status and IYCF. Check for the effects size estimates on lines 224, 237, 260, and 278.  **Authors’ response:** Thanks for the suggestion. The association refers to the original article where confidence interval was not reported. So, confidence interval was not mentioned, however, the statement has been rephrased.  **Action taken:** The significance level has been accordingly mentioned for all the pooled associations throughout the manuscript.  “Analysis found the pooled odds ratios for providing early initiation of breastfeeding, exclusive breastfeeding, and complementary feeding among employed mothers to be 0.79 (95% CI: 0.49, 1.27; p = 0.33), 0.32 (95% CI: 0.16, 0.67; p = 0.002), 1.07 (95% CI: 0.81, 1.42; p = 0.63), respectively (Fig 2-4).” |
| **20** | **Reviewer’s comment:**  • Limitations section. On lines 401-404, four databases that were included in the review stand for a strength of the review instead of the weakness.  **Authors’ response:** Thanks for the suggestion.  **Action taken:** The strengths and limitations section has been edited accordingly (L350-357)  “Four common databases were selected for literature search.” |
| **21** | **Reviewer’s comment:**  References • Repetition of the websites for the first citation number 1 should be avoided.  • The format of references needs some editing. The use of capital letters for the titles of citations needs to be edited: citations number 17, 19, 23, 28 and 30.  **Authors’ response:** Thanks for the suggestions.  **Action taken:** Repetition of the cited website has been corrected.  Reference section has been formatted by using reference manager ‘Mendeley’. |
