# Supplementary material for: How is maternal employment associated with infant and young child feeding in Bangladesh? A systematic literature review and meta-analysis
Source: PLoS One. 2025 Jan 24;20(1):e0316436. doi: 10.1371/journal.pone.0316436 (PMC11759378; doi:10.1371/journal.pone.0316436)
Supplement: Attachment — Submitted filename: Response to reviewers.docx [file pone.0316436.s003.docx]

**Response to academic editor and reviewers’ comments**

Highlighted phrases refer to the text in the revised manuscript.

**Response to academic editor’s comments**

| **1** | **Academic editor’s comment:**  I would firstly like to thank you for your patience. Securing reviewers proved to be a challenging task after several original reviewers indicated unavailability.  This systematic review provides valuable insights for the Bangladesh context about how employment may impact on breastfeeding and complementary feeding. For the most part, an appropriate level of detail is provided around the methodology to enable repetition. That said, there are a few minor corrections that are required.  Regarding Reviewer 1's comments specific to updating the search and the timing of data collection, as long as the endpoint of the search is clearly denoted and timing is noted in limitations, there is no expectation that you need to re-do the search and update the analysis as this late stage in the manuscript review process. Similarly, regarding the suggestion on Table 1, you can note the study design in the first column instead of creating an entirely new column if you face space constraints.  Once the remaining points are addressed, we should be able to proceed.  **Authors’ response:** Thank you for providing us with an opportunity to revise the manuscript. We understand the challenges you faced while securing the required number of reviewers.  At this point, we highly appreciate your suggestion regarding Reviewer 1’s comments specific to updating the search. As we have clearly mentioned the endpoint of literature search and highlighted this in the limitation section, we are not updating the search. Furthermore, as our emphasis was to review the studies conducted based on nationally representative data, the availability of new studies is unlikely given that the latest Bangladesh Demographic and Health Survey data is not publicly available yet. Also thank you for your suggestion concerning Table 1.  **Action taken:** The limitation regarding the timeframe of literature search is highlighted in the manuscript as follows.  “As the duration of database search was limited until March 2023, consideration of including any records published beyond that timeframe might result in changes in the pooled associations.” (L364-366)  As you suggested, we have revised Table 1 and incorporated the study design information in a new column (Table 1). We have addressed all the comments from reviewers that are presented below. |
| --- | --- |

**Response to comments from Reviewer #1**

| **1** | **Reviewer’s comment:** Methods, Search Strategy, Line 100: The database searches are now out of date (over 12 months ago). These need to be updated prior to publication. In addition, academic rigor requires all searches to be performed on the same date.  **Authors’ response:** Thank you for your suggestion. According to academic editor, at this point, updating literature search might not be mandatory subject to mentioning the timeframe of literature search as a limitation, which we have done. It was the review process which took a long time and caused the timeframe of database search being out of date. Furthermore, as our emphasis was to review the studies conducted based on nationally representative data, the availability of new studies is unlikely given that the latest Bangladesh Demographic and Health Survey data is not publicly available yet. So, considering all of these, we have not updated the literature search. Thank you in advance for your consideration.  **Action taken:** The limitation regarding the timeframe of literature search is highlighted in the manuscript as follows.  “As the duration of database search was limited until March 2023, consideration of including any records published beyond that timeframe might result in changes in the pooled associations.” (L364-366) |
| --- | --- |
| **2** | **Reviewer’s comments:** Methods, Inclusions and Exclusion Criteria, Lines 102-111: Most of this section would be better presented in a table. The last sentence is incomplete.  **Authors’ response:** Thank you for your suggestion. We have restructured the paragraph and provided concise information about inclusion and exclusion criteria. However, we have not presented the inclusion and exclusion criteria in a Table because the exclusion criteria for exposure seems to be the comparator (mothers without employment) in this case. So, the information might be repeating, leading to a not good readability. Therefore, we concisely highlighted the inclusion and exclusion criteria in a narrative manner.  **Action taken:** The paragraph has been edited as follows.  “The PECO framework was used to determine record inclusion as follows: population (P) = mothers having an infant or a young child in Bangladesh; exposure (E) = mothers being employed in any public or private sector jobs; comparator (C) = mothers without employment; and outcome (O) = status of IYCF practices. Only observational studies were considered for inclusion because the current study aims to observe the association between the maternal occupation and IYCF practices in real-world settings. There was no limit on the publication period, and only records published in English were considered. Records that did not fulfill the inclusion criteria were excluded.” (L104-110) |
| **3** | **Reviewer’s comments:** Results, Meta-analysis, Line 247: The wording of this sentence is unclear. Do the authors mean four studies provided data for inclusion in the effect size estimates for early initiation of breastfeeding? Similarly, my query also relates to the wording structure of the rest of the sentence.  **Authors’ response:** We have edited the sentence as follows to clarify the meaning. Yes, it meant how many studies were eligible for the meta-analysis of the respective IYCF components. For example, four studies were included in the meta-analysis when early initiation of breastfeeding was the outcome variable. Furthermore, we have omitted the following phrase related to effect size as this was already discussed in the method section.  **Action taken:**  “In total, four studies observing the early initiation of breastfeeding among employed mothers compared to their counterparts were included in the meta-analysis. Similarly, seven studies involving exclusive breastfeeding, and five studies involving complementary feeding were included.” (L245-L248) |
| **4** | **Reviewer’s comments:** Results, Meta-analysis, Line 249: Can the authors confirm whether original extracted data from the papers were used to calculate the pooled estimates? This is best practice, rather than using reported OR/RR/HR to calculate pooled estimates.  **Authors’ response:** Original data from the papers was not used to calculate the pooled associations. We have clarified this in the method section. We did not plan to use the original data for estimating pooled associations given that the variables adjusted in each study might be different. Furthermore, studies which were not nationally representative might not be published with raw data as supplementary file; therefore, we did not plan to contact with the corresponding authors for the raw data.  **Action taken:** In the methods, we have clarified the sources of effect estimates as follows.  “Effect estimates and their 95% confidence intervals reported in the studies were considered for meta-analysis.” (L235-136) |
| **5** | **Reviewer’s comments:** Results, Table 1: Since the study includes different types of observational studies, a column in the table should be included to indicate this information about each included paper.  **Authors’ response:**  We have used an additional column to represent the study design information.  **Action taken:** Column 3 of Table 1 shows the study design characteristics. (Table 1) |
| **6** | **Reviewer’s comments:** Formatting: Please check the font throughout the discussion as there are some inconsistencies, mostly occurring at the insertion points for references.  Please ensure the manuscript adheres to standard English conventions, including the writing of numbers under 10 as words, and numbers over 10 as numerals, e.g. Line 247, ‘4 effect sizes’ should be ‘four effect sizes’.  **Authors’ response:** Thank you for your suggestions. We have formatted the manuscript as per guideline and had the manuscript checked by native English speaker to improve language and readability.  **Action taken:** The manuscript has been formatted accordingly. The number below 10 were written in words. |

**Response to comments from Reviewer #2**

| **1** | **Reviewer’s comment:** This is a strong manuscript that merits publication. The following suggestions are shared to strengthen a few points and to address proofreading errors.  **Authors’ response:** Thank you for your comment. |
| --- | --- |
| **2** | **Reviewer’s comment:** The pooled analysis study results need to state more overtly that only EBF was statistically significant, whereas there was not a statistically significant difference by employment status for the other IYCF outcomes of interest. While p-values are now included, there is still a need to make this point clearly. The sentences that report results should signal this in the abstract and meta-analysis section. The first sentence of the discussion (lines 268-9) and also lines 372-3 should be reviewed and revised. The discussion of the divergent findings between studies is done well.  **Authors’ response:** Thank you for the suggestion. The issue of statistical significance has been highlighted in the abstract and meta-analysis sections. The suggested sentences have been edited accordingly.  **Action taken:**  In the abstract, the following phrase has been added to emphasize statistical significance.  “For example, there was a statistically significant lower likelihood of exclusive breastfeeding practice among employed mothers as compared to those who were not employed.” (L48-L50)  In the meta-analysis section, the following phrase has been added to emphasize the statistical significance.  “…., highlighting a statistically significant lower likelihood of exclusive breastfeeding among employed mothers than those who were not employed.” (L251-252)  The first sentence of the discussion is revised as follows.  “In this review we found maternal employment to be both a protective and detrimental factor of recommended IYCF practices in Bangladesh depending on the IYCF components. While the pooled association demonstrated maternal employment to be negatively associated with early initiation of breastfeeding and exclusive breastfeeding practices, maternal employment was also found to be a protective factor for recommended complementary feeding practices.” (L301-L305)  Sentence 372-3 in the previous version of the manuscript is rephased as follows.  “However, it appeared that maternal employment represented a statistically significant negative association with exclusive breastfeeding.” (L380-L381) |
| **3** | **Reviewer’s comment:** For data synthesis when ORs were not used, specify what was used to calculate them into ORs. If this was not done using raw data, this needs to be noted as a limitation.  **Authors’ response:** We have clarified in the methods section that if ORs were not reported in the article, we calculated it from the frequency distribution tables. Consideration of reported ORs over ORs synthesized from raw data has been mentioned in the limitation section.  **Action taken:** Following statement has been added to the methods section to clarify the consideration of reported ORs for meta-analysis.  “Effect estimates and their 95% confidence intervals reported in the studies were considered for meta-analysis.” (L135-L136)  Following sentence has been added to the limitation section.  “When conducting the meta-analysis, reported effect estimates (ORs) were considered rather than synthesizing ORs from the raw data.” (L368-L369) |
| **4** | **Reviewer’s comment:** Check pagination. After page 8, the page numbers restarted at 1.  **Authors’ response:** Thank you for the suggestion.  **Action taken:** The pagination has been synchronized accordingly. |
| **5** | **Reviewer’s comment:**  Address wording in the following:  -line 220, change 'rest one study' to 'remaining study'  -line 241-2, change 'private job' to 'private sector job'  -line 256, change 'week' to 'weak'  **Authors’ response:** Thank you for the suggestions.  **Action taken:** Wording has been addressed accordingly as follows.  ……remaining study found the opposite….  **……** mothers having private sector job…...  **…….** indicating a weak stability of the pooled effect size……. |
| **6** | **Reviewer’s comment:** Figure 3 is missing the red dashed reference line at 1 (this was included for Figures 2 and 4).  **Authors’ response:** Thank you for figuring it out.  **Action taken:** The reference line in Figure 3 has been added. (Fig 3) |
| **7** | **Reviewer’s comment:** On line 278, what was the basis for expecting that breastfeeding outcomes would be better for employed mothers? Most global literature has found consistently that employment is a barrier to EBF (see 2016 Lancet series, for instance). Consider rewriting this sentence in a more nuanced way, to reflect how literature on whether employment facilitates or hinders optimal IYCF depending on the outcome of interest. This is done well in the introduction section of the manuscript.  **Authors’ response:** We appreciate your suggestion.  **Action taken:** The sentences have been edited accordingly as follows.  “Engagement of women in the workforce appears to be a barrier to optimum breastfeeding practices, largely due to inadequate policy support, even though it is an important indicator of development.” (L310-L311) |
| **8** | **Reviewer’s comment:** My final question is about the location of the quality assessment section. I thought this would be better placed in the results section vs discussion. The limitations section can then reiterate any concerns in the discussion section. However, the authors can be guided by conventions and preferences regarding this point.  **Authors’ response:** Thank you.  **Action taken:** The quality appraisal part has been put along with the result section. |
