# Supplementary material for: How is maternal employment associated with infant and young child feeding in Bangladesh? A systematic literature review and meta-analysis
Source: PLoS One. 2025 Jan 24;20(1):e0316436. doi: 10.1371/journal.pone.0316436 (PMC11759378; doi:10.1371/journal.pone.0316436)
Supplement: Attachment — Submitted filename: Response to reviewers.docx [file pone.0316436.s004.docx]

**Response to academic editor and reviewers’ comments**

Highlighted phrases refer to the text in the revised manuscript.

**Response to comments from Reviewer #4**

| **1** | **Reviewer’s comment:** I have with great interest read the manuscript "How is maternal employment associated with infant and young child feeding in Bangladesh? A systematic literature review and meta-analysis". The manuscript is easy to read and was conducted through a rigorous method with the use of validated tools. This work addresses an important issue regarding the development of healthy feeding behavior in infant. Above all, I particularly appreciated the approach based on a socio-structural determinant of health (maternal employment) which is the most promising way to improve the health of populations and fight against social inequalities in health. This last aspect deserves to be highlighted in the manuscript. The authors propose concrete courses of action for public authorities. For these reasons, I approve the publication of the manuscript, with the following minor revisions considered. **Authors’ response:** Thank you for the nice words! We are happy to further edit the manuscript as you suggested. |
| --- | --- |
| **2** | **Reviewer’s comments:** Introduction: Start of introduction: Given that PLOS ONE is a general journal covering all domains of science and not specific to infant feeding behaviors, it would be useful to briefly remind readers of the WHO recommendations regarding breastfeeding and complementary feeding.  **Authors’ response:** Thank you for your suggestion. WHO recommendations sometimes vary with country specific recommendations, e.g., recommended duration of exclusive breastfeeding. So, we have provided an overview of IYCF guideline which is followed in Bangladesh context.  **Action taken:** Line 56-61 in the revised manuscript highlighted an overview of IYCF and its components for the readers. |
| **3** | **Reviewer’s comments:** Line 58. “In Bangladesh, 65% of infants aged less than 6 months are exclusively breastfed…”. This 65% value remains relatively high compared to other countries and is even higher than the global exclusive breastfeeding rate, which reached 48% in 2023 according to the Global Breastfeeding Scorecard from UNICEF and WHO. To better understand breastfeeding practices in Bangladesh, I suggest comparing this rate with those of other low- and lower middle-income countries. Another option would be to discuss the evolution of the exclusive breastfeeding rate in Bangladesh: Exclusive breastfeeding declines with age, from 85% among children age 0-1 months to 40% among those age 4-5 months (ref n°2) + exclusive breastfeeding rate at 6 months if you have this information. This wording would be more representative.  **Authors’ response:** Thank you. Our aim was to observe the association between maternal employment and IYCF practices in Bangladesh, regardless of whatever the IYCF status was. So, we did not dig it further down concerning which age group had better exclusive breastfeeding practices because it is likely that exclusive breastfeeding rate decreases as the children become older. Furthermore, national surveys, e.g., BDHS, also present the exclusive breastfeeding status among under-5 children not further delineated by age groups, so we could not provide the information accordingly. However, in the revised version, we have presented the exclusive breastfeeding status in some low- and lower-middle income countries.  **Action taken:** Exclusive breastfeeding practice in neighboring countries, India and Nepal, have been provided (Line 65-66 in revised version). |
| **4** | **Reviewer’s comments:** Lines 81-83. “Sub-optimum policy support related to maternity leave or lack of infant-friendly workplaces, including breastfeeding breaks, can lead to poor breastfeeding practices among employed mothers (18).” Considering the major influence of maternal leave policy and the close relationship with maternal employment status, it seems important to give more details of the specific situation in Bangladesh: how long is maternity leave? Are they well-paid? Is there parental leave (gender equity)?  **Authors’ response:** Thanks for bringing up the important issue. In that line (81-83), we aimed to present when and how maternal employment can be a risk factor of breastfeeding. However, in the revised version, the context of Bangladesh is highlighted. Yes, there is a paternity leave but not more than a week, so we omitted this in the manuscript.  **Action taken:** Line 89-92 in the revised manuscript highlighted the implementation of maternity leave in Bangladesh and how it is associated with IYCF. A reference (22) was also provided. |
| **5** | **Reviewer’s comments:** Line 91-93. “This could facilitate the implementation of IYCF policies and programs for both employed and unemployed mothers in Bangladesh to improve IYCF practices.” As mentioned above, addressing IYCF practices regarding maternal employment status (which is a socio-structural determinant of health) is a particularly relevant approach to reducing social inequalities in health. This aspect deserves to be highlighted in introduction and/or conclusion.  **Authors’ response:** In conclusion section, we have highlighted the type of policy support required for mothers falling behind in specific IYCF practices, e.g., breastfeeding and complementary feeding. Hope this helps.  “Support through policy and its implementation should be extended to employed mothers to allow them to practice exclusive breastfeeding during the first six months. Similarly, support should be given to unemployed mothers to allow for improved complementary feeding practices.” |
| **6** | **Reviewer’s comments:** Methods: Inclusion and exclusion criteria:  Line 104. “population (P) = mothers having an infant or a young child in Bangladesh”. What do you mean by ‘young child’, have you set an age limit?  “exposure (E) = mothers being employed in any public or private sector jobs”. Did you exclude independent jobs? (self-employed) “outcome (O) = status of IYCF practices” Not clear enough. Please provide some example in brackets (breastfeeding status, food offered, diversity, quality…)  **Authors’ response:** Thank you. Age range for infants and young children are 0-11m and 12-23m, respectively. Yes, self-employed jobs were also considered as private jobs, so we have highlighted this. The outcome component was clarified.  **Actions taken:** Line 114-122 highlight the changes. |
| **7** | **Reviewer’s comments:** Line 108. “…between the maternal occupation and IYCF practices”. Please replace ‘occupation’ by ‘employment status’ to be homogenous.  **Authors response:** Thank you. The word was replaced accordingly. (Line 119) |
| **8** | **Reviewer’s comments:** Line 109. “There was no limit on the publication period”. The introduction mentions changes in socioeconomic status of the population and women’s participation in the workforce over the last three decades. This could have been an argument for selecting articles since 1990. Why not set a date limit? Does the 1981 study (Jain) remain interpretable given the different context?  **Authors response:** We did not put any limit on publication period to obtain the number of evidence as many as possible. Furthermore, there were only few nationally representative surveys which yielded most of the articles. Yes, it is true that Jain 1981 is very old evidence, but it gives an idea about the association of the variables of interest in that time. Lastly, meta-analysis was carried out with only recent evidence. So, we believe putting no limit on the publication period did not affect the scientific rigor of study. |
| **9** | **Reviewer’s comments:** Results: Line 220. “Of the three studies investigating the association of maternal employment and breastfeeding duration ». It seems that article n°30 (Ayesha) have measured breastfeeding duration (maybe exclusive and/or mixed) and not only exclusive breastfeeding duration. In this case, please specify “..maternal employment and any breastfeeding duration” to be in agreement.  **Authors response:** Thank you! Breastfeeding duration or continued breastfeeding is also an outcome variable, which is clarified in the PICO in the revised version. |
| **10** | **Reviewer’s comments:** Line 233. “… employed mothers were more likely to provide complementary feeding than their unemployed counterparts”. This is not clear enough. Do you mean to provide adequate complementary feeding? Please add details.  **Authors response:** Thank you. We have changed the phrasing. It would be ‘recommended complementary feeding’ (Line 245). |
| **11** | **Reviewer’s comments:** Line 239. “… with government jobs demonstrated better performance than mothers having private sector jobs”. Similarly, this sentence is not clear enough, what do you mean by ‘better performance’, please provide some details.  **Authors response:** The phrase has been re-structured (Line 250-252). |
| **12** | **Reviewer’s comments:** Discussion: Line 301. “In this review we found maternal employment to be both a protective and detrimental factor of recommended IYCF practices in Bangladesh depending on the IYCF components”. The first sentence of the discussion is expected to precisely summarize the results. It would be better to specify the 'IYCF components': breastfeeding initiation and duration, MDD, and MAD. Also, please remain consistent in terms, IYCF components/indicators.  **Authors response:** Thank you! In the first sentence of discussion, we have concisely mentioned the IYCF components and their associations with exposure variable, maternal employment (Line 313-314). |
| **13** | **Reviewer’s comments:** Line 319. “In addition, cesarean delivery, which has been found to be a barrier to early breastfeeding in Bangladesh (43,54), was more common among employed mothers than unemployed mothers”. Do you have any explanation for this result? If so, please provide some details to better understand the mechanisms.  **Authors response:** Breastfeeding within 1 hour of birth is difficult for mothers who just had cesarean section delivery because of physical condition. In Bangladesh, the proportion of cesarean section is increasing and sometimes it is reported to be unnecessary. Furthermore, cesarean delivery in Bangladesh is usually paid by the mothers because of very low health insurance coverage. Therefore, employed mothers can better afford it. For the interested readers, we have provided references to our discussion so that they can further explore the mechanisms in detail (Line 328-334). |
| **14** | **Reviewer’s comments:** Line 324 “Considering the aforementioned findings, the conditions of employment in Bangladesh, coupled with suboptimal policy support…” Please replace “the conditions of employment” by “the maternal employment status”. Indeed, your review does not focus on employment conditions (e.g., job insecurity, flexibility, hours, physical strain etc.) because most of the selected studies explore employment in a dichotomous manner (working yes/no).  **Authors response:** Thank you! The sentence has been re-structured accordingly (Line 334-336). |
| **15** | **Reviewer’s comments:** Line 328. “In general, working mothers were found to have more control over food choices due to improved access through income. In addition, they were found to have better knowledge about child health and nutrition, which may lead to better complementary feeding practices”. What about the education level? This is known to be a strong determinant of health, and I suppose that Bangladeshi employed mothers are more educated than their unemployed counterparts. As you mentioned, this may imply a higher degree of health awareness, better health literacy, more resources to seek help, and better compliance with the existing recommendations but also access to high-skill jobs that promote work-life balance.  **Authors response:** Thank you! Education issue is highlighted accordingly (Line 339-340). |
| **16** | **Reviewer’s comments:** Strengths, limitations, and future scopes: I suggest adding a methodological limitation: the fact that the studies explore maternal employment in a dichotomous manner, which does not allow for a detailed understanding of employment conditions and stressors (e.g., flexibility, occupational exposure to chemical, type of contract, atypical schedules etc.). There is a need to better describe the characteristics of maternal employment. Unfortunately, this is rarely done in studies.  **Authors response:** Thank you for very important suggestions! We have revised the limitation section according to your suggestion (Line 380-384). |

**Response to comments from Reviewer #5**

| **1** | **Reviewer’s comment:** I believe the authors have address the reviewer comments and that further delays in publication are not necessary. That said, in addition to a final round of proofreading, I have a few suggestions the authors may want to consider.  **Authors’ response:** Thank you for the nice words. |
| --- | --- |
| **2** | **Reviewer’s comment:** 1. In the abstract, the final sentence could be more specific about breastfeeding-friendly workplace policies for employed mothers and could specify what policies may support unemployed mothers.  **Authors’ response:** Thank you. We have highlighted the issue in the abstract (Line 51). |
| **3** | **Reviewer’s comment:** 2. On p4, line 81, I wonder if the correct term is infant-friendly workplaces or breastfeeding-friendly? I'm more familiar with the latter, but this is not a sticking point.  **Authors’ response:** Thank you. We have changed the word to “breastfeeding-friendly” (Line 88). |
| **4** | **Reviewer’s comment:** 3. Either under the terminologies section or under each result area, consider adding the technical definitions of the different IYCF practices, such as early initiation, EBF, etc.  **Authors’ response:** Thank you. In the revised manuscript we have provided the definitions of IYCF components when they appeared first, e.g., early initiation of breastfeeding and exclusive breastfeeding in the introduction section (L58-L61) and complementary feeding in the methods section (L139-L144). |
| **5** | **Reviewer’s comment:** 4. Line 237, mention of 'association' should be 'associations'  **Authors’ response:** Thank you. The wording has been changed accordingly. |
| **6** | **Reviewer’s comment:** 5. Line 272, you can also cross-reference Table 1 now for the NOS scores, in addition to S1  **Authors’ response:** Thank you. Table 1 has been cited accordingly (Line 284). |
| **7** | **Reviewer’s comment:** Congratulations to the authors on an interesting paper that belongs in the public domain.  **Authors’ response:** Thank you very much! |
